# Supplementary material for: Explainable chemical artificial intelligence from accurate machine learning of real-space chemical descriptors
Source: Nat Commun. 2024 May 21;15:4345. doi: 10.1038/s41467-024-48567-9 (PMC11522690; doi:10.1038/s41467-024-48567-9)
Supplement: Supplementary file 1 — Supplementary Information [file 41467_2024_48567_MOESM1_ESM.pdf]

Supplementary Information: Explainable Chemical Artificial Intelligence From Accurate Machine Learning of Real-Space Chemical Descriptors. M. Gallegos et al.

Supplementary Note 1. SchNetPack (SPK) architecture

SchNet is a state-of-the-art DL architecture for learning quantum mechanical descriptors in the context of physics and chemistry (1; 2) such as energies and forces. Developed within the framework of Deep Tensor Neural Network (DTNNs), (3) it relies on the combination of a NN model and a continuous-filter convolutional approach as well as a built-in molecular featurization block. In contrast to discrete filters, continuous convolutional algorithms deal with heterogeneously sparse input features, such as the arbitrary positions taken by atoms in the molecular framework. Moreover, avoiding driscretization favors the smooth reconstruction of Potential Energy Surfaces (PES) while preventing discontinuities or abrupt changes in their topological features, key for applications such as MD simulations. (4) In SchNet, the continuity is achieved through NNs which model the function used to recover the filter values from the corresponding atomic positions (the continuous-filer convolutions). Very succinctly, i) the molecular representation is initialized from atomic embeddings, $\mathbf{x^0} = \mathbf{a}_Z$, ii) interaction blocks (**atom-wise** layers coupled to **cfconv** continuous filter-generators) refine the previous features by accounting for pair-wise interactions, iii) the updated input features are fed into dense **atom-wise** layers which output the atomistic predictions and iv) pooling layers are used to reconstruct the global properties. In this way, SchNet does not only learn the mapping between input features and target properties in the chemical space, but also the actual representation of the data, bypassing the chemical featurization problem. (5; 6) All of these features explain why, since its first proposal, SchNet has proven to be one of the most useful and general approaches for the construction of physical chemistry oriented ML models in many fields such as photochemistry, (7) phase transitions, (8) molecular design, (9) material science (10) or many-body interaction energies of weakly bounded clusters, (11) to name just a few. On the other hand, much effort has been devoted in recent years to reduce the gap between developing new ML architectures and building actual tailor-made tools with them. It is precisely within this context where SchNetPack (12) was derived as an open-source toolbox to aid the creation and implementation of ML models. SchNetPack is a self-contained PyTorch (13) package for the training of NNs targeted at the prediction of multiple chemical properties and, in particular, in the framework of molecular PES and ML force fields. It accounts for all the basic steps involved in the creation of new ML applications: architecture and model selection, chemical featurization (including the SchNet representation), training and evaluation of the models as well as actual production stages. Just as in the case of SchNet, SchNetPack has a pretty self-explanatory and intuitive design comprised by two main blocks:

- **Representation block:** it creates the input array (as Atomic Environment Vectors, AEVs) of the DL models starting from the atomic positions and types as given by the chemical featurization strategy of choice.
- **Prediction block:** it receives the previously computed AEVs which are fed into the AI models for their training, and subsequent performance evaluation, on the prediction of the target quantum chemical properties.

Altogether, the built-in utilities of SPK reduce enormously the cost required to produce accurate state-of-the-art quantum chemical ML models.

Supplementary Note 2. Source code (SchNet4AIM)

The current section gathers a summary of the modifications made to the original SchNetPack code (12) as well as the main features and architecture of the resultant toolbox. Before starting, it should be mentioned that the here presented version of SPK is targeted, exclusively, at the prediction of local descriptors and thus its performance has only been tested within the latter framework. Moreover, some of the additional features provided in the original SchNetPack toolbox have been disabled for the sake of convenience, as their compatibility with the prediction of atomic or inter-atomic properties has not been explored (being out of the scope of this work). Hence, unifying the original and modified SPK versions into a single toolbox able to handle molecular, atomic or inter-atomic properties remains for a future work.

Given that the prediction of atomic or inter-atomic quantities involves some fundamentally different strategies when compared to molecular observables, several changes had to be made to the architectures and modules:

Setting up the environment variables

For the sake of convenience, we have decided to make use of a collection of bash environment variables, defined by the user and read during the initialization of the SPK code, to control the behavior of the code:

- **SPK.AIMmode**: it is used to define whether the target local property is a one particle (1P) or a two-particle (2P) dependent quantity. This should be defined by the user before invoking the main SchNetPack modules. It can take the values "1p" or "2p".
- **SPK.dbmode**: it corresponds to the format of the database in which the input data is stored. Two different databases are currently available: JSON or ASE. The former is used in combination with a in-house implemented extended XYZ format which can be used for any molecular or local property (further details are provided in the upcoming sections). On the other hand, the latter is the one commonly used as default in the original version of SPK and it is only suited, in this context, for molecular or 1P local properties. This variable can take the values "db" or "json".
- **SPK.frset**: it is used to select the possible chemical constituents of the systems in the database, as gathered in the `elements` frozenset used by SchNet4AIM. The chemical elements should be specified by their corresponding atomic numbers (Z), as integers. This environment variable can be easily defined from a list of values (e.g `elem`) as:

```
elem       =  [1,8]
os.environ['frset'] = os.pathsep.join(str(i) for i in sorted(elem))
```

It should be noted that all these environment variables must be explicitly set before importing any of the SchNetPack modules.

Modules and main kernel files

Naturally, and besides including the aforementioned environment variables, some minor modifications were made to the source files to allow the architecture to deal explicitly with local quantities:

- **__init__.py**: the flag file used by the Python interpreter to identify that a given directory actually stores the main files of a module. This file gets implicitly executed when invoking a given package and thus it can be used to set some main attributes or objects upon initialization. A small block of code was included to read the bash environment variables (previously specified) so that the latter are locally defined within the SchNetPack framework.

```
AIMmode = os.environ['AIMmode']
DBmode  = os.environ['DBmode']
if ("frset" in os.environ):
   frset  = os.environ['frset'].split(os.pathsep)
   frset  = [int(item) for item in frset]
else:   # if the elements set has not been defined, the default CHON is used.
   elements = [1,6,7,8]
   os.environ['frset'] = os.pathsep.join(str(i) for i in sorted(elements))
   frset  = os.environ['frset'].split(os.pathsep)
```

- **SPK.atomistic**: this module contains some of the most fundamental blocks of SPK.

  For instance the **output_modules.py** section controls the output modules with which the quantum chemical descriptors are computed, and thus the specific way in which the final output is reconstructed from the NN predictions. The original version of SPK accounts for a collection of predefined output modules specifically oriented to the computation of different properties such as the `Atomwise`, `Polarizability` or `DipoleMoment` modules, to name a few. In this context, three additional modules have been developed:

  – 1) `AIMwise`: this output module is based on the original `Atomwise` one but it is instead designed to predict local properties and, thus, the output does not arise form the accumulation of particle-wise predictions. The default `sum` aggregation mode is replaced by a new one, named `atomic`, which invokes a dummy pooling layer (`DummyAggregate`) that removes the final aggregation step, commonly used in SchNet4AIM models. It should be noticed that the latter is the only possible aggregation layer than can be used in combination with the `AIMwise` module. The output of the model is now an array rather than a scalar, corresponding to the collection of particle-wise local values to be predicted. For the sake of simplicity and convenience, some additional features of the general output module have been removed, for instance the `self.derivative`, `self.stress` and `self.create_graph` attributes are (disabled) turned off. In this way, the main role of the output model is simply to set the output network along with the standardization and aggregation layers, required for running the predictions.

  – 2) `ElementalAIMwise (AIMwise)`: following an analogous strategy to that employed in the original `ElementalAtomwise` module, each chemical element is parsed through different NN models. In this way, atom-type specific predictions can be achieved. For a system containing M different chemical elements, a total of M different models will be built. Just as in the case of the `AIMwise` module, the `atomic` aggregation mode is used to avoid combining the individual outputs into a global property. Additionally, the elements frozenset (defining the chemical composition of the system) is given by a bash environment variable (**SPK.frset**) rather than been fixed internally, which increases the versatility of the model creation step.

  – 3) `ElementalPairAIMwise (AIMwise)`: this is equivalent to the `ElementalAIMwise` module, but this time each different pair of atoms is parsed through a pair-specific neural network model. For a system containing M different chemical elements, $M*(M+1)/2$ different models will be built, corresponding to the total number of non-equivalent atomic pairs. An atomic pair identifier is created using a unique scalar descriptor (named `indij`) which gets stored in a symmetric matrix (`pairmat`). The `indij` values are created as a vector of integers as follows:

```
indij=[]
elements = frozenset((spk.frset)
for i in np.arange(0,max(elements)):
    indij.append((i)*(2*max(elements)-i-1))
indij=np.array(indij,int)
```

  where `elements` is the frozenset which specifies the possible elemental composition of our system. It should be noticed that this approach will create unique identifiers for all the atomic pairs which can be formed with elements of Z number up to the maximum value of the frozenset (rather than just accounting exclusively for the explicit elements contained in the frozenset). On the other hand, the `pairmat` values can now be readily obtained by iterating through all possible atomic pair combinations (j $\geq$ i) with Z up to the previously specified maximum value of the frozenset, as:

```
pairmat = np.zeros((max(elements),max(elements)))
for i in np.arange(0,max(elements)):     # i = 1, n
    for j in np.arange(i,max(elements)): # j = i, n
        value = int(indij[i] + j+1)
        pairmat[i][j] = value
        pairmat[j][i] = value
```

  This leads to a symmetric matrix whose upper and lower off-diagonal components are unique. For instance let's consider a system made up of elements with Z values in the range of 1 (H) to 8 (O), then the `pairmat` matrix would take the following values:

```
1   2   3   4   5   6   7   8
2  16  17  18  19  20  21  22
3  17  29  30  31  32  33  34
4  18  30  40  41  42  43  44
5  19  31  41  49  50  51  52
6  20  32  42  50  56  57  58
7  21  33  43  51  57  61  62
8  22  34  44  52  58  62  64
```

As can be seen, this is an MxM symmetric matrix containing $M*(M+1)/2$ different values, offering a very suitable approach to uniquely identify any possible atomic pair combination, while holding the permutational invariance (pairmat[i,j] = pairmat [j,i]). On the other hand, and as far as the pooling layer is regarded, the `atomic` aggregation mode is used. Moreover, the architecture and features of the output network, as specific by `outnet`, are modified from the regular `GatedNetwork`, used in the `ElementalAIMwise` output module, to a new one (`GatedNetwork2P`) which receives as input the usual information in combination with the `pairmat` and `indij` values. Further details about the latter can be found in the `SPK.nn` block.

It is worth noticing that, whereas the `AIMwise` output module can be generally applied for both 1P and 2P properties, the particle-specific `ElementalAIMwise` and `ElementalPairAIMwise` models are only compatible with 1P and 2P descriptors, respectively.

Besides the **output_modules** section of `SPK.atomistic`, the **model** block was also modified. The latter contains a main class (`AtomisticModel`) which accounts for both the representation and prediction units involved in the training and evaluation of the NN models. Whereas the prediction block was essentially left unmodified with respect to the original SPK toolbox, new featurization schemes were included in the representation unit. In the particular case of atom-wise descriptors, the representation is obtained as usual, [`inputs["representation"] = self.representation(inputs)`], corresponding to a standard SchNet featurization. However, if two-particle properties are to be predicted, a pairwise descriptor must be employed instead. For such a purpose, atomistic SchNet4AIM representations are first computed for each atom in the system:

```
atomic_envs = self.representation(inputs)
```

Then, the inter-atomic distances are estimated on-the-fly:

```
dist = []
dist.clear()
for i in np.arange(int(inputs["npair"])):
    atomi = int(inputs["pari"][0,i])-1
    atomj = int(inputs["parj"][0,i])-1
    ri=inputs["_positions"][0,atomi]
    rj=inputs["_positions"][0,atomj]
    distij=torch.norm(ri-rj)
    dist.append(distij)
dist=np.asarray(dist,dtype=np.single)
dist=torch.from_numpy(dist)
```

And, finally, the featurization descriptor of each atomic pair is obtained form the concatenation of the corresponding atomic SchNet4AIM features and the inter-atomic distance.

```
dumconcat=[]
for i in np.arange(int(inputs["npair"])):
    dum=torch.cat((atomic_envs[:,int(inputs["pari"][0,i])-1,:],
                   atomic_envs[:,int(inputs["parj"][0,i])-1,:]),1)
    dumdist=torch.cat((torch.reshape(dist[i],(1,1)),tecla),1)
    dumconcat.append(dumdist)
dumconcat=torch.cat(dumconcat)
dumconcat=torch.reshape(dumconcat,(1,dumconcat.size()[0],dumconcat.size()[1]))
inputs["representation"] = dumconcat
```

Based on our findings, including the inter-atomic distance is absolutely crucial to achieve an accurate representation of the pair of particles. Otherwise, exclusively accounting for the SchNet4AIM atomic environment is unlikely to be able to uniquely characterize a given atomic pair. Indeed, many two-body local quantum chemical descriptors are known to be dominated, up to a large extent, by the inter-atomic distance. Moreover, such a finding is not unexpected as applying certain transformations on the input data is known to affect both the training times and accuracy, as for instance happens with the Fourier features. (14) Thus, we expect that the inclusion of certain terms that highlight in an explicit way some relationships between the variables characterizing a system and the properties to be predicted may be of great interest in this respect (e.g. terms of the form $1/R$ in the prediction of electrostatic components). We also note on passing that, in this case, the length of the representation vector is $[2n + 1]$, where n is the number of features used to expand the SchNet4AIM atomic environment. Hence, smaller values of n, when compared to those used for molecular or 1P local properties, should be employed to prevent excessively large featurization descriptors that could result in models with huge complexities, increasing the risk of overfitting.

- **SPK.data**: the **atoms.py** module has been updated to handle both database formats (JSON and ASE). As far as the former is regarded, a new function has been included which creates an ASE atoms object starting from a JSON database:

```
def json_2_ase_atoms(jsondb,idx):

    label=''.join(jsondb["ele"][idx])
    cords = jsondb["pos"][idx]
    ase_atoms=ase.Atoms(label,cords)

    return ase_atoms
```

The remaining part of the code proceeds essentially as usual if a DB database format is used. On the other hand, some of the functions are modified to account for the JSON format. For instance, the `get_properties` function contains a block of code to create an ASE object form the JSON database and extract the main properties found in the latter:

```
[IN] def get_properties(self, idx, load_only=None):
     # read database
     if (spk.DBmode == "json"):
        with open(self.dbpath) as jsonfile:
             jsondat=json.load(jsonfile)
        jsonfile.close()
        # create an ase object
        at=json_2_ase_atoms(jsondat,idx)
     # extract properties
     properties = {}
     properties.clear()
     .
     .
     if (spk.DBmode == "json"):
        for pname in load_only:
            properties[pname] = jsondat[pname][idx]
```

The `len` attribute of the database object is redefined for non-ASE databases:

```
def __len__(self):
    with open(self.dbpath) as f:
        dum = json.load(f)
    f.close()
    count=int(len(dum["name"]))

    return count
```

The _get_available_properties function contains a section to load the JSON database from the corresponding file and subsequently extract the keys (labels) of the properties:

```
[IN] def _get_available_properties(self, properties):
if (spk.DBmode == "json"):
 if os.path.exists(self.dbpath) and len(self.dbpath) != 0:
      with open(self.dbpath) as jsonfile:
           jsondat=json.load(jsonfile)
      jsonfile.close()
         atmsrw = conn.get(1)
      db_properties = list(jsondat.keys())
 else:
      db_properties = None
```

Finally, the `torchify_dict` function is modified to create PyTorch compatible tensors from other objects such as numpy arrays, invoked when loading the information from the JSON-like database. Additionally, some default properties (`ele`, `name` and `pos`) are dropped from the data for the sake of convenience:

```
[IN] def torchify_dict(data):
torch_properties = {}
if (spk.DBmode =="json"):
  data.pop("ele")
  data.pop("name")
  data.pop("pos")
  for pname, prop in data.items():
      prop=np.array(prop)
    if prop.dtype in [np.int, np.int32, np.int64]:
          torch_properties[pname] = torch.LongTensor(np.array(prop,float))
      elif prop.dtype in [np.float, np.float32, np.float64]:
          torch_properties[pname] = torch.FloatTensor(prop.copy())
```

Moreover, some minor changes were also included in the **stats.py** file, devoted to the computation and update of the statistics of the data.

```
n_batch = sample_value.shape[0]
n_atoms = sample_value.shape[1]  # Number of particles (atoms or atomic pairs)
# Update the cumulative variables with the samples.
for i in range(n_batch):
    if (spk.DBmode == "db"):
        for j in range(n_atoms):
            self._add_sample(sample_value[i, j, :])
    elif (spk.DBmode == "json"):
        for j in range(n_atoms):
            self._add_sample(sample_value[i,j])
```

By doing so, the atom- or pair-specific statistical variables can be computed.

- **SPK.nn**: it controls the architecture and main features of the neural networks employed in SPK. On a general basis, most of the routines and pieces of code were left unmodified with respect to the original version, however some additional classes were included in `SPK.nn/blocks.py` to account for the pairwise-specific networks to be used in the prediction of 2P properties:
  - `GatedNetwork2P`: this is a modification of the original `GatedNetwork` output network architecture. First, it determines the maximum number of elements and elemental pairs which can be found in the data:

```
self.nelem = len(elements)
self.npair = int(len(elements)*(len(elements)+1)/2)
```

    Notice that for the number of pairs, both homoatomic and heteroatomic scenarios are considered. Then, a new frozenset (`pairs`) is created, which stores the unique identifiers of all possible atomic pairs which can be found within our data:

```
pairs=[]
dummyelem=[]
for x in elements:
    dummyelem.append(x)
dummyelem=np.array(dummyelem,int)
for i in np.arange(0,len(elements)):
    for j in np.arange(i,len(elements)):
        pairs.append(int(pairmat[dummyelem[i]-1,dummyelem[j]-1]))
pairs=frozenset(tuple(pairs))
```

The previous information will be then parsed to the `ElementalGate2P` block.

– `ElementalGate2P`: this is a modification of the original `ElementalGate` output network architecture. It receives the previously computed information and creates a one-hot vector (tensor) describing the position of an atomic pair formed by atoms i and j in the `pairs` frozenset space. This is obtained by simply finding the atomic pair identifier attributed to the combinations of atoms i and j, as:

```
pari = inputs["pari"]
parj = inputs["parj"]
interatomic_numbers = []
for i in np.arange(0,len(pari[0,:])):
    interatomic_numbers.append(int(self.pairmat[atomic_numbers[0,pari[0,i]-1]-1
                                ,atomic_numbers[0,parj[0,i]-1]-1]))
interatomic_numbers = np.array(interatomic_numbers,int)
interatomic_numbers=torch.from_numpy(interatomic_numbers)
interatomic_numbers=torch.reshape(interatomic_numbers,(1,int(interatomic_numbers.size()[0])))
representation = inputs["representation"]
gated_network = self.gate(interatomic_numbers) * self.network(representation)
```

- **SPK.representation**: this block controls the featurization approach used to encode the chemical information of the data into a machine readable format. The **representation/hdnn.py** file was modified so that the element frozenset is defined starting from the list stored in the previously defined environment variable. The remaining blocks of code were left unmodified.
- **SPK.train**: the train module comprises the classes employed to control the basic training process of the SPK models. The **train/metrics.py** file was modified so that the tensors coming from the json database are reshaped to match the requirements of the remaining SchNetPack architectures.

```
if (spk.DBmode == "json"):
    y = torch.reshape(y,(y.size()[0],y.size()[1],1))
```

Additionally, and for the sake of convenience, only some basic metric classes have been enabled:

```
class ModelBias(Metric):
class MeanSquaredError(Metric):
class RootMeanSquaredError(MeanSquaredError):
class MeanAbsoluteError(Metric):
```

In this way, the MAE, MSE and RMSE metrics should be well defined and computed regardless of the database format and operating modes (1p or 2p) employed for the training process. Additionally, some modifications were made to the **loss.py** file so that the adequate loss function kernel is defined depending on the nature of the local target property:

```
if (spk.AIMmode == "1p"):
   def loss_fn(batch, result):
       loss = 0.0
       for prop, factor in zip(properties, loss_tradeoff):
           diff = batch[prop] - result[prop]
           diff = diff ** 2
           err_sq = factor * torch.mean(diff)
```

```
            loss += err_sq
        return loss
    elif (spk.AIMmode == "2p"):
        def loss_fn(batch, result):
            loss = 0.0
            for prop, factor in zip(properties, loss_tradeoff):
                diff = torch.reshape(batch[prop],(batch[prop].size()[0],
                                    batch[prop].size()[1],1)) - result[prop]
                diff = diff ** 2
                err_sq = factor * torch.mean(diff)
                loss += err_sq
            return loss
```

Uncertainty evaluation

As stated in the main text, one of the major criticisms of common ML models is the lack of proper uncertainty estimates accompanying the model predictions. In this regard, SchNet4AIM is in a particularly privileged spot owing, once again, to the rigor of the underlying real-space theories where it was proposed in the first place. As such, we can exploit the physics governing the local quantum chemical properties learned by SchNet4AIM to estimate the reliability of its predictions.

For instance, the expected value of the total number of electrons $< N >$ must be exactly reconstructed for each molecule. This can be formulated either in terms of the partial charges, that is:

$$\sum_{i=1}^{M} q_i = 0, \tag{1}$$

in the particular case of dealing with neutral molecules (as in our case), or in terms of the localized and delocalized electron populations, as measured by the localization (λ) and delocalization (δ) QTAIM metrics,

$$\sum_{i=1}^{M} \lambda_i + \sum_{i=1}^{M} \sum_{j>i}^{M} \delta_{i,j} = N, \tag{2}$$

where both summations run over the number of atoms of the system (M). Hence, the offset between the expected and the reconstructed molecular quantities provides an explicit measurement of the error of the SchNet4AIM cumulative output, which can be traced back to the individual one-body and two-body predictions.

Following such a reasoning, we have implemented two very simple functions to estimate the uncertainty of the built-in SchNet4AIM QTAIM predictions:

```
def calc_q_errors(q,atom_labels):
    """
    A function to compute the atomic errors in the reconstruction
    of the atomic charges (Q).
    """
    q=np.array(q,float)
    atom_labels=standarize_labels(atom_labels)
    zsum=0
    natoms=int(len(atom_labels))
    for elem in atom_labels:
        try:
            zsum += Z[elem]
        except KeyError:
            raise AssertionError ("Atom symbol not in the reference dictionary",elem)
    nelec = zsum-molec_q
    s4aim_charge=q.sum()
    s4aim_nelec = zsum-s4aim_charge
    s4aim_error= abs(s4aim_nelec-nelec)
    atomic_error=s4aim_error/float(natoms)
    return atomic_error
```

```python
def calc_li_di_errors(li,di,atom_labels):
    """
    A function to compute the atomic and pairwise errors in the
    reconstruction of the number of electrons from the LI and DI
    SchNet4AIM predictions.
    """
    zsum=0
    natoms=int(len(atom_labels))
    for elem in atom_labels:
        try:
            zsum += Z[elem]
        except KeyError:
            raise AssertionError ("Atom symbol not in the reference dictionary",elem)
    nelec = zsum-molec_q
    li=np.array(li,float)
    di=np.array(di,float)
    s4aim_nelec=li.sum() + di.sum()
    s4aim_error= abs(s4aim_nelec-nelec)
    local_error = s4aim_error/(natoms*(natoms+1)/2)
    return local_error
```

which have been included in the SchNet4AIM repository. As such, a typical SchNet4AIM prediction is provided with uncertainty bounds, as

```
 ----------------------------------------------------------------------------
 ATOMIC CHARGE (Q)
 ----------------------------------------------------------------------------
 Atom     1 (C ) q = +2.281 +- 0.001 : +2.280 to +2.282 electrons.
 Atom     2 (O ) q = -1.255 +- 0.001 : -1.256 to -1.254 electrons.
 Atom     3 (O ) q = -1.266 +- 0.001 : -1.267 to -1.265 electrons.
 Atom     4 (C ) q = -0.028 +- 0.001 : -0.029 to -0.027 electrons.
 Atom     5 (C ) q = -0.026 +- 0.001 : -0.027 to -0.025 electrons.
 Atom     6 (C ) q = +0.542 +- 0.001 : +0.541 to +0.543 electrons.
 Atom     7 (C ) q = +0.058 +- 0.001 : +0.057 to +0.059 electrons.
 Atom     8 (C ) q = -0.014 +- 0.001 : -0.015 to -0.013 electrons.
 Atom     9 (C ) q = -0.036 +- 0.001 : -0.037 to -0.035 electrons.
 ......
 ......
 ----------------------------------------------------------------------------
```

```
 ----------------------------------------------------------------------------
 ELECTRON LOCALIZATION INDEX (LI)
 ----------------------------------------------------------------------------
 Atom     1 (C ) li =  2.395 +- 0.001 : 2.394 to 2.396 electrons.
 Atom     2 (O ) li =  8.387 +- 0.001 : 8.386 to 8.388 electrons.
 Atom     3 (O ) li =  8.403 +- 0.001 : 8.402 to 8.404 electrons.
 Atom     4 (C ) li =  3.908 +- 0.001 : 3.907 to 3.909 electrons.
 Atom     5 (C ) li =  3.966 +- 0.001 : 3.965 to 3.967 electrons.
 Atom     6 (C ) li =  3.531 +- 0.001 : 3.530 to 3.532 electrons.
 Atom     7 (C ) li =  3.911 +- 0.001 : 3.909 to 3.912 electrons.
 Atom     8 (C ) li =  3.862 +- 0.001 : 3.861 to 3.864 electrons.
 Atom     9 (C ) li =  3.958 +- 0.001 : 3.957 to 3.959 electrons.
 ......
 ......
 ----------------------------------------------------------------------------
```

```
-----------------------------------------------------------------------------
ELECTRON DELOCALIZATION INDEX (DI)
-----------------------------------------------------------------------------
Pair     1     2 (C ,O ) DI = +1.263 +- 0.001 : +1.262 to +1.265 electrons.
Pair     1     3 (C ,O ) DI = +1.266 +- 0.001 : +1.265 to +1.267 electrons.
Pair     1     4 (C ,C ) DI = -0.006 +- 0.001 : -0.007 to -0.005 electrons.
Pair     1     5 (C ,C ) DI = -0.004 +- 0.001 : -0.005 to -0.003 electrons.
Pair     1     6 (C ,C ) DI = -0.004 +- 0.001 : -0.005 to -0.003 electrons.
Pair     1     7 (C ,C ) DI = -0.006 +- 0.001 : -0.007 to -0.005 electrons.
Pair     1     8 (C ,C ) DI = -0.005 +- 0.001 : -0.006 to -0.004 electrons.
Pair     1     9 (C ,C ) DI = -0.003 +- 0.001 : -0.004 to -0.002 electrons.
Pair     1    10 (C ,C ) DI = -0.007 +- 0.001 : -0.008 to -0.006 electrons.
Pair     1    11 (C ,C ) DI = -0.005 +- 0.001 : -0.006 to -0.004 electrons.
Pair     1    12 (C ,C ) DI = -0.006 +- 0.001 : -0.007 to -0.005 electrons.
Pair     1    13 (C ,H ) DI = -0.000 +- 0.001 : -0.001 to +0.001 electrons.
......
......
-----------------------------------------------------------------------------
```

In a similar way, it is possible to envision alternative kernels to estimate the accuracy of SchNet4AIM models trained to predict other properties. For instance, in the particular case of the energy, one could use the virial ratio of equilibrium geometries ($2T + V = 0$) to estimate the error of SchNet4AIM models trained on the kinetic and potential energy terms of equilibrium systems.

Supplementary Table. 1 gathers the estimated atomic and pairwise uncertainties in the SchNet4AIM computation of the QTAIM electronic metrics used as a prototypical model in this work. The results, corresponding to the best performing models, for different scenarios are shown.

Data	Uncertainty in Q	Uncertainty in λ and δ
QTAIM electronic database (Train)	0.00008	0.00001
QTAIM electronic database (Test)	0.00060	0.00005
Extrapolation domain (Chemical reaction)	0.00326	0.00300
Supramolecular domain (13-CO_2, 300K)	0.00927	0.00100
Supramolecular domain (13-CO_2, 900K)	0.00310	0.00102

Supplementary Table 1: **Uncertainties in the SchNet4AIM prediction of the QTAIM electron metrics** Estimated uncertainty in the estimation of the atomic charges, Q, along with the localization, λ, and delocalization, δ, indices of the best-performing SchNet4AIM models trained to predict the QTAIM electronic metrics. The results for different scenarios are shown. All values are reported, per atom or atomic pair, in electrons.

Supplementary Note 3. Additional database formats

Besides tuning the main architecture of the SchNetPack toolbox, predicting local quantities requires updating the database format used to parse the input data into the models. In particular, a completely new approach must be proposed for 2P dependent quantities, since the default ASE utility only allows molecular and atomic properties to be parsed. For the sake of convenience, we have proposed a new tailor-made extended XYZ format which can be used to encode any property (molecular, atomic or inter-atomic) into SPK:

\# N
\# name m_1 m_2 m_3 .... m_M
\# $label^1$ $r^1(3)$ $a^1{}_1$.......$a^1{}_A$
\# . . . .
\# . . . .
\# . . . .
\# $label^N$ $r^N(3)$ $a^N{}_1$.......$a^N{}_A$
\# $pair_i^1$ $pair_j^1$ i_1^1......i_I^1
\# . . . .
\# . . . .
\# . . . .
\# $pair_i^P$ $pair_j^P$ i_1^P......i_I^P

Where N is the number of constituting atoms in a molecular entity, with M molecular properties (m), A atomic properties (a) and I inter-atomic properties (i). The heading (first line) of the XYZ file is used to specify the number of constituting atoms in the system and the information about the molecular, atomic and interatomic properties is provided in subsequent blocks:

- 1) Molecular block: comprises the name of the system followed by the values of the M molecular properties in the system.
- 2) Atomic block: it contains a total of N lines (same as the number of atoms), each of them comprising the following information: atomic symbol (label), XYZ coordinates (in Å) and the values taken by the A atomic properties of a given atom.
- 3) Interatomic block: it contains a total of P lines, corresponding to the number of different atomic pairs present in the system. It should be mentioned that P can take two different values depending on whether the diagonal terms are taken into account, $(N * (N + 1)/2)$, or not, $(N * (N - 1)/2)$. These diagonal terms would generally correspond, from a practical point of view, to one-body properties and thus are not very often employed in the context of 2P descriptors. Each of the lines in this block contains the index identifiers of the atoms i and j that form a given atomic pair, followed by the values taken by the I inter-atomic properties for that pair. The numbering used in the index specification starts at 1, corresponding to the first atom in the system.

Hence, this simple extended XYZ format provides a straightforward and intuitive way to handle any global or local (1P or 2P) quantum chemical property of interest. Then, the latter file is stored in a JSON like database which can be then fed into the new version of the SPK code. It should be noticed however that, due to the way more efficient data handling, training the models with ASE databases is considerably faster than with its JSON analogs, so the latter should be preferably used only for 2P properties.

As an example, the following block shows an extended XYZ file for a water cluster $(H_2O)_2$ accounting for one molecular, atomic and inter-atomic properties:

```
          6
snap_100_2_trj     -152.848513
H    -2.655296  -3.184218  -6.741360        0.353031
H    -3.442233  -2.508277  -5.614466        0.365944
O    -2.850602  -3.251289  -5.794186       75.095746
H    -1.269583  -2.081152  -7.082810        0.350927
H    -0.782205  -2.533390  -8.502945        0.359913
O    -1.458094  -2.045788  -8.056412       75.089310
    1    2         0.135089
    1    3        -0.547684
    1    4         0.097329
    1    5         0.069869
    1    6        -0.180111
    2    3        -0.542737
    2    4         0.066622
    2    5         0.048091
    2    6        -0.113070
    3    4        -0.160300
    3    5        -0.110242
    3    6         0.245045
    4    5         0.133758
    4    6        -0.540801
    5    6        -0.547273
```

Supplementary Note 4. General computational details

The training and evaluation of the SchNet4AIM models were performed within a standard CONDA environment, running under a Python 3.8 interpreter on an Intel(R) Xeon(R) Silver 4114 CPU @ 2.20GHz computer equipped with 40 cores distributed over 2 nodes and a 128 GB RAM. Similarly, the GPU performance tests were run on a NVIDIA GeForce GTX 750 Ti GPU with 640 CUDA cores and 2GB of dedicated memory.

All electronic structure calculations were performed in the gas-phase with the aid of the Gaussian09 (15) and ORCA (16) packages, while the MD simulations were computed as implemented in ORCA. (16) For each database, a computational methodology capable of affording consistent reference data was selected. For the electronic properties, the level of theory was chosen to match that employed in the already existing database. (17) On the other hand, wide-range applicable DFT functionals, known to afford a trustworthy IQA decomposition of the energy of simple $(H_2O)_n$ clusters, (18) were employed to obtain the energetic reference data. QTAIM calculations were performed with the AIMAll (19) and PROMOLDEN (20) codes, whereas the latter was used to run the IQA partitioning (numerical integration parameters can be found in Supplementary Note 5).

Supplementary Note 5. Further details on the IQA calculations

The IQA partitioning of the energy was achieved with the PROMOLDEN code (20) (Version 1.81) without employing any explicitly symmetry options. The numerical integration was performed with the aid of β-spheres. In β-integrals were performed using 226 points, as Gauss-Chebychev radial quadratures, and 2905 angular Lebedev quadratures. On the other hand, integrations outside the β-spheres utilized 276 and 2905 radial and angular quadrature points, respectively. The maximum angular momentum (L) was set to 12 and 10 for out-β and in-β integrations. Similarly, the B3LYP DFT functional was employed throughout.

Supplementary Note 6. SchNet4AIM models

IQA energetic quantities

Unless otherwise specified in the manuscript, all SchNet4AIM models targeted at the prediction of IQA energetic quantities were trained until convergence using an initial learning rate of $5{\cdot}10^{-4}$ units with the Adam optimizer as implemented in PyTorch. The former was progressively reduced throughout the training with the aid of ReduceLROn-Plateau hook (patience=5, factor=0.8, min_lr=1e-6). All the input data was normalized with respect to the training subset. Moreover, as far as the SchNet4AIM representation is regarded, 25 gaussian functions were employed in combination with 3 interaction blocks and a cutoff radius of 5.00 Å using a CosineCutoff cutoff function as implemented in the original version of SchNetPack. (12) For 1P properties, a total of 128 SchNet4AIM features were computed to describe each atomic environment, whereas this number was reduced to 64 in the case of 2P quantities. The L2 metric was employed as a loss function while the L1 error was used to follow the performance of the model during the training procedure. In the case of 1P properties, an atomic-to-molecular tradeoff term (with a weight of 1% weight in the molecular quantities) was included in the loss function, whereas inter-atomic terms used, solely, the value of the local descriptors as loss metric.

QCT electronic properties

Unless otherwise specified in the manuscript, all SchNet4AIM models targeted at the prediction of QCT electronic quantities were trained until convergence using an initial learning rate of $5{\cdot}10^{-4}$ units with the Adam optimizer as implemented in PyTorch. The former was progressively reduced throughout the training with the aid of ReduceLROn-Plateau hook (patience=5, factor=0.8, min_lr=1e-6). All the input data was normalized with respect to the training subset. Moreover, as far as the SchNet4AIM representation is regarded, 25 gaussian functions were employed in combination with 4 interaction blocks and a cutoff radius of 5.00 Å using a CosineCutoff cutoff function as implemented in the original version of SchNetPack. (12) For 1P properties, a total of 128 SchNet4AIM features were computed to describe each atomic environment, whereas this number was reduced to 64 in the case of 2P quantities. The L2 metric was employed as a loss function while the L1 error was used to follow the performance of the model during the training procedure. In the case of 1P properties, an atomic-to-molecular tradeoff term (with a weight of 10% weight in the molecular quantities) was included in the loss function, whereas inter-atomic terms used, solely, the value of the local descriptors as loss metric.

Supplementary Note 7. Previously reported models (NNAIMQ)

For the sake of comparison, the performance achieved by SchNet4AIM in the prediction of local properties was compared to that afforded by previously reported models. Namely, the prediction accuracy and extrapolation abilities were compared against NNAIMQ, (17) which is, according to our knowledge, the only general-purpose ML model for the prediction of QTAIM atomic charges. Further details about the parameters employed in the creation of its chemical featurization descriptors and the training of the NNAIMQ models can be found elsewhere in the literature. (17)

Supplementary Note 8. Databases

The database of QTAIM electronic descriptors comprises a total of 45865 molecules accounting for, roughly, 10^6 atomic quantities and 10^7 inter-atomic descriptors. In the original work (17), 8065 molecules were randomly extracted from the latter to be used as testing dataset, whereas the remaining 37800 points were left for the actual training and validation purposes. However, and given that we are not interested in obtaining any model for actual production purposes, a 10% randomly selected subsample of the data will be used instead. The resultant 3865 data points were divided in training, validation and testing subsamples of 3100, 385 and 380 molecules, respectively, corresponding, roughly, to a 80-10-10 split.

Similarly, the database of the IQA energetic quantities, comprising a total of 1016 molecular instances, was divided in 750 training, 150 validation and 116 testing datapoints.

The following tables gather the single point geometries of the $(HCONH_2)_n$ clusters used for evaluating the evolution of the error in the reconstruction of the neutral molecular character predicted by the AI models with the size of the systems. All XYZ coordinates will be given in Å.

Atom	X	Y	Z
C	-2.55853	-0.56264	0.00000
O	-2.91903	0.60365	0.00000
N	-1.25296	-0.93915	0.00000
H	-3.25456	-1.41692	0.00000
H	-0.54797	-0.21360	0.00000
H	-0.96437	-1.90678	0.00000

Supplementary Table 2: $(HCONH_2)_1$ **cluster**

Atom	X	Y	Z
C	-2.38215	-1.09981	-0.98868
O	-2.52899	0.11215	-0.90974
N	-1.58200	-1.81396	-0.15731
H	-2.88660	-1.72478	-1.74310
H	-1.06722	-1.32618	0.58045
H	-1.46271	-2.81345	-0.22462
C	-0.39876	0.91355	1.88304
O	-0.25175	-0.29838	1.80396
N	-1.19893	1.62769	1.05168
H	0.10553	1.53849	2.63758
H	-1.31837	2.62716	1.11911
H	-1.71358	1.13994	0.31381

Supplementary Table 3: $(HCONH_2)_2$ **cluster**

Atom	X	Y	Z
C	-2.58085	-1.50368	0.26635
O	-2.74307	-0.34984	-0.10755
N	-1.38659	-2.02191	0.64216
H	-3.40323	-2.23414	0.33605
H	-0.54031	-1.44453	0.62738
H	-1.26983	-2.97203	0.96099
C	-1.61971	2.41530	2.65536
O	-0.59033	1.87748	3.03991
N	-2.38427	1.94615	1.64150
H	-2.01866	3.34285	3.09712
H	-2.13652	1.09586	1.14313
H	-3.22660	2.42636	1.32064
C	2.01458	-0.24753	1.32075
O	1.13313	-0.93495	0.82531
N	1.79003	0.77005	2.18814
H	0.83250	1.01519	2.45672
H	2.52632	1.33182	2.58780
H	3.08544	-0.39645	1.10716
C	-4.80493	2.72641	-0.82296
N	-4.29651	1.60844	-1.40082
H	-3.68594	0.99012	-0.86128
H	-4.48345	1.34958	-2.35798
O	-4.57192	3.05982	0.33044
H	-5.44764	3.30756	-1.50381

Supplementary Table 4: $(HCONH_2)_4$ cluster

Atom	X	Y	Z
C	-9.58341	2.05155	-0.97849
C	-4.43937	-1.85504	1.46557
C	-5.72937	4.68905	0.17962
C	-1.87668	-1.47661	-1.42870
C	-5.60019	0.46412	-1.67547
C	-6.62623	1.55622	1.71583
C	-1.35496	2.91684	-0.28025
C	-2.17773	0.62691	-4.40909
O	-7.24166	0.52893	1.45213
O	-8.63541	2.81520	-1.09666
O	-4.64125	4.67492	0.74049
O	-0.69967	2.01552	-0.78689
O	-1.10031	0.33150	-4.90246
O	-4.73225	1.32941	-1.68225
O	-2.83986	-2.22909	-1.51035
O	-3.86154	-0.77511	1.47623
N	-5.27650	1.62842	1.81175
H	-7.12105	2.52634	1.88465
H	-4.70357	0.79443	1.67069
H	-4.78763	2.50629	1.94550
N	-9.56800	0.94419	-0.19203
H	-10.54250	2.19080	-1.50366
H	-8.73705	0.72474	0.35785
H	-10.36750	0.33477	-0.09257
N	-5.78989	-1.97586	1.54658
H	-3.91951	-2.82411	1.39153
H	-6.35898	-1.13076	1.60105
H	-6.25544	-2.87120	1.55429
N	-1.73406	-0.57015	-0.43151
H	-1.06413	-1.43769	-2.17262
H	-2.43943	-0.50376	0.29890
H	-1.03027	0.16618	-0.46830
N	-5.35440	-0.86714	-1.75319
H	-4.39717	-1.22191	-1.75609
H	-6.08794	-1.55906	-1.71514
H	-6.67628	0.69436	-1.61631
N	-6.07571	3.79900	-0.78089
H	-7.03565	3.70249	-1.11618
H	-5.41325	3.09411	-1.09409
H	-6.53934	5.39201	0.43281
N	-2.70716	2.89028	-0.18917
H	-3.23482	2.10692	-0.56620
H	-3.24500	3.66358	0.20361
H	-0.91002	3.83103	0.14477
N	-2.31522	1.26560	-3.22109
H	-1.49271	1.52020	-2.68040
H	-3.22219	1.42742	-2.78943
H	-3.14521	0.38564	-4.87821

Supplementary Table 5: $(HCONH_2)_8$ cluster

Atom	X	Y	Z	Atom	X	Y	Z
C	-9.14459	3.43653	-1.08639	C	-9.46111	-1.14114	-0.06961
C	-5.17638	-2.93985	1.51047	C	-5.79350	6.12246	-2.40647
C	-4.92191	3.96099	0.91974	C	1.13309	0.25318	-3.30795
C	-1.04413	-1.13079	0.15782	C	-0.23602	2.69335	0.67508
C	-5.80546	0.21594	-2.06906	C	-2.66638	-4.74079	-0.50480
C	-6.23010	0.80845	2.58255	C	-6.66033	-4.50117	-1.42339
C	-2.90071	2.43891	-2.96116	C	-9.86142	2.52857	2.19816
C	-2.73202	-2.30444	-3.90628	C	-7.97747	6.40677	1.23525
O	-7.04720	-0.09661	2.68980	O	-9.81531	3.64816	2.68963
O	-8.27998	4.30239	-1.11585	O	-7.02861	7.08364	0.86090
O	-4.00903	3.30075	1.40255	O	-5.06530	5.57619	-3.22531
O	-2.44649	1.47126	-2.35767	O	0.60329	-0.42842	-4.17567
O	-3.83528	-2.51265	-3.41798	O	0.68974	2.32938	-0.03773
O	-6.24905	1.05434	-1.29393	O	-3.43709	-5.66656	-0.72106
O	-0.80561	-2.14097	0.80623	O	-7.81218	-4.10795	-1.29757
O	-4.66630	-2.04195	0.84994	O	-10.19188	-0.16357	0.04490
N	-5.02003	0.65930	1.99059	N	-10.98399	1.98301	1.66623
H	-6.40573	1.83230	2.95079	H	-11.85933	2.48464	1.69879
H	-4.75196	-0.24100	1.60233	H	-10.94771	1.07516	1.19965
H	-4.38967	1.44877	1.86620	H	-8.98558	1.86645	2.11282
N	-8.86993	2.11016	-1.10457	N	-8.00410	5.74301	2.41809
H	-10.22226	3.65418	-1.01140	H	-7.23402	5.82239	3.06500
H	-7.90668	1.78477	-1.17059	H	-8.77790	5.11956	2.66116
H	-9.58374	1.39771	-0.96905	H	-8.89136	6.25716	0.63793
N	-6.22057	-2.74598	2.35364	N	-5.32111	6.79370	-1.32584
H	-4.83883	-3.98815	1.47820	H	-4.32636	6.89777	-1.19153
H	-6.60467	-1.80647	2.47806	H	-5.94812	7.15222	-0.60212
H	-6.63744	-3.49194	2.89163	H	-6.89383	6.09987	-2.45588
N	-2.29715	-0.75503	-0.20276	N	0.42752	1.04322	-2.46305
H	-0.26330	-0.44509	-0.20421	H	-0.58274	1.09096	-2.53593
H	-3.09462	-1.30980	0.10079	H	0.85048	1.56782	-1.69439
H	-2.47682	0.05608	-0.78546	H	2.21911	0.27757	-3.12340
N	-5.31033	-0.98098	-1.67960	N	-1.53529	2.62424	0.29359
H	-5.28626	-1.24570	-0.69814	H	-1.77760	2.27040	-0.62419
H	-4.88437	-1.62837	-2.34658	H	-2.30770	2.92766	0.88723
H	-5.75490	0.36683	-3.15995	H	-0.10287	3.10442	1.68881
N	-5.89493	3.42357	0.14554	N	-1.91281	-4.64334	0.61773
H	-6.65234	3.98168	-0.25227	H	-1.92801	-5.37333	1.31375
H	-5.92128	2.42777	-0.07047	H	-1.33933	-3.81323	0.79272
H	-5.03452	5.04632	1.07062	H	-2.53655	-3.88452	-1.18610
N	-4.01808	3.09704	-2.57732	N	-6.18233	-5.61367	-0.81246
H	-4.55351	2.80418	-1.76885	H	-6.79348	-6.19026	-0.25385
H	-4.34346	3.94657	-3.04702	H	-5.19635	-5.87622	-0.88970
H	-2.42782	2.87316	-3.85748	H	-5.89874	-3.97995	-2.02580
N	-2.05716	-1.14026	-3.74878	N	-9.69980	-2.14648	-0.94604
H	-2.44924	-0.38153	-3.20276	H	-10.51494	-2.12853	-1.54050
H	-1.11289	-1.00065	-4.11639	H	-9.05088	-2.93199	-1.05132
H	-2.18154	-3.04533	-4.50860	H	-8.53699	-1.28476	0.51419

Supplementary Table 6: $(HCONH_2)_{16}$ **cluster**

Atom	X	Y	Z	Atom	X	Y	Z
C	-8.07398	3.16257	-1.26320	C	-9.16683	-0.40052	-0.92123
C	-4.90846	-1.95359	0.89689	C	-5.95612	6.81262	-2.90989
C	-3.28965	4.91652	1.19609	C	-0.52950	0.63704	-4.78605
C	-0.23921	-2.23323	0.93842	C	-0.20101	1.69515	-0.15758
C	-3.64003	1.60622	-2.07527	C	-2.31338	-4.78325	-0.69471
C	-5.19280	1.89648	2.19701	C	-6.82835	-5.44564	-1.78250
C	-6.12332	2.15994	-4.44091	C	-9.18668	2.27942	2.85434
C	-2.44410	-2.58031	-3.85656	C	-7.40013	5.78186	1.40929
O	-6.11386	1.11713	2.41793	O	-8.87231	3.24319	3.54000
O	-7.94142	4.05690	-2.09230	O	-6.55086	6.32812	0.71418
O	-2.32196	4.18233	1.34912	O	-5.27743	6.71477	-3.92522
O	-5.28441	1.54644	-5.08591	O	-0.99142	0.99376	-5.86412
O	-2.11769	-1.70665	-3.06361	O	0.16705	0.86533	-0.98005
O	-4.26426	2.43087	-1.41654	O	-3.09545	-5.17957	-1.54879
O	-0.60643	-3.07919	1.74237	O	-7.98738	-5.56092	-1.40335
O	-3.97849	-1.19485	0.64589	O	-9.71878	0.18159	0.00290
N	-3.98406	1.51087	1.72141	N	-9.93548	2.36027	1.72858
H	-5.26323	2.97980	2.37694	H	-10.29786	3.25717	1.40149
H	-3.81319	0.53540	1.48661	H	-10.10960	1.54156	1.14443
H	-3.25717	2.18944	1.51224	H	-8.87038	1.24811	3.08069
N	-7.04890	2.42310	-0.77376	N	-7.15190	5.23905	2.62572
H	-9.05054	2.87194	-0.84671	H	-6.23251	5.28405	3.06211
H	-6.08866	2.56512	-1.08697	H	-7.87179	4.70876	3.12128
H	-7.20221	1.69591	-0.08410	H	-8.44767	5.65794	1.09534
N	-6.00427	-1.57182	1.59994	N	-5.44728	7.19086	-1.71035
H	-4.95249	-3.00081	0.56063	H	-4.47599	7.45108	-1.62649
H	-6.06227	-0.63146	1.98832	H	-6.00856	7.16270	-0.85727
H	-6.75273	-2.22801	1.83069	H	-7.03153	6.57875	-2.86675
N	-1.09190	-1.49264	0.18910	N	-0.38935	1.45824	-3.71811
H	0.82004	-2.01288	0.72818	H	-0.68941	2.43144	-3.75270
H	-2.09860	-1.58251	0.30875	H	-0.05120	1.12138	-2.81570
H	-0.76074	-0.83569	-0.51466	H	-0.18344	-0.38999	-4.58557
N	-3.42779	0.32753	-1.68087	N	-0.69377	2.91248	-0.48386
H	-3.77388	-0.01902	-0.79050	H	-0.77754	3.19996	-1.45638
H	-2.88590	-0.33306	-2.24184	H	-1.10524	3.52467	0.22101
H	-3.20057	1.82689	-3.06114	H	-0.19118	1.51457	0.92989
N	-4.24196	4.70052	0.25680	N	-2.59725	-4.74869	0.63067
H	-5.07785	5.28172	0.20943	H	-3.49654	-5.07052	0.98368
H	-4.19791	3.88148	-0.35101	H	-1.94167	-4.33391	1.29432
H	-3.48071	5.81339	1.80703	H	-1.30684	-4.40155	-0.92932
N	-5.93745	3.42060	-3.98393	N	-5.76001	-5.48141	-0.95428
H	-5.08878	3.92463	-4.20821	H	-5.85891	-5.58992	0.05325
H	-6.63091	3.87729	-3.39615	H	-4.80158	-5.40309	-1.30048
H	-7.09843	1.73570	-4.15153	H	-6.54806	-5.30491	-2.83936
N	-2.91227	-2.30906	-5.09840	N	-7.92309	-0.92970	-0.83087
H	-3.02085	-1.35224	-5.40902	H	-7.41173	-0.91637	0.04326
H	-3.21672	-3.05146	-5.73377	H	-7.49463	-1.42772	-1.61292
H	-2.39023	-3.65926	-3.64158	H	-9.62316	-0.53865	-1.91515

C	-2.29882	4.82209	-3.06750	O	-10.78084	4.67099	0.36612
C	-1.49110	4.40919	-7.61003	H	-12.05946	5.94405	-0.61040
C	-9.67213	6.50872	-4.85988	O	-8.32319	-3.08803	1.97892
C	-6.17774	4.59210	-7.15073	H	-10.23948	-2.48050	1.57588
C	-11.06820	5.47259	-0.51283	O	-11.34222	-4.42259	-1.97888
C	-11.55015	-5.62951	-1.97489	H	-12.54679	-6.07839	-2.11456
C	-9.35828	-3.09260	1.32352	O	-5.16034	-5.39399	1.81832
C	-5.54788	-5.46859	2.97745	H	-5.00335	-6.01930	3.76193
C	-4.06598	5.52211	5.06831	O	-4.76722	5.09008	4.16343
C	-6.30415	2.47647	5.72734	O	-6.89518	-1.17184	9.22123
C	-7.38115	-3.87722	6.99455	O	-7.22678	-4.74946	6.15089
C	-6.84905	-0.01441	8.82804	O	-5.90775	2.82157	6.83537
C	-4.02772	-0.55024	-7.73221	O	-5.18864	-0.93803	-7.75480
C	-5.28841	-4.45829	-6.75226	O	-4.08364	-4.24459	-6.71619
C	-6.24403	-1.46000	-4.01284	O	-6.83792	-2.04051	-3.11084
C	-9.50895	-3.70369	-5.05775	O	-8.72449	-3.43194	-5.95773
O	-7.36361	4.71830	-6.87637	N	-3.56387	0.35015	-6.83055
O	-10.30470	7.00894	-3.93930	H	-4.21363	0.80842	-6.18630
O	-1.39138	4.00102	-3.03876	H	-2.59158	0.65104	-6.77610
O	-2.48730	4.65892	-6.94405	H	-3.24586	-0.92384	-8.41302
N	-8.92354	5.38525	-4.72891	N	-6.18881	-3.59890	-7.28774
H	-8.86127	4.89432	-3.84480	H	-5.87234	-2.70245	-7.65403
H	-8.38630	5.02096	-5.52102	H	-7.19016	-3.70815	-7.13660
H	-9.64302	6.92839	-5.87880	H	-5.75735	-5.35883	-6.32383
N	-5.19444	5.33023	-6.57746	N	-6.71979	-1.37779	-5.27757
H	-4.21101	5.16218	-6.78983	H	-7.55258	-1.89531	-5.54762
H	-5.41749	6.01386	-5.85932	H	-6.19182	-0.93936	-6.03141
H	-5.79623	3.86064	-7.88175	H	-5.28344	-0.93966	-3.87292
N	-2.93515	5.20908	-4.19825	N	-9.26503	-3.45329	-3.74798
H	-2.65116	4.84280	-5.10606	H	-8.38452	-3.02894	-3.46112
H	-3.68854	5.89690	-4.18783	H	-9.92689	-3.71314	-3.01750
H	-2.69044	5.31930	-2.16597	H	-10.48881	-4.17834	-5.22886
N	-0.73340	3.29546	-7.45528	N	-6.24532	1.19441	5.28639
H	-0.97584	2.59592	-6.75158	H	-5.85618	0.46796	5.87096
H	0.09217	3.11172	-8.00719	H	-6.48317	0.96123	4.32385
H	-1.11356	5.07599	-8.40244	H	-6.71730	3.17562	4.98410
N	-10.19515	5.86464	-1.47147	N	-4.12968	5.09252	6.35484
H	-10.45849	6.47516	-2.24881	H	-4.78880	4.35922	6.61238
H	-9.26330	5.46972	-1.51356	H	-3.54196	5.46744	7.08553
N	-9.53242	-3.83768	0.20626	H	-3.31064	6.31116	4.92020
H	-10.37690	-3.81245	-0.35934	N	-5.68768	0.64373	8.58705
H	-8.78832	-4.44119	-0.14115	H	-4.80264	0.19326	8.77071
N	-10.56915	-6.54789	-1.79650	H	-5.68379	1.58460	8.18906
H	-9.60801	-6.23104	-1.64344	H	-7.74270	0.59195	8.60659
H	-10.73777	-7.54340	-1.77489	N	-6.46271	-3.58422	7.94772
N	-6.67785	-4.88136	3.43751	H	-5.60333	-4.11067	8.00158
H	-7.27769	-4.33443	2.82310	H	-6.60563	-2.81224	8.60315
H	-6.94980	-4.93758	4.42121	H	-8.27578	-3.23559	7.04885

Supplementary Table 7: $(\mathbf{HCONH_2})_{32}$ **cluster**

Supplementary Note 9. Prediction of IQA energetic quantities

For the sake of simplicity, the label Elemental will be used to refer to ElementalAIMwise or ElementalPairAIMwise SchNet4AIM models in the case of 1P and 2P properties, respectively, throughout the upcoming sections. The following figures gather the dispersion plots (observed vs predicted data) for the SchNet4AIM models trained on the kinetic energy of the H atoms of the water cluster database (T^H).

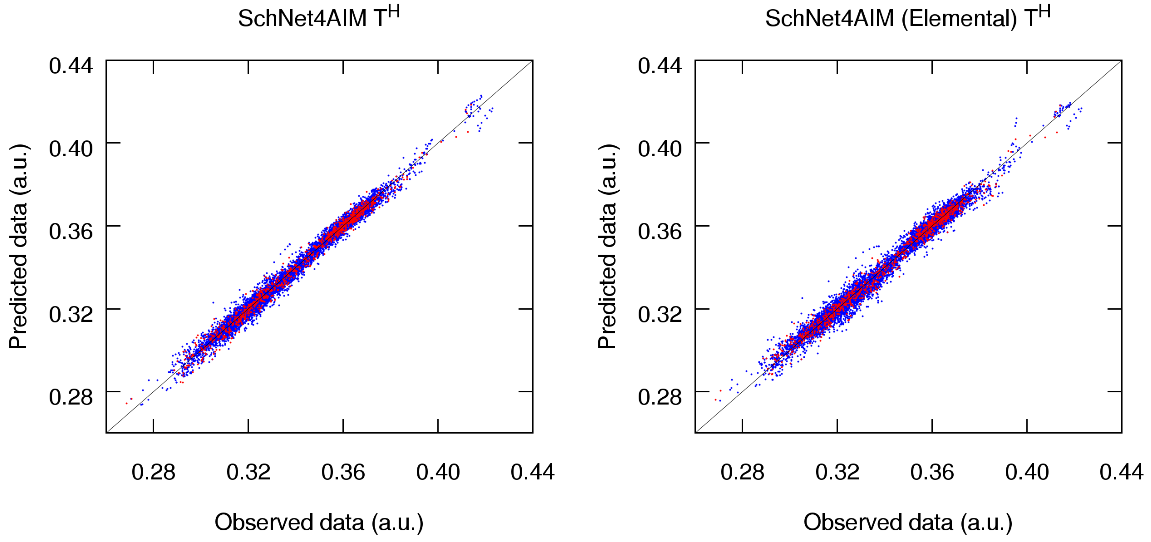

Supplementary Figure 1: T^H dispersion plots. Dispersion plot for the predicted kinetic energy contribution to the intra-atomic energy of the H atoms, T^H, of the water cluster database as estimated by different AI architecture models. The training and testing subsets are shown in blue and red, respectively. Source data are provided as a Source Data file.

On the other hand, the upcoming figures gather the dispersion plots for the SchNet4AIM models trained on the total IQA interaction energies between the H-H and O-O atoms of the water cluster database ($E^{\text{H-H}}_{inter}$ and $E^{\text{O-O}}_{inter}$).

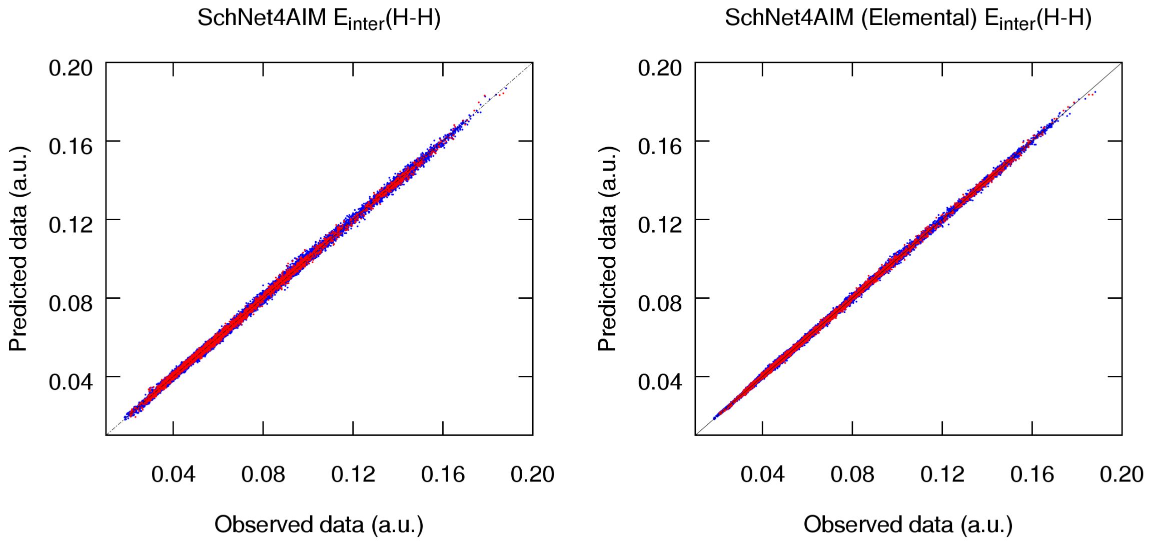

Supplementary Figure 2: $E^{\text{H-H}}_{inter}$ dispersion plots. Dispersion plot for the predicted H-H interaction energy, $E^{\text{H-H}}_{inter}$, of the water cluster database as estimated by different AI architecture models. The training and testing data points are shown in blue and red, respectively. Source data are provided as a Source Data file.

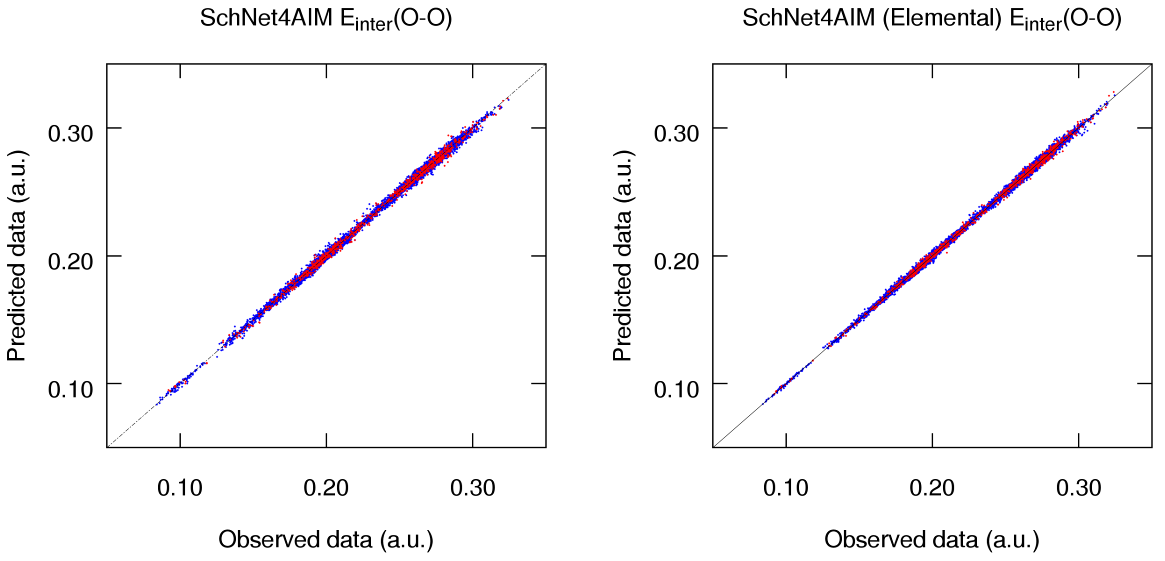

Supplementary Figure 3: $\mathbf{E^{O\text{-}O}_{inter}}$ **dispersion plots.** Dispersion plot for the predicted O-O interaction energy, $E^{O\text{-}O}_{inter}$, of the water cluster database as estimated by different AI architecture models. The training and testing data points are shown in blue and red, respectively. Source data are provided as a Source Data file.

Similarly, the following tables gather the performance metrics, reported in terms of the MAE and RMSE estimators of the different models trained on atomic and inter-atomic IQA quantities.

Property	MAE_{tr}	MAE_{ts}	$RMSE_{tr}$	$RMSE_{ts}$
T^{O}	0.003	0.003	0.004	0.004
T^{H}	0.002	0.002	0.003	0.003
$E^{O\text{-}O}_{inter}$	0.002	0.002	0.002	0.002
$E^{H\text{-}H}_{inter}$	0.001	0.001	0.001	0.001
$E^{O\text{-}H}_{inter}$	0.001	0.002	0.002	0.002

Supplementary Table 8: **SchNet4AIM (AIMwise) performance in the prediction of IQA quantities.** The labels *tr* and *ts* are used to refer to the training and testing datasets, respectively. The performance is reported in terms of the Mean Absolute Error (MAE) and Root Mean Squared Error (RMSE) metrics, given in atomic units (a.u.). The results for the kinetic energy of the O, T^{O}, and H, T^{H}, atoms as well as for the interaction energy of the O-O, $E^{O\text{-}O}_{inter}$, H-H, $E^{H\text{-}H}_{inter}$ and O-H, $E^{O\text{-}H}_{inter}$, atomic pairs are shown.

Property	MAE_{tr}	MAE_{ts}	$RMSE_{tr}$	$RMSE_{ts}$
T^{O}	0.003	0.004	0.005	0.005
T^{H}	0.002	0.002	0.003	0.003
$E^{O\text{-}O}_{inter}$	0.001	0.001	0.002	0.002
$E^{H\text{-}H}_{inter}$	0.001	0.001	0.001	0.001
$E^{O\text{-}H}_{inter}$	0.001	0.001	0.002	0.002

Supplementary Table 9: **SchNet4AIM (ElementalAIMwise or ElementalPairAIMwise) performance in the prediction of IQA quantities.** The labels *tr* and *ts* are used to refer to the training and testing datasets, respectively. The performance is reported in terms of the Mean Absolute Error (MAE) and Root Mean Squared Error (RMSE) metrics, given in atomic units (a.u.). The results for the kinetic energy of the O, T^{O}, and H, T^{H}, atoms as well as for the interaction energy of the O-O, $E^{O\text{-}O}_{inter}$, H-H, $E^{H\text{-}H}_{inter}$ and O-H, $E^{O\text{-}H}_{inter}$, atomic pairs are shown.

The following figure shows the evolution of the reference inter-atomic energy with the distance between the interacting atoms throughout the entire water cluster database. As can be seen, such a 2P quantity is mainly controlled by the inter-atomic distance.

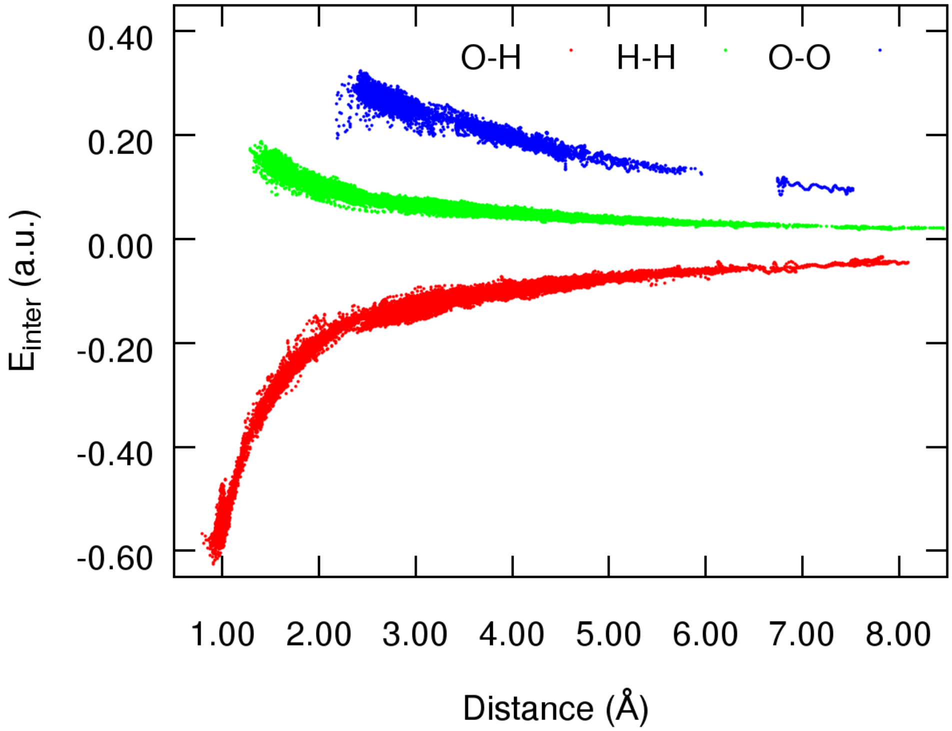

Supplementary Figure 4: **Evolution of E_{inter} with the inter-atomic distance.** Evolution of the interaction energy, E_{inter}, for the H-H, O-O and H-O bond pairs with the inter-atomic distance. The data corresponds to the water cluster database. Source data are provided as a Source Data file.

Supplementary Note 10. Prediction of QCT electronic properties

The current section gathers the results, given in terms of the error metrics and dispersion plots, obtained when training the models on the prediction of a collection of QCT electronic properties of a subset of the NNAIMQ database (17). All values will be given in atomic units (a.u.) unless otherwise specified. The following tables and figures comprise the results obtained when using a single NN output model for all the particle (atom or atomic pair) types, as given by the `AIMwise SPK.atomistic` output module.

Property	MAE_{tr}	MAE_{ts}	RMSE_{tr}	RMSE_{ts}
λ^{C}	0.008	0.009	0.011	0.013
λ^{H}	0.005	0.006	0.007	0.008
λ^{O}	0.011	0.013	0.014	0.018
λ^{N}	0.016	0.022	0.021	0.029

Supplementary Table 10: SchNet4AIM performance in the prediction of the localization index (λ). The labels *tr* and *ts* are used to refer to the training and testing datasets, respectively. The performance is reported in terms of the Mean Absolute Error (MAE) and Root Mean Squared Error (RMSE) metrics, given in electrons.

Property	MAE_{tr}	MAE_{ts}	RMSE_{tr}	RMSE_{ts}
Q^{C}	0.005	0.007	0.007	0.011
Q^{H}	0.005	0.005	0.006	0.007
Q^{O}	0.006	0.008	0.008	0.011
Q^{N}	0.008	0.014	0.011	0.019

Supplementary Table 11: SchNet4AIM performance in the prediction of the atomic charges (Q). The labels *tr* and *ts* are used to refer to the training and testing datasets, respectively. The performance is reported in terms of the Mean Absolute Error (MAE) and Root Mean Squared Error (RMSE) metrics, given in electrons.

Property	MAE_{tr}	MAE_{ts}	RMSE_{tr}	RMSE_{ts}
δ(C,C)	0.004	0.005	0.006	0.008
δ(C,H)	0.002	0.002	0.003	0.003
δ(C,O)	0.004	0.005	0.006	0.007
δ(C,N)	0.005	0.007	0.008	0.011
δ(H,H)	0.001	0.001	0.002	0.002
δ(H,O)	0.002	0.002	0.003	0.003
δ(H,N)	0.003	0.003	0.004	0.005
δ(O,O)	0.004	0.005	0.006	0.008
δ(O,N)	0.005	0.006	0.008	0.009
δ(N,N)	0.007	0.008	0.011	0.015

Supplementary Table 12: SchNet4AIM performance in the prediction of the delocalization index (δ(A,B)). The labels *tr* and *ts* are used to refer to the training and testing datasets, respectively. The performance is reported in terms of the Mean Absolute Error (MAE) and Root Mean Squared Error (RMSE) metrics, given in electrons.

Property	MAE_{tr}	MAE_{ts}	RMSE_{tr}	RMSE_{ts}
$\sum \lambda$	0.009	0.022	0.015	0.034
$\sum Q$	0.004	0.014	0.008	0.021
$\sum \delta$	0.037	0.049	0.053	0.076

Supplementary Table 13: SchNet4AIM performance in the reconstruction of molecular observables. The labels *tr* and *ts* are used to refer to the training and testing datasets, respectively. The performance is reported in terms of the Mean Absolute Error (MAE) and Root Mean Squared Error (RMSE) metrics, given in electrons. The results for the localized (λ) and delocalized (δ) electron counts, as well as the net molecular charge (Q), are shown.

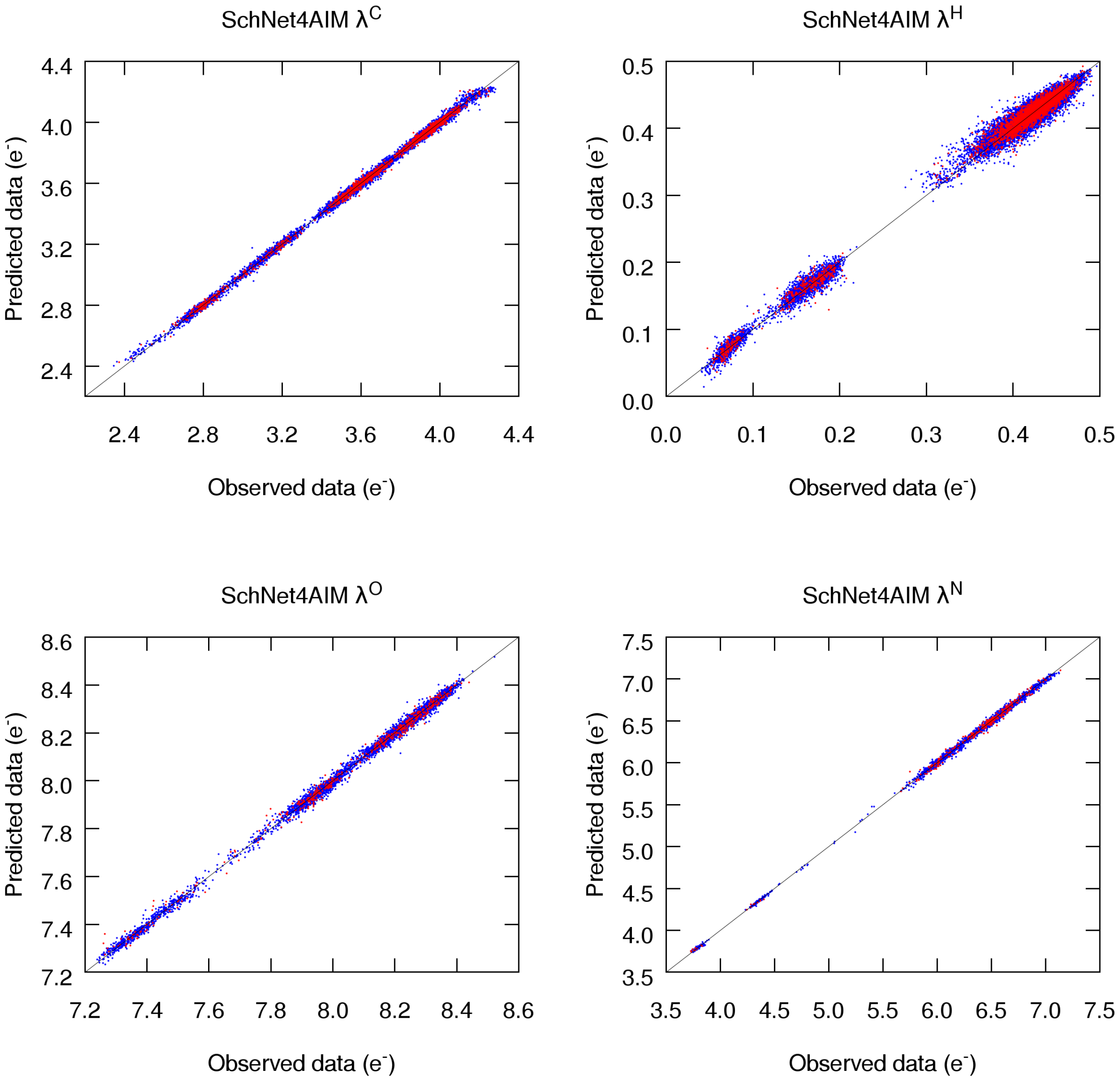

Supplementary Figure 5: **λ dispersion plots.** Dispersion plot for the predicted localization index (λ) of the NNAIMQ database as estimated by the SchNet4AIM models. The training and testing data points are shown in blue and red, respectively. All values are given in electrons (e^-). Source data are provided as a Source Data file.

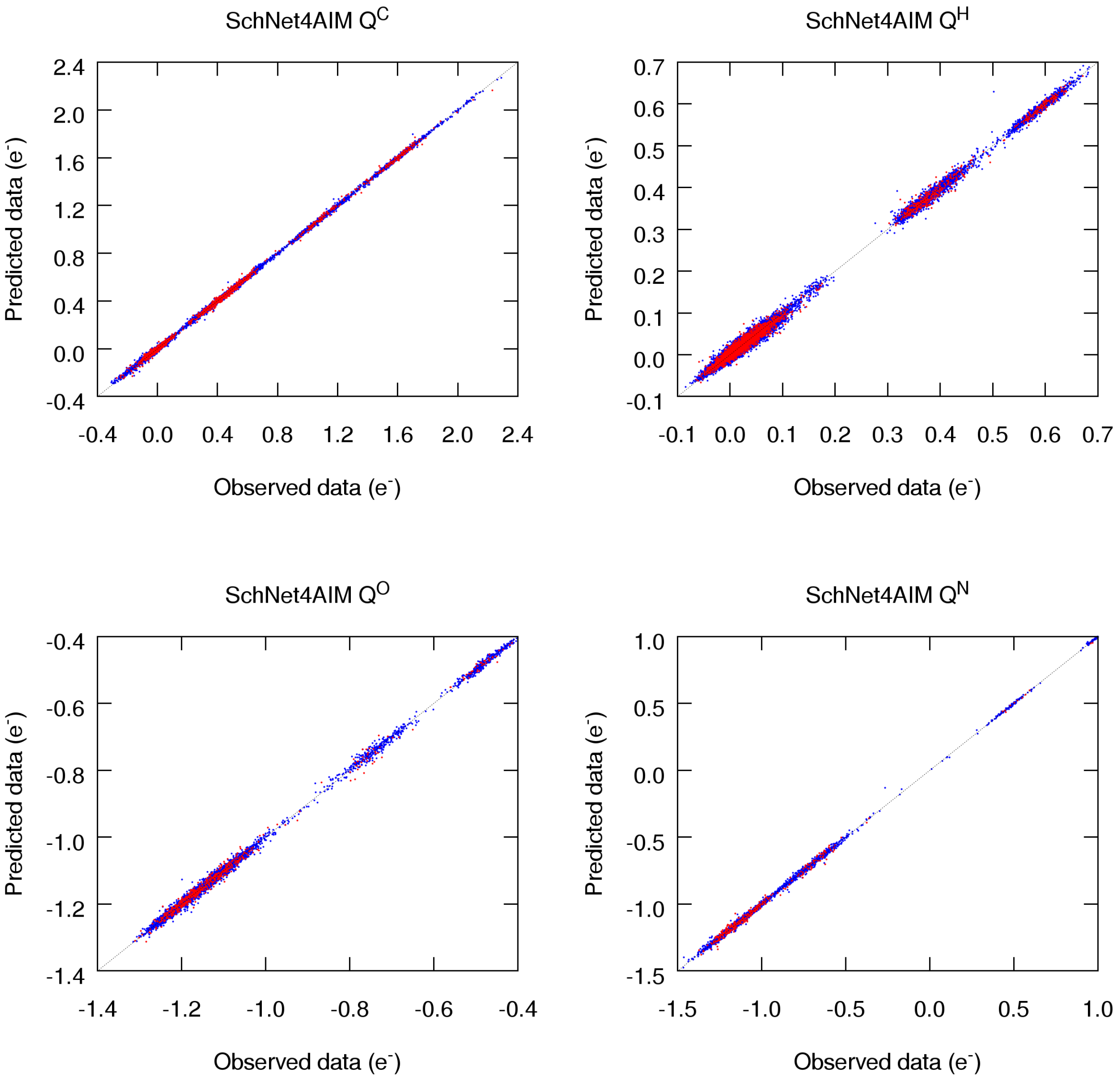

Supplementary Figure 6: ***Q* dispersion plots.** Dispersion plot for the predicted atomic charges (Q) of the NNAIMQ database as estimated by the SchNet4AIM models. The training and testing data points are shown in blue and red, respectively. All values are given in electrons (e^{-}). Source data are provided as a Source Data file.

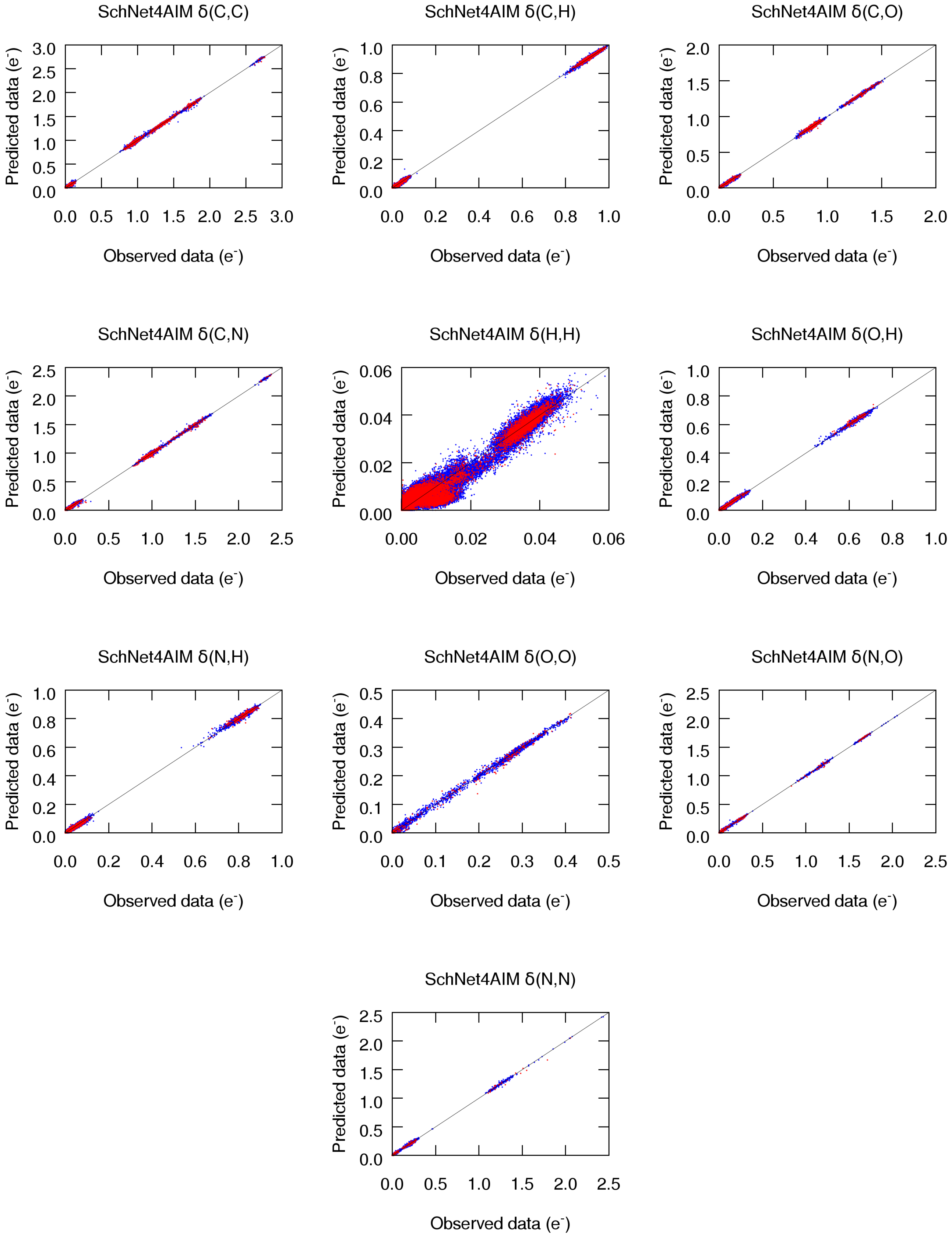

Supplementary Figure 7: **δ(A,B) dispersion plots.** Dispersion plot for the predicted delocalization index, δ(A,B), of the NNAIMQ database as estimated by the SchNet4AIM models. The training and testing data points are shown in blue and red, respectively. All values are given in electrons (e^-). Source data are provided as a Source Data file.

The following tables and figures comprise the results obtained when using particle-type specific NN models, as given by the `ElementalAIMwise` or `ElementalPairAIMwise` `SPK.atomistic` output modules.

Property	MAE_{tr}	MAE_{ts}	RMSE_{tr}	RMSE_{ts}
λ^{C}	0.007	0.008	0.009	0.011
λ^{H}	0.005	0.005	0.006	0.007
λ^{O}	0.009	0.011	0.012	0.015
λ^{N}	0.013	0.020	0.017	0.026

Supplementary Table 14: **SchNet4AIM (Elemental) performance in the prediction of the localization index (λ).** The labels *tr* and *ts* are used to refer to the training and testing datasets, respectively. The performance is reported in terms of the Mean Absolute Error (MAE) and Root Mean Squared Error (RMSE) metrics, given in electrons.

Property	MAE_{tr}	MAE_{ts}	RMSE_{tr}	RMSE_{ts}
Q^{C}	0.006	0.008	0.008	0.011
Q^{H}	0.005	0.006	0.007	0.008
Q^{O}	0.006	0.008	0.008	0.011
Q^{N}	0.009	0.014	0.012	0.019

Supplementary Table 15: **SchNet4AIM (Elemental) performance in the prediction of the atomic charges (Q).** The labels *tr* and *ts* are used to refer to the training and testing datasets, respectively. The performance is reported in terms of the Mean Absolute Error (MAE) and Root Mean Squared Error (RMSE) metrics, given in electrons.

Property	MAE_{tr}	MAE_{ts}	RMSE_{tr}	RMSE_{ts}
δ(C,C)	0.003	0.003	0.005	0.006
δ(C,H)	0.001	0.001	0.002	0.002
δ(C,O)	0.003	0.004	0.005	0.006
δ(C,N)	0.004	0.006	0.007	0.010
δ(H,H)	0.001	0.001	0.001	0.001
δ(H,O)	0.001	0.001	0.002	0.003
δ(H,N)	0.002	0.002	0.003	0.004
δ(O,O)	0.002	0.004	0.004	0.006
δ(O,N)	0.004	0.005	0.006	0.009
δ(N,N)	0.005	0.010	0.010	0.017

Supplementary Table 16: **SchNet4AIM (Elemental) performance in the prediction of the delocalization index (δ(A,B)).** The labels *tr* and *ts* are used to refer to the training and testing datasets, respectively. The performance is reported in terms of the Mean Absolute Error (MAE) and Root Mean Squared Error (RMSE) metrics, given in electrons.

Property	MAE_{tr}	MAE_{ts}	RMSE_{tr}	RMSE_{ts}
$\sum\lambda$	0.006	0.018	0.011	0.028
$\sum Q$	0.007	0.014	0.012	0.021
$\sum\delta$	0.026	0.036	0.039	0.053

Supplementary Table 17: **SchNet4AIM (Elemental) performance in the reconstruction of molecular observables.** The labels *tr* and *ts* are used to refer to the training and testing datasets, respectively. The performance is reported in terms of the Mean Absolute Error (MAE) and Root Mean Squared Error (RMSE) metrics, given in electrons. The results for the localized (λ) and delocalized (δ) electron counts, as well as the net molecular charge (Q), are shown.

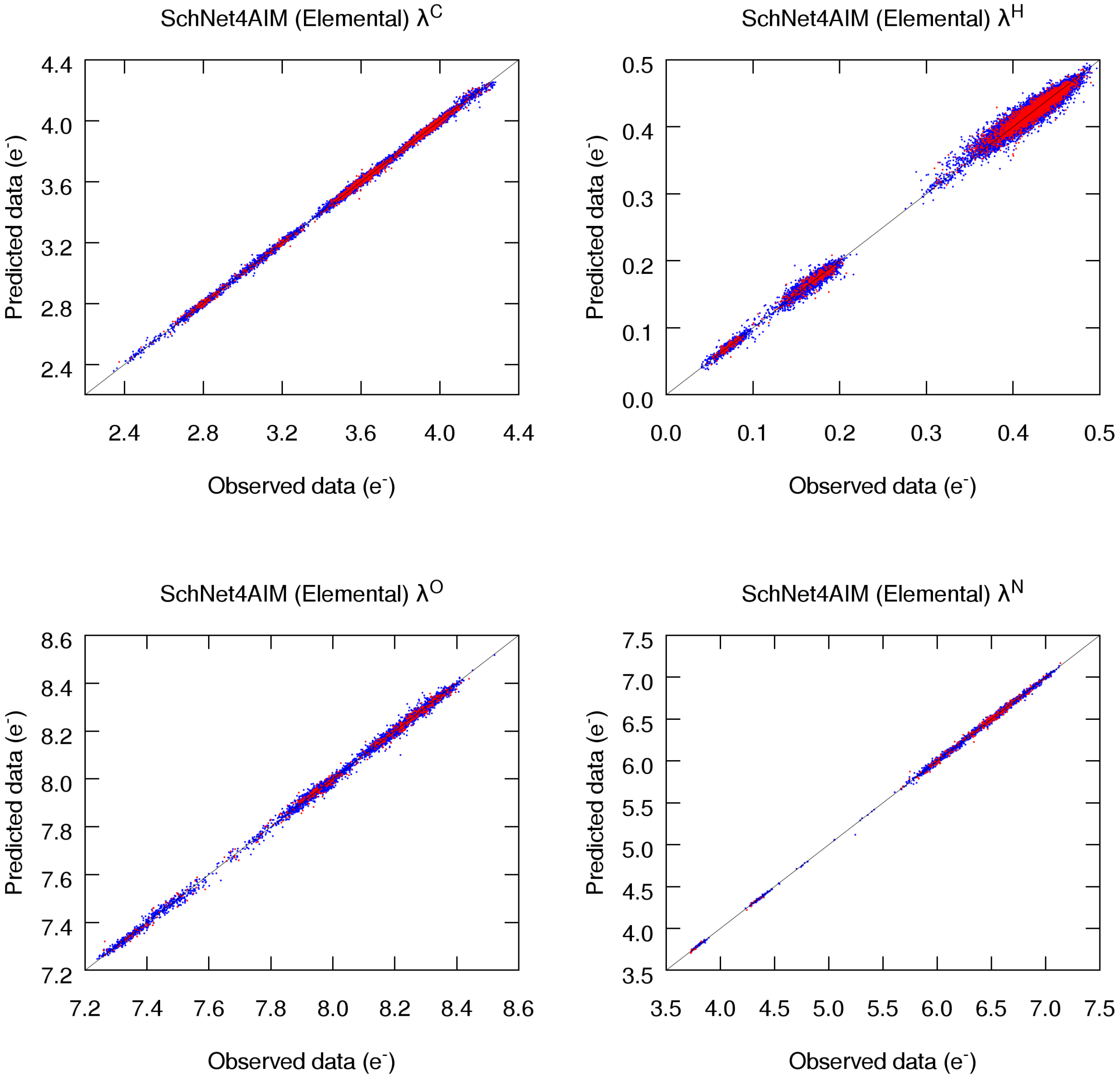

Supplementary Figure 8: **λ dispersion plots.** Dispersion plot for the predicted localization index (λ) of the NNAIMQ database as estimated by the SchNet4AIM-Elemental models. The training and testing data points are shown in blue and red, respectively. All values are given in electrons (e^-). Source data are provided as a Source Data file.

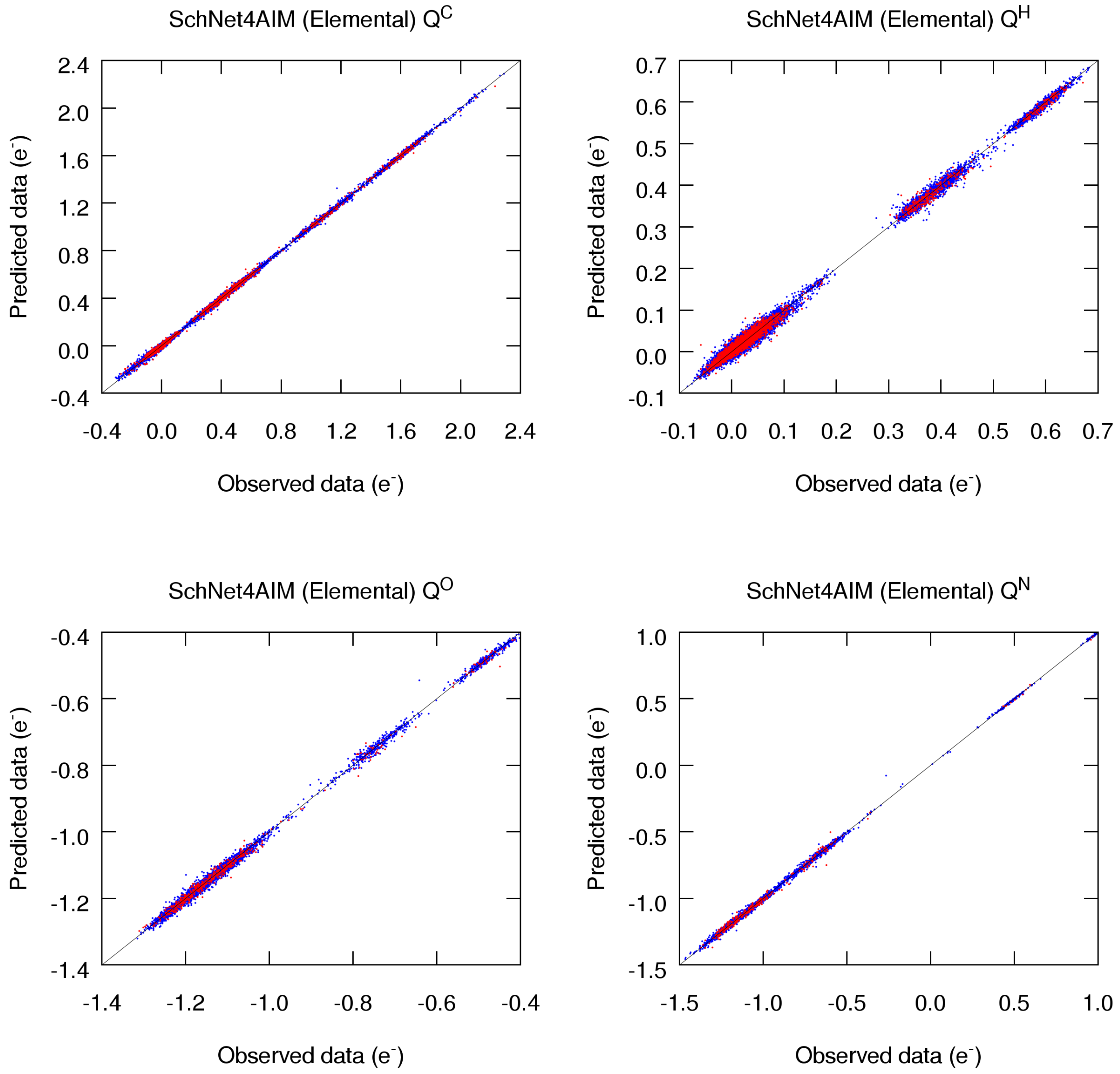

Supplementary Figure 9: ***Q*** **dispersion plots.** Dispersion plot for the predicted atomic charges (Q) of the NNAIMQ database as estimated by the SchNet4AIM-Elemental models. The training and testing data points are shown in blue and red, respectively. All values are given in electrons (e^{-}). Source data are provided as a Source Data file.

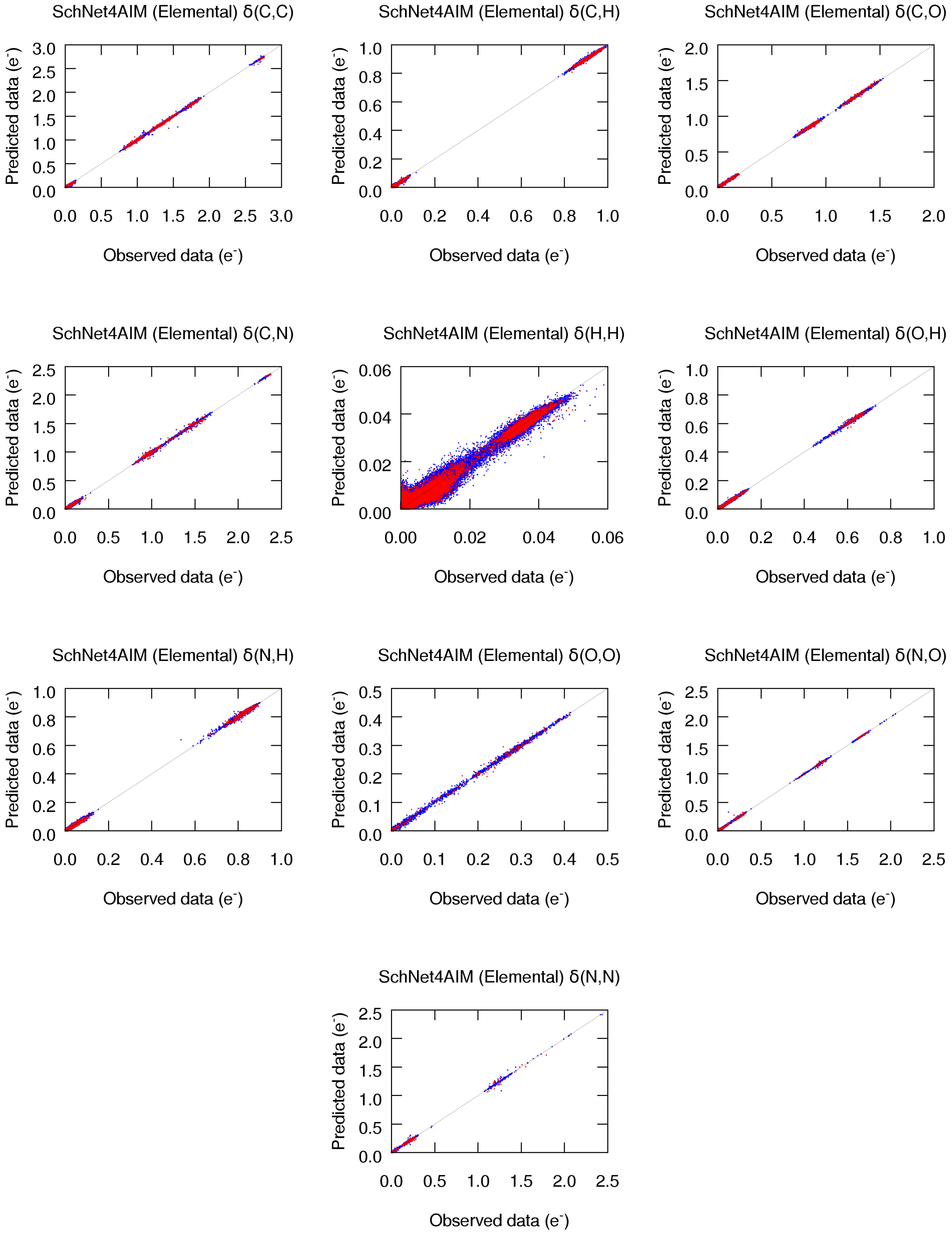

Supplementary Figure 10: **δ(A,B) dispersion plots.** Dispersion plot for the predicted delocalization index, δ(A,B), of the NNAIMQ database as estimated by the SchNet4AIM-Elemental models. The training and testing data points are shown in blue and red, respectively. All values are given in electrons (e^-). Source data are provided as a Source Data file.

The following figures gather the dispersion plot of the molecular properties as reconstructed by the SchNet4AIM models trained on the local quantities with different output modules.

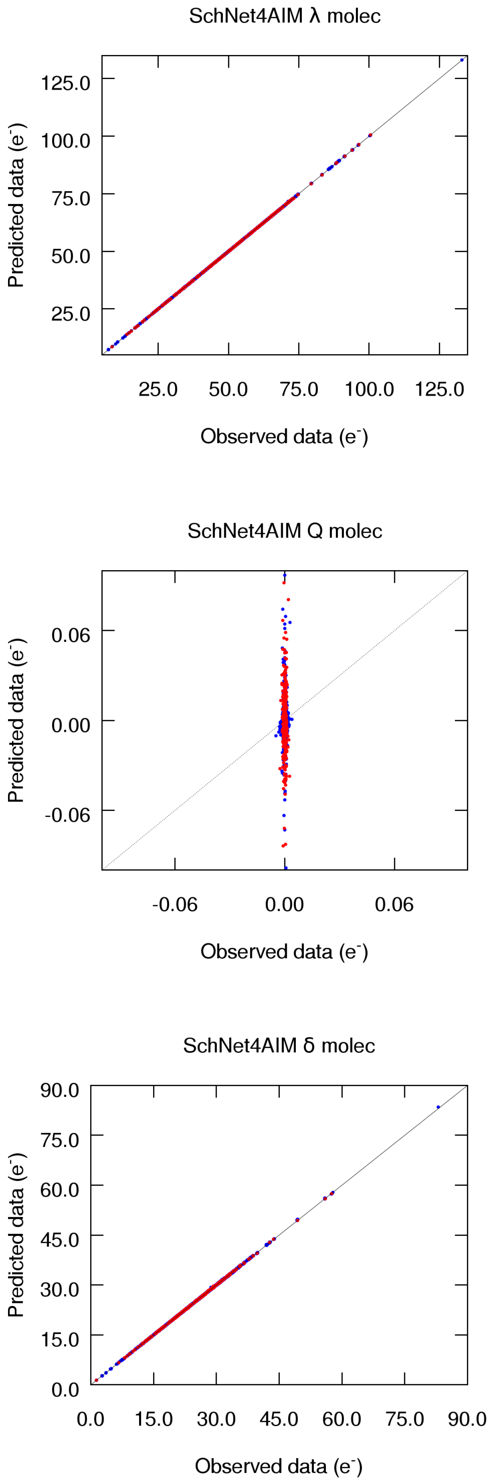

Supplementary Figure 11: **Dispersion plots for the SchNet4AIM reconstructed molecular observables.** The training and testing data points are shown in blue and red, respectively. The results for the localization index (λ), charge (Q) and delocalization index (δ) are shown. All values are given in electrons (e^-). Source data are provided as a Source Data file.

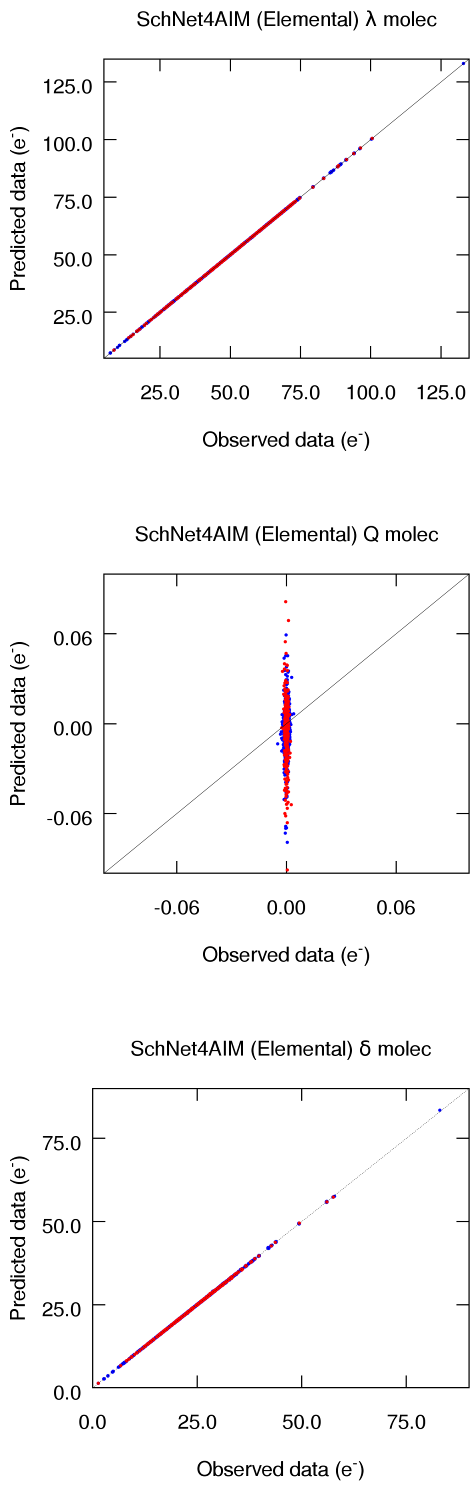

Supplementary Figure 12: **Dispersion plots for the SchNet4AIM (Elemental) reconstructed molecular observables.** The training and testing data points are shown in blue and red, respectively. The results for the localization index (λ), charge (Q) and delocalization index (δ) are shown. All values are given in electrons (e^{-}). Source data are provided as a Source Data file.

On the other hand, the upcoming figure shows a histogram of the distribution of the errors made in the estimation of the molecular charges of the training dataset by different NN models.

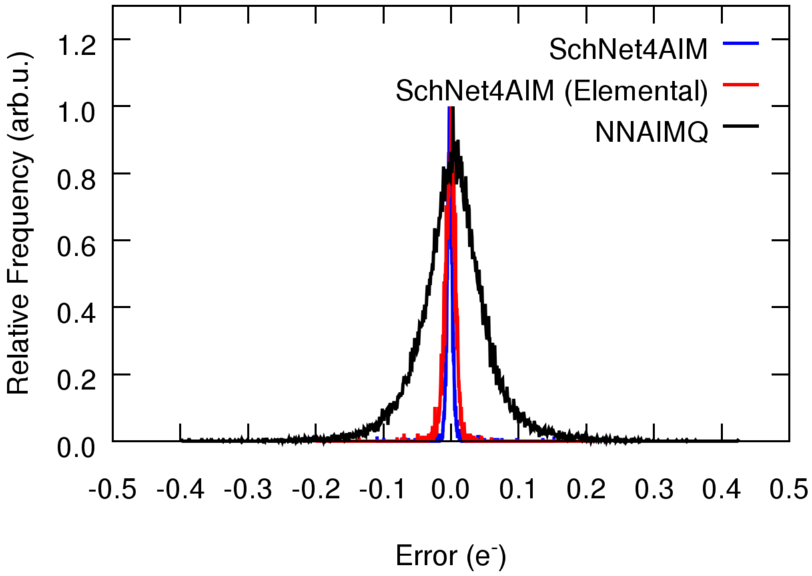

Supplementary Figure 13: **Error distribution in the estimation of molecular charges.** Distribution of the errors committed in the reconstruction of the electroneutral character of the molecules of the training dataset by different AI architectures. All values are given in electrons (e^-). Source data are provided as a Source Data file.

Supplementary Note 11. SchNet4AIM vs NNAIMQ for the prediction of atomic charges

This section comprises a comparison of the performance of the best-performing SchNet4AIM models with the previously reported attempt (17) for the prediction of the atomic charges of the NNAIMQ database.

As can be seen in Supplementary Table. 18, SchNet4AIM reduces significantly the MAE and RSME prediction errors resulting in narrowly scattered dispersion plots. Such a finding is quite remarkable given that the SchNet4AIM models are trained on $\approx 1/10$ fraction of the whole data employed by the original parent architecture (NNAIMQ) relying on a single NN to account for all the atom types as opposed to the 4 atomistic networks required by the latter. It is only in the case of N, where a more prominent offset between the predicted and observed data is found. This finding arises from the combination of a low abundance of heteroatoms in the database (17) and the large spectrum of values visited by Q^{N}, with a range of almost 3.0 electrons. Indeed, if measured on a relative scale, it will the H atoms the ones displaying slightly higher dispersions. Such a trend, reflected in most common $1\mathrm{P}^{\mathrm{H}}$ or 2P(H,H) terms, arises as a manifestation of the slightly larger uncertainty inherent to the estimation of QCT data of H atoms, being particularly problematic to compute.

Property	MAE (SchNet4AIM)	MAE (NNAIMQ)	RMSE (SchNet4AIM)	RMSE (NNAIMQ)
Q^{C}	0.007	0.010	0.011	0.015
Q^{H}	0.005	0.007	0.007	0.009
Q^{O}	0.008	0.008	0.011	0.011
Q^{N}	0.014	0.016	0.019	0.022

Supplementary Table 18: **Testing performance metrics.** Performance metrics, for the testing dataset, of the SchNet4AIM and NNAIMQ models trained to predict the atomic charges (Q) of the CHON database. The metrics for NNAIMQ were gathered from the literature. (17) The performance is reported in terms of the Mean Absolute Error (MAE) and Root Mean Squared Error (RMSE) metrics, given in electrons (e^-).

Besides the accuracy in the estimation of the local values, it is also worth exploring the ability of the ML models to recover the electroneutral character of the molecules, as measured by the $\sum_{i=1}^{M} Q$.

Property	MAE	RMSE
$\sum Q$	0.014	0.021
$\sum \lambda$	0.018	0.028
$\sum \delta$	0.036	0.053
$\sum Q$ (NNAIMQ)	(0.039) 0.063	(0.054) 0.082

Supplementary Table 19: **Error in the reconstruction of the molecular observables.** Error made by the SchNet4AIM models in the reconstruction of the molecular QCT electronic properties of the testing database. For the NNAIMQ models, the results for the training subset are shown between brackets. The performance is reported in terms of the Mean Absolute Error (MAE) and Root Mean Squared Error (RMSE) metrics, given in electrons (e^-). The results for the localized (λ) and delocalized (δ) electron counts, as well as the net molecular charge (Q), are shown.

As evidenced from Supplementary Table 19, collecting the error made in the estimation of the global quantities, SchNet4AIM appealingly reconstructs the molecular charges, showing training MAEs in the range of 10^{-3} electrons. The latter, being only one order of magnitude larger than the uncertainty bound of conventional QTAIM calculations (from 10^{-4} to 10^{-3} electrons), evidences the success of SchNet4AIM in the obtention of physically well-behaved partial charges. Actually, this performance is considerably superior than that afforded by some of our previously reported works (17), with MAEs of $\approx 10^{-2}$ electrons.

Further insights about the physical behavior of the resultant atomic charges can be achieved by analyzing the evolution of the global errors with the molecular size, as comprised in Supplementary Figure 14. For the sake of comparison, the testbed systems employed in the original work of the parent model (17) have been used, corresponding to a collection of progressively larger formamide clusters $(HCONH_2)_n$.Generally speaking, the errors increase with the size of the system as a result of the non-ideal canceling nature of the atomistic predictions, which can easily build up the global errors. Although this is found for both ML architectures, the accuracy of SchNet4AIM drops at a considerably lower rate than that of NNAIMQ. In fact, while the latter undergoes an outburst in the errors for $N > 40$, the former is able to afford reasonable results up to $N \approx 100$. This appealing result could be further improved after a fine-tuning of the SchNet4AIM representation (number of features, interaction blocks and cutoff radius), as the complexity of big systems inevitably requires more sophisticated input features for their adequate description. Altogether, the aforementioned results suggest that the SPK implementation paves the way towards not only more accurate, but better behaved, local properties.

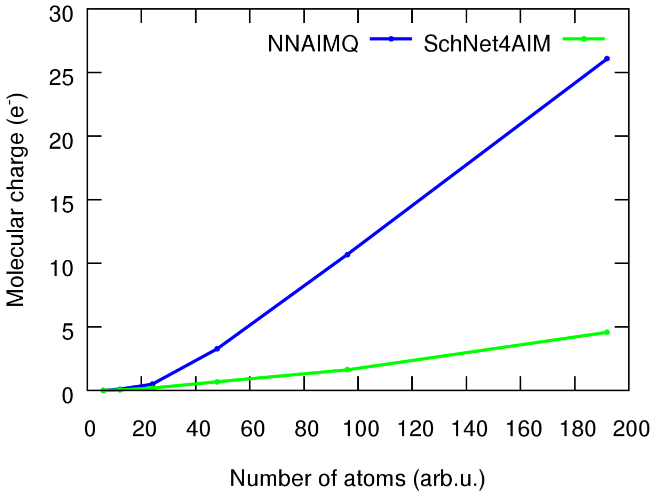

Supplementary Figure 14: **Offset in the reconstructed molecular charge with the size of the system.** Evolution of the error in the net molecular charge, as reconstructed from the atomistic predictions of the NNAIMQ and SchNet4AIM models, with the number of atoms in the formamide clusters. All values are given in electrons (e^-). Source data are provided as a Source Data file.

Supplementary Note 12. Performance in the prediction of the λ and δ metrics

The previously mentioned trends are, however, not exclusive to the atomic charges, but similar results are found for the λ and δ metrics. Actually, the former exhibits MAEs fairly close to those of the Q values, ranging between 0.005 and 0.020 electrons for the best- and worst-performing case scenario, respectively. Analogously, the δ values are estimated with high accuracy showing, on a general basis, narrow scatterings about the ideal prediction regimes. These prediction abilities are further evidenced by the low error metrics, with testing MAEs well below the 0.010 electron bound for most atomic pairs (see Supplementary Table 20). It is only in the particular case of δ(H,H) where the quality of the predictions gets noticeably diminished. The previously mentioned noise inherent to the computation of QCT properties of H atoms, accentuated by the fairly monotonous values visited by δ(H,H) (with a range of 0.06 e^{-}) inevitably decreases the performance of the models. These results could be further improved by introducing a penalizing term in the loss function to account for physically implausible values (e.g negative λ or δ values). This, however, is out of the scope of our work and should be addressed in the future.

Finally, the molecular localized and delocalized electron counts are accurately reconstructed by SchNet4AIM with training MAEs of 0.006 and 0.026 electrons, respectively. Considering the relatively large molecular delocalization values (with net DIs of up to $\approx$ 80 electrons), the prediction accuracy in the estimation of, even 2P, molecular observables is undeniable.

Property	MAE	RMSE
δ(C,C)	0.003	0.006
δ(C,H)	0.001	0.002
δ(C,O)	0.004	0.006
δ(C,N)	0.006	0.010
δ(H,H)	0.001	0.001
δ(H,O)	0.001	0.003
δ(H,N)	0.002	0.004
δ(O,O)	0.004	0.006
δ(O,N)	0.005	0.009
δ(N,N)	0.010	0.017

Supplementary Table 20: **SchNet4AIM (best-performing) performance in the prediction of the delocalization index (δ(A,B)).** Performance metrics, for the testing subset, of the SchNet4AIM models trained to predict the delocalization index (δ(A,B)). The performance is reported in terms of the Mean Absolute Error (MAE) and Root Mean Squared Error (RMSE) metrics, given in electrons (e^{-}).

Supplementary Note 13. Extrapolation abilities: chemical reaction

The extrapolation abilities of the models were tested with the archetypal chemical reaction between acetonitrile oxide and ethylene, corresponding to a 1,3-dipolar cycloaddition, considered to proceed by means of quasi-concerted mechanism. For the sake of simplicity, we will refer to the progress of the reaction coordinate by means of a collection of integers (χ, in arb.u.), corresponding to the single point calculation performed along the Intrinsic Reaction Coordinate (IRC) path. Under this notation, the transition state (TS) is located at χ =25. Single point calculations were performed, in the gas phase, at the M06-2X/def2-TZVP level of theory throughout the IRC path of the reaction, with the aid of the Gaussian09 quantum chemistry package (15). The latter was computed starting from the optimized structures of the reactant complex and transition states, as given in the original work of the parent NNAIMQ model (17). Finally, the electronic descriptors were computed with the aid of the AIMAll (19) and `PROMOLDEN` (20) codes.

The following figure shows the evolution of the error in the reconstruction of the neutral molecular character, as predicted by the NNAIMQ and SchNet4AIM models, throughout the progress of the reaction.

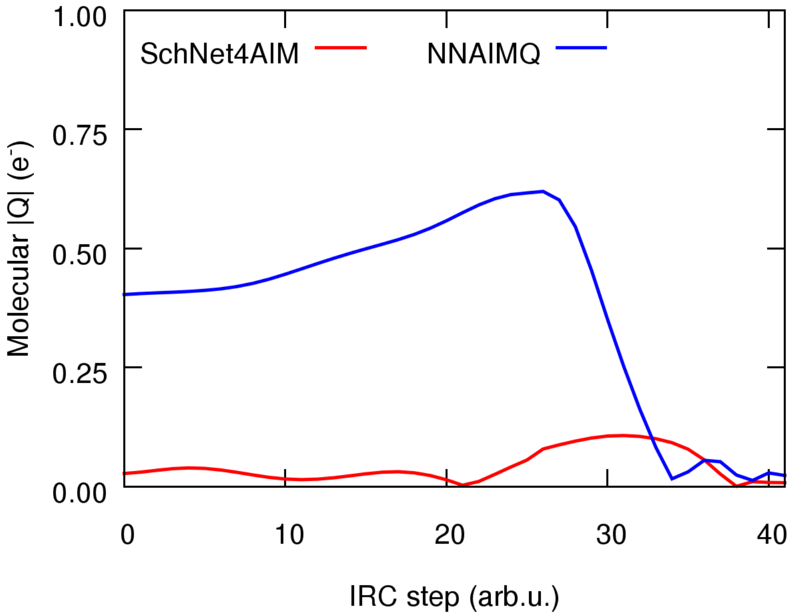

Supplementary Figure 15: **Reconstructed molecular charge throughout the reaction.** Evolution of the absolute error in the reconstruction of the neutral molecular character, |Q|, throughout the reaction, reported in electrons (e^-), as predicted by the NNAIMQ and SchNet4AIM models. The progress of the reaction is reported in terms of integer units (i.e steps) across the intrinsic reaction coordinate (IRC). Source data are provided as a Source Data file.

Similarly, the following table gathers the accuracy, as measured by the MAE and RMSE metrics, of the NNAIMQ and SchNet4AIM estimations of the atomic charges of the main atoms involved in the chemical reaction.

Atom	MAE (NNAIMQ)	RMSE (NNAIMQ)	MAE (SchNet4AIM)	RMSE (SchNet4AIM)
1	0.134	0.165	0.035	0.040
2	0.164	0.173	0.031	0.038
3	0.053	0.063	0.023	0.026
4	0.048	0.066	0.015	0.018
7	0.016	0.022	0.057	0.062

Supplementary Table 21: **SchNet4AIM and NNAIMQ performances in the prediction of the atomic charges throughout a chemical reaction.** Mean Absolute Error (MAE) and Root Mean Squared Error (RMSE) metrics, in electrons, committed by the NNAIMQ and SchNet4AIM models in the prediction of the QTAIM partial charges of a collection of atoms throughout the progress of the reaction.

The following figure comprises the evolution of the atomic charges of the main atoms involved in the reaction, as predicted by the NNAIMQ and SchNet4AIM models.

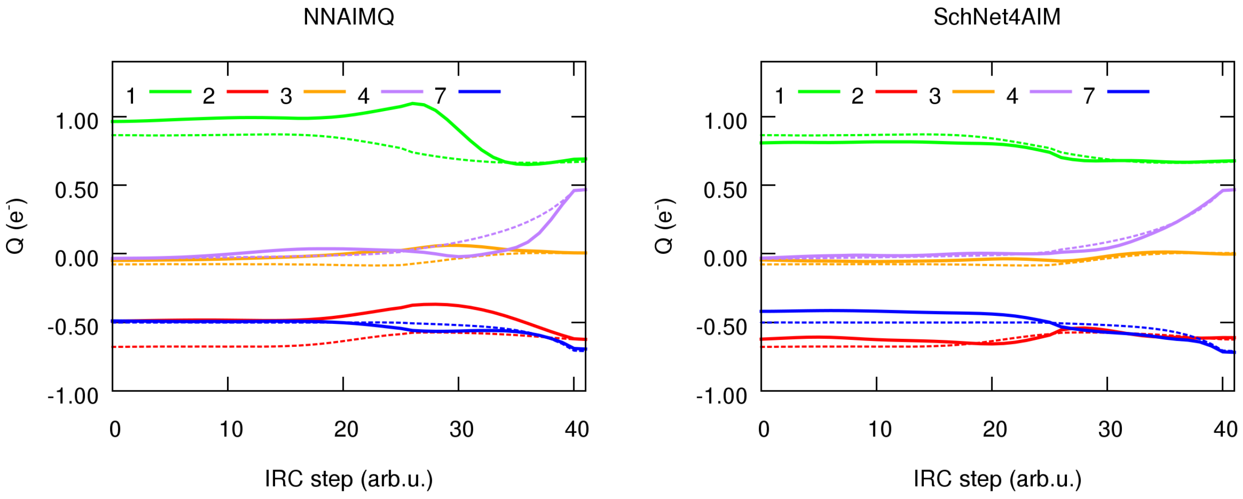

Supplementary Figure 16: **Evolution of the estimated atomic charges (Q) throughout the reaction.** Solid and dashed lines are used to represent the predicted and observed data, respectively. All values are reported in electrons (e^-). The labels correspond to the numbering shown in the main manuscript. The progress of the reaction is reported in terms of integer units (i.e steps) across the intrinsic reaction coordinate (IRC). Source data are provided as a Source Data file.

The following figure shows the evolution of the inter-molecular localized and delocalized electron counts between both reactants throughout the progress of the reaction.

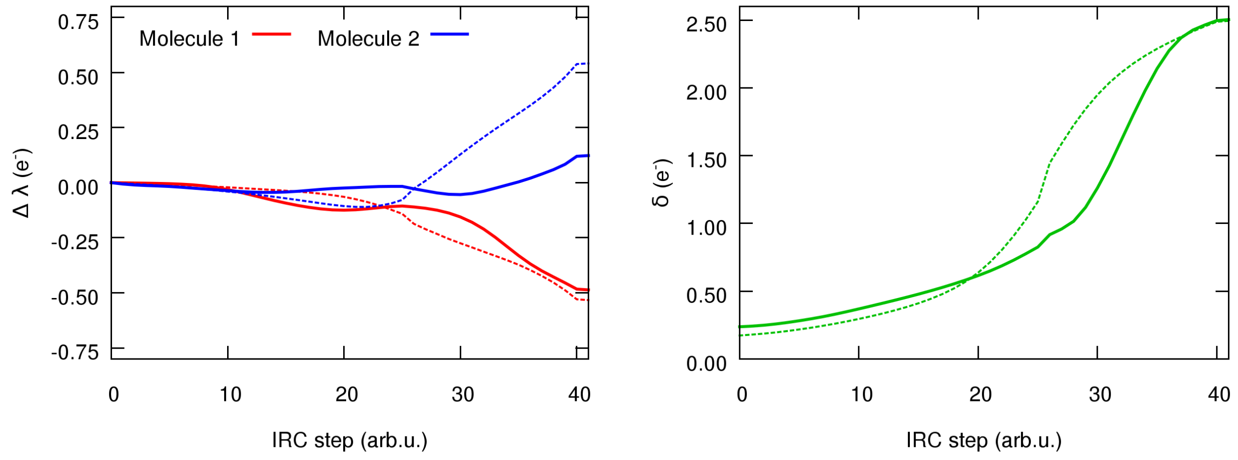

Supplementary Figure 17: **Evolution of the estimated group localized (λ) and delocalized (δ) electron populations throughout the reaction.** Solid and dashed lines are used to represent the predicted (SchNet4AIM) and observed data, respectively. All values are reported in electrons (e^-). The progress of the reaction is reported in terms of integer units (i.e steps) across the intrinsic reaction coordinate (IRC). For the sake of convenience, λ values are reported relative to the starting reactant complex, as $\Delta\lambda$. Source data are provided as a Source Data file.

The QM calculations show that the electron transfer accompanying the reaction crystallizes almost entirely in changes in the localized electron populations with $\Delta \sum \lambda \approx \pm\, 0.6$ electrons. Although SchNet4AIM reproduces this finding for the ethylene molecule, it underlocalizes the acetonitrile oxide by about 0.4 electrons. Such a more asymmetric shift in the group λ values suggests that our predictions slightly underestimate the decrease in the intra-molecular electron delocalization undergone throughout the reaction. On the contrary, much more subtle and smooth changes in the electron counts are found during the preparation stages. Indeed, the interaction between both reagents promotes the inter-fragment delocalization at the expense of reducing their respective λ values, as reflected by the subtle decrease in

the latter found just before the TS region. This promotion in electron delocalization is clearly seen in the evolution of the inter-group δ, gathered in the right panel of the previous figure, which undergo a substantial, yet steady, growth even in the first stages of the reaction.

Finally, the following figure shows the evolution of the predicted δ values of the bonds that get broken, or weakened, along the chemical transformation.

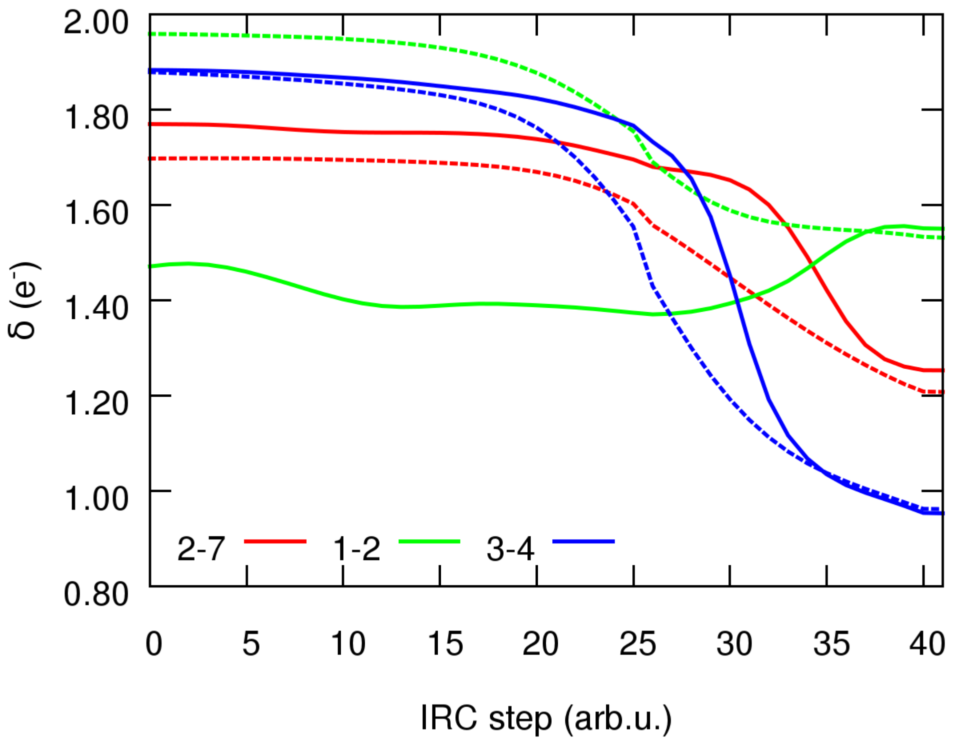

Supplementary Figure 18: **Evolution of the intra-molecular electron delocalization (δ) between the main atoms involved in the reaction**. Labels are used to indicate the atoms involved in each of the relevant atomic pairs, relying on the numbering shown in the main manuscript. Solid and dashed lines are used to refer to the SchNet4AIM predicted and quantum mechanically computed values, respectively. All values are reported in electrons (e^-). The progress of the reaction is reported in terms of integer units (i.e steps) across the intrinsic reaction coordinate (IRC). Source data are provided as a Source Data file.

Finally, it is worth stressing that, generally speaking, ML models are designed to interpolate. Otherwise, the latter can provide erratic behaviors when forced to make predictions on extrapolation regimes. Taking this into account, the extrapolation abilities of our models are a particularly interesting, and fairly unique, feature. Naturally, such a capability emerges from the use of real-space local quantum chemical properties, which display a remarkable transferability. That said, we expect the extrapolation abilities of SchNet4AIM to be limited by three main factors:

- The size and quality of the training dataset: presenting the model with a large varied collection of training instances can aid it to distill the hierarchical representations that enable the mapping from the input features to the output properties. In this way, an adequate sampling of the chemical space can result in more robust and reliable SchNet4AIM models with better generalization abilities even in extrapolation regimes.

- The representation kernel employed: relying on a local representation, SchNet4AIM has different parameters that allow to control the extent to which the local environment of an atom extends in the space. Selecting a small to medium size cutoff radius will generally enhance the transferability of SchNet4AIM predictions, often, at the expense of reducing the accuracy with which the local environment vectors describe the atomic and interatomic features.

- Finally, the extrapolation abilities are also highly dependent on the complexity of the target properties, and the influence that the local environment has on the latter.

As such, we expect larger and more complex systems to be more challenging. Besides being supported by the aforementioned results, this is in fact a general behavior that accepts a very simple and intuitive interpretation: when given larger and more complex systems, SchNet4AIM is more likely to encounter never-seen local chemical environments which are very distant to those found throughout the learning stage, and thus worsening the quality of the predictions. We note in passing that in these particularly challenging situations, model regularization (e.g L2 regularization or dropout techniques) can come particularly handy as they can prevent SchNet4AIM from excessively fitting the reference data used throughout its learning stages.

Currently we employ a combination of different approaches to effectively enhance the generalization abilities of our models in such complex scenarios:

- Tuning the SchNet4AIM representation parameters: as mentioned above, the SchNet4AIM representation parameters used to describe the local environments can be tuned to improve the model's generalization. More specifically, imposing more locality, reducing the number of interaction layers as well as the number of chemical features yields more local, compact and short-sighted descriptors which allow the model to better generalize in complex never-seen scenarios. Naturally, this comes at the expense of decreasing the accuracy with which the physical variables dominating the systems are captured and thus could result in a subtle lowering of the mean model performances.

- Controlling the importance of the molecular and local properties: as already discussed in previous sections, a global-to-local tradeoff function is used to create the final loss function term from the errors made in the prediction of the local values as well as in the reconstruction of the molecular observables. Increasing the weight of the molecular information prevents the model from excessively fitting the local values, while ensuring a better reconstruction of the global quantities. As such, this tradeoff is tuned depending on the specific task at hand and complexity of the dataset, effectively acting as a regularizer.

- Loss function term: finally, the actual kernel used to compute the loss functions is also modified to control the strength with which SchNet4AIM is penalized when deviating from the reference data used for its training. As such different cost function kernels, or combinations of them, are used, such as the L1 and L2 norms, to impose an elastic regularization of the models.

Furthermore different regularization approaches, beyond the explicit hyper-parameter tuning or early-stopping approaches, could be implemented in the future, such as:

- Layer regularizers: regularizers could be included in particular layers of the SchNet4AIM architecture to penalize the excessive fitting of certain model parameters to the reference data. This could include kernel, bias and activity regularizers, among others.

- Dropout technique: additional layers, known as dropout layers, which deliberately discard the output of certain neurons to prevent overfitting. The dropout rate can be used to control the probability of a neuron of getting deactivated throughout the training, controlling the strength with which the model gets regularized.

- Batch normalization: additional standardization operations can be applied to the input of a layer so that the latter receives a properly normalized input. Doing so can greatly enhance the ability of that layer to extract relevant hierarchical representations which ultimately results in better learning protocols. Besides this, the normalization operation introduces noise throughout its training which effectively acts as a regularizer as it hampers the model from focusing too much on specific node activations. Doing so, forces the model to learn more general features, reducing the risk of overfitting.

to name a few.

Supplementary Note 14. δ as a valuable metric of chemical interactions in MD simulations

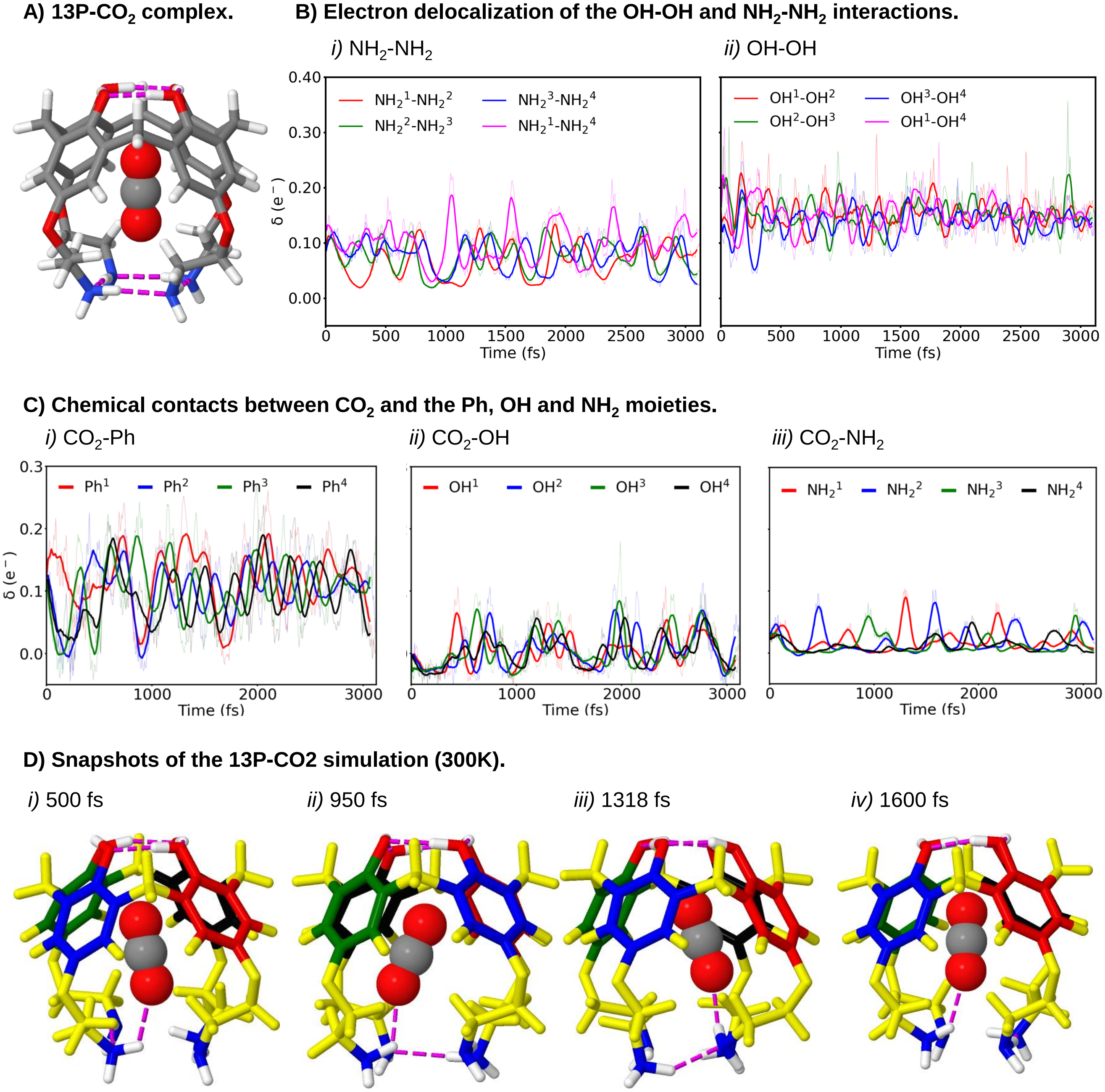

Supplementary Figure 19: **13P-CO_2 simulation at 300 K.** **(a)** Optimized geometry of the 13P-CO_2 (closed) complex. **(b)** Evolution of the SchNet4AIM electronic descriptors (namely the electron delocalization, δ) of the intramolecular NH_2 (*i*) and OH (*ii*) HB network throughout the 300 K MD simulation of 13P-CO_2. **(c)** Evolution of the SchNet4AIM electronic descriptors (δ) between CO_2 and the phenyl (*i*), hydroxo (*ii*) and amino (*iii*) moieties of 13P throughout the 300 K MD simulation of 13P-CO_2. **(d)** Snapshots of the 13P-CO_2 system at 500 (*i*), 950 (*ii*), 1318 (*iii*) and 1600 (*iv*) fs throughout the 300 K simulation, corresponding to some of the local maxima in the $\delta(CO_2,NH_2)$. The color of the aromatic scaffolds has been selected to match that used in the numbering of the graphs. All molecular representations were rendered with Jmol (21). For the electronic metrics, the raw and bin-averaged data are shown, in electrons (e^-). Source data are provided as a Source Data file.

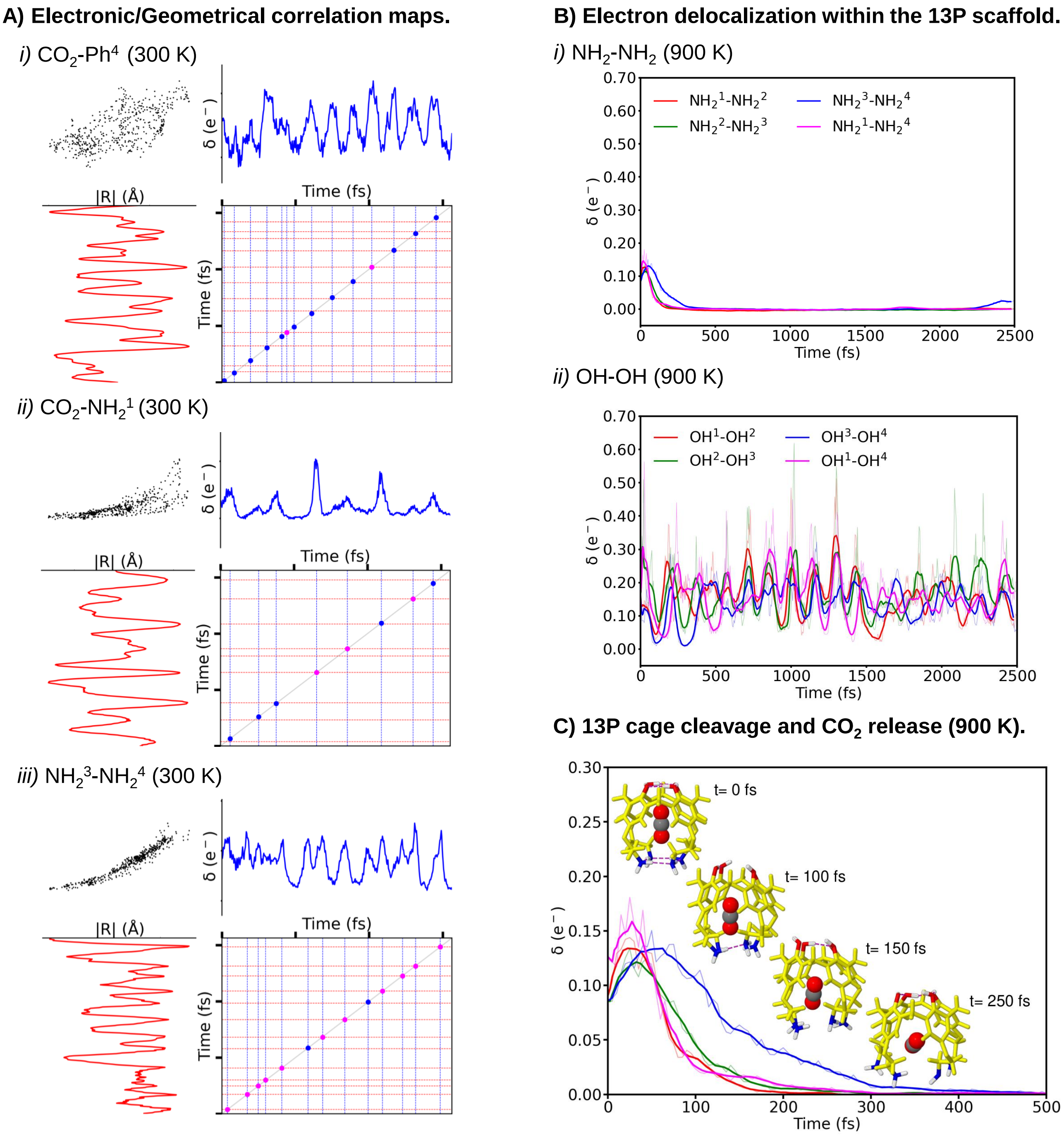

Supplementary Figure 20: **Chemical insights on the 13P-CO_2 complexation and release from SchNet4AIM predictions.** **(a)** Correlation maps between the electronic and geometrical descriptors for the CO_2-Ph^4 (i), CO_2-NH_2^1 (ii) and NH_2^3-NH_2^4 (iii) contacts throughout the 300 K 13P-CO_2 simulation. Blue dots indicate those binding events exclusively predicted by SchNet4AIM, whereas magenta ones show contacts simultaneously ($\pm$ 10 fs) estimated by the geometrical and electronic metrics. The electron delocalization, δ, and distance, $|R|$, axes have been omitted for the sake of simplicity. Each tick corresponds to 1000 fs. **(b)** Evolution of the SchNet4AIM predicted δ values of the intramolecular NH_2 (i) and OH (ii) HB networks throughout the 900 K 13P-CO_2 simulation. **(c)** Snapshots of the system at 0, 100, 150 and 250 fs throughout the 900 K 13P-CO_2 simulation, along with the SchNet4AIM computed δ(NH_2-NH_2), showing the cage opening phenomenon. The same color code as in Supplementary Figure 19B(i) is employed. For the electronic metrics, the raw and bin-averaged data are shown. The electron delocalization is given in electrons (e^-). Source data are provided as a Source Data file.

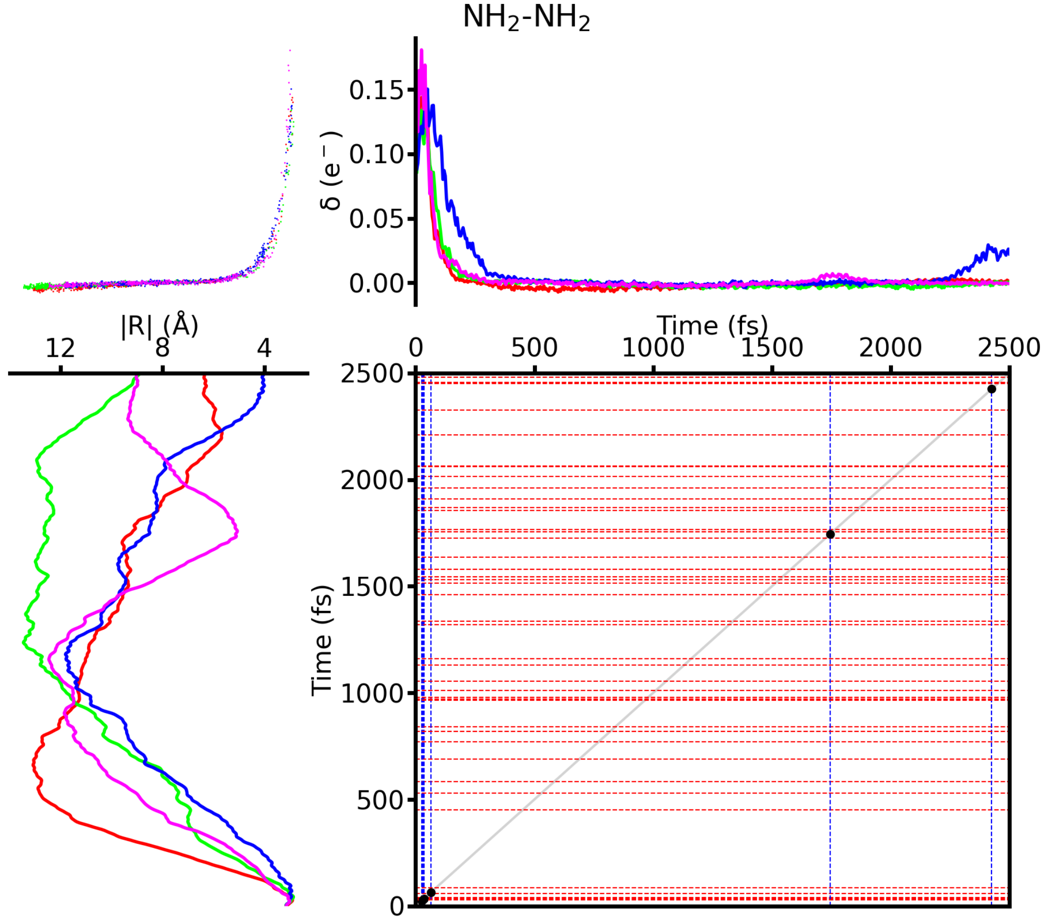

Supplementary Figure 21: **Correlation maps between the electronic and geometrical descriptors.** Correlation map for the interaction between neighboring NH_2-NH_2 groups in the 13P scaffold throughout the 900 K 13P-CO_2 MD simulation. Black dots show the binding events of each pair of amino groups. The same color code as that used in 19B(i) is employed. The evolution of the electron delocalization, δ in electrons, along with the distance between the centers of mass, |R|, is shown. Source data are provided as a Source Data file.

Supplementary Figure 19C gathers the evolution of the δ values between CO_2 and the different moieties of the 13P: the hydroxo (OH), amino (NH_2) and phenyl (Ph) groups. The fairly steady δ(CO_2,Ph) values, of about 0.10 electrons, suggest quite frequent contacts owing to their close proximity (3.6 Å on average) which allows for a non-negligible π-π interaction between the electron clouds of both fragments. On the contrary, δ(CO_2,NH_2) and δ(CO_2,OH) behave in a drastically different way, exhibiting abrupt and localized spikes. This result, particularly prominent for the NH_2-CO_2 pair, reveals the formation of directional chemical contacts such as HB or dipole-dipole interactions between the polar moieties of both groups. The formation of these interactions weakens the collective HB network at the bottom of the cage, while strengthening certain NH_2-NH_2 contacts as found, for instance, for groups 1-4, shown in the left panel of Supplementary Figure 19B. On the other hand, the OH-OH interactions show quite monotonous oscillations about their mean values, suggesting that the $(OH)_4$ network at the top of the Calix[4]arene is rarely disrupted by the presence of CO_2. Additionally, null electron delocalization values were found between CO_2 and the linker O atoms as the latter are facing the outer-part of the main skeleton, being thus inaccessible to the ligand.

The fluctuating nature of the distance throughout the simulations masks the subtle changes in the latter attributed to the emerging interactions. This, coupled to the inability of the distance to account for the relative orientation of the molecules, difficults the characterization of discrete binding events relying, solely, on geometrical features. Actually, a comparison of the performance of the electronic and geometrical descriptors can be achieved from correlation plots, shown in Supplementary Figure 20A. We note in passing that the greatest discrepancies are found for the CO_2-O contacts, for which the changes in the distance would lead us to mistakenly conclude the presence of multiple fictitious (non-existing) contacts. Despite the larger oscillations induced by the prominent perturbations of the bonds at higher temperatures (900 K), the δ(OH,OH) values, shown in the bottom panel of Supplementary Figure 20B, exhibit relatively steady trends indicating that, although perturbed, the net $(OH)_4$ network is not broken. Diametrically opposed findings

are observed for the bottom backbone of the cage (top panel of Supplementary Figure 20B) for which the vanishing δ values suggest the full cleavage of the NH_2-NH_2 HB network. These findings in the electronic features are in perfect agreement with the sequence of events found throughout the trajectories, comprised in Supplementary Figure 20C.

Finally, looking at the NH_2-NH_2 correlation plot, shown in Supplementary Figure 21, reveals a strong lack of correlation between the topologies of the geometrical and electronic features: with the exception of the evident HB contacts (represented as dots), the evolution of the distance exhibits numerous critical points which do not correspond to any proper binding event. Such an observation, in agreement with the previously mentioned trends proves, again, the validity of our approach as a more robust indicator of the chemical interactions emerging throughout a dynamic process.

CO_2 capture by Calix[4]arene 13P

The current section gathers the results found throughout the MD simulation (300 K) of the 13P-CO_2 complex.

The following figures show the evolution of the total energy and temperature of the system (13P-CO_2) throughout the progress of the MD simulation.

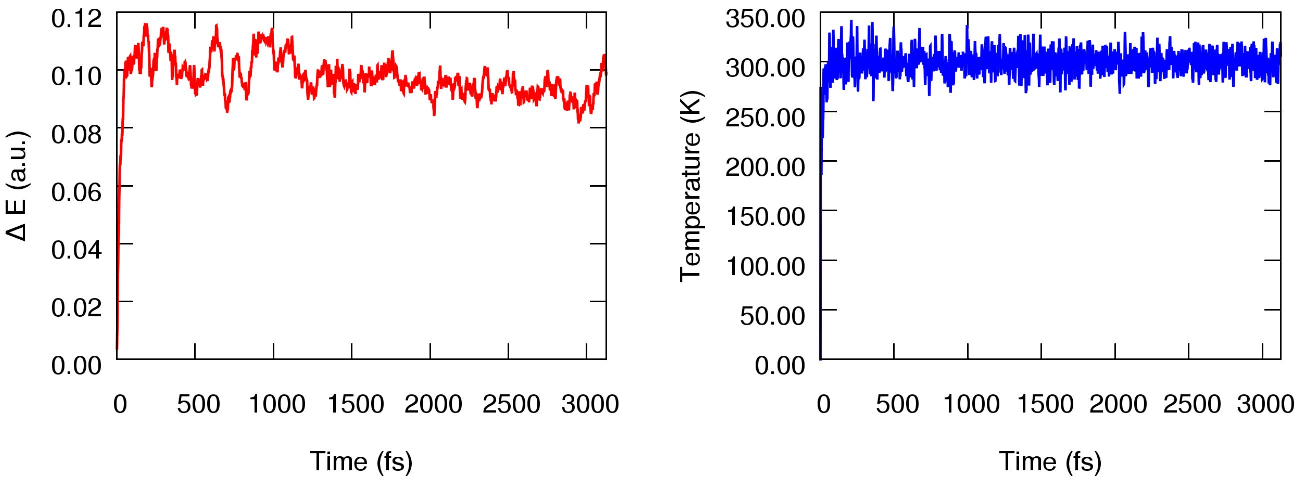

Supplementary Figure 22: **Energy (E) and temperature (T) fluctuations at 300 K.** Evolution of the change in the total energy of the system, ΔE, (left) and temperature (right) throughout the MD simulation. Source data are provided as a Source Data file.

The following figures show the evolution of the geometrical features, reported as the distance between the centers of mass, between the different scaffolds of the system throughout the MD simulation.

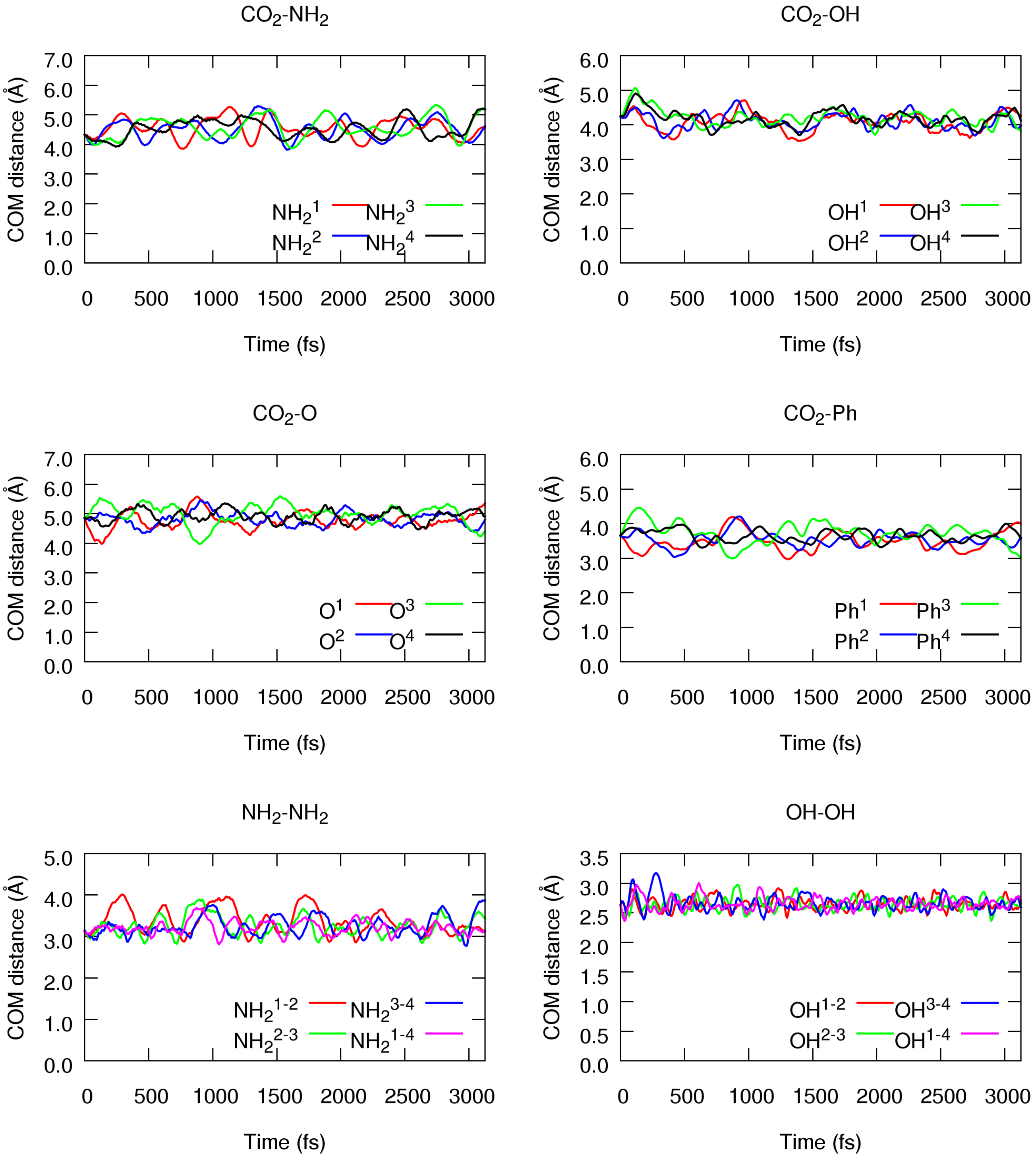

Supplementary Figure 23: **Center of mass (COM) distances in the 13P-CO_2 complex at 300 K.** Evolution of the distance between the main scaffolds of the 13P-CO_2 system throughout the simulation. Source data are provided as a Source Data file.

The following figures show the correlation maps (distance vs electron delocalization) for the interaction between the different scaffolds throughout the simulation. To ease the peak detection, the data was smooth through convolution using a kernel size of 20. The vertical and horizontal lines show the time at which different binding events are predicted according to the geometrical and electronic descriptors. Magenta dots show binding events which are simultaneously predicted by both descriptors, allowing for a maximum offset of 10 fs, whereas blue dots show binding events estimated, exclusively, by the electronic metrics.

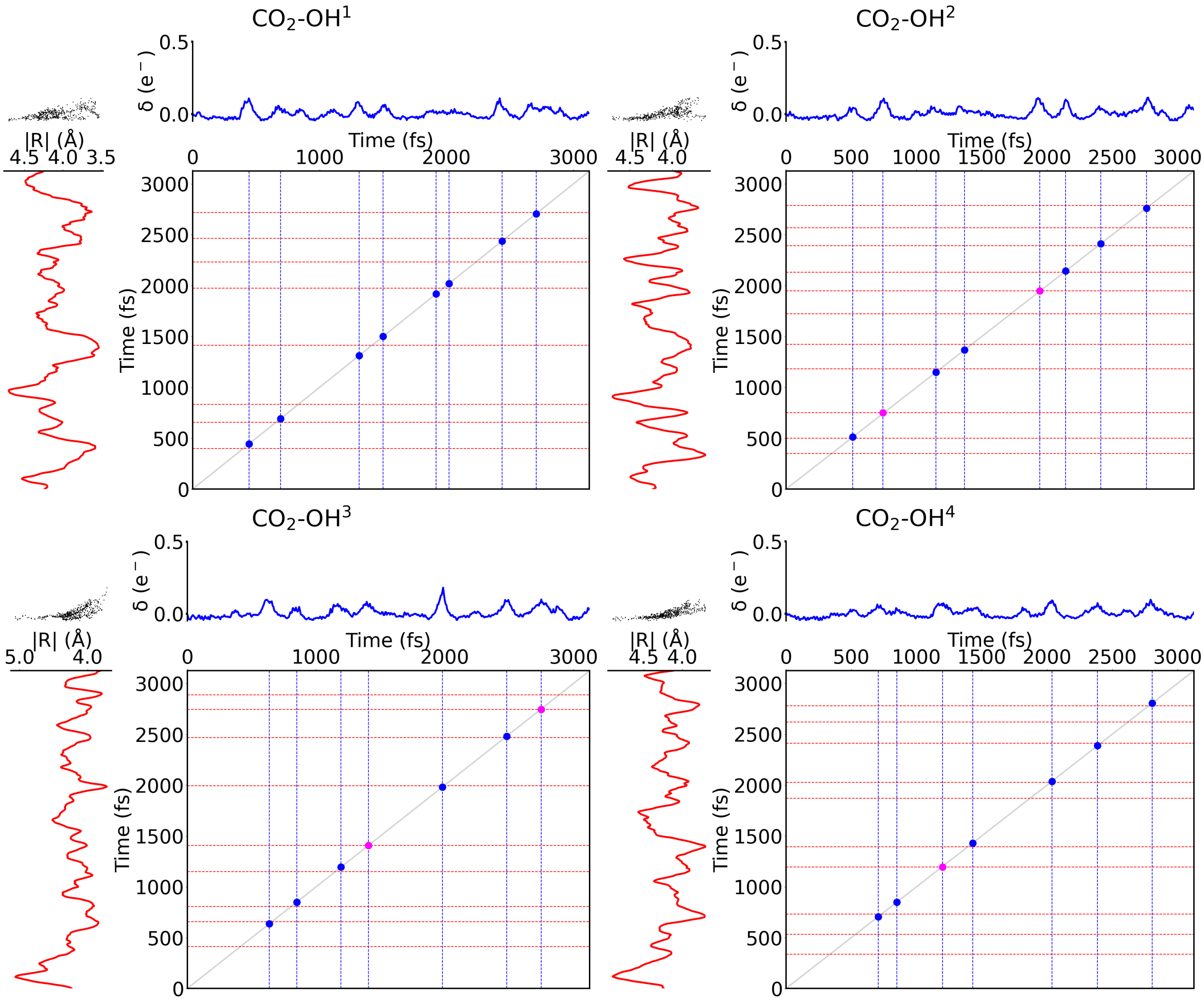

Supplementary Figure 24: **CO_2-OH correlation maps at 300 K.** Correlation maps between CO_2 and the OH moieties of the 13P Calix[4]arene. The evolution of the group electron delocalization, δ (in electrons, e^-), and the distance between the centers of mass, |R|, across the simulations is shown. Source data are provided as a Source Data file.

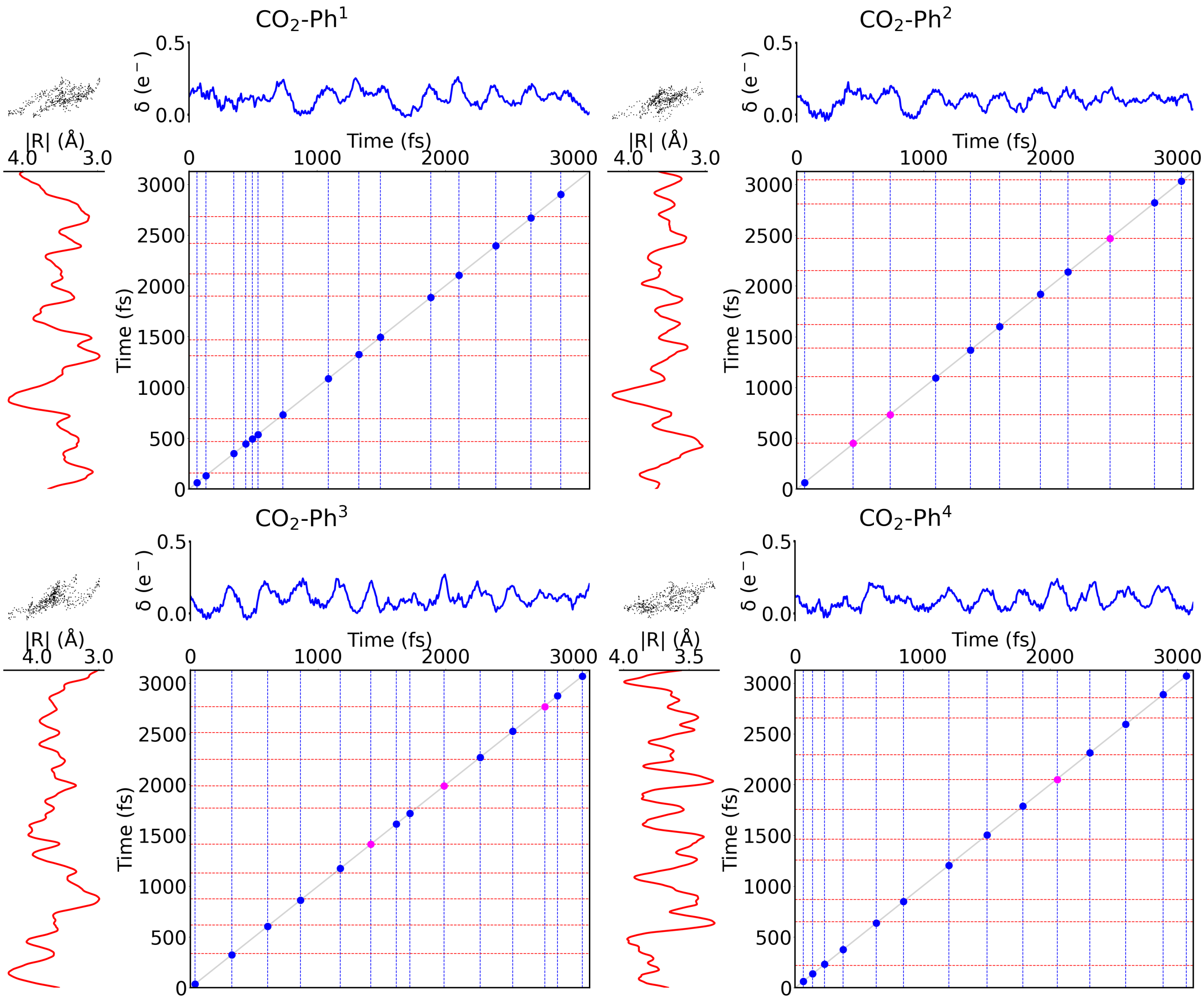

Supplementary Figure 25: **CO_2-Ph correlation maps at 300 K.** Correlation maps between CO_2 and the Ph moieties of the 13P Calix[4]arene. The evolution of the group electron delocalization, δ (in electrons, e^-), and the distance between the centers of mass, |R|, across the simulations is shown. Source data are provided as a Source Data file.

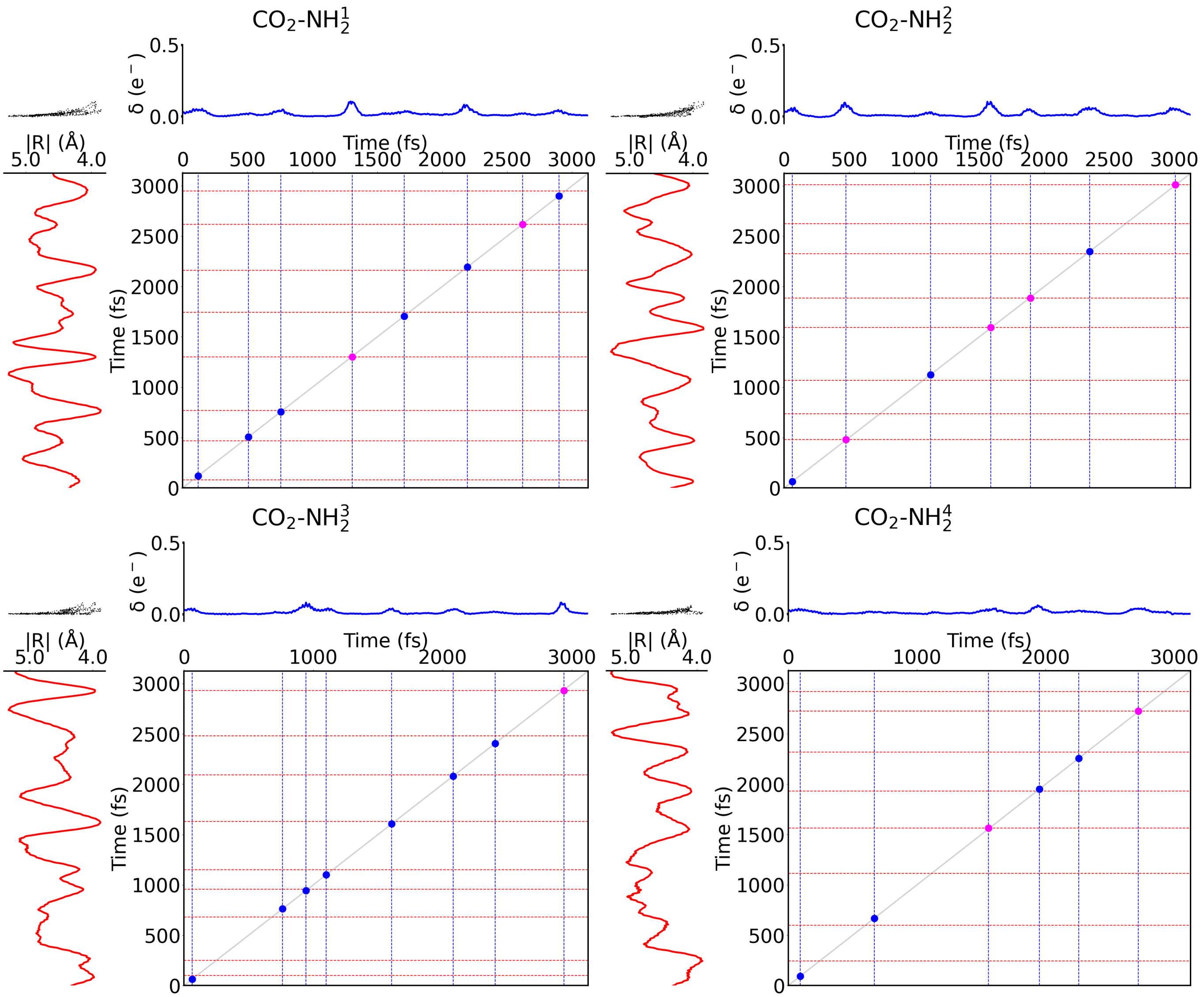

Supplementary Figure 26: **CO_2-NH_2 correlation maps at 300 K.** Correlation maps between CO_2 and the NH_2 moieties of the 13P Calix[4]arene. The evolution of the group electron delocalization, δ (in electrons, e^-), and the distance between the centers of mass, |R|, across the simulations is shown. Source data are provided as a Source Data file.

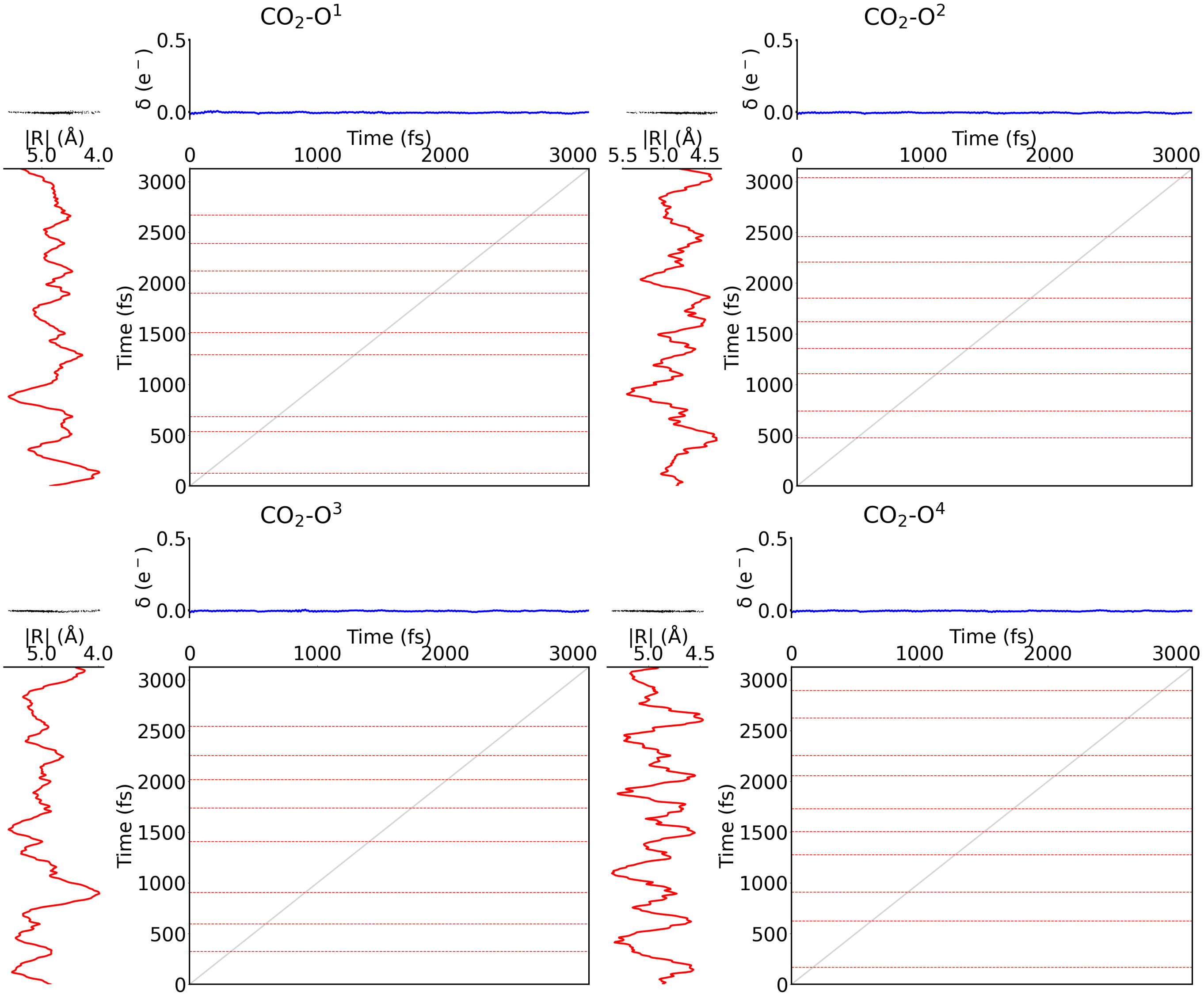

Supplementary Figure 27: **CO_2-O correlation maps at 300 K.** Correlation maps between CO_2 and the O moieties of the 13P Calix[4]arene. The evolution of the group electron delocalization, δ (in electrons, e^-), and the distance between the centers of mass, |R|, across the simulations is shown. Source data are provided as a Source Data file.

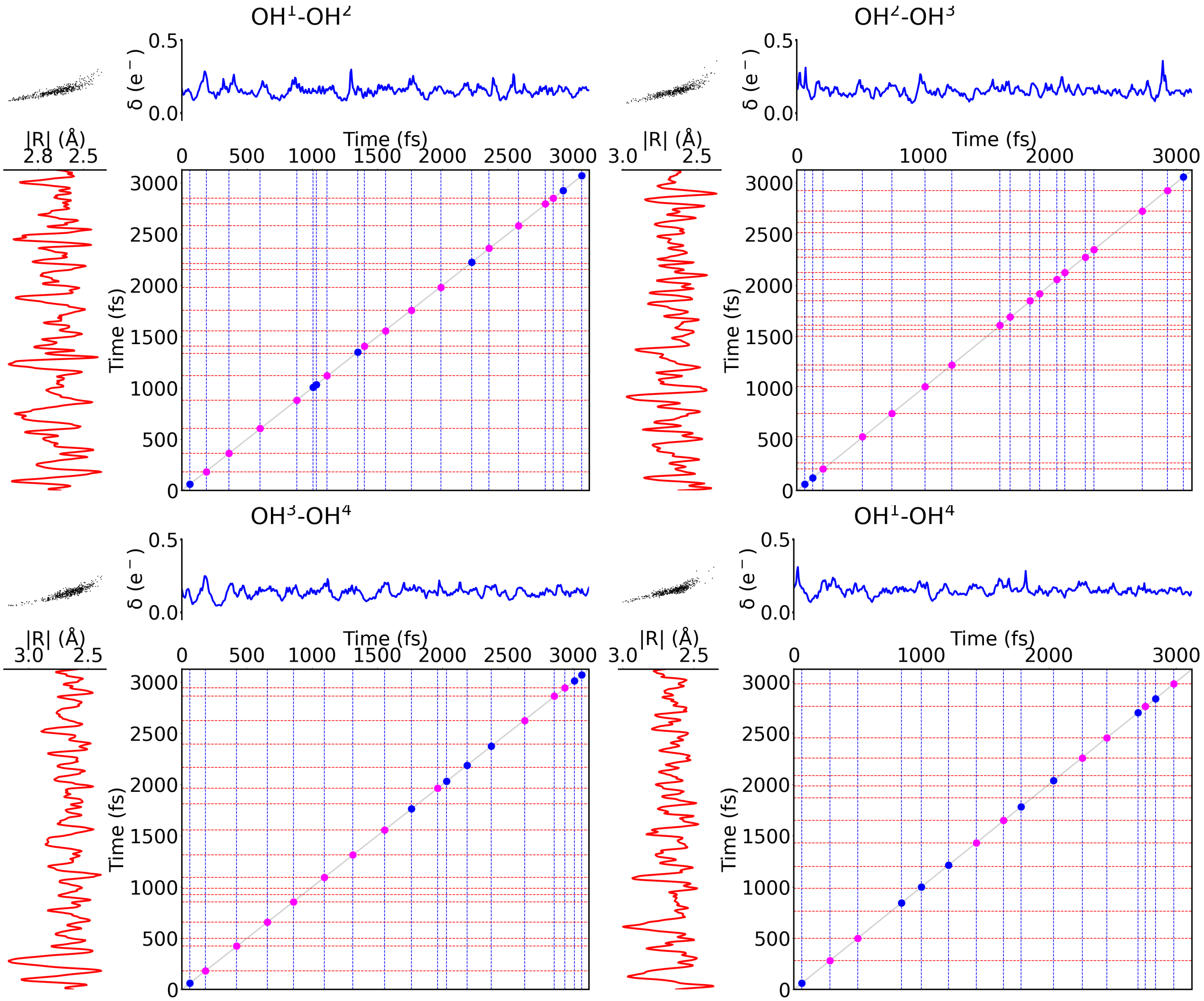

Supplementary Figure 28: **OH-OH correlation maps at 300 K.** Correlation maps between OH moieties of the 13P Calix[4]arene. The evolution of the group electron delocalization, δ (in electrons, e^-), and the distance between the centers of mass, |R|, across the simulations is shown. Source data are provided as a Source Data file.

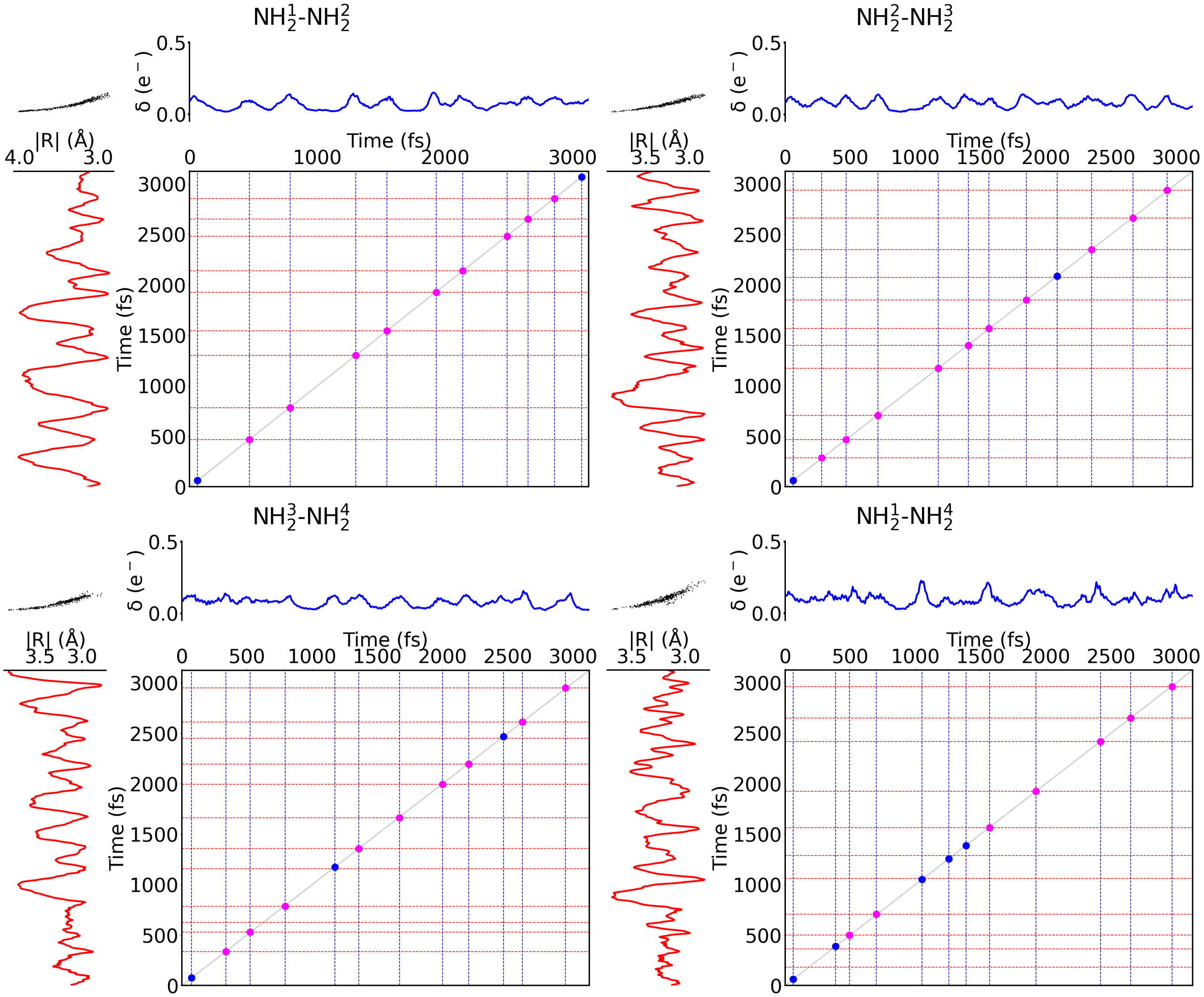

Supplementary Figure 29: **NH_2-NH_2 correlation maps at 300 K.** Correlation maps between NH_2 moieties of the 13P Calix[4]arene. The evolution of the group electron delocalization, δ (in electrons, e^-), and the distance between the centers of mass, |R|, across the simulations is shown. Source data are provided as a Source Data file.

The following figures show the evolution of the group delocalization index (δ), reported in electrons, between the different scaffolds of the system throughout the MD simulation.

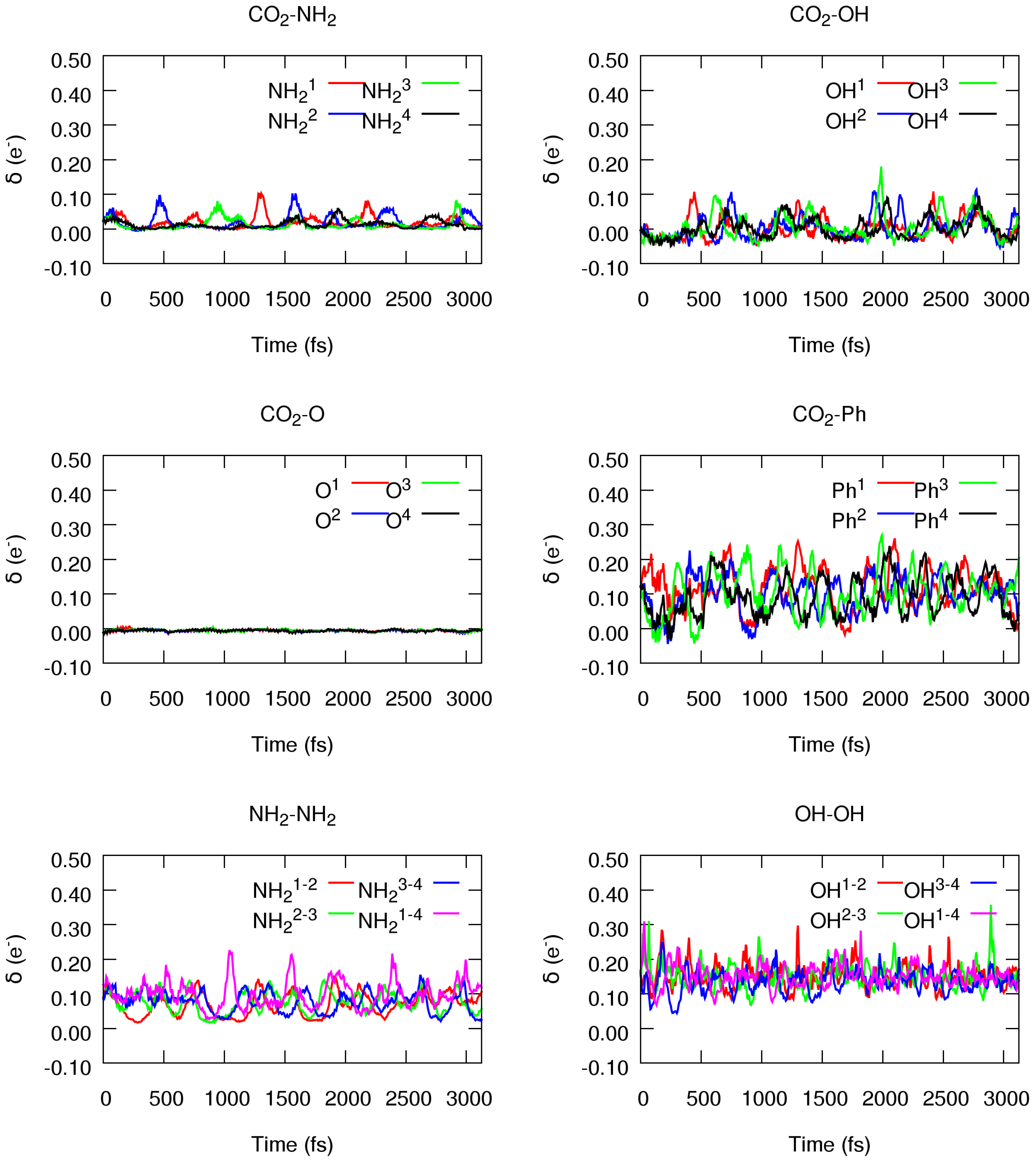

Supplementary Figure 30: **Electron delocalization in 13P-CO_2 complex at 300 K.** Evolution of the electron delocalization, δ, estimated by SchNet4AIM, between the main scaffolds of the 13P-CO_2 system throughout the simulation. All values are given in electrons (e^-). Source data are provided as a Source Data file.

CO_2 release by Calix[4]arene 13P

The current section gathers the results found throughout the MD simulation (900 K) of the 13P-CO_2 complex. The following figures show the evolution of the geometrical features, reported as the distance between the centers of mass, between the different scaffolds of the system throughout the MD simulation.

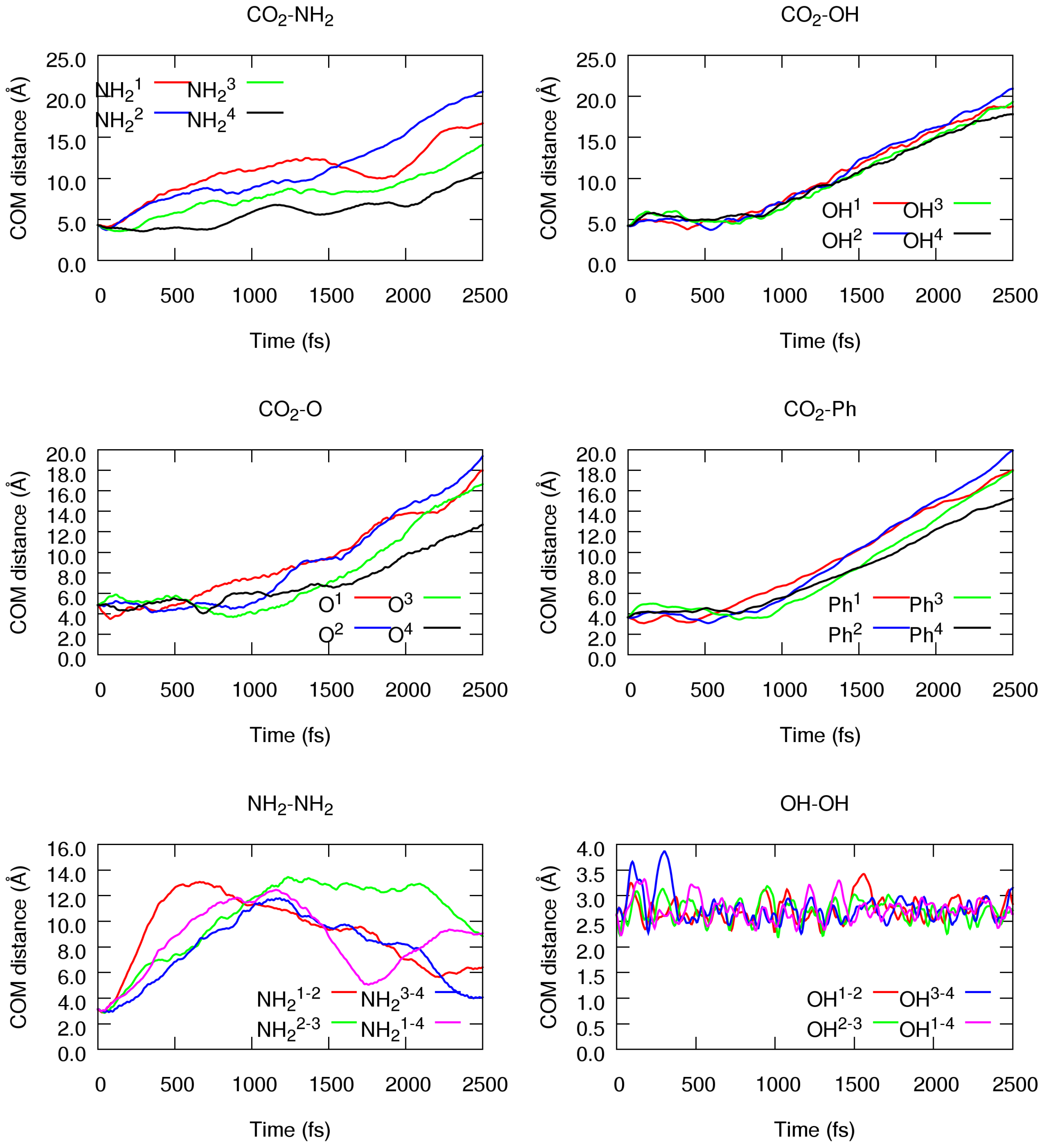

Supplementary Figure 31: **Center of mass (COM) distances in the 13P-CO_2 complex at 900 K.** Evolution of the distance between the main scaffolds of the 13P-CO_2 system throughout the simulation. Source data are provided as a Source Data file.

The following figures show the correlation maps (distance vs electron delocalization) for the interaction between the different scaffolds throughout the simulation. To ease the peak detection, the data was smooth through convolution using a kernel size of 20. The vertical and horizontal lines show the time at which different binding events are predicted according to the geometrical and electronic descriptors. Magenta dots show binding events which are simultaneously predicted by both descriptors, allowing for a maximum offset of 10 fs, whereas blue dots show binding events estimated, exclusively, by the electronic metrics.

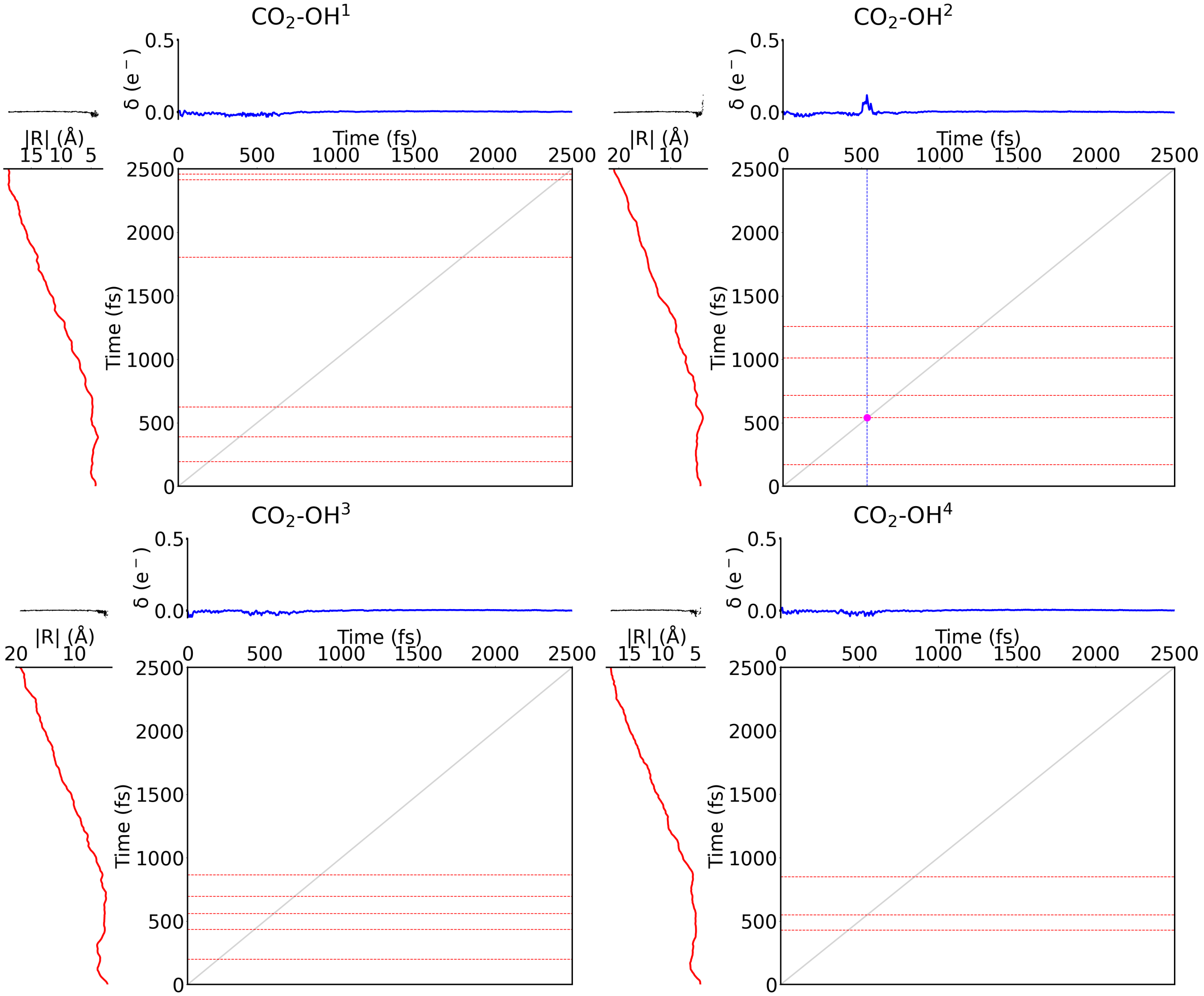

Supplementary Figure 32: **CO_2-OH correlation maps at 900 K.** Correlation maps between CO_2 and the OH moieties of the 13P Calix[4]arene. The evolution of the group electron delocalization, δ (in electrons, e^-), and the distance between the centers of mass, $|R|$, across the simulations is shown. Source data are provided as a Source Data file.

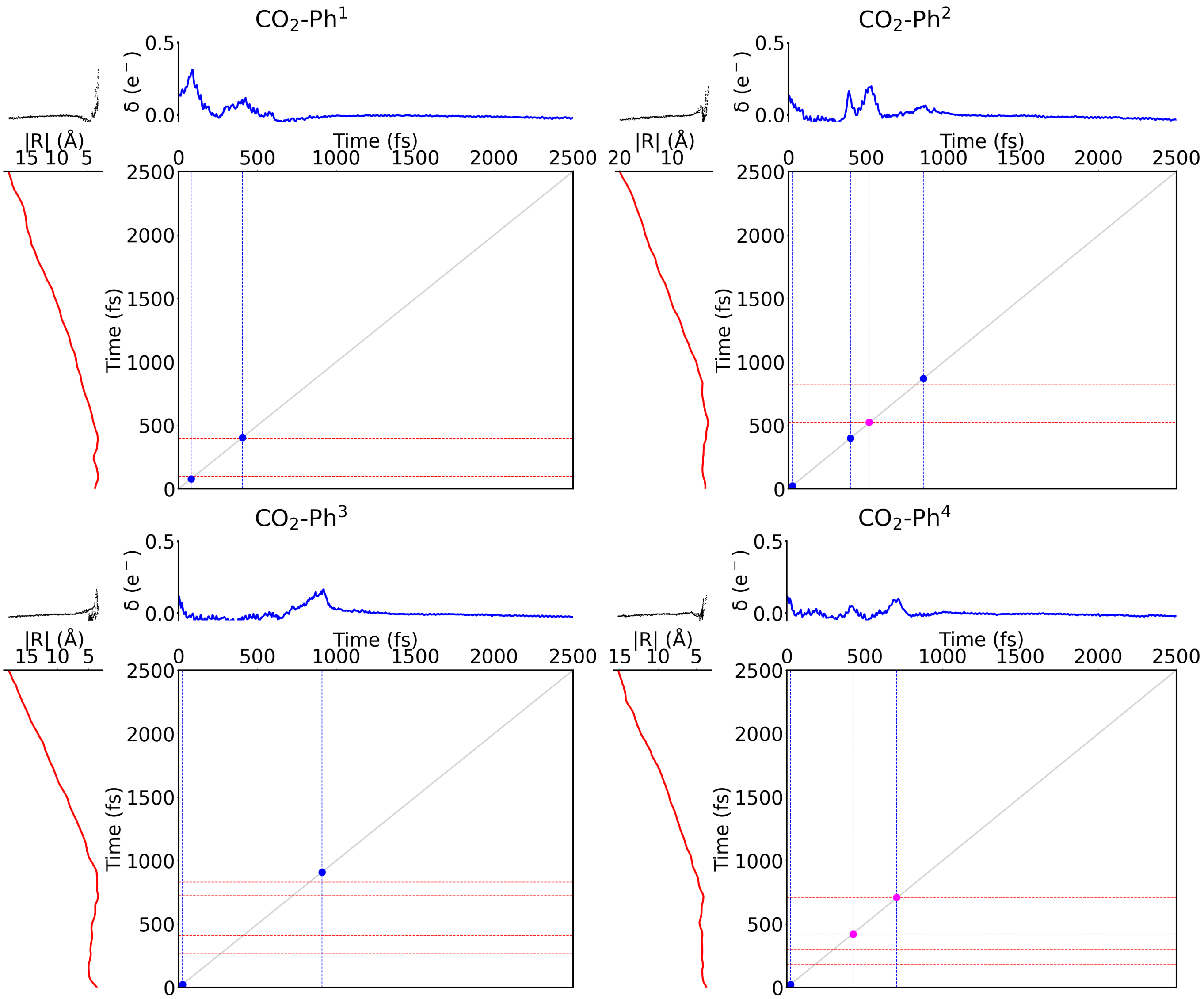

Supplementary Figure 33: **CO_2-Ph correlation maps at 900 K.** Correlation maps between CO_2 and the Ph moieties of the 13P Calix[4]arene. The evolution of the group electron delocalization, δ (in electrons, e^-), and the distance between the centers of mass, |R|, across the simulations is shown. Source data are provided as a Source Data file.

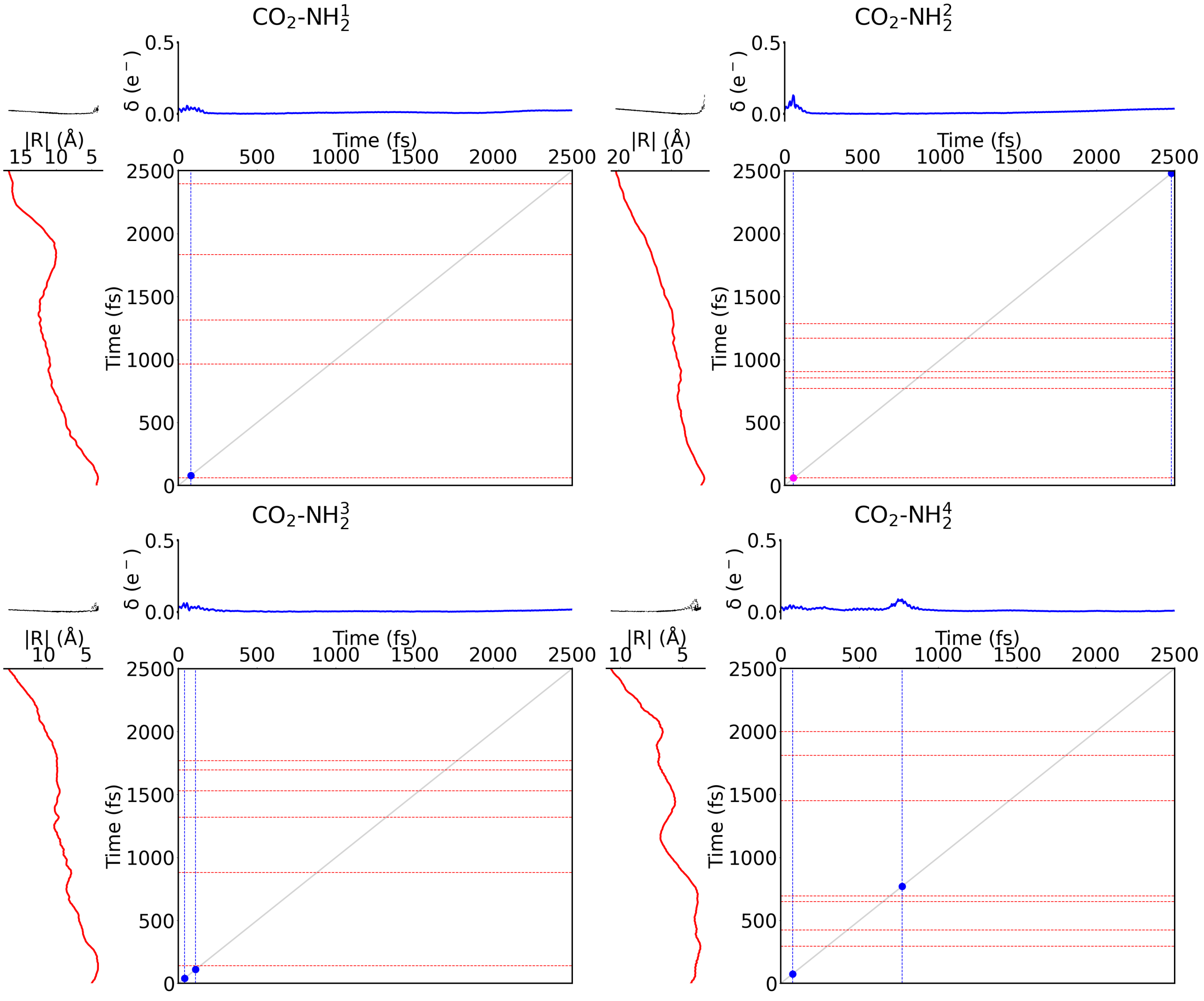

Supplementary Figure 34: **CO_2-NH_2 correlation maps at 900 K.** Correlation maps between CO_2 and the NH_2 moieties of the 13P Calix[4]arene. The evolution of the group electron delocalization, δ (in electrons, e^-), and the distance between the centers of mass, |R|, across the simulations is shown. Source data are provided as a Source Data file.

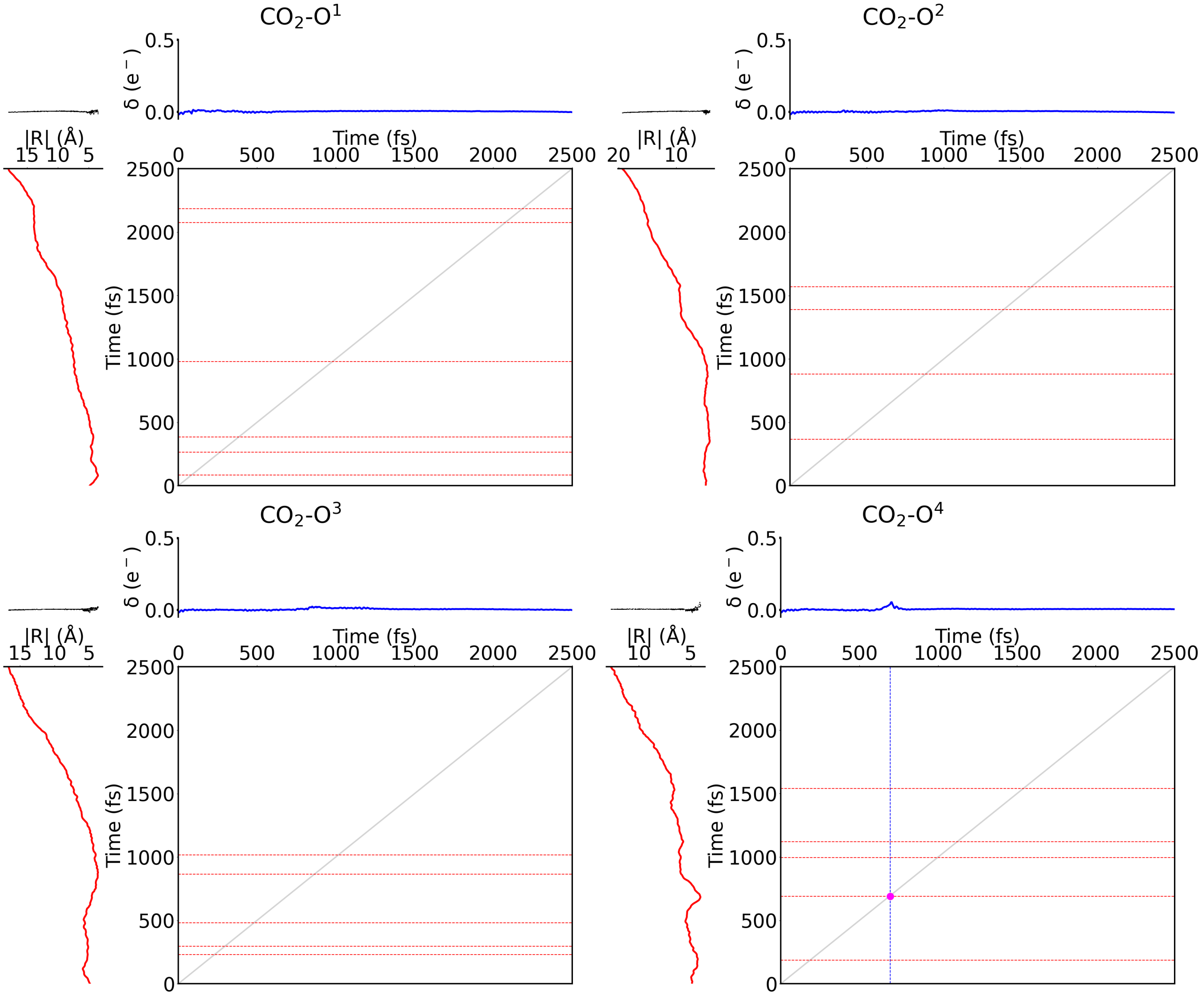

Supplementary Figure 35: **CO_2-O correlation maps at 900 K.** Correlation maps between CO_2 and the O moieties of the 13P Calix[4]arene. The evolution of the group electron delocalization, δ (in electrons, e^-), and the distance between the centers of mass, |R|, across the simulations is shown. Source data are provided as a Source Data file.

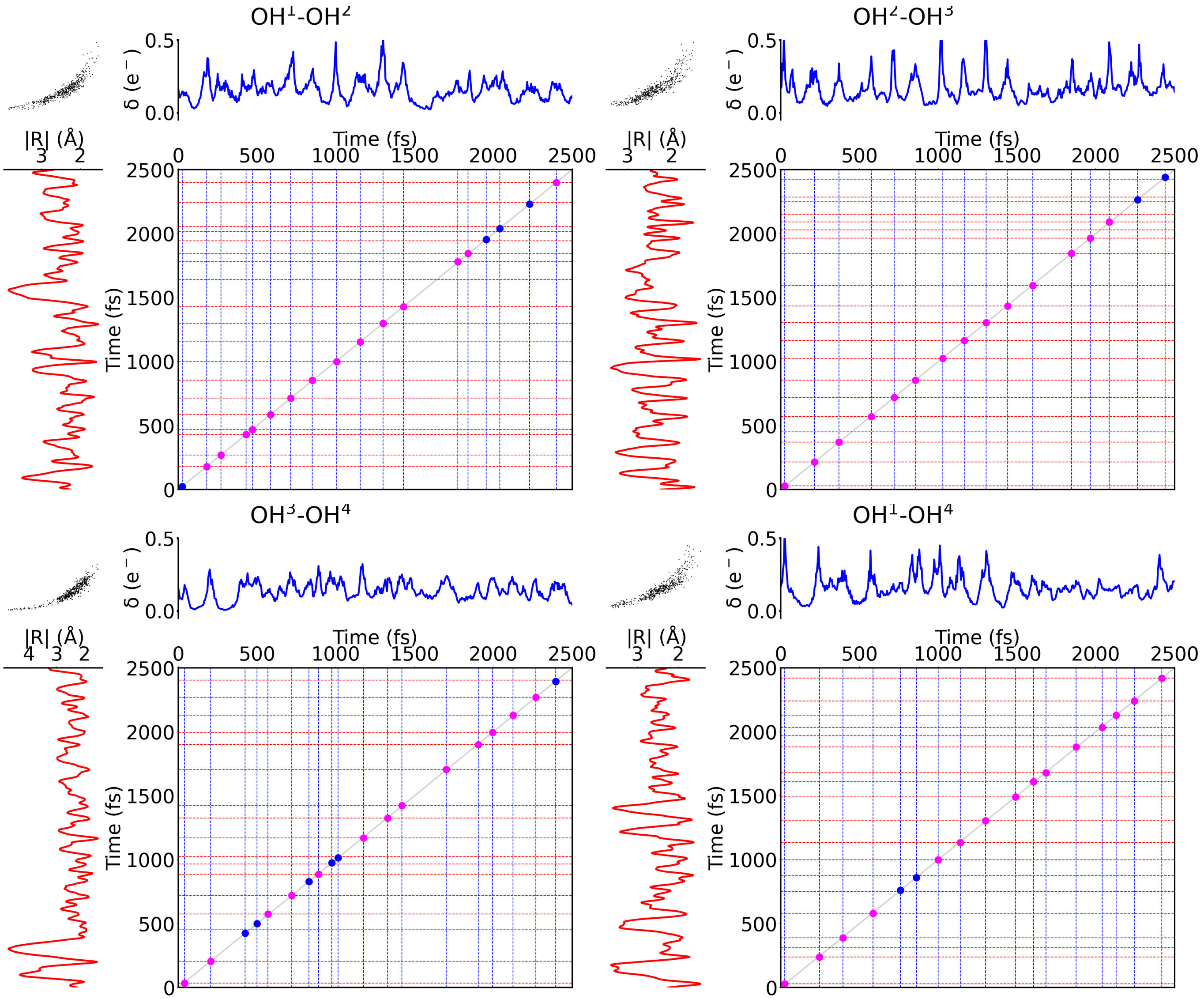

Supplementary Figure 36: **OH-OH correlation maps at 900 K.** Correlation maps between OH moieties of the 13P Calix[4]arene. The evolution of the group electron delocalization, δ (in electrons, e^{-}), and the distance between the centers of mass, $|R|$, across the simulations is shown. Source data are provided as a Source Data file.

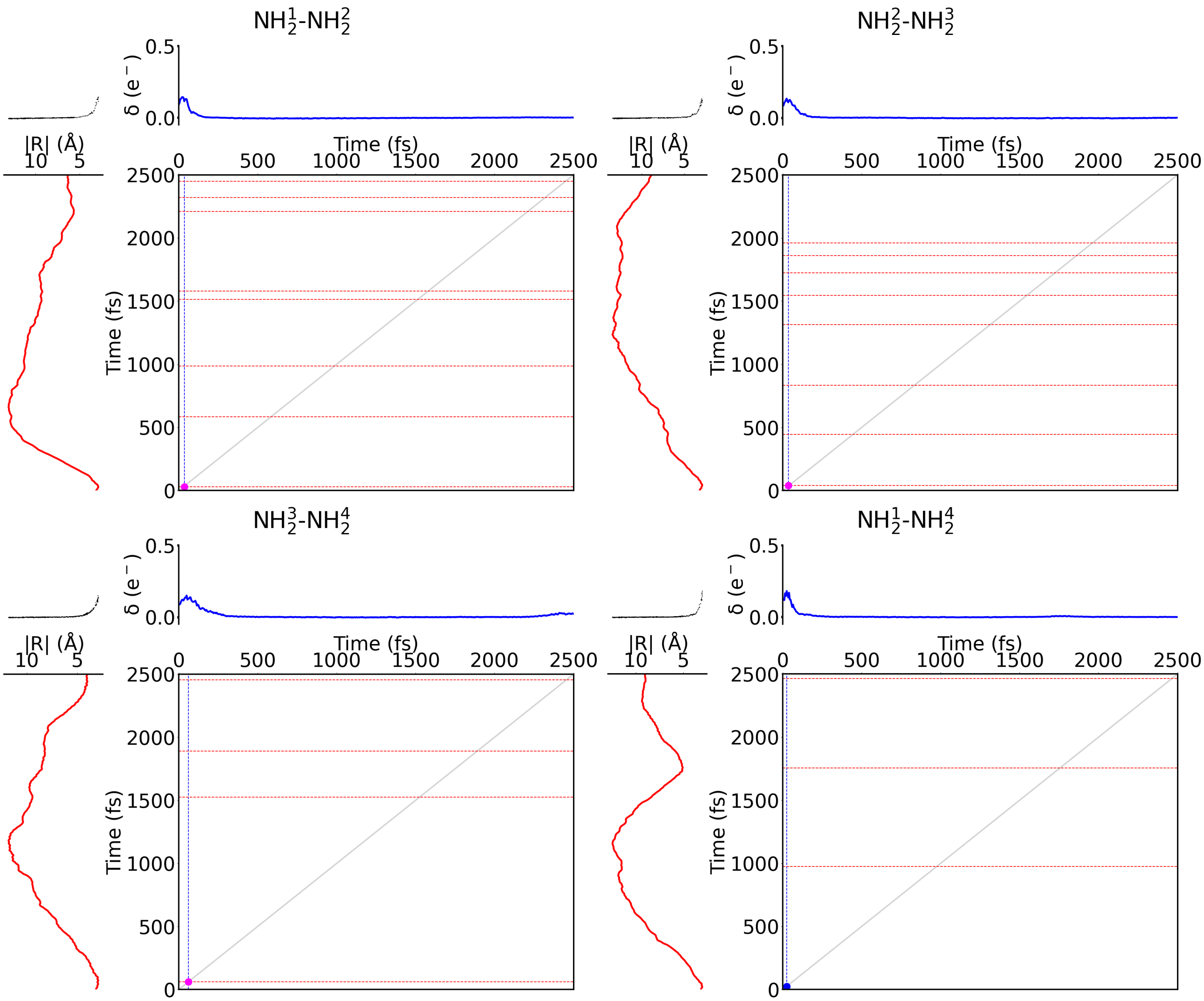

Supplementary Figure 37: **NH_2-NH_2 correlation maps at 900 K.** Correlation maps between NH_2 moieties of the 13P Calix[4]arene. The evolution of the group electron delocalization, δ (in electrons, e^-), and the distance between the centers of mass, |R|, across the simulations is shown. Source data are provided as a Source Data file.

The following figures show the evolution of the group delocalization index (δ), reported in electrons, between the different scaffolds of the system throughout the MD simulation.

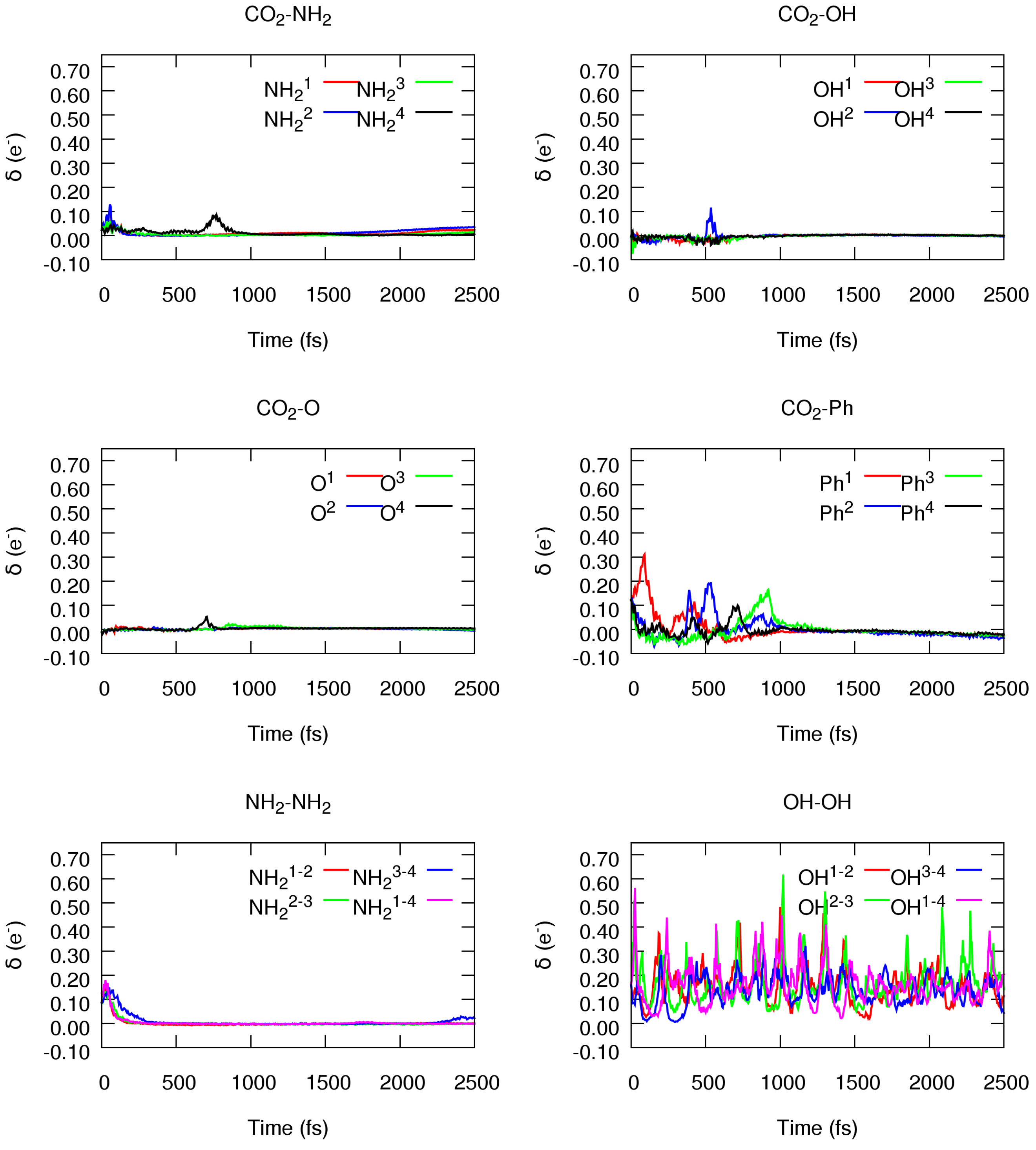

Supplementary Figure 38: **Electron delocalization in 13P-CO_2 complex at 900 K.** Evolution of the electron delocalization, δ, estimated by SchNet4AIM, between the main scaffolds of the 13P-CO_2 system throughout the simulation. All values are given in electrons, e^-. Source data are provided as a Source Data file.

Supplementary Note 15. Electronic/Geometrical correlation maps

The following figure shows the individual components comprising a correlation map used to show the correspondence between the electronic and geometrical features in the description of a given supramolecular binding event.

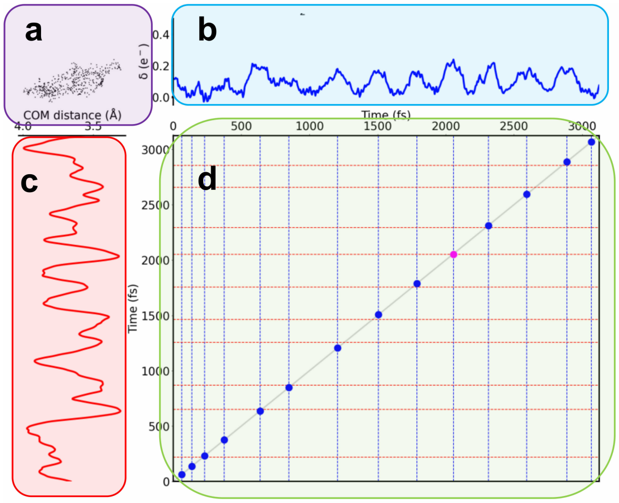

a.Dispersion plot: representation of the COM distance vs the SchNet4AIM DI predictions throughout the MD simulation.
b.Evolution of the DI values: representation of the evolution of the SchNet4AIM estimated DI values with time.
c.Evolution of the COM distance: representation of the evolution of the COM distance with time. The x and y axes have been rotated 90 degrees.
d.DI vs COM distance correlation: representation of the correlation between the local maxima in the DI (shown as blue lines and dots) and the local minima in the COM distance (shown as red lines). Magenta dots indicate the frames corresponding simultaneously to a maxima and minima in the DI and COM distance values, respectively.

Supplementary Figure 39: **A prototypical correlation map**. General diagram of the four components of a prototypical correlation map between the electronic (δ) and geometrical descriptors. The labels DI and COM are used to denote the delocalization index (i.e electron delocalization) and the center of mass, respectively.

Supplementary Note 16. Validating SchNet4AIM predictions in the 13P-CO_2 (900 K) MD simulation.

In order to check the reliability of the SchNet4AIM predictions, well beyond the interpolation domains of the proof-of-concept models shown so far, a set of reference calculations were performed using the 13P-CO_2 complex simulation (900 K) as a prototypical model.

All computations, including single-point calculation, wave-function generation and QTAIM analyses, were performed in a Intel(R) Xeon(R) Silver 4114 CPU @ 2.20GHz computer equipped with a total of 40 CPUs. For the sake of efficiency, the calculations were run in parallel using 24 processors with the aid of Gaussian09 (15) and AIMAll (19) quantum chemistry suites, respectively. The default convergence and integration parameters were used throughout. In the particular case of the QTAIM analyses, one atom was computed at a time.

Taking into account the large computational cost attributed to the QTAIM analysis of the electron density of large systems, a benchmarking study was performed to check the impact of using smaller basis sets on the quality, and time, of the resultant reference calculations. Supplementary Table. 22 comprises the total time taken to compute the reference (quantum-mechanically computed) data for a single frame of the 13P-CO_2 900 K MD simulation. The latter is obtained from the addition of the times required by the single-point calculation, and subsequent wave-function generation, followed by the corresponding QTAIM analysis. The results for different basis-sets, namely STO-3G, 3-21G, def2-SVP and def2-TZVP, in combination with the reference M06-2X DFT functional, are shown.

Basis set	N_{basis}	$N_{primitives}$	t_{wfn} (s)	t_{AIM} (s)	t_{total} (s)
STO-3G	299	897	57	34736	34794
3-21G	547	897	126	18190	18315
def2-SVP	934	1583	775	24993	25768
def2-TZVP	1845	2953	3525	38310	41835

Supplementary Table 22: **Timings involved in the quantum mechanical (conventional) QTAIM calculation of the 13P-CO_2 complex.** Times required for single-point calculation and wave-function generation, t_{wfn}, along with the QTAIM analyses of the electron density, t_{AIM}, of one of the points of the 13P-CO_2 MD trajectory (900 K). The total time arising from the addition of the previous contributions, t_{total}, is also reported. All values are given in seconds. The labels N_{basis} and $N_{primitives}$ refer to the number of basis and primitives used at each level of theory.

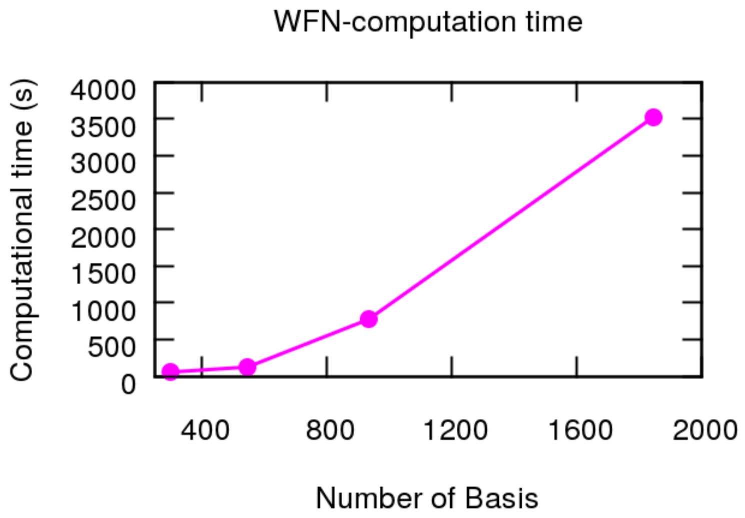

Supplementary Figure 40: **Time required for single-point calculation and wave-function generation as a function of the number of basis.** Evolution of the time required to perform a single-point calculation, and the subsequent wave-function generation, of a single frame of the 13P-CO_2 MD trajectory (900 K) as a function of the number of basis. Source data are provided as a Source Data file.

As expected, the net computational time involved in the wave-function generation grows very rapidly with the number of basis functions. This becomes even more evident from Supplementary Fig. 40, which seems to point out that the cost of this computation follows an $\mathcal{O}(N^2)$-$\mathcal{O}(N^3)$ scaling law. Under this scenario, achieving the exact same level of theory of the reference data used to train the underlying models (i.e def2-TZVP) would take $\approx$ 1 hour per frame, just to obtain the corresponding wave-function.

Similarly, the computational cost of the QTAIM analysis, rapidly scales with the quality of the basis set used to describe the system, which seems to follow a fairly linear scaling, $\mathcal{O}(N)$, with the number of basis functions. It should be noted that such a general trend is not fulfilled for the minimal basis description, STO-3G, which, as reflected in Supplementary Fig. 41, is almost as expensive as our golden standard (def2-TZVP). This effect is endemic to the use of very low quality basis sets, which provide a very poor description of the electron density field, in combination with adaptative integration grids. In fact the latter generally yields rough scalar fields which hamper their topological analyses and in turn result in longer computational times. Using fixed-size grids would likely result in considerably shorter computational times at the expense of lower numerical accuracies. Finally, and as expected, it is worth mentioning that the QTAIM calculation is the major bottleneck in the obtention of the reference data, taking $\approx$ 11 hours per MD frame at the M06-2X/def2-TZVP level of theory.

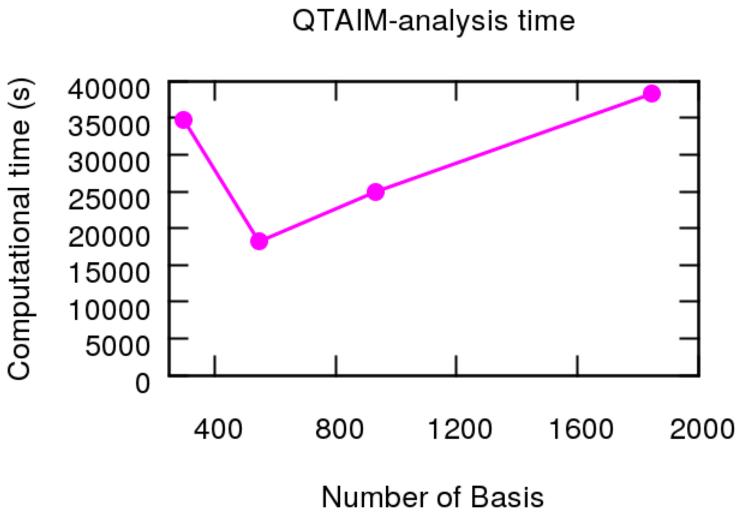

Supplementary Figure 41: **Time required for the QTAIM analysis of the electron density as a function of the number of basis.** Evolution of the time required to perform the QTAIM analysis of the electron density of a single frame of the 13P-CO_2 MD trajectory (900 K) as a function of the number of basis. Source data are provided as a Source Data file.

Finally, Supplementary Table. 23 gathers the errors, reported in terms of the L1 and L2 norms, made in the quantum mechanical calculation of the atomic charges (Q) along with the localization (λ) and delocalization (δ) indices of the 13P-CO_2 snapshot with different basis sets. All the errors are reported with respect to the results found for the reference methodology (M06-2X/def2-TZVP).

Basis set	MAE_Q	$RMSE_Q$	MAE_λ	$RMSE_\lambda$	MAE_δ	$RMSE_\delta$
STO-3G	0.091	0.109	0.086	0.108	0.001	0.008
3-21G	0.076	0.116	0.070	0.112	0.001	0.008
def2-SVP	0.053	0.066	0.050	0.070	0.001	0.004

Supplementary Table 23: **Offsets in the QTAIM analysis of the electron density for different basis sets.** Mean Absolute Errors (MAE) and Root Mean Squared Errors (RMSE) made by the conventional QTAIM analyses of the electron density of a single frame of the 13P-CO2 complex (900 K) computed at the M06-2X level of theory in combination with different basis sets. The errors are reported with respect to the def2-TZVP results, taken as reference. All values are given in electrons.

As can be seen from the latter, the results are slightly dependent on the basis set employed. Despite such an offset (in the range of 10^{-2}-10^{-3} electrons for the Mean Absolute Errors, MAEs), already reported in similar bonding analyses

studies, (22) we expect the relative values to follow similar behaviors across the overall trajectory. Considering this, and accounting for the decent compromise between accuracy and computational time, we have decided to run the quantum-chemical calculations on the 13P-CO_2 MD trajectory (900 K) at the M06-2X/3-21G level of theory. However, and considering the large computational cost involved in this process, single point calculations were performed for one in every 4 frames of the first 1000 fs of the trajectory (as it is precisely within this period where relevant binding events take place). The remaining parameters were left unmodified with respect to those indicated in the main manuscript.

Supplementary Figs. 42-43, show the evolution of the quantum mechanically computed (left) and SchNet4AIM predicted (right) group electron delocalizations of the 13P-CO_2 complex throughout the 900 K MD simulation. It should be noted that due to numerical integration errors some of the reference calculations had to be discarded, whereas the truly computed values are shown as squared points connected by interpolation lines.

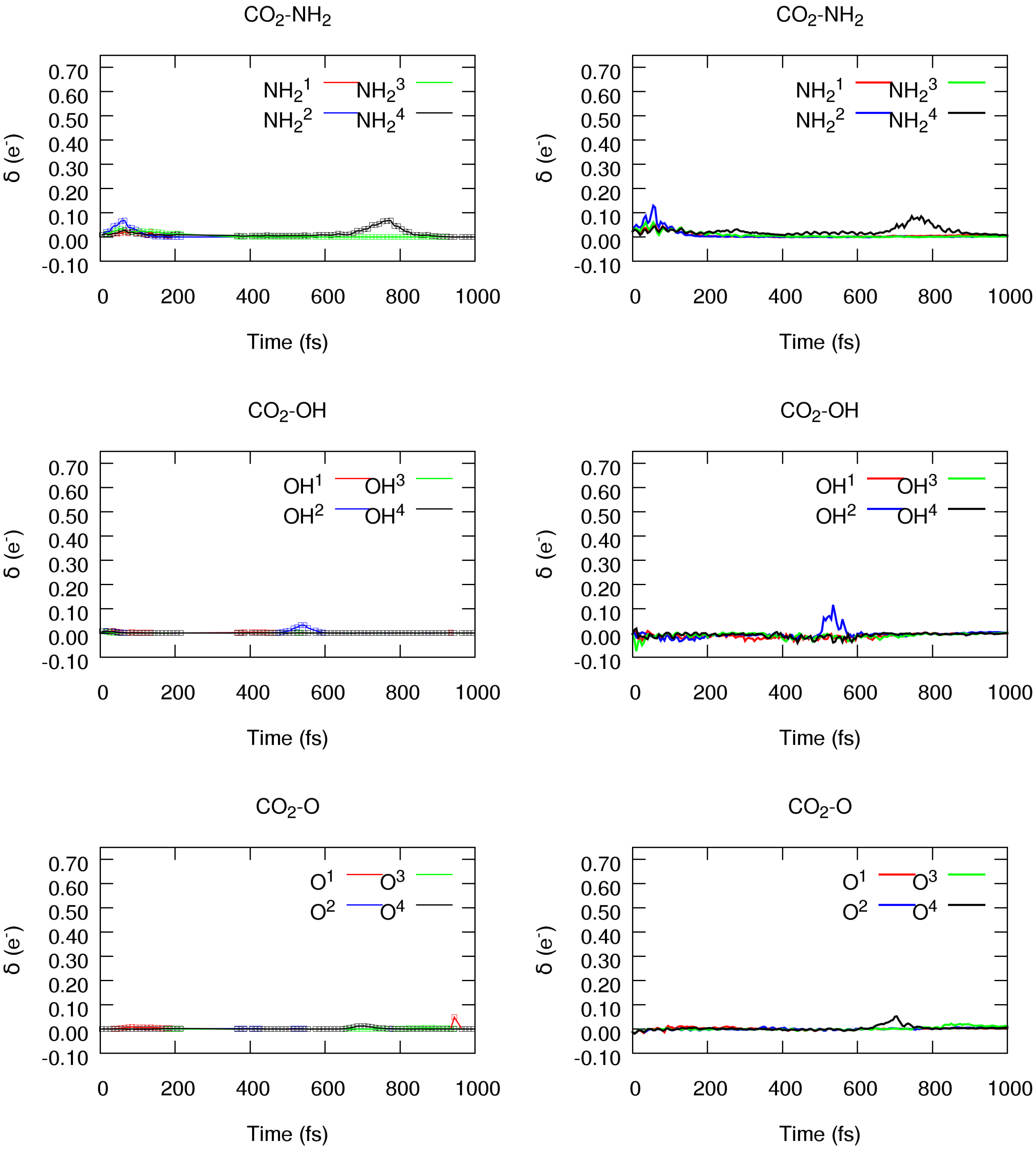

Supplementary Figure 42: **Accuracy of the predicted electron delocalization in the 13P-CO_2 complex at 900 K (A).** Evolution of the quantum-mechanically computed (left) and SchNet4AIM estimated (right) electron delocalization (δ) between the main scaffolds of the 13P-CO_2 system throughout the simulation. In the left panels, the computed frames across the MD trajectory are shown as points, connected by interpolation curves. The results for M06-2X/3-21G gas-phase calculations, taken as reference, are shown for the latter. All values are given in electrons (e^-). Source data are provided as a Source Data file.

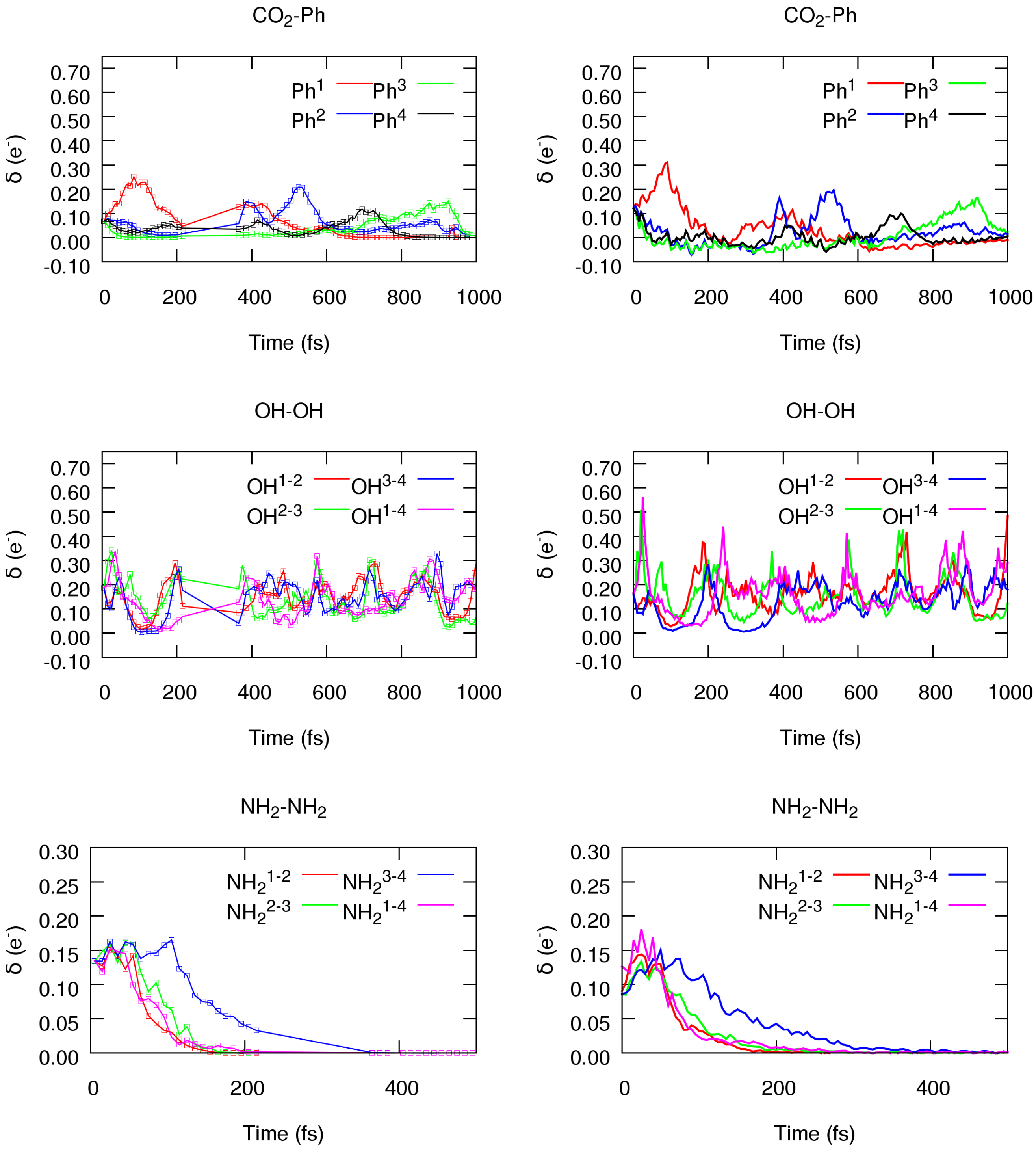

Supplementary Figure 43: **Accuracy of the predicted electron delocalization in the 13P-CO_2 complex at 900 K (B).** Evolution of the quantum-mechanically computed (left) and SchNet4AIM estimated (right) electron delocalization (δ) between the main scaffolds of the 13P-CO_2 system throughout the simulation. In the left panels, the computed frames across the MD trajectory are shown as points, connected by interpolation curves. The results for M06-2X/3-21G gas-phase calculations, taken as reference, are shown for the latter. All values are given in electrons (e^-). Source data are provided as a Source Data file.

From the latter it becomes evident that, in accordance with the trends previously discussed throughout of the manuscript, SchNet4AIM is capable of accurately predicting the group electron delocalization even in supramolecular systems, far away from the training domains of the underlying models. In addition to an ideal qualitative agreement in

the evolution of the QTAIM metrics, the quantitative values are in decent consonance. This is particularly noteworthy since, due to the use of different basis sets for the training and reference calculations used in this case, a quantitative agreement between the two is not necessarily guaranteed.

Supplementary Table. 24 gathers the errors, reported in terms of the MAE and RMSE metrics, made by SchNet4AIM in the prediction of the group electron delocalization of the studied chemical contacts. All values, given in electrons, are reported relative to the M06-2X/3-21G quantum chemical calculations, taken as reference. As can be seen, SchNet4AIM accurately predicts the group DIs even in such extrapolation regimes, showing very reasonable errors in the range of 10^{-3} to 10^{-2} electrons. Besides this, there is a remarkable qualitative agreement in the evolution of the reference and predicted binding events, as evidenced from the previous figures.

Group DI	MAE	RMSE
CO_2-NH_2^1	0.006	0.009
CO_2-NH_2^2	0.005	0.010
CO_2-NH_2^3	0.004	0.006
CO_2-NH_2^4	0.010	0.012
NH_2^1-NH_2^2	0.005	0.007
NH_2^1-NH_2^4	0.003	0.006
NH_2^2-NH_2^3	0.006	0.012
NH_2^3-NH_2^4	0.007	0.014
OH^2-OH^3	0.025	0.037
OH^1-OH^2	0.021	0.032
OH^1-OH^4	0.025	0.034
OH^3-OH^4	0.019	0.025
CO_2-O^1	0.004	0.007
CO_2-O^2	0.003	0.005
CO_2-O^3	0.005	0.007
CO_2-O^4	0.005	0.008
CO_2-Ph^1	0.037	0.042
CO_2-Ph^2	0.032	0.039
CO_2-Ph^3	0.036	0.040
CO_2-Ph^4	0.031	0.035
CO_2-OH^1	0.012	0.015
CO_2-OH^2	0.012	0.016
CO_2-OH^3	0.012	0.017
CO_2-OH^4	0.010	0.013

Supplementary Table 24: **Errors in the estimation of the group electron delocalization in the 13P-CO_2 complex at 900 K.** Error metrics, reported in terms of the Mean Absolute Error (MAE) and Root Mean Squared Error (RMSE) in the SchNet4AIM prediction of the group electron delocalizations throughout the 13P-CO_2 900 K simulation. All values, corresponding to the first 1000 fs of the simulation, are measured (in electrons) with respect to the M06-2X/3-21G quantum chemical calculations, taken as reference.

Supplementary Note 17. Computational efficiency.

The current section comprises a brief study of the computational efficiency of SchNet4AIM in the prediction of the QTAIM electronic metrics of a system, when compared to conventional quantum mechanical calculations. For such a purpose, the QTAIM electron metrics (atomic charge, Q, localization, λ, and delocalization, δ indices) were computed for a set of progressively larger systems, gathered in Supplementary Fig. 44, with SchNet4AIM and conventional (quantum mechanical) approaches.

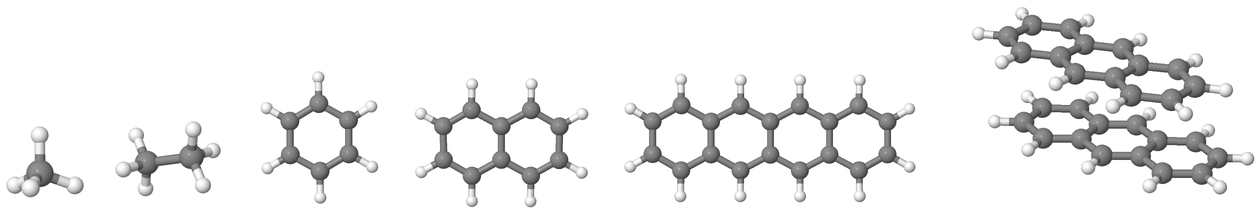

(a) N=10 (b) N=18 (c) N=42 (d) N=68 (e) N=120 (f) N=188

Supplementary Figure 44: **Prototypical molecules used to check the computational efficiency of SchNet4AIM.** Model systems employed to test the computational efficiency of SchNet4AIM when compared to conventional calculations. The labels (N) show the number of electrons for each molecule. Source data are provided as a Source Data file.

In order to achieve a fair comparison between the conventional and SchNet4AIM calculations, all were performed under the same scenario. More specifically, all computations, including single-point calculation, wave-function generation and QTAIM analyses, were performed in a Intel(R) Xeon(R) Silver 4114 CPU @ 2.20GHz computer equipped with a total of 40 CPUs. For the sake of efficiency, the calculations were run in parallel using 24 processors with the aid of Gaussian09 (15) and AIMAll (19) quantum chemistry suites, respectively. The default convergence and integration parameters were used throughout. In the particular case of the QTAIM analyses, one atom was computed at a time.

Supplementary Table. 25 collects the time required for wave-function generation, t_{wfn}, and subsequent QTAIM analysis of the electron density, t_{AIM}, for the systems shown in Supplementary Fig. 44. As expected, the total computational

N_{elec}	N_{atoms}	N_{basis}	$N_{primitives}$	t_{wfn} (s)	t_{AIM} (s)	t_{total} (s)
10	5	55	83	2	46	47
18	8	98	150	3	96	99
42	12	222	354	21	225	245
68	18	358	574	124	451	575
120	30	630	1014	563	1098	1661
188	48	988	1588	4134	16372	20506

Supplementary Table 25: **Time involved in the conventional calculation of QTAIM electronic metrics.** Evolution of the times involved in a conventional (quantum-mechanics based) calculation of the QTAIM electron metrics of progressively larger systems. The total times, t_{total}, account for the time required fro wave-function generation, t_{wfn} , and subsequent QTAIM analysis of the electron density field, t_{AIM}. Additionally, the number of electrons, N_{elec}, atoms, N_{atoms}, basis functions, N_{basis}, and primitives, $N_{primitives}$, for each system are also shown.

time involved in the estimation of the QTAIM electron metrics through conventional approaches grows exponentially with the size of the system. This becomes even more evident from Supplementary Fig. 45, which suggests that the conventional computational cost monotonously increases with the number of centers, electrons and basis functions. In fact, such a rapid growth means that the latter becomes quickly prohibitive for medium size systems, and specially those bearing heavy atoms.

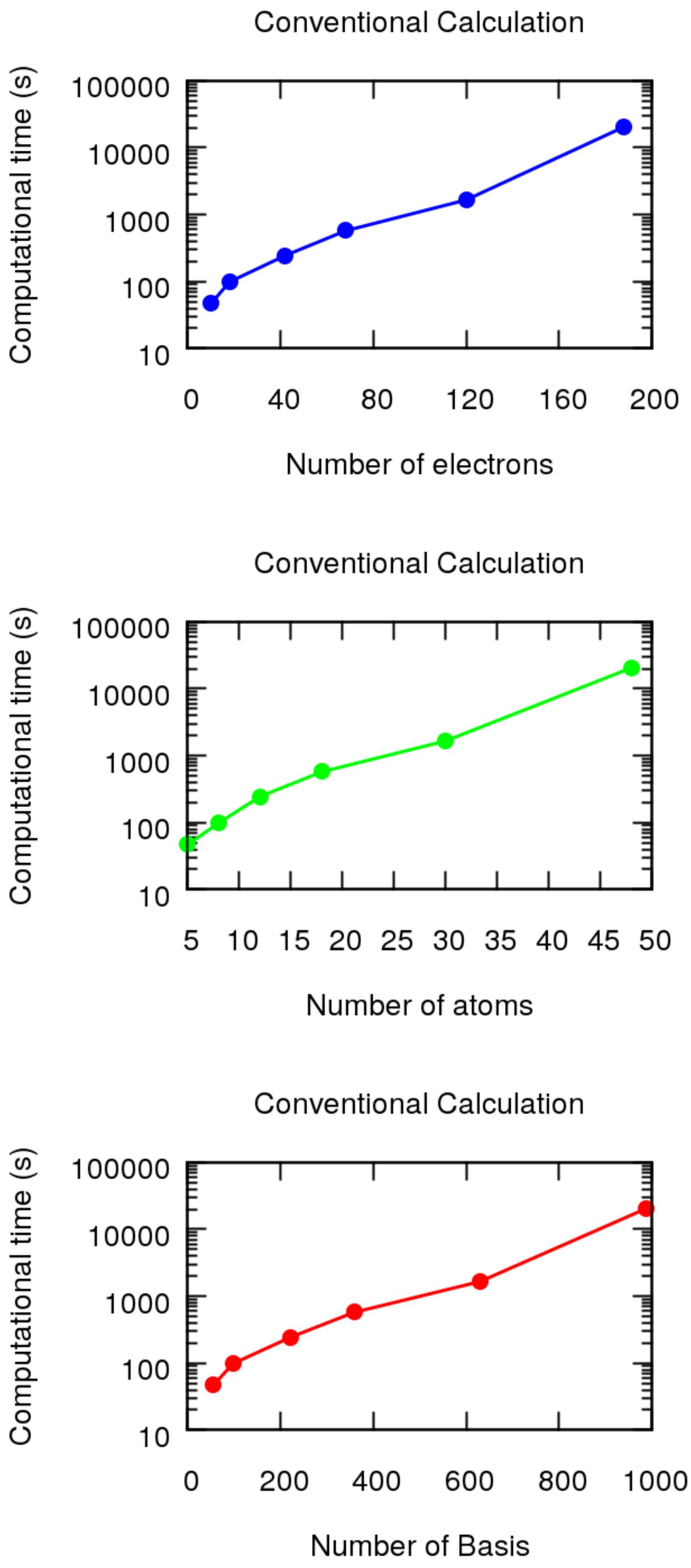

Supplementary Figure 45: **Evolution of the conventional computational times with the size of the system.** Evolution of the computational time, in seconds, required by a conventional calculation of the QTAIM electron metrics as a function of the number of electrons, atoms and basis employed in the calculation. For the sake of clarity, a logarithmic scale has been used for the y-axis. Source data are provided as a Source Data file.

By the same token, Supplementary Fig. 46 and Supplementary Table. 26 comprise the time required by SchNet4AIM in the prediction of the QTAIM electronic metrics, along with the speedup provided with respect to conventional calculations, as a function of the size of the system. It should be noted that since SchNet4AIM only relies on the atomic coordinates and their chemical identities, the results are only dependent on the number of atoms and not on the number of electrons nor basis functions.

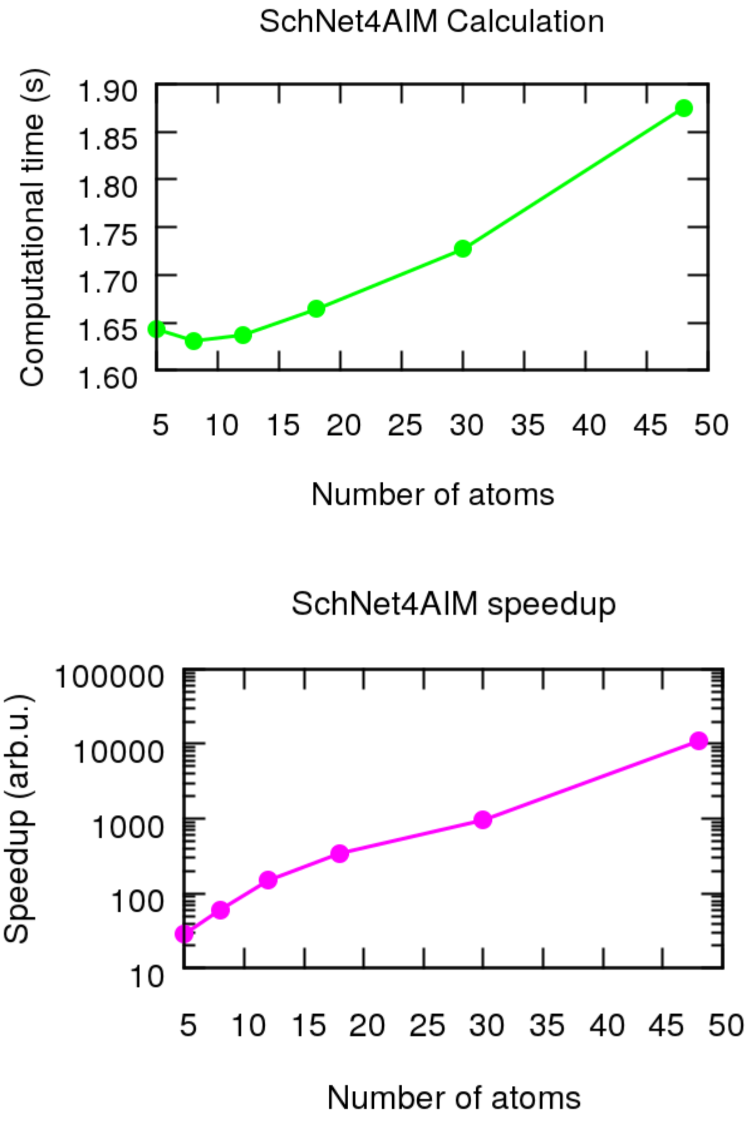

Supplementary Figure 46: **Evolution of the SchNet4AIM computational times with the size of the system.** (Left) Evolution of the computational time, in seconds, required by SchNet4AIM to predict the QTAIM electron metrics as a function of the number of atoms of the system. (Right) Evolution of the computational speedup provided by SchNet4AIM, with respect to conventional calculations performed under the same scenario, in the estimation of the QTAIM electronic metrics as a function of the number of atoms of the system. For the sake of convenience, a logarithmic scale is used for the y-axis. Source data are provided as a Source Data file.

As can be seen, despite the SchNet4AIM computational times increase with the number of atoms their magnitude is insignificant (less than 2 seconds for the largest systems) when compared to standard calculations. Moreover, for very small systems the time seems to saturate at ≈ 1.6 seconds. This observation is likely to arise from the time involved in the loading and initialization of the required modules and libraries. Accounting for such time-delay results in nearly insignificant computational costs (< 1 second). In this way, SchNet4AIM is able to provide enormous speedup factors, of up to 10^4 within the explored prototypical systems, when compared to conventional calculations, something which becomes more pronounced as the size of the system increases.

N_{atoms}	t_{S4AIM} (s)	Speedup
5	1.64	29
8	1.63	61
12	1.64	150
18	1.66	346
30	1.73	962
48	1.88	10937

Supplementary Table 26: **Computational times and speedup of SchNet4AIM in the prediction of QTAIM electron metrics.** Times required by SchNet4AIM in the estimation of the QTAIM electron metrics, t_{S4AIM}, along with their speedups, as a function of the number of atoms of the system. The speedup factors are reported relative to the conventional (quantum mechanics based) calculations.

Finally, Supplementary Table. 27 gathers the time required for the training of SchNet4AIM models targeted at the prediction of different QTAIM electron metrics. Training on both CPU and GPU based computer nodes are reported. Whereas for the former, the previously detailed computational resources were employed (Intel(R) Xeon(R) Silver 4114 CPU @ 2.20GHz), GPU testing was achieved on a single NVIDIA GeForce GTX 750 Ti GPU with 640 CUDA cores and 2GB of dedicated memory. As can be seen from this table, training on two-particle quantities is considerably more

Property	Module	Time (h)	Node	Epochs	N_{molec}	N_{local}
Q	AIMwise	6.1	20 CPUs	287	3100	69160
Q	ElementalAIMwise	6.4	20 CPUs	262	3100	69160
λ	AIMwise	5.9	20 CPUs	279	3100	69160
λ	ElementalAIMwise	7.5	20 CPUs	305	3100	69160
δ	AIMwise	138.9	1 GPU	322	3100	799549
δ	ElementalPairAIMwise	143.6	1 GPU	312	3100	799549

Supplementary Table 27: **SchNetAIM training times**. Time required for the learning stage of the SchNet4AIM models trained on the QTAIM-electronic metrics database, reported in hours. The results for different properties learned through various output modules (Module) are shown. Both CPU and GPU-based computer nodes were employed, for 1P and 2P properties, respectively. The number of epochs required for the training (Epochs) as well as the size of the training subset, both in terms of molecular, N_{molec}, and local, N_{local}, properties are also reported. The results for the atomic charge, Q, along with the localized (λ) and delocalized (δ) electron counts are shown.

expensive when compared with their atomic (one-body) counterparts. Such a finding is not surprising at all, owing to the very rapid growth of pairwise components with the number of constituting atoms in a molecule. Indeed, this is clearly reflected from the number of local terms comprising the training subsets: the net 3100 molecular instances found in the QTAIM electronic database account for a total of $\approx 7 \cdot 10^4$ and $8 \cdot 10^5$ one-body and two-body local datapoints, respectively.

In this regard, and generally speaking, we expect the influence of the training dataset on the model's performance to be highly dependent on several factors, including:

- The model hyper-parameters, and specially regarding the SchNet4AIM representation employed. Imposing more local representations reduces the diversity and size of the dataset required to train SchNet4AIM at the expense of, probably, reducing the prediction accuracy.
- The quality and nature of the reference data. Often, high quality data help the models to identify the particular combination of input features that result in a given output, reducing the required size of the training data.
- The size of the molecular instances included in the database. Since SchNet4AIM is trained on local properties, the number of datapoints in a given molecular instances scales linearly (N) or quadratically ($N * (N-1)/2$) with the size of the system. As such, larger molecules provide more local information than their smaller counterparts, reducing the number of training points required throughout the learning stages.

Moreover, we expect the implementation of active learning protocols to come particularly handy in this regard, and specially as far as the transferability of the models is concerned.

Supplementary Note 18. Explaining SchNet4AIM predictions.

In this section, we demonstrate how the physically rigorous local properties used by SchNet4AIM in the reconstruction of group quantities pave the way towards a proper understanding of the behavior of the latter. We illustrate this by identifying the pairwise contributions that govern the aforementioned supramolecular binding events in the 13P-CO_2 complex. First, we show which are the most relevant contributions to the overall interaction between CO_2 and the OH and NH_2 moieties of the 13P skeleton at 300 K. Then, we delve into the slightly more complex 900 K simulation, showing how the interplay of vanishing and emerging contacts is responsible for the observed supramolecular binding events throughout the process.

13P-CO_2 simulation (300 K)

Supplementary Fig. 47 shows the most relevant pairwise interactions dominating the net electron delocalization between CO_2 and the NH_2 moieties of 13P throughout the 300 K MD simulation, as predicted by SchNet4AIM.

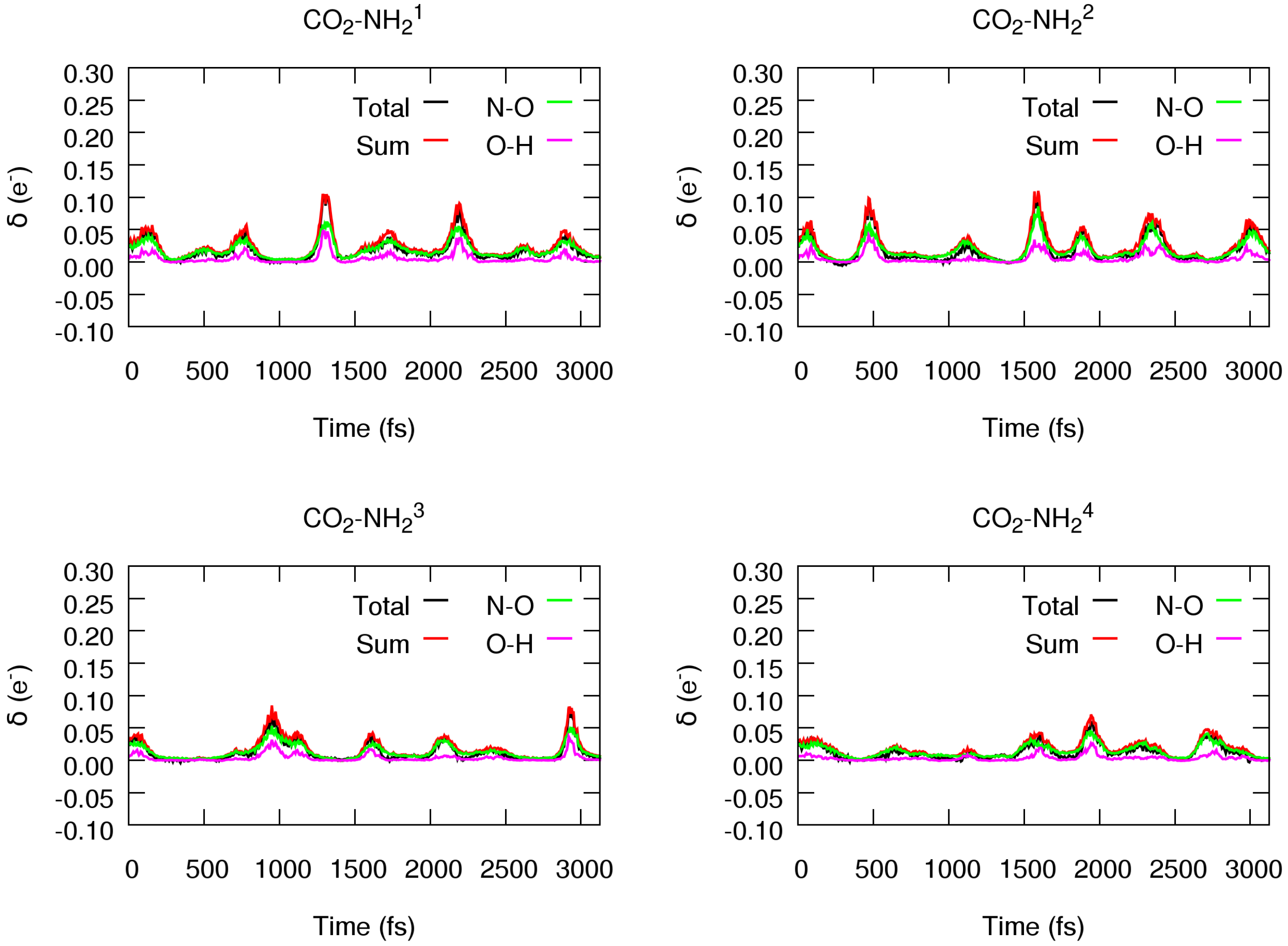

Supplementary Figure 47: **Explained CO_2-NH_2 interactions at 300 K.** Evolution of the SchNet4AIM predictions of the CO_2-NH_2 group electron delocalization, δ, (black) along with the contribution of the most dominant pairwise terms throughout the 13P-CO_2 MD simulation at 300 K. The additive contribution of the latter terms, Sum, is also shown in red. All values are given in electrons, e^-. Source data are provided as a Source Data file.

As can be seen from the latter, in all cases, the global behavior of the CO_2-NH_2 group delocalization is governed by two very well defined components explicitly involving the terminal O atom of CO_2 which is directly pointing towards the bottom of the cage. As represented in Supplementary Fig. 48, these comprise the interactions with the N (O-N, shown in green) and H atoms involved in the NH_2-NH_2 HB network (O-H, shown in pink). Indeed, the cumulative

contribution of just these two terms (shown in red in Supplementary Fig. 47) is able to reconstruct almost exactly for the net group DI (highlighted in black). These observations are in perfect agreement with our intuition, showing an almost negligible contribution of the outer H atoms of the NH_2 scaffolds which being very distant to, and not oriented towards, the aforementioned O atom have little to no impact on the net group DIs.

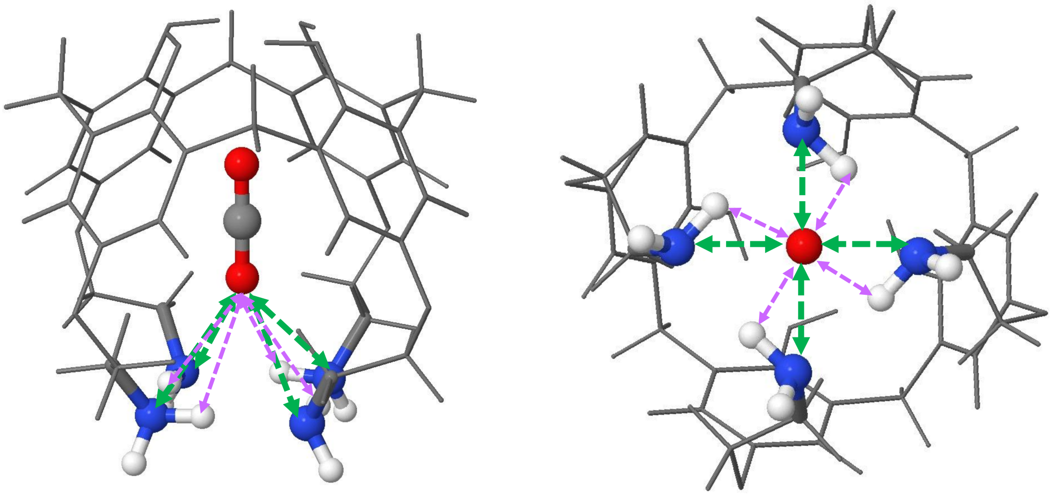

Supplementary Figure 48: **Dominant interactions in the CO_2-NH_2 group electron delocalization (300 K).** Schematic representation of the O-N and O-H contacts, shown in pink and green respectively, dominating the CO_2-NH_2 electron delocalization index. Front (left) and bottom (right) views are shown.

Interestingly enough, strikingly different observations are found when studying the net binding between the CO_2 molecule and the OH moieties at the top of the 13P skeleton. As shown in Supplementary Fig. 49, it is now the interaction between one of the terminal O atoms of CO_2 and that of the OH moieties, the term dominating the entire CO_2-OH delocalization, as represented in Supplementary Fig. 50. In fact, such a term, highlighted in red in Supplementary Fig. 49, seems to account almost entirely for the corresponding group delocalization, in black.

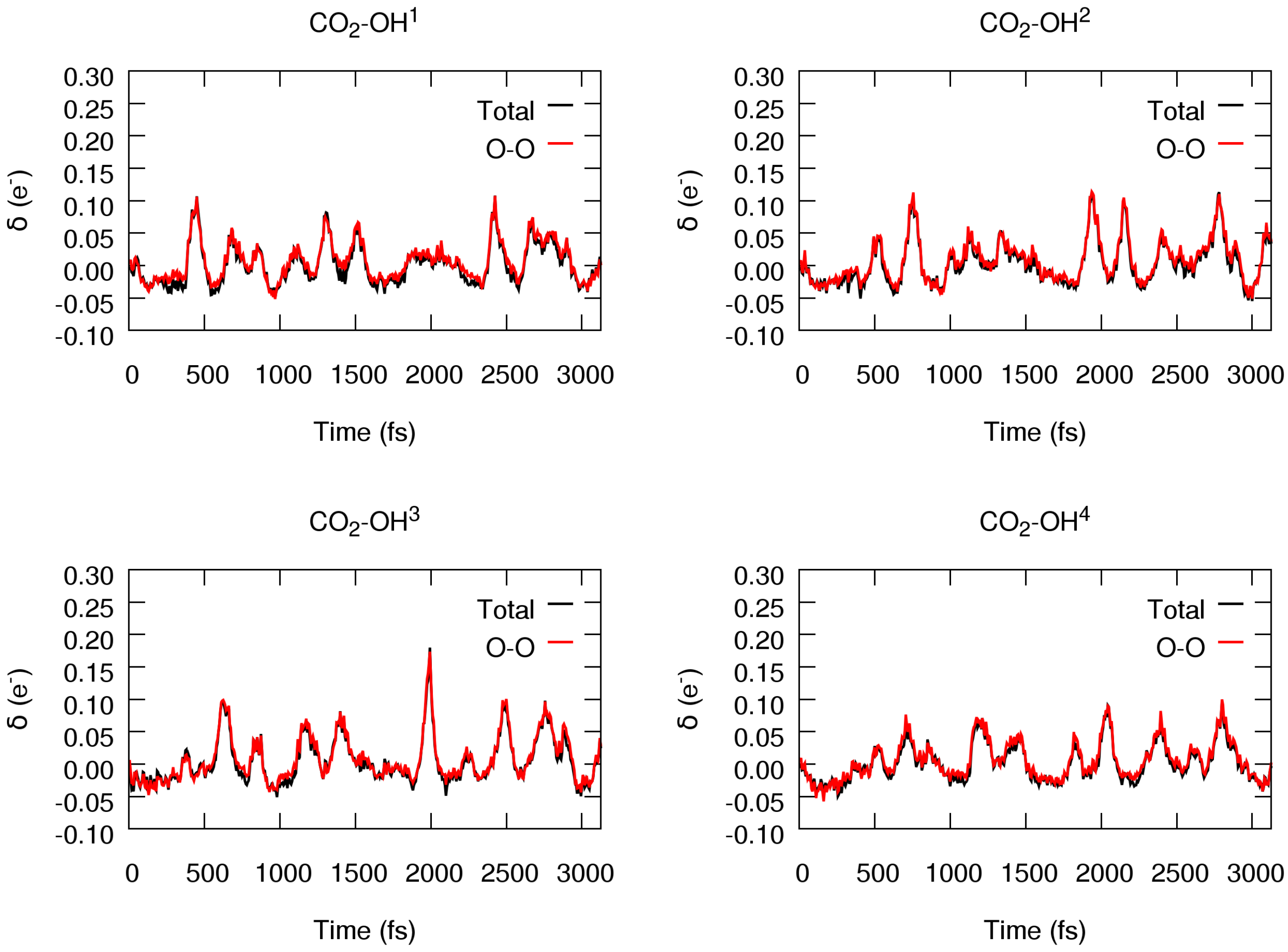

Supplementary Figure 49: **Explained CO_2-OH interactions at 300 K.** Evolution of the SchNet4AIM predictions of the CO_2-OH group electron delocalization, δ, (black) along with the contribution of the most dominant pairwise terms throughout the 13P-CO_2 MD simulation at 300 K. Since there is only one dominant pairwise term, the additive contribution is not shown. All values are given in electrons, e^-. Source data are provided as a Source Data file.

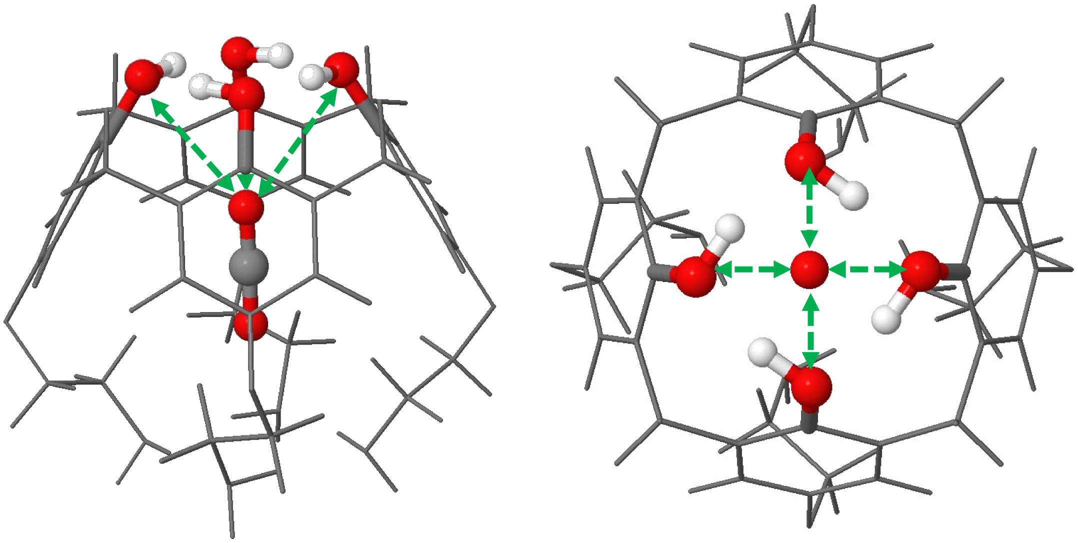

Supplementary Figure 50: **Dominant interactions in the CO_2-OH group electron delocalization (300 K).** Schematic representation of the O-H contacts, shown in green, dominating the CO_2-OH electron delocalization index. Front (left) and top (right) views are shown.

These observations point out that now the H atoms of the OH groups do not take an active role in the binding of the latter to the trapped CO_2 molecule. Such a finding could arise as a manifestation of the stronger O-H hydrogen bond network which along with the much more proton-like character of the H atoms in OH, owing to the superior electronegativity of O when compared to N, decreases its ability to share electrons with the nearby CO_2 molecule.

13P-CO_2 simulation (900 K)

Let's now delve into the slightly more complex scenario of the high-temperature MD simulation, where different well-defined binding events have been found to take place. Supplementary Figs. 51-52 comprise a collection of particularly interesting chemical contacts observed throughout such simulation, along with their explanations:

- CO_2-OH^2: as shown before, the CO_2 experiences a subtle increase in its delocalized electron count with one of the OH groups of the 13P skeleton at around 550 fs. Taking a look at the top panel of Supplementary Fig. 51 reveals that this arises entirely from an O-O interaction, shown in green, accompanied by a secondary and much more subtle O-H contact.

- CO_2-NH_2^4: as can be seen from the bottom panel of Supplementary Fig. 51, the behavior of the overall electron count between the CO_2 and NH_2^4 moieties in such a time window is entirely dominated by three well-defined contacts. Initially, the subtle group DI seems to emerge mainly from the long-distance interaction between N and one of the O atoms of CO_2, N-O_2 shown in orange. However at around 800 fs, the CO_2 molecule rotates leading to a structure where O_3 is directly facing the NH_2 moiety. This results in a very rapid increase of the O_3-H and O_3-N interactions, in green and pink respectively, yielding the observed spike in the group DI.

- CO_2-O^4: the top panel of Supplementary Fig. 52 shows the evolution of the group DI between CO_2 and the O^4 linker in the 13P skeleton. As expected, an analysis of the SchNet4AIM individual predictions reveals that the latter is controlled by the O-O interaction. As such, the latter is directly responsible for the observed spike around 700 fs, corresponding to the strengthening of such a dipole-dipole interaction, which precedes the previously discussed CO_2-NH_2^4 spike at 800 fs.

- CO_2-Ph^2: naturally, as the complexity of the groups involved increases, so does the number of active contributions to the group DI. This is directly reflected from the bottom panel of Supplementary Fig. 52 which shows that in this time regime, the interaction between CO_2 and one of the Ph scaffolds arises mainly from the interaction with the top-left skeleton of the aromatic ring. Two particularly prominent binding events are found throughout the process. The first, found at 400 fs, is attributed to the contact with one of the CH moieties, being dominated by O_2-C_d and O_2-H_a interactions (in green and orange respectively). As the simulation progresses, the CO_2 leans towards the top part of the ring, favoring the formation of a π-π interaction between both electron densities, and yielding a second spike at about 500 fs. Indeed, a closer look to the individual contributions reveals that this binding event arises from the O_3-C_a and O_3-C_b contacts, in red and blue, respectively.

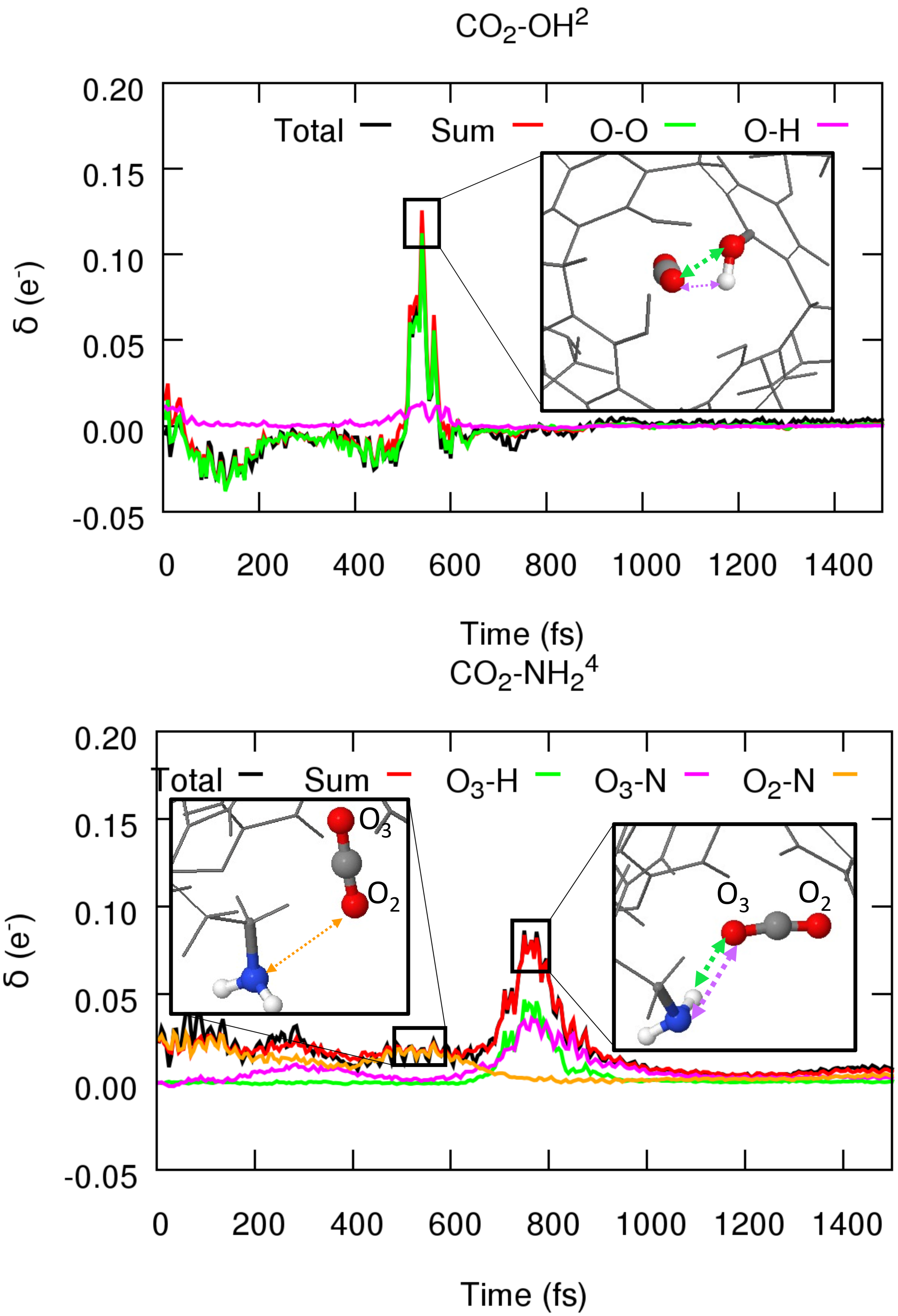

Supplementary Figure 51: **Explained CO_2-OH^2 and CO_2-NH_2^4 interactions at 900 K.** Evolution of the SchNet4AIM predictions of the CO_2-OH^2 (top) and CO_2-NH_2^4 (bottom) group electron delocalization, δ, (black) along with the contribution of the most dominant pairwise terms throughout the 13P-CO_2 MD simulation at 900 K. The additive contribution of the latter terms, Sum, is also shown in red. A schematic representation of those frames corresponding to particularly prominent binding events is shown. The results for the first 1500 fs, involving explicit 13P-CO_2 contacts, are presented. All values are given in electrons, e^-. Source data are provided as a Source Data file.

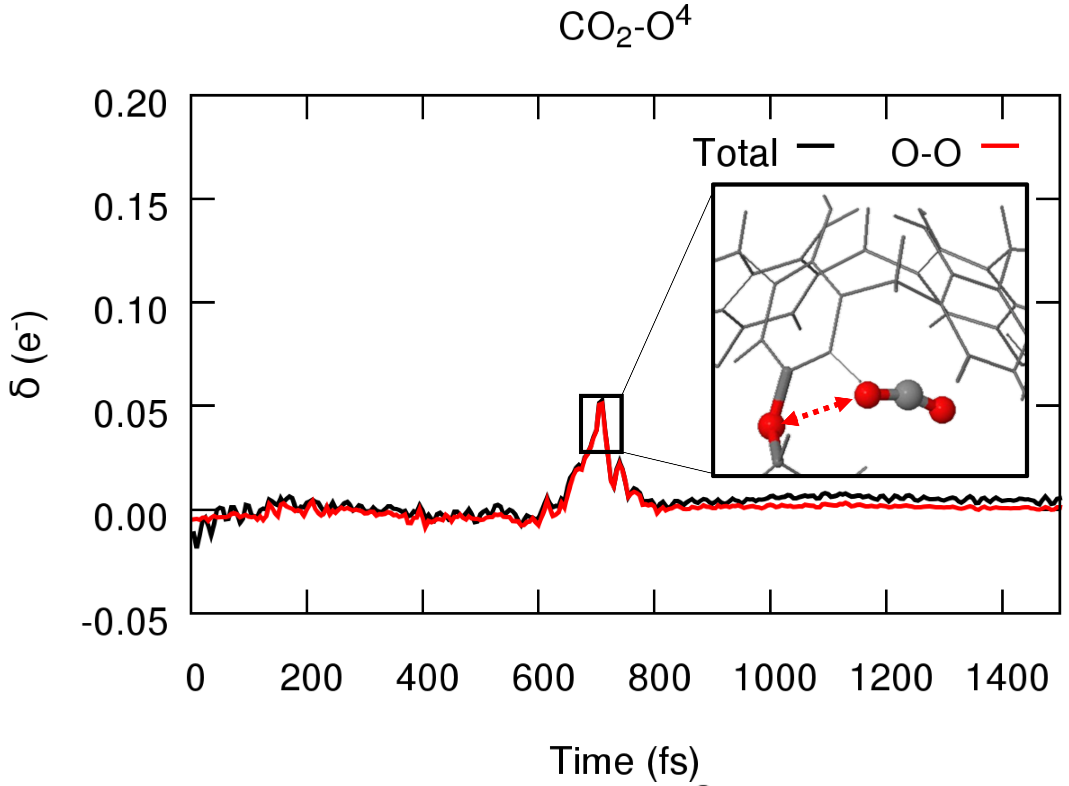

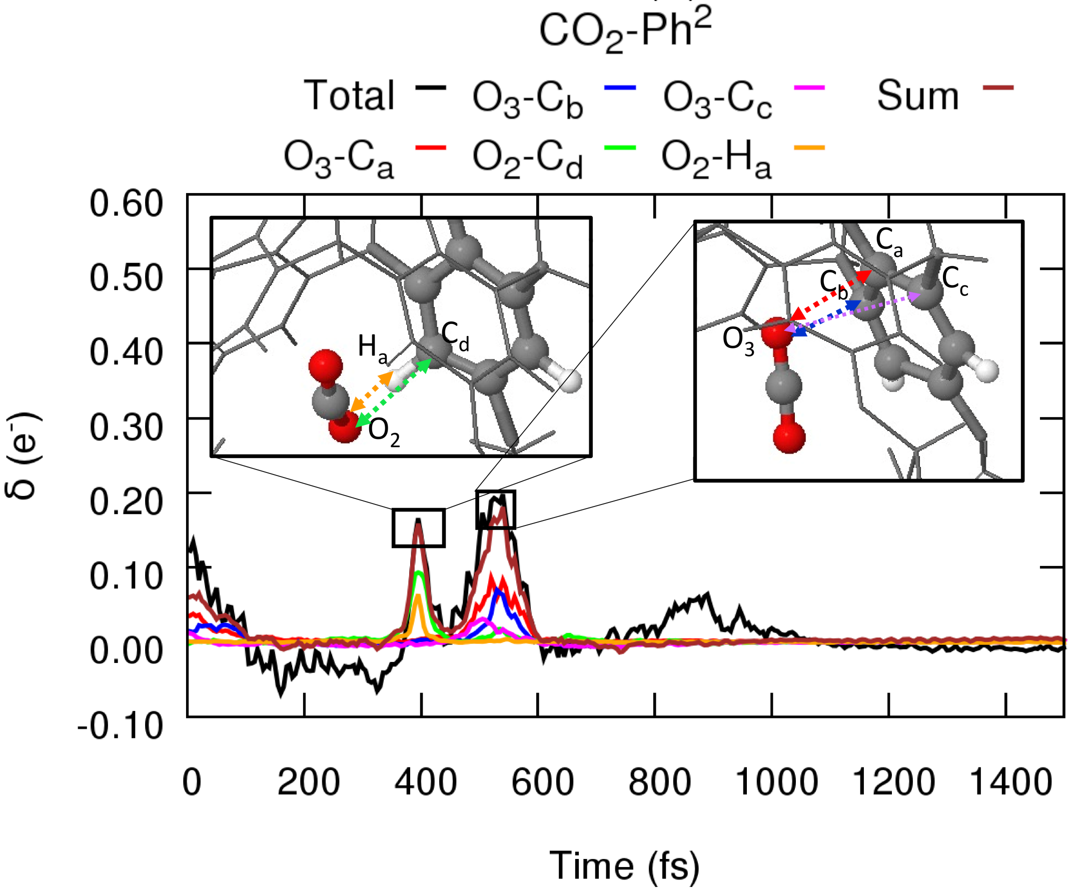

Supplementary Figure 52: **Explained CO_2-O^4 and CO_2-Ph^2 interactions at 900 K.** Evolution of the SchNet4AIM predictions of the CO_2-O^4 (top) and CO_2-Ph^2 (bottom) group electron delocalization, δ, (black) along with the contribution of the most dominant pairwise terms throughout the 13P-CO_2 MD simulation at 900 K. A schematic representation of those frames corresponding to particularly prominent binding events is shown. The results for the first 1500 fs, involving explicit 13P-CO_2 contacts, are presented. In the case of CO_2-Ph^2, the additive contribution of the most relevant pairwise terms, Sum, is also included. All values are given in electrons, e^-. Source data are provided as a Source Data file.

After showing how SchNet4AIM affords easily interpretable outputs, it may be worth discussing the limitations to the explainability of our models. It is worth recalling at this point that this is something inherent to XCAI, as this field is still in its infancy. In the particular case of the SchNet4AIM models presented so far, one can identify two main challenges in their explainability:

- On the one hand, the number of atomic pairs grows very rapidly with the size of the system. As such, identifying the most relevant pairwise interactions dominating a given process becomes more difficult with the complexity of the supramolecular system, and specially when studying the interaction between very large molecular moieties.
- On the other hand, our XCAI implementation is currently limited to explaining how molecular or group quantities are dominated by their local components, while the latter cannot be easily mapped to the input space domain. In fact, arising from an end-to-end approach, the learned SchNet4AIM features are unlikely to be easily understandable on their own. It should be noted that this is endemic to most deep learning based models, whose accuracy comes at the expense of complexity. However, the combination of SchNet4AIM with other techniques such as extrinsic explainable approaches could shed light on to this last point. In this way, we will explore in the near-future the possibility of using additional extrinsic explainability tools to further understand our local predictions in terms of the atomic identities and positions.

Supplementary Note 19. Effect of the Training Set Size on the accuracy of SchNet4AIM predictions.

With the aim of evaluating the influence of the amount of training data in the prediction accuracy of the models, SchNet4AIM was trained to predict the atomic charges of the QTAIM electronic database using a varying number of molecular training points. In order to achieve a reliable estimation of the prediction accuracy against never-seen data, fixed validation and testing subsets were employed, as indicated in Supplementary Note 8. Instead, progressively larger training sets were generated by randomly selecting molecules from the original 3100 pool, as detailed in Supplementary Note 8. For the sake of simplicity, all models were trained as specified in Supplementary Note 6, relying on the `AIMwise` output module. Supplementary Fig. 53 gathers the evolution of the errors made by SchNet4AIM in the prediction of the testing subset as a function of the number of molecular instances used to train the models. The black lines show the mean values reported for all the atomic constituents, regardless of their chemical identities.

As expected, the prediction errors against never-seen data decrease smoothly with the size of the training subset. Such a monotonous increase in prediction accuracy exhibits, however, a saturation effect, as reflected by the obvious convergent trend found in both MAE and RMSE metrics. Moreover, C and H atoms seem to converge faster (≈ 1500 points) than O and specially N, for which the error metrics seem to stabilize for around 2000 molecular instances. This finding arises as an inevitable manifestation of the lower predominance that heteroatoms have in the common CHON chemical space, which reduces the amount of atomic data available for these elements per molecular instance. Furthermore, this ultimately explains the higher errors committed, on average, for the heteroatoms when compared to more abundant elements (e.g H or C).

Altogether, these results evidence that SchNet4AIM shows quite fast-learning curves, yielding (with the particular exception of N) testing MAEs below the 0.010 electrons threshold for training datasets of just ≈ 1700 molecular instances.

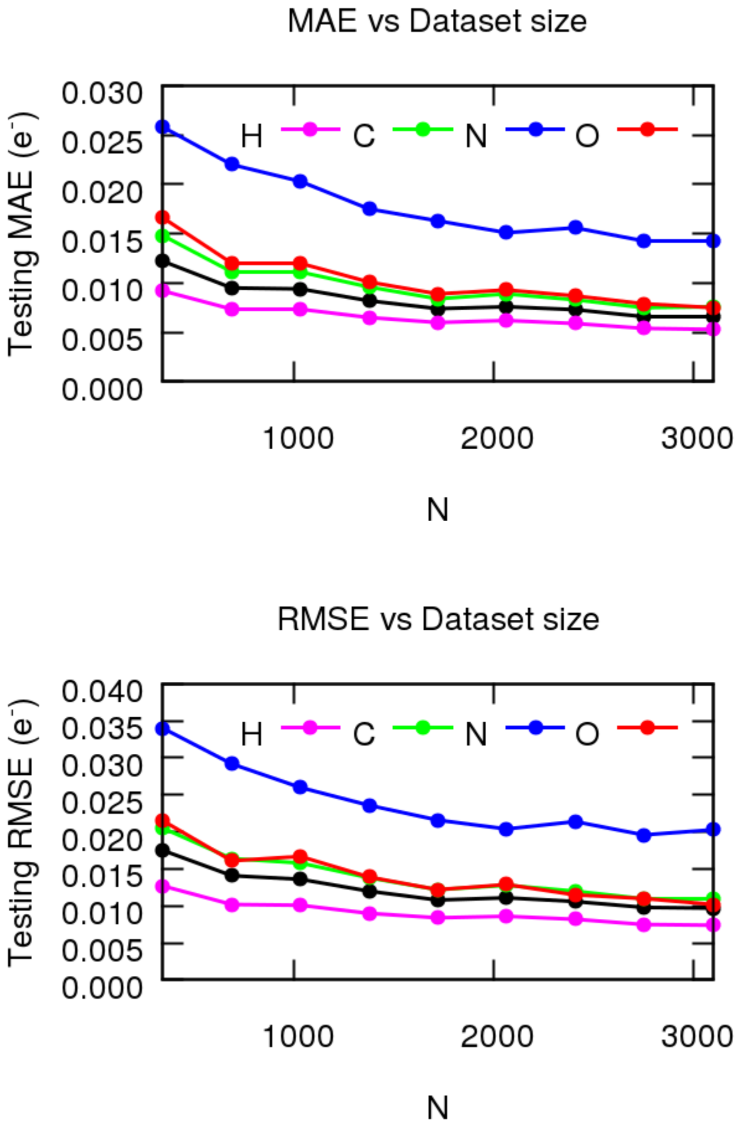

Supplementary Figure 53: **Influence of the training set size on the SchNet4AIM prediction accuracy.** Evolution of the SchNet4AIM prediction errors, reported in terms of the Mean Absolute Error (MAE, top) and Root Mean Squared Error (RMSE, bottom), in the estimation of the atomic charges of the testing subset of the QTAIM electronic database as a function of the number of points used to train the underlying models (N). The error metrics are reported in electrons. The black curve shows the mean results, regardless of the chemical identity of the atoms. N molecular points were randomly selected from the original training set, while the original (fixed-size) validation and testing subsets were used throughout. Details on these subsets are collected in Supplementary Note 8. All models were trained with the `AIMwise` output module as indicated in Supplementary Note 6. Source data are provided as a Source Data file.

References

[1] K. Schütt, P.-J. Kindermans, H. E. Sauceda Felix, S. Chmiela, A. Tkatchenko, K.-R. Müller in *Advances in Neural Information Processing Systems*, Curran Associates, Inc. https://proceedings.neurips.cc/paper_files/paper/2017/file/303ed4c69846ab36c2904d3ba8573050-Paper.pdf.

[2] K. T. Schütt, H. E. Sauceda, P.-J. Kindermans, A. Tkatchenko, K.-R. Müller, *The Journal of Chemical Physics* **2018**, *148*, 241722.

[3] K. T. Schütt, F. Arbabzadah, S. Chmiela, K. R. Müller, A. Tkatchenko, *Nature Communications* **2017**, *8*, 13890.

[4] P. Hurd, T. Cusati, M. Persico, *Journal of Computational Physics* **2010**, *229*, 2109–2116.

[5] S. Raghunathan, U. D. Priyakumar, *International Journal of Quantum Chemistry* **2021**, *122*, 26870.

[6] A. Karthikeyan, U. D. Priyakumar, *Journal of Chemical Sciences* **2021**, *134*, 2.

[7] J. Westermayr, M. Gastegger, P. Marquetand, *The Journal of Physical Chemistry Letters* **2020**, *11*, 3828–3834.

[8] T. Braeckevelt, R. Goeminne, S. Vandenhaute, S. Borgmans, T. Verstraelen, J. A. Steele, M. B. J. Roeffaers, J. Hofkens, S. M. J. Rogge, V. V. Speybroeck, *Chemistry of Materials* **2022**, *34*, 8561–8576.

[9] N. W. A. Gebauer, M. Gastegger, S. S. P. Hessmann, K.-R. Müller, K. T. Schütt, *Nature Communications* **2022**, *13*, 973.

[10] J. Zhang, J. Chen, P. Hu, H. Wang, *Chinese Chemical Letters* **2020**, *31*, 890–896.

[11] M. Shiranirad, C. J. Burnham, N. J. English, *Chemical Physics* **2022**, *552*, 111347.

[12] K. T. Schütt, P. Kessel, M. Gastegger, K. A. Nicoli, A. Tkatchenko, K.-R. Müller, *Journal of Chemical Theory and Computation* **2019**, *15*, 448–455.

[13] A. Paszke, S. Gross, F. Massa, A. Lerer, J. Bradbury, G. Chanan, T. Killeen, Z. Lin, N. Gimelshein, L. Antiga, A. Desmaison, A. Kopf, E. Yang, Z. DeVito, M. Raison, A. Tejani, S. Chilamkurthy, B. Steiner, L. Fang, J. Bai, S. Chintala in *Advances in Neural Information Processing Systems 32*, Curran Associates, Inc., **2019**, pp. 8024–8035.

[14] M. Tancik, P. P. Srinivasan, B. Mildenhall, S. Fridovich-Keil, N. Raghavan, U. Singhal, R. Ramamoorthi, J. T. Barron, R. Ng, *Fourier Features Let Networks Learn High Frequency Functions in Low Dimensional Domains*, **2020**.

[15] M. J. Frisch, G. W. Trucks, H. B. Schlegel, G. E. Scuseria, M. A. Robb, J. R. Cheeseman, G. Scalmani, V. Barone, B. Mennucci, G. A. Petersson, H. Nakatsuji, M. Caricato, X. Li, H. P. Hratchian, A. F. Izmaylov, J. Bloino, G. Zheng, J. L. Sonnenberg, M. Hada, M. Ehara, K. Toyota, R. Fukuda, J. Hasegawa, M. Ishida, T. Nakajima, Y. Honda, O. Kitao, H. Nakai, T. Vreven, J. A. Montgomery, Jr., J. E. Peralta, F. Ogliaro, M. Bearpark, J. J. Heyd, E. Brothers, K. N. Kudin, V. N. Staroverov, R. Kobayashi, J. Normand, K. Raghavachari, A. Rendell, J. C. Burant, S. S. Iyengar, J. Tomasi, M. Cossi, N. Rega, J. M. Millam, M. Klene, J. E. Knox, J. B. Cross, V. Bakken, C. Adamo, J. Jaramillo, R. Gomperts, R. E. Stratmann, O. Yazyev, A. J. Austin, R. Cammi, C. Pomelli, J. W. Ochterski, R. L. Martin, K. Morokuma, V. G. Zakrzewski, G. A. Voth, P. Salvador, J. J. Dannenberg, S. Dapprich, A. D. Daniels, O. Farkas, J. B. Foresman, J. V. Ortiz, J. Cioslowski, D. J. Fox, *Gaussian 09 Revision E.01*, Gaussian Inc. Wallingford CT 2009.

[16] F. Neese, F. Wennmohs, U. Becker, C. Riplinger, *The Journal of Chemical Physics* **2020**, *152*, 224108.

[17] M. Gallegos, J. M. Guevara-Vela and Á. Martín Pendás , *The Journal of Chemical Physics* **2022**, *156*, 014112.

[18] F. Jiménez-Grávalos, J. L. Casals-Sainz, E. Francisco, T. Rocha-Rinza, Á. Martín Pendás and J. M. Guevara-Vela, *Theoretical Chemistry Accounts* **2020**, *139*, 5.

[19] T. A. Keith, *AIMALL, TK Gristmill Software, Overland Park, KS, USA*, **2019**.

[20] Á. Martín Pendás and E. Francisco, *Promolden. A QTAIM/IQA code (Avaliable from the authors upon request).*

[21] *Jmol: an open-source Java viewer for chemical structures in 3D. http://www.jmol.org/.*

[22] M. Jablonski, M. Palusiak, *The Journal of Physical Chemistry A* **2010**, *114*, 12498–12505.
