# Supplementary figures and images for: Explainable chemical artificial intelligence from accurate machine learning of real-space chemical descriptors
Source: Nat Commun. 2024 May 21;15:4345. doi: 10.1038/s41467-024-48567-9 (PMC11522690; doi:10.1038/s41467-024-48567-9)

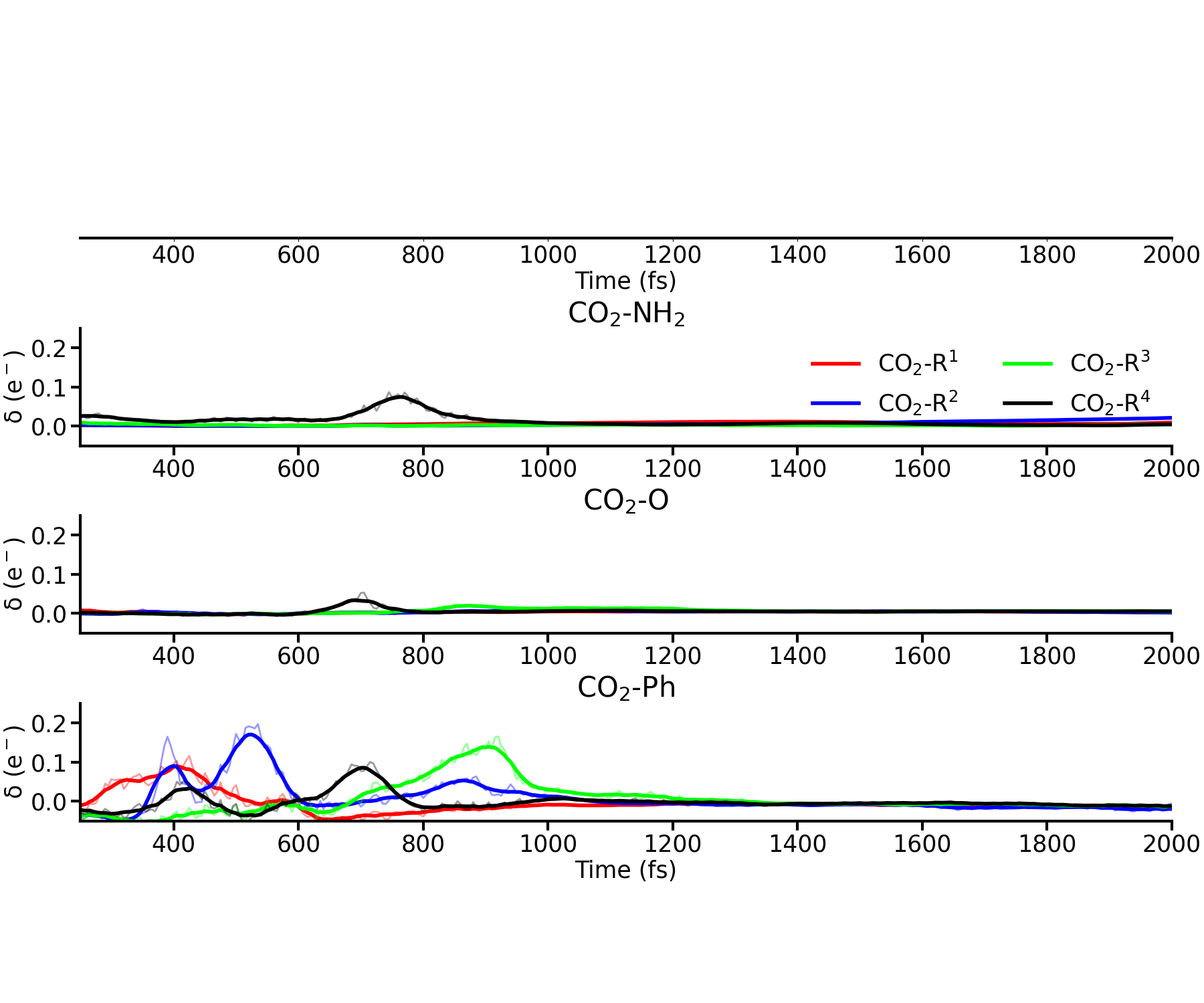

Supplement: Supplementary file 4 — Source Data [file 41467_2024_48567_MOESM4_ESM.zip › main/Fig6/CO2_O_NH2_PH_interactions.png]

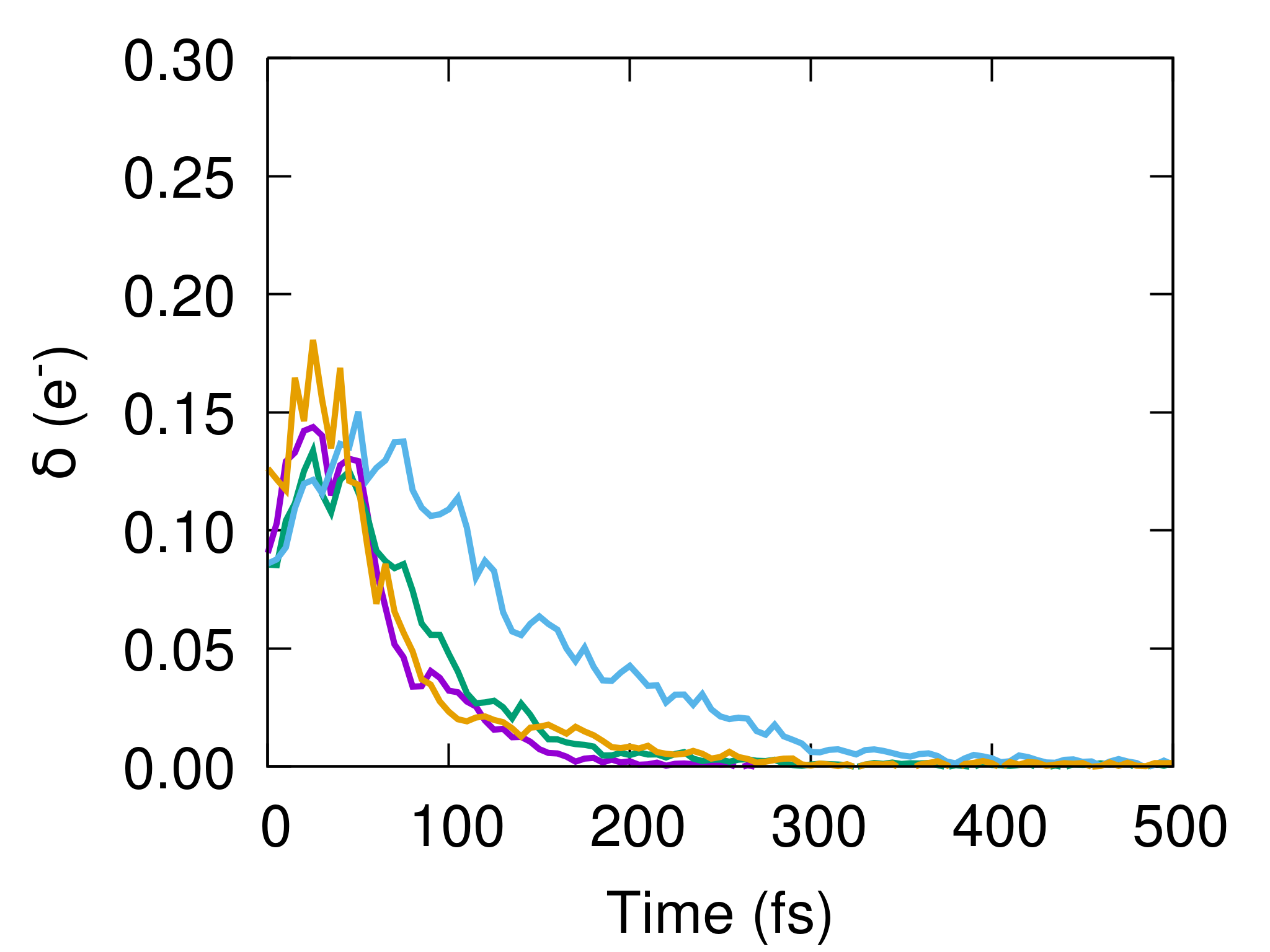

Supplement: Supplementary file 4 — Source Data [file 41467_2024_48567_MOESM4_ESM.zip › main/Fig5/C/13P_HT_NH2_NH2_closeup.png]

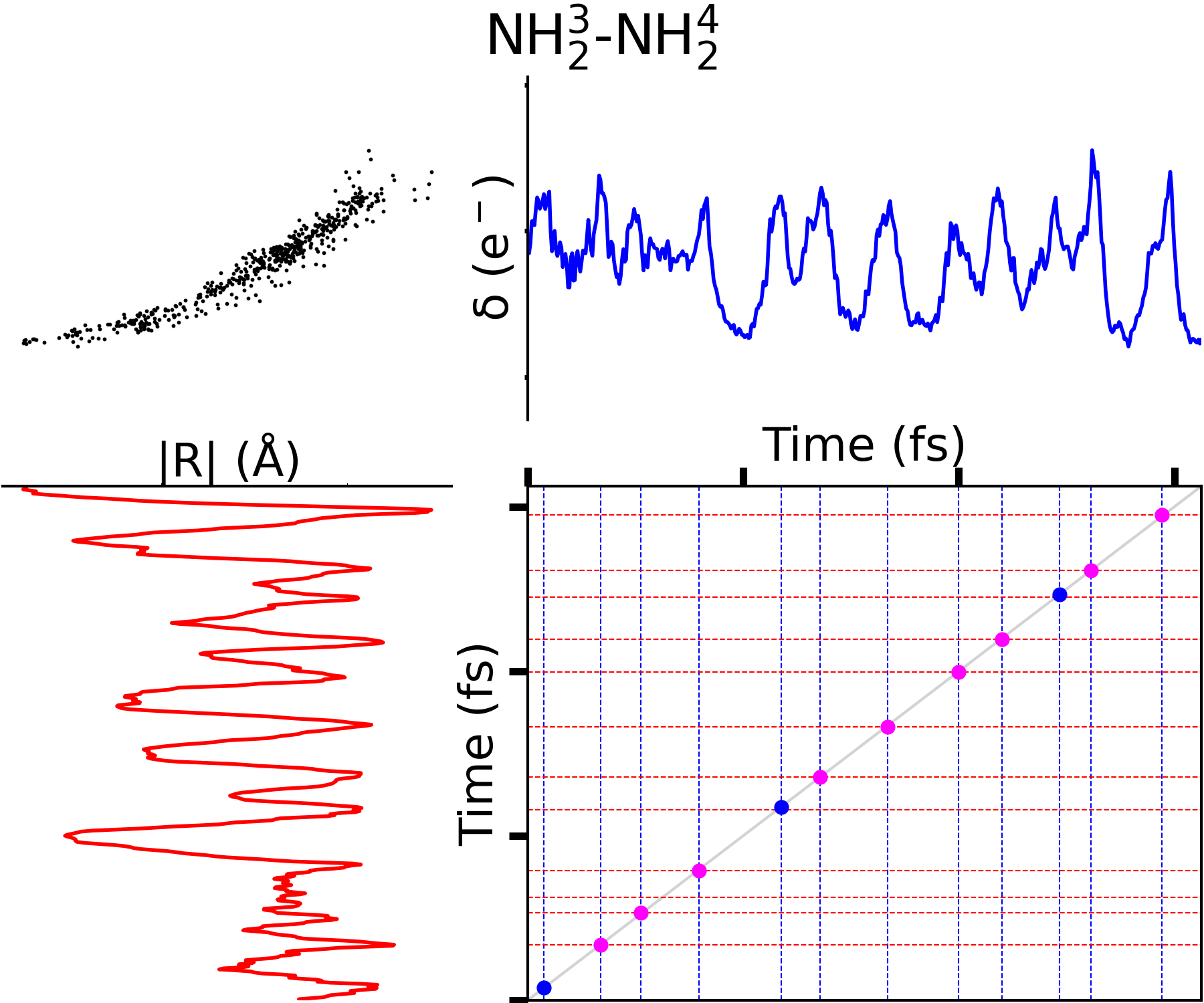

Supplement: Supplementary file 4 — Source Data [file 41467_2024_48567_MOESM4_ESM.zip › main/Fig5/B/NH23_NH24_corrmap_paper.png]

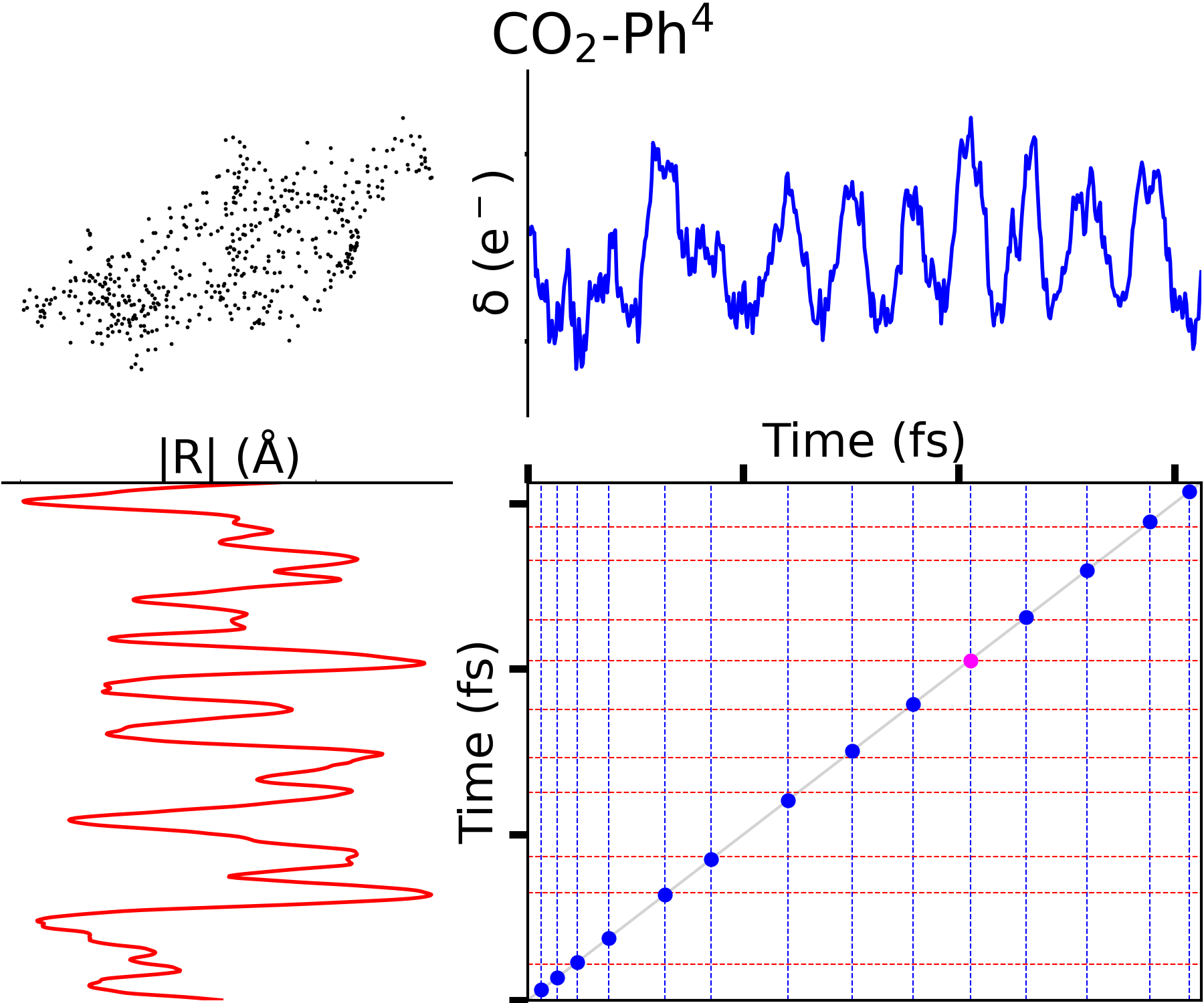

Supplement: Supplementary file 4 — Source Data [file 41467_2024_48567_MOESM4_ESM.zip › main/Fig5/B/CO2_PH4_corrmap_paper.png]

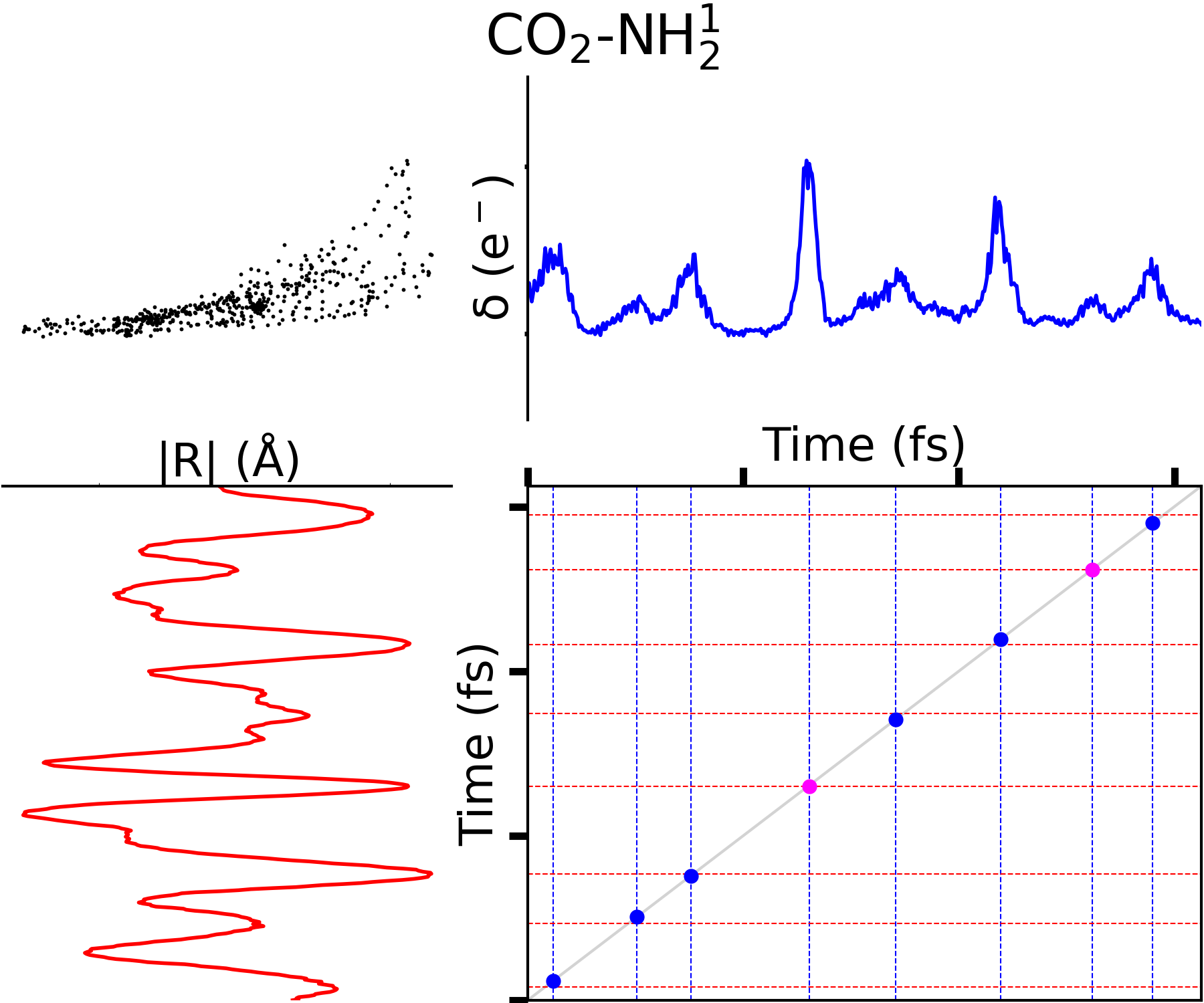

Supplement: Supplementary file 4 — Source Data [file 41467_2024_48567_MOESM4_ESM.zip › main/Fig5/B/CO2_NH21_corrmap_paper.png]

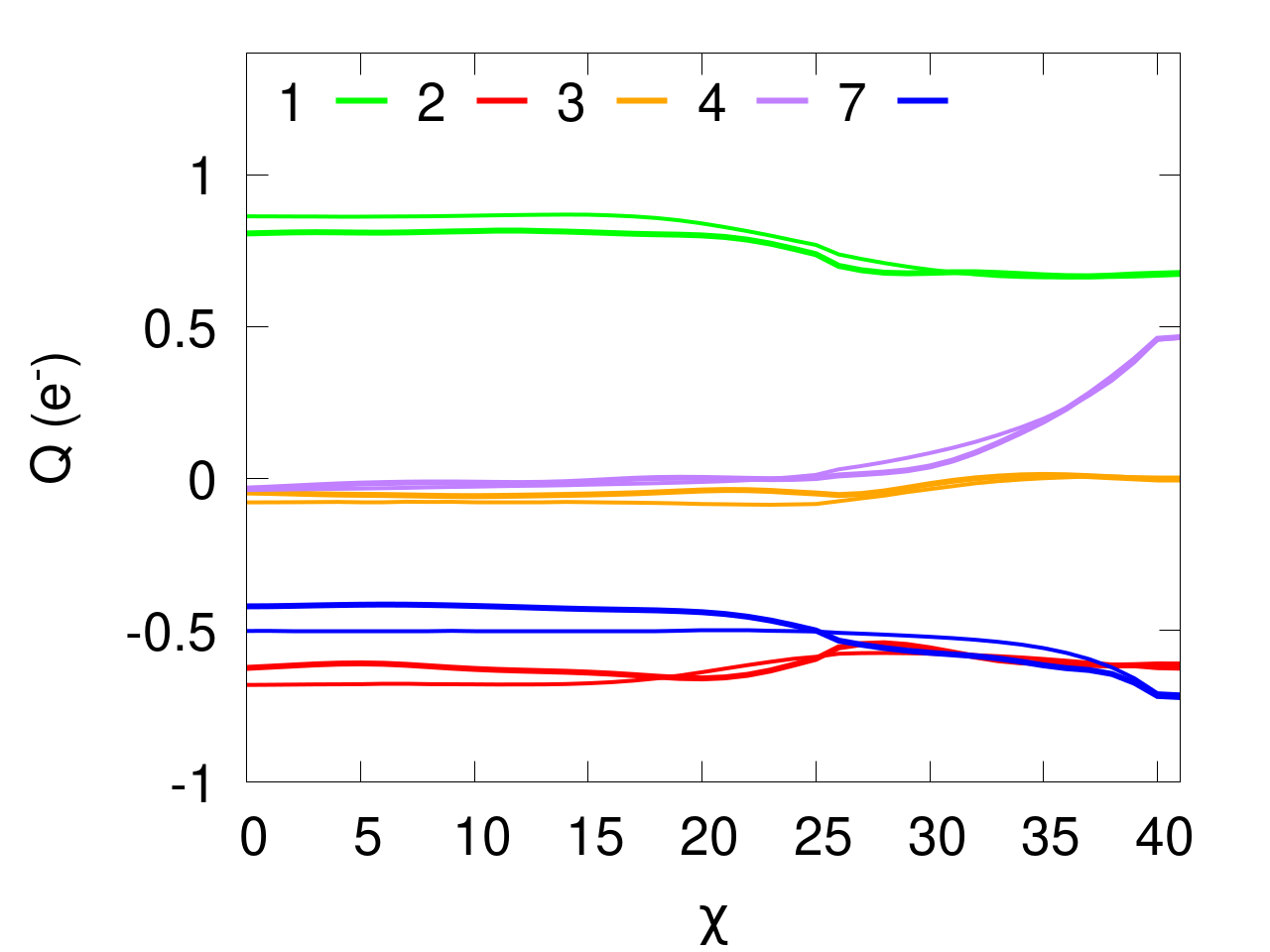

Supplement: Supplementary file 4 — Source Data [file 41467_2024_48567_MOESM4_ESM.zip › main/Fig4/atomic_charge/q_atoms_spk_ampli.png]

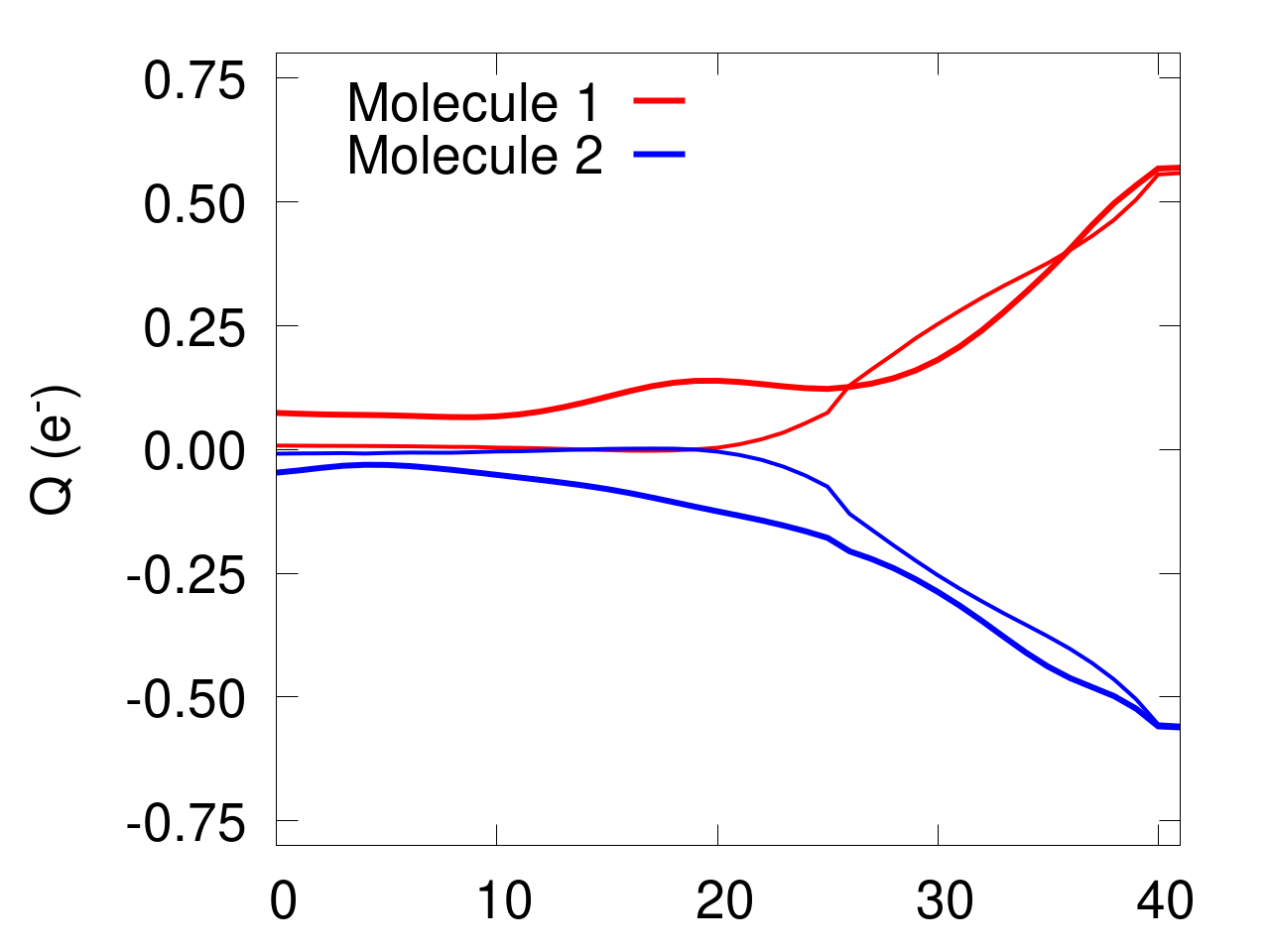

Supplement: Supplementary file 4 — Source Data [file 41467_2024_48567_MOESM4_ESM.zip › main/Fig4/electron_transfer/q_groups_ampli.png]

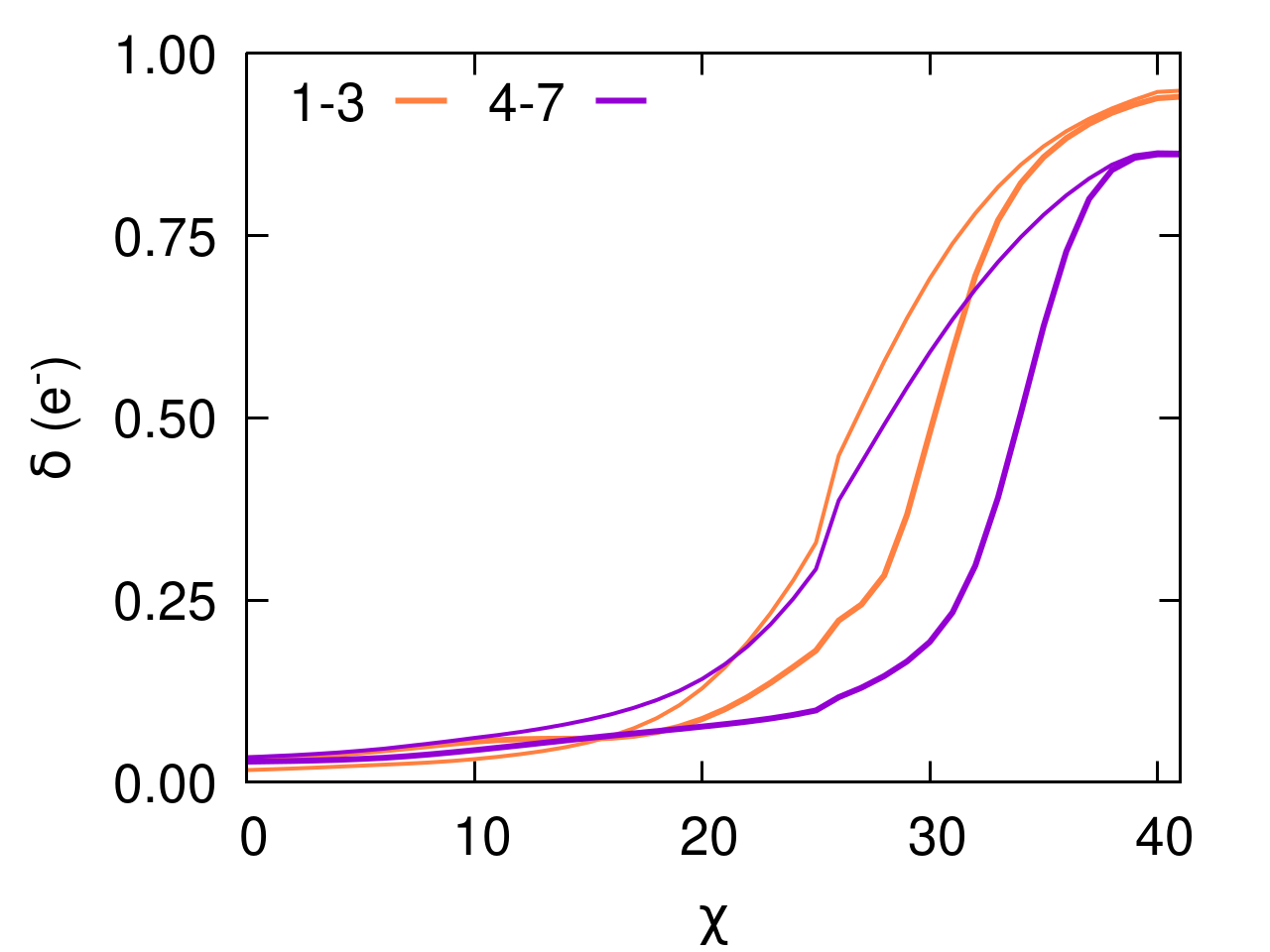

Supplement: Supplementary file 4 — Source Data [file 41467_2024_48567_MOESM4_ESM.zip › main/Fig4/electron_delocalization/di_inter_ampli.png]

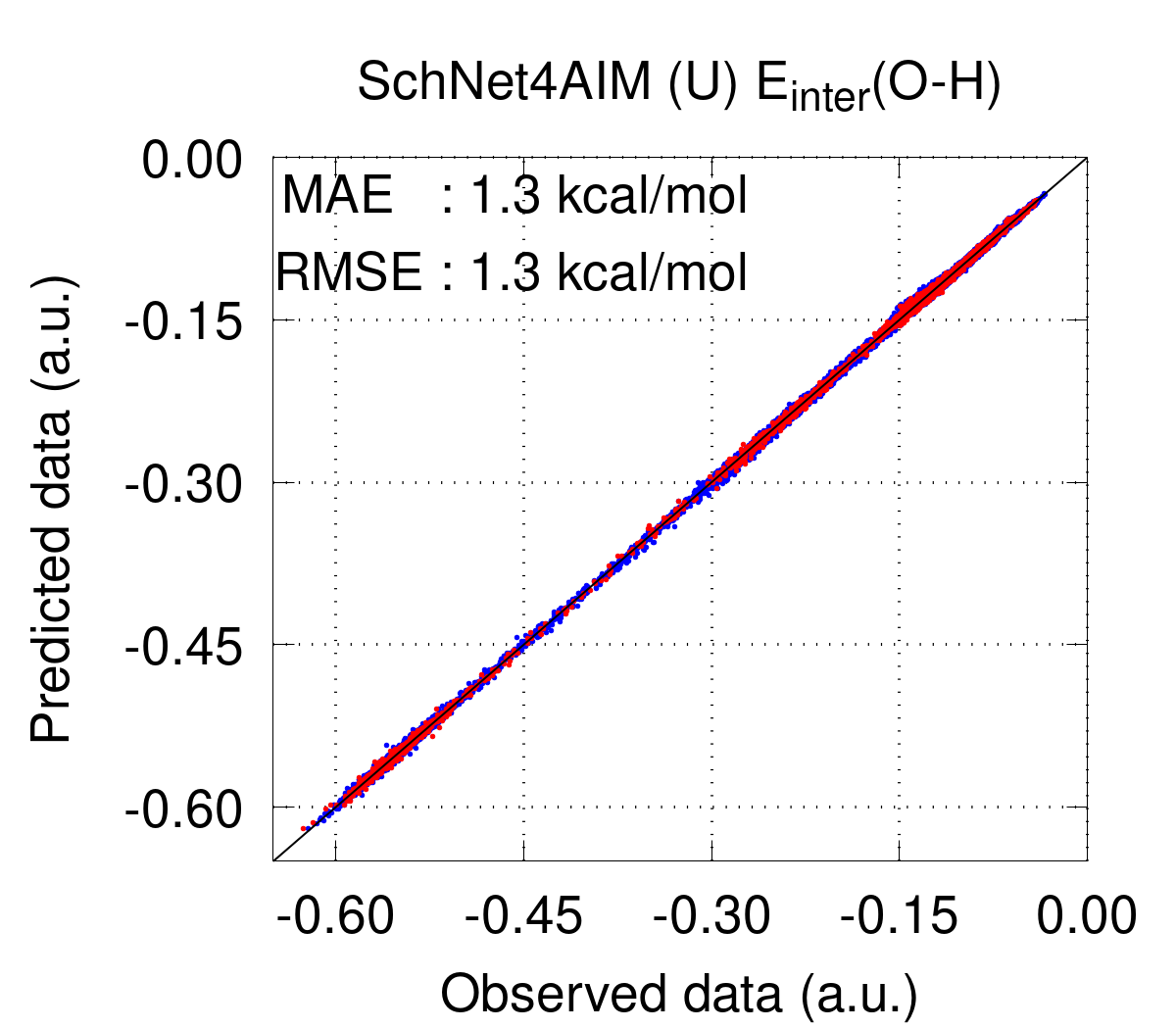

Supplement: Supplementary file 4 — Source Data [file 41467_2024_48567_MOESM4_ESM.zip › main/Fig2/panelC/SPK_aimwise_inter_HO_paper_new.png]

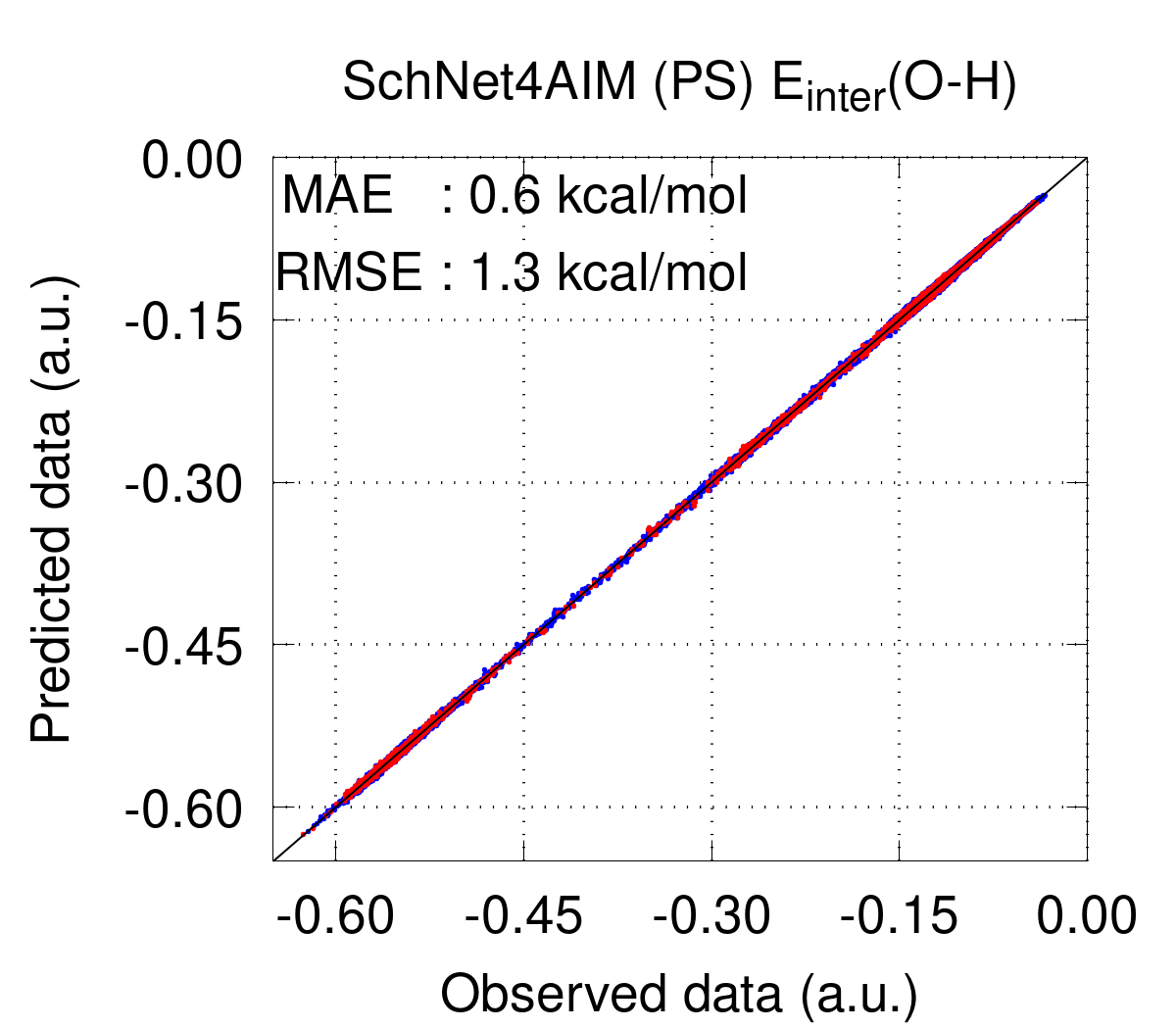

Supplement: Supplementary file 4 — Source Data [file 41467_2024_48567_MOESM4_ESM.zip › main/Fig2/panelD/SPK_elementalaimwise_inter_HO_paper_new.png]

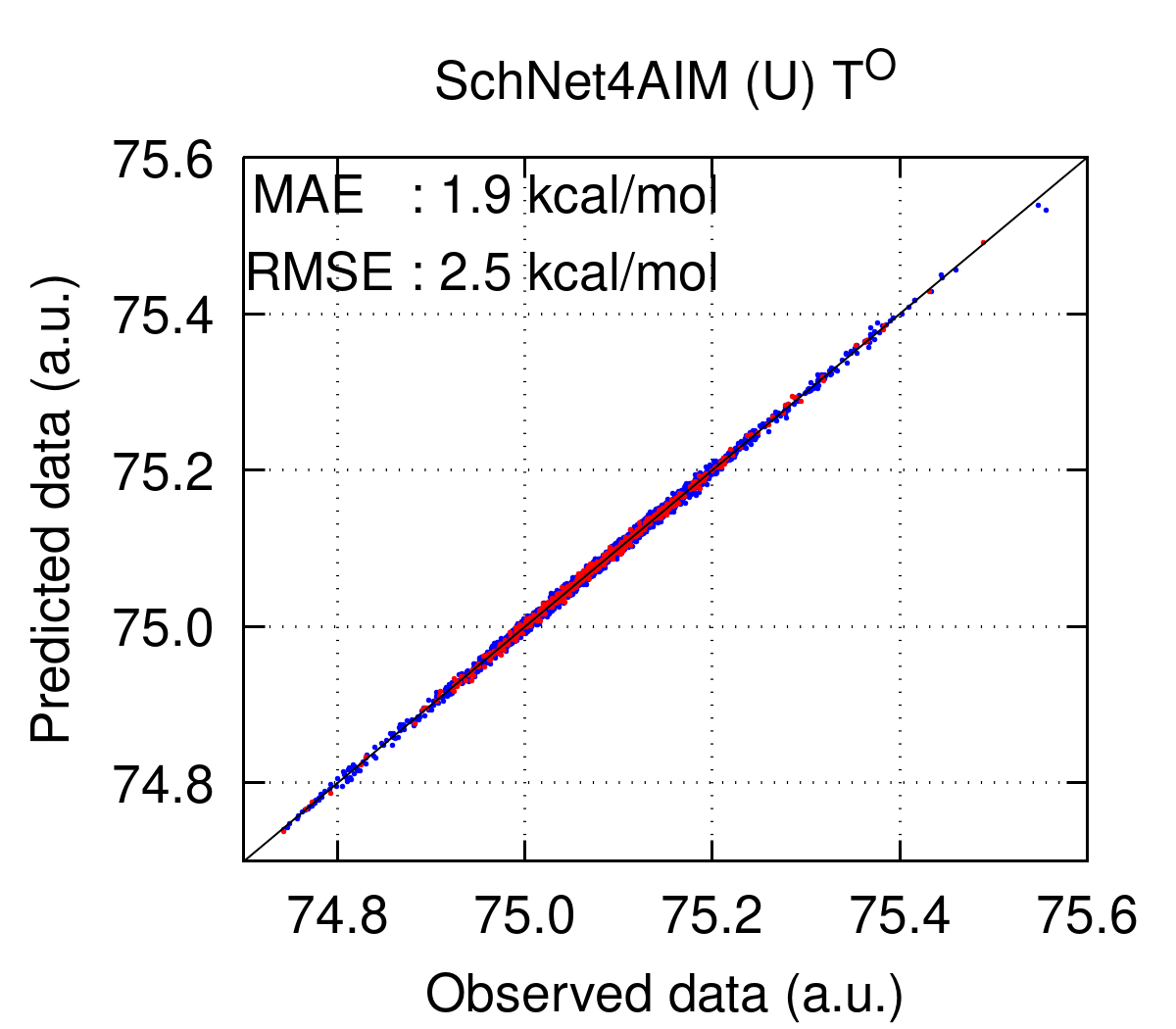

Supplement: Supplementary file 4 — Source Data [file 41467_2024_48567_MOESM4_ESM.zip › main/Fig2/panelA/SPK_aimwise_kin_O_paper_new.png]

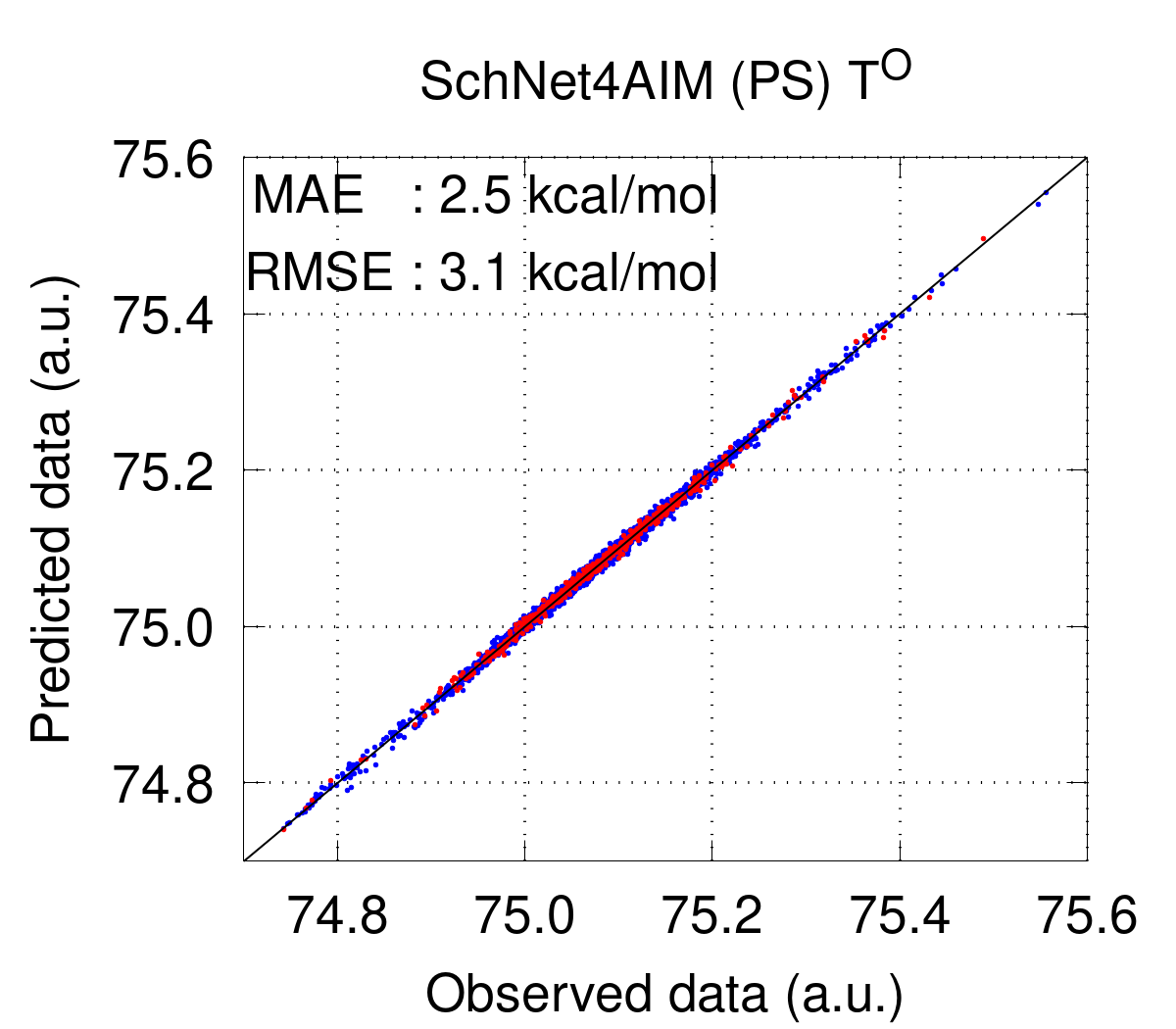

Supplement: Supplementary file 4 — Source Data [file 41467_2024_48567_MOESM4_ESM.zip › main/Fig2/panelB/SPK_elementalaimwise_kin_O_paper_new.png]

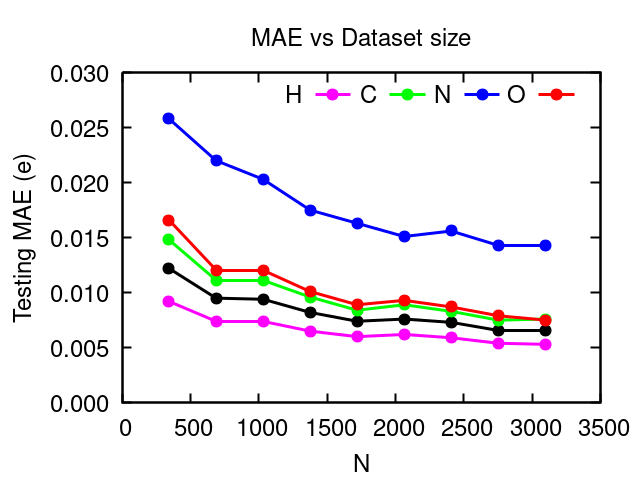

Supplement: Supplementary file 4 — Source Data [file 41467_2024_48567_MOESM4_ESM.zip › SI/Supplementary_Note_19/Supplementary_Figure_53/mae_testing.png]

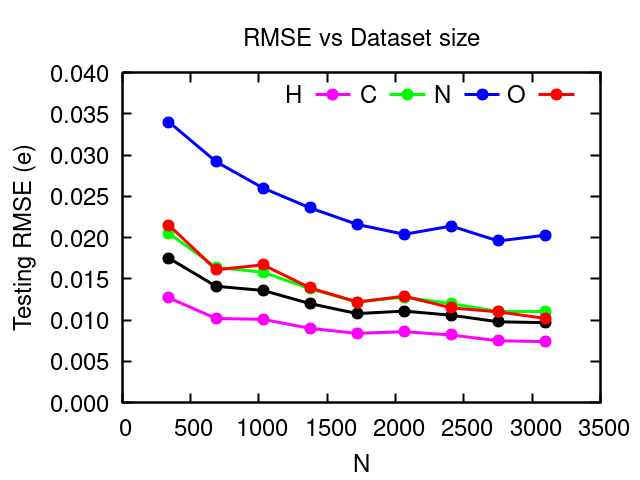

Supplement: Supplementary file 4 — Source Data [file 41467_2024_48567_MOESM4_ESM.zip › SI/Supplementary_Note_19/Supplementary_Figure_53/rmse_testing.png]

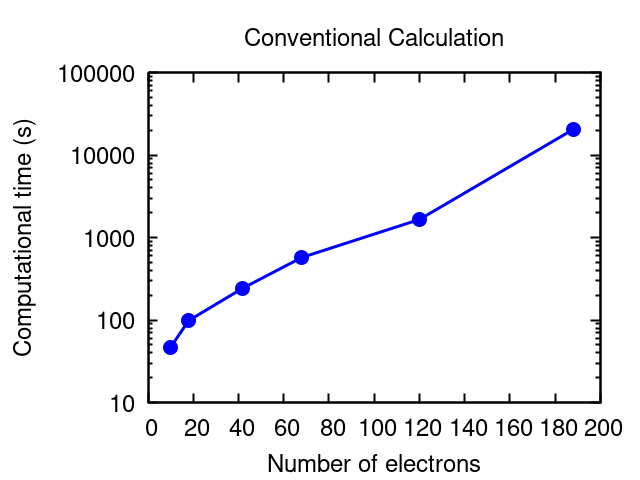

Supplement: Supplementary file 4 — Source Data [file 41467_2024_48567_MOESM4_ESM.zip › SI/Supplementary_Note_17/Supplementary_Figure_45/qm_time_nelec.png]

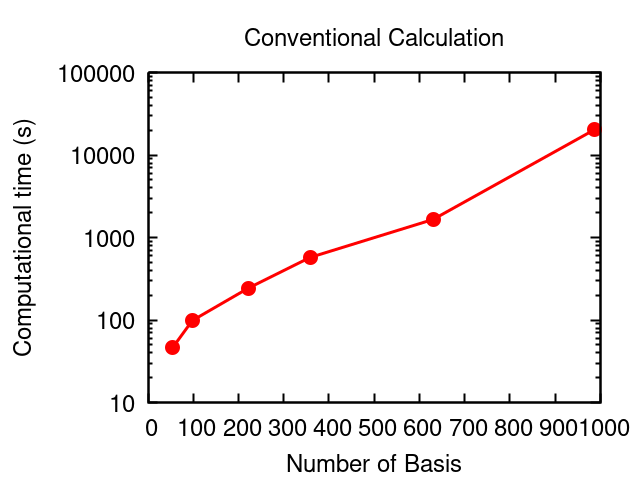

Supplement: Supplementary file 4 — Source Data [file 41467_2024_48567_MOESM4_ESM.zip › SI/Supplementary_Note_17/Supplementary_Figure_45/qm_time_nbasis.png]

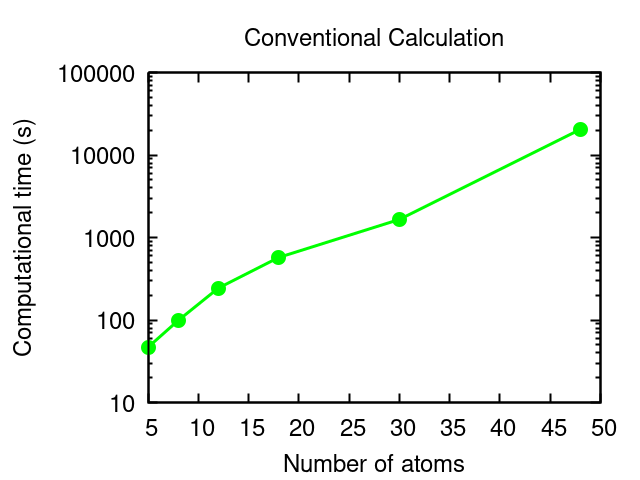

Supplement: Supplementary file 4 — Source Data [file 41467_2024_48567_MOESM4_ESM.zip › SI/Supplementary_Note_17/Supplementary_Figure_45/qm_time_natoms.png]

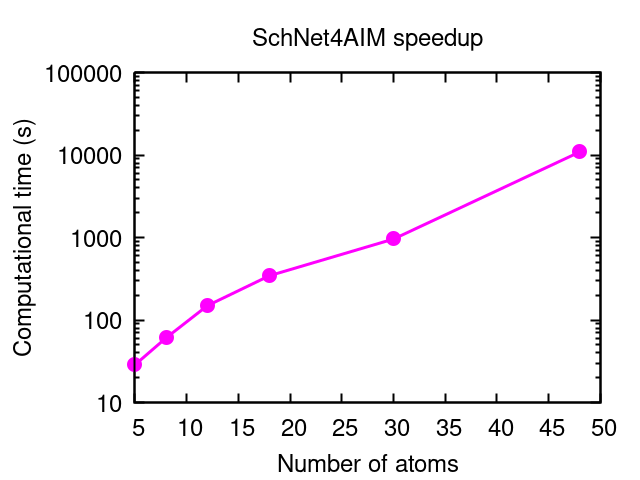

Supplement: Supplementary file 4 — Source Data [file 41467_2024_48567_MOESM4_ESM.zip › SI/Supplementary_Note_17/Supplementary_Figure_46/s4aim_speedup.png]

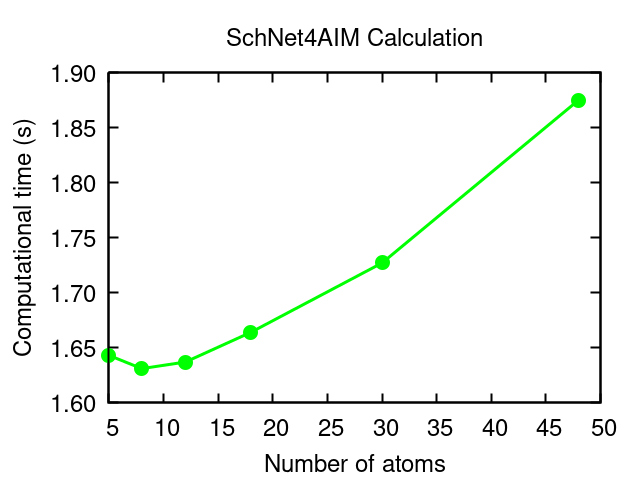

Supplement: Supplementary file 4 — Source Data [file 41467_2024_48567_MOESM4_ESM.zip › SI/Supplementary_Note_17/Supplementary_Figure_46/s4aim_time_natoms.png]

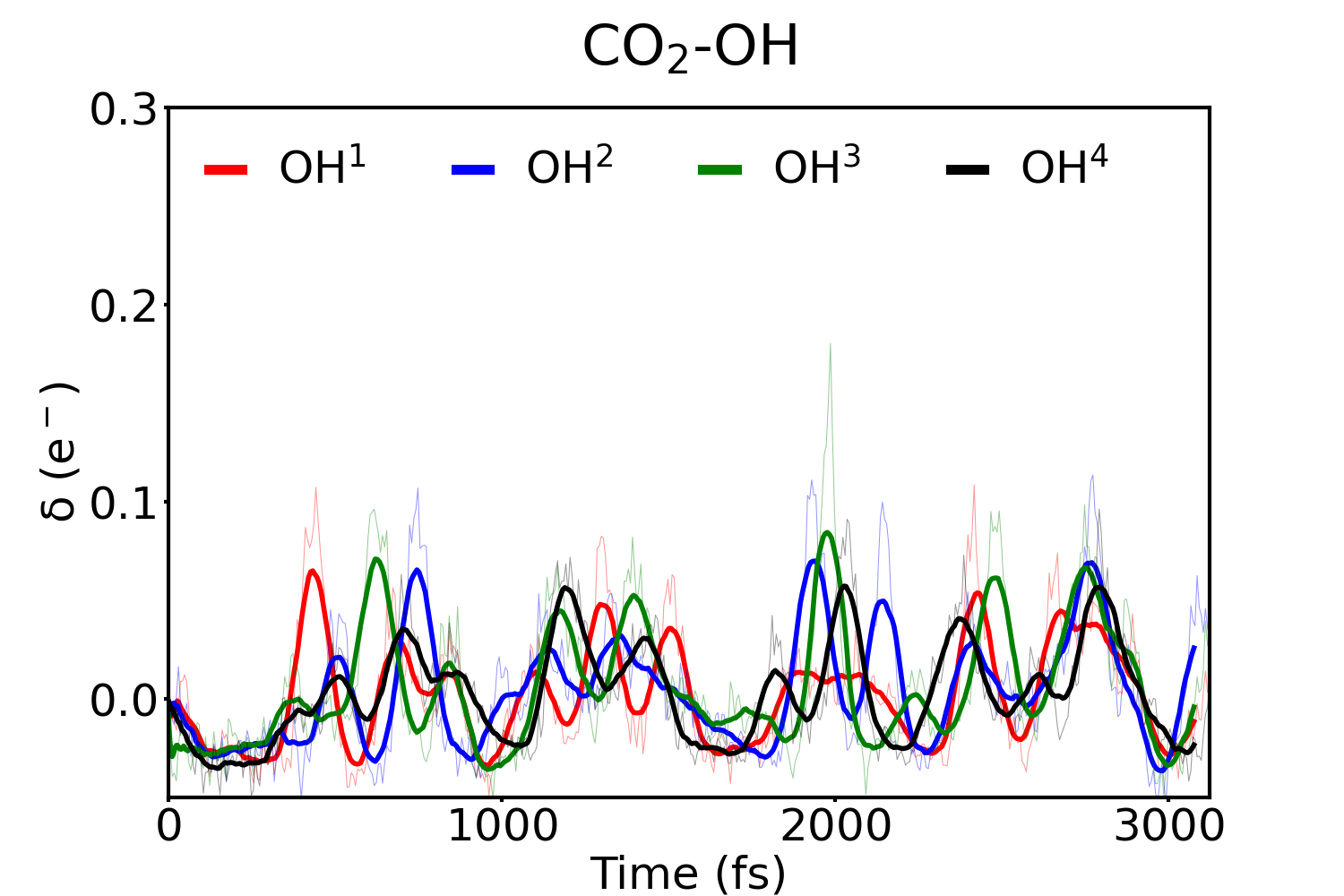

Supplement: Supplementary file 4 — Source Data [file 41467_2024_48567_MOESM4_ESM.zip › SI/Supplementary_Note_14/Supplementary_Figure_19/C/13P_CO2_OH_smooth.png]

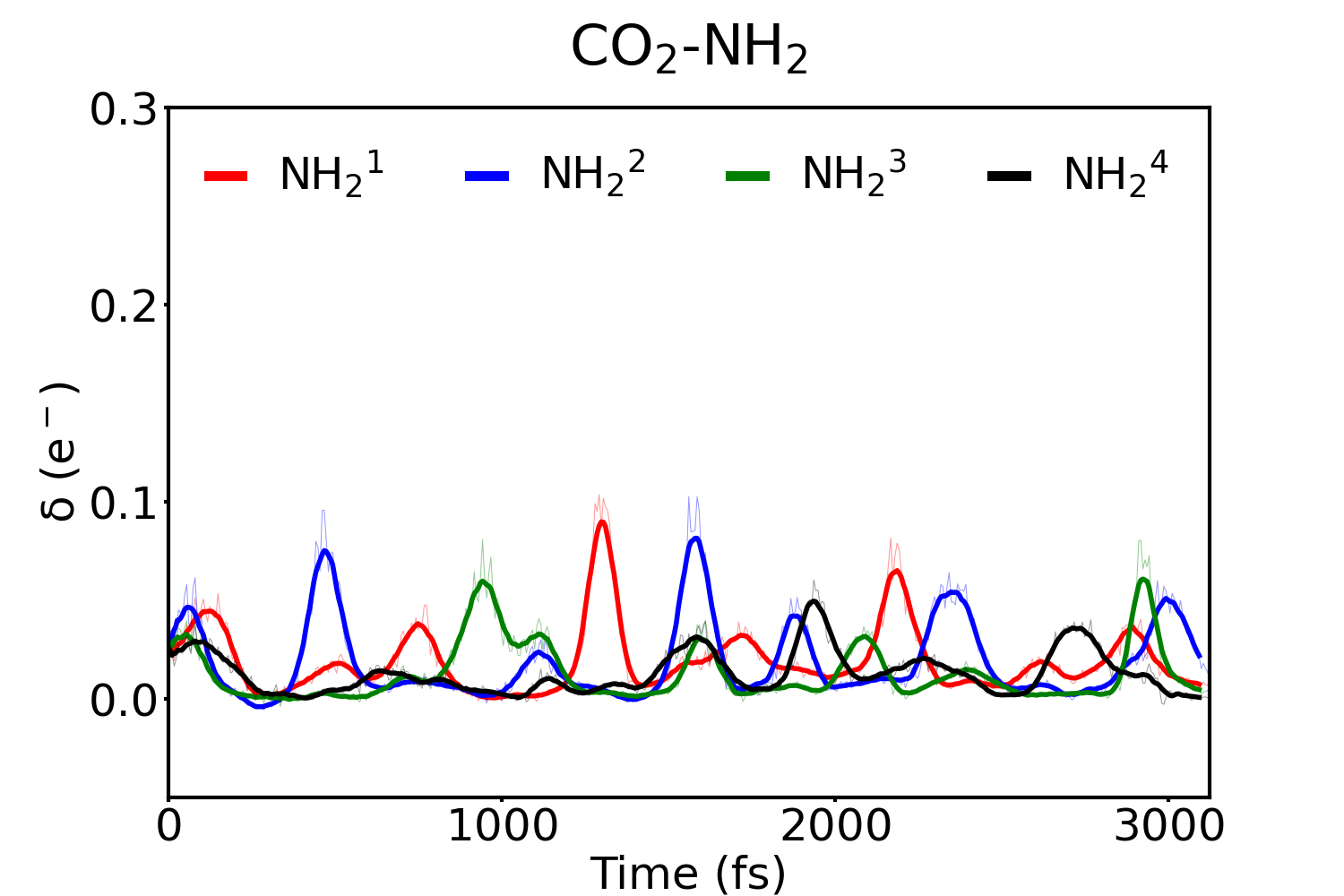

Supplement: Supplementary file 4 — Source Data [file 41467_2024_48567_MOESM4_ESM.zip › SI/Supplementary_Note_14/Supplementary_Figure_19/C/13P_CO2_NH2_smooth.png]

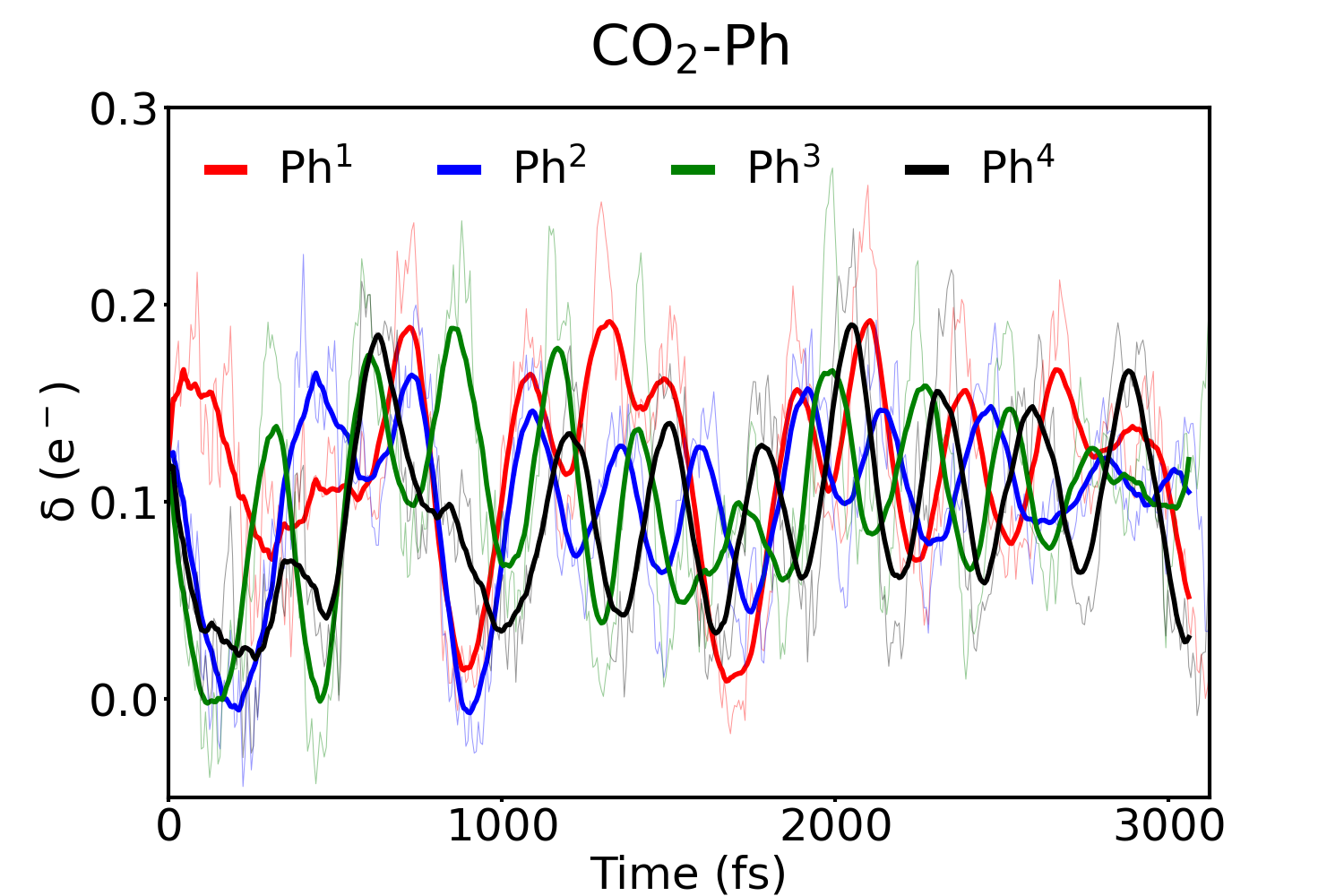

Supplement: Supplementary file 4 — Source Data [file 41467_2024_48567_MOESM4_ESM.zip › SI/Supplementary_Note_14/Supplementary_Figure_19/C/13P_CO2_PH_smooth.png]

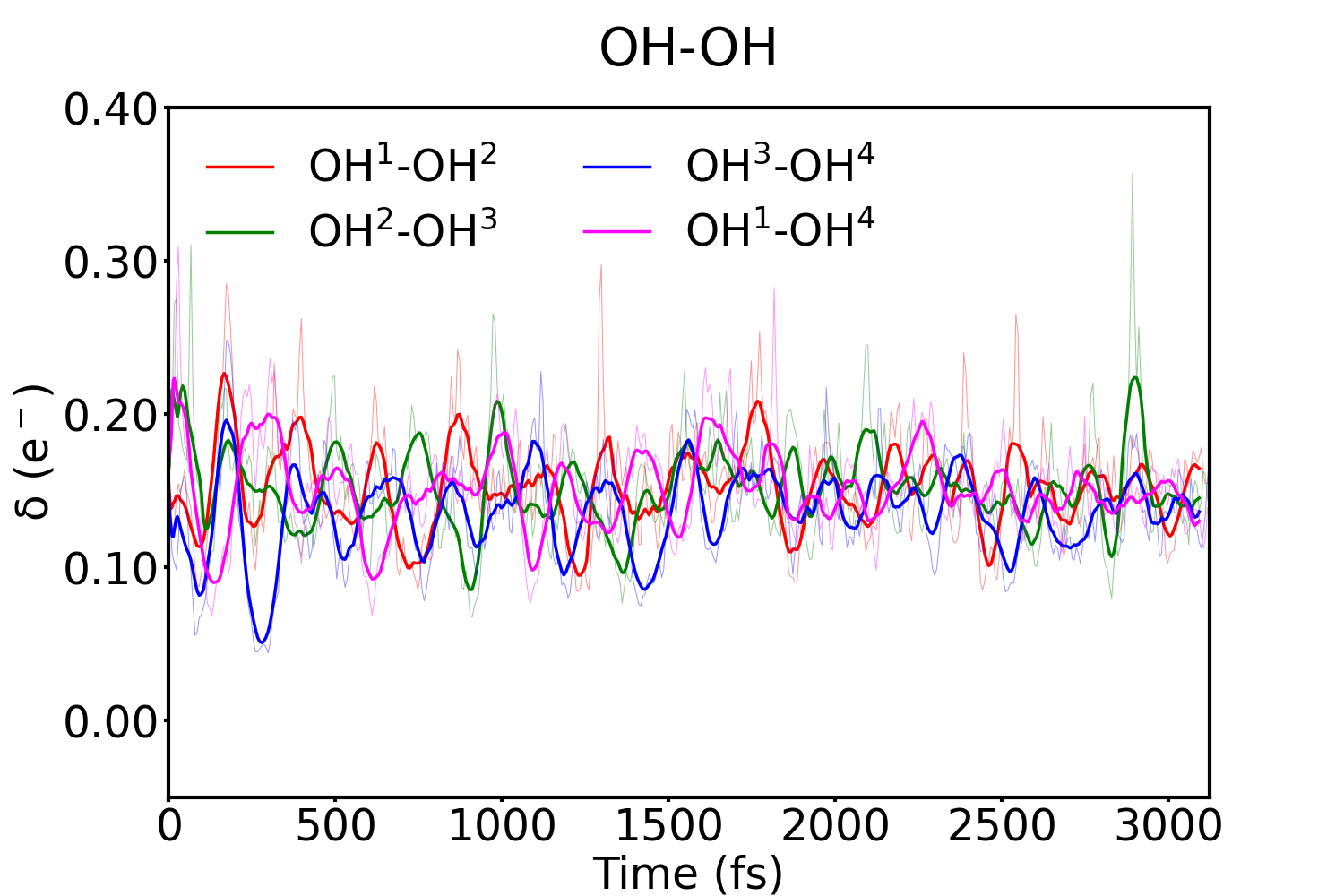

Supplement: Supplementary file 4 — Source Data [file 41467_2024_48567_MOESM4_ESM.zip › SI/Supplementary_Note_14/Supplementary_Figure_19/B/13P_OH_smooth.png]

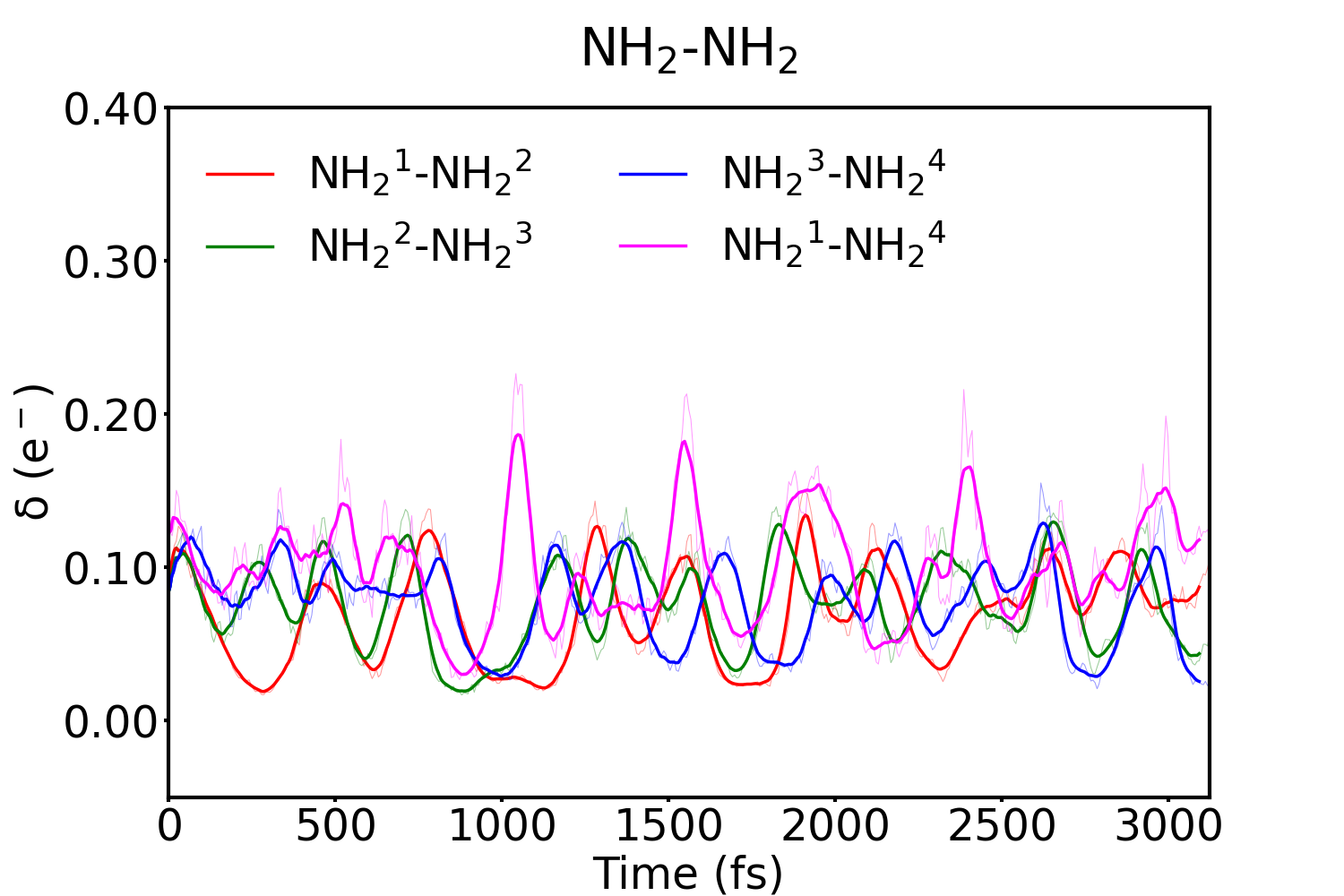

Supplement: Supplementary file 4 — Source Data [file 41467_2024_48567_MOESM4_ESM.zip › SI/Supplementary_Note_14/Supplementary_Figure_19/B/13P_NH2_smooth.png]

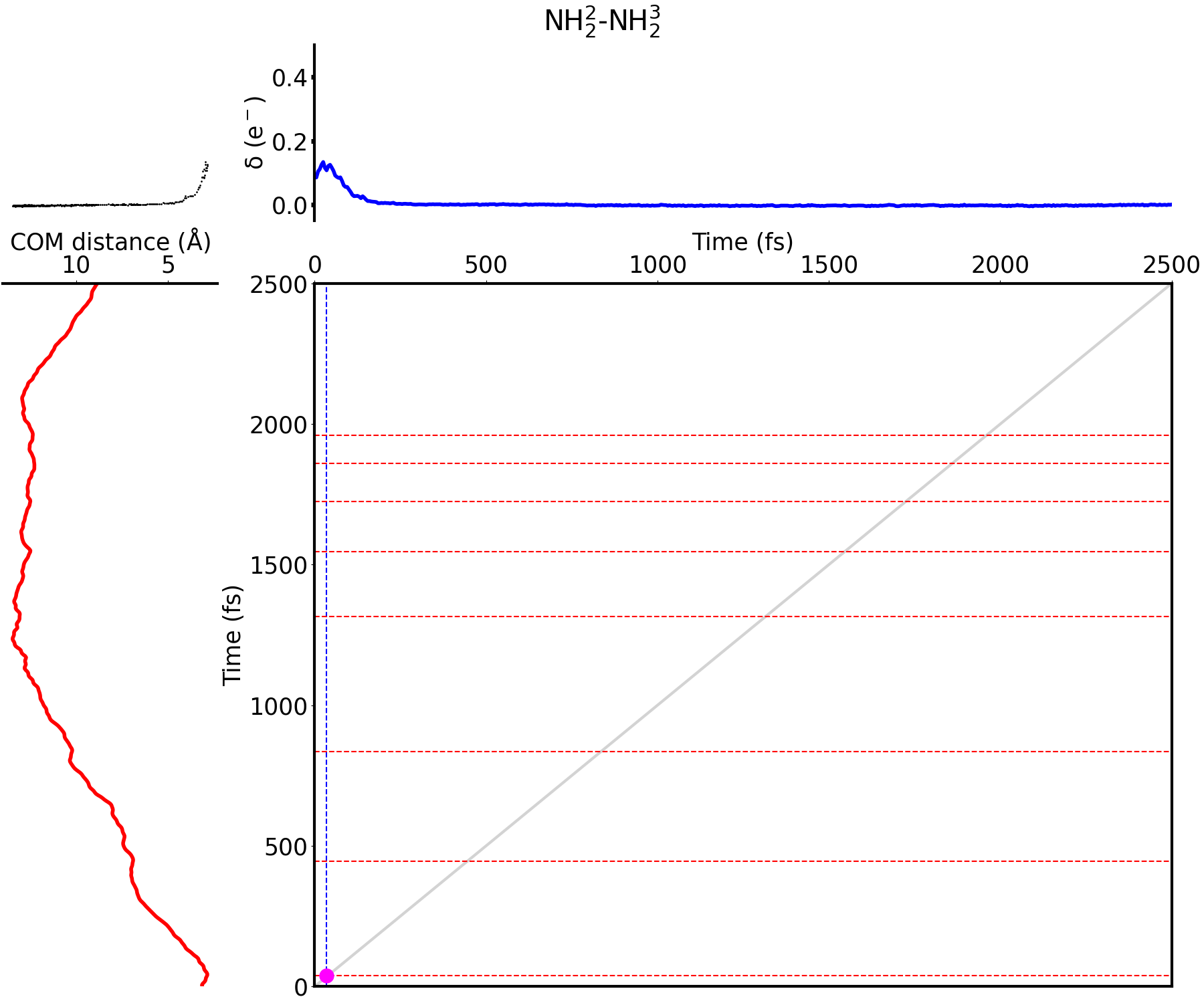

Supplement: Supplementary file 4 — Source Data [file 41467_2024_48567_MOESM4_ESM.zip › SI/Supplementary_Note_14/Supplementary_Figure_37/HT_NH22_NH23_corrmap.png]

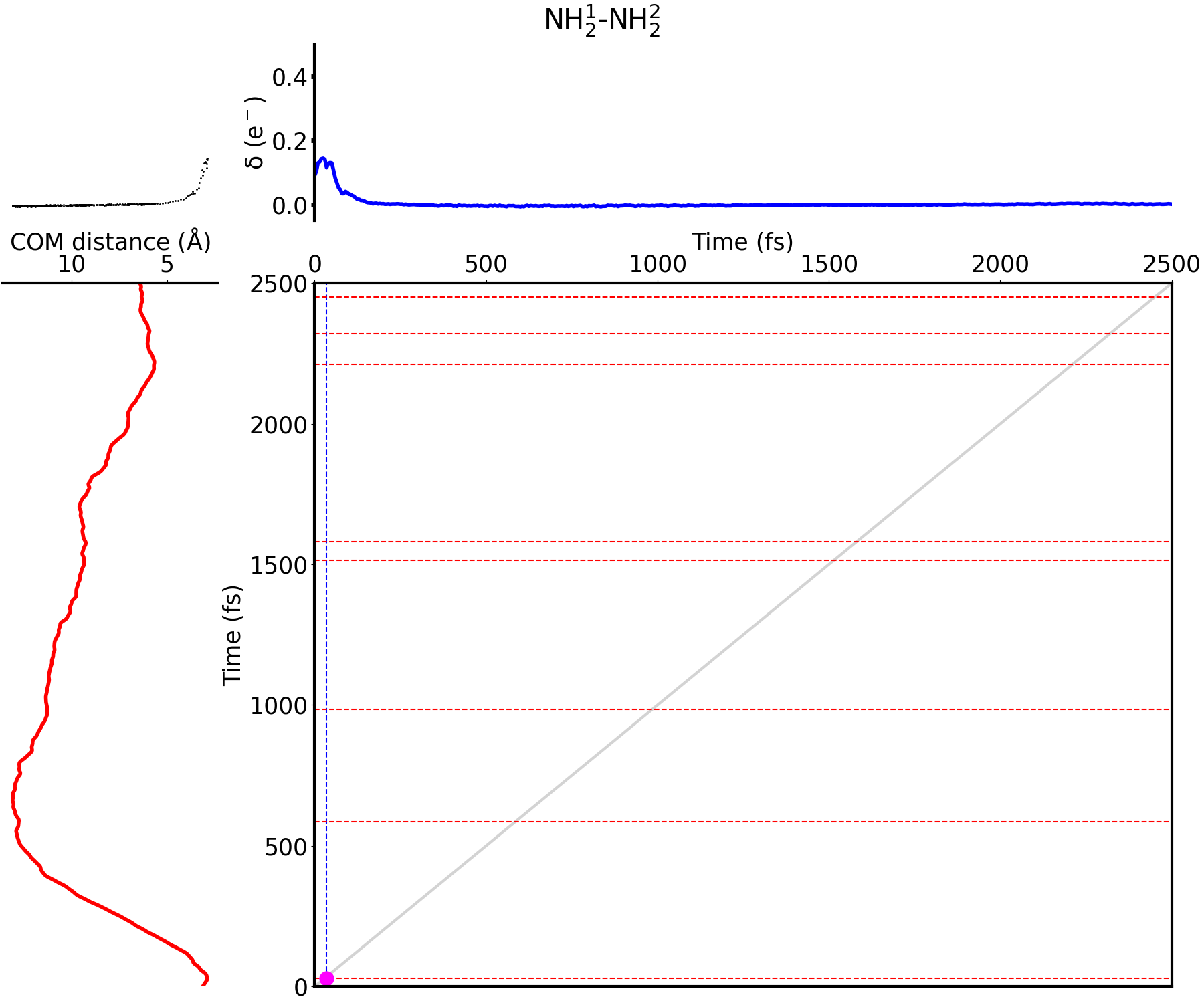

Supplement: Supplementary file 4 — Source Data [file 41467_2024_48567_MOESM4_ESM.zip › SI/Supplementary_Note_14/Supplementary_Figure_37/HT_NH21_NH22_corrmap.png]

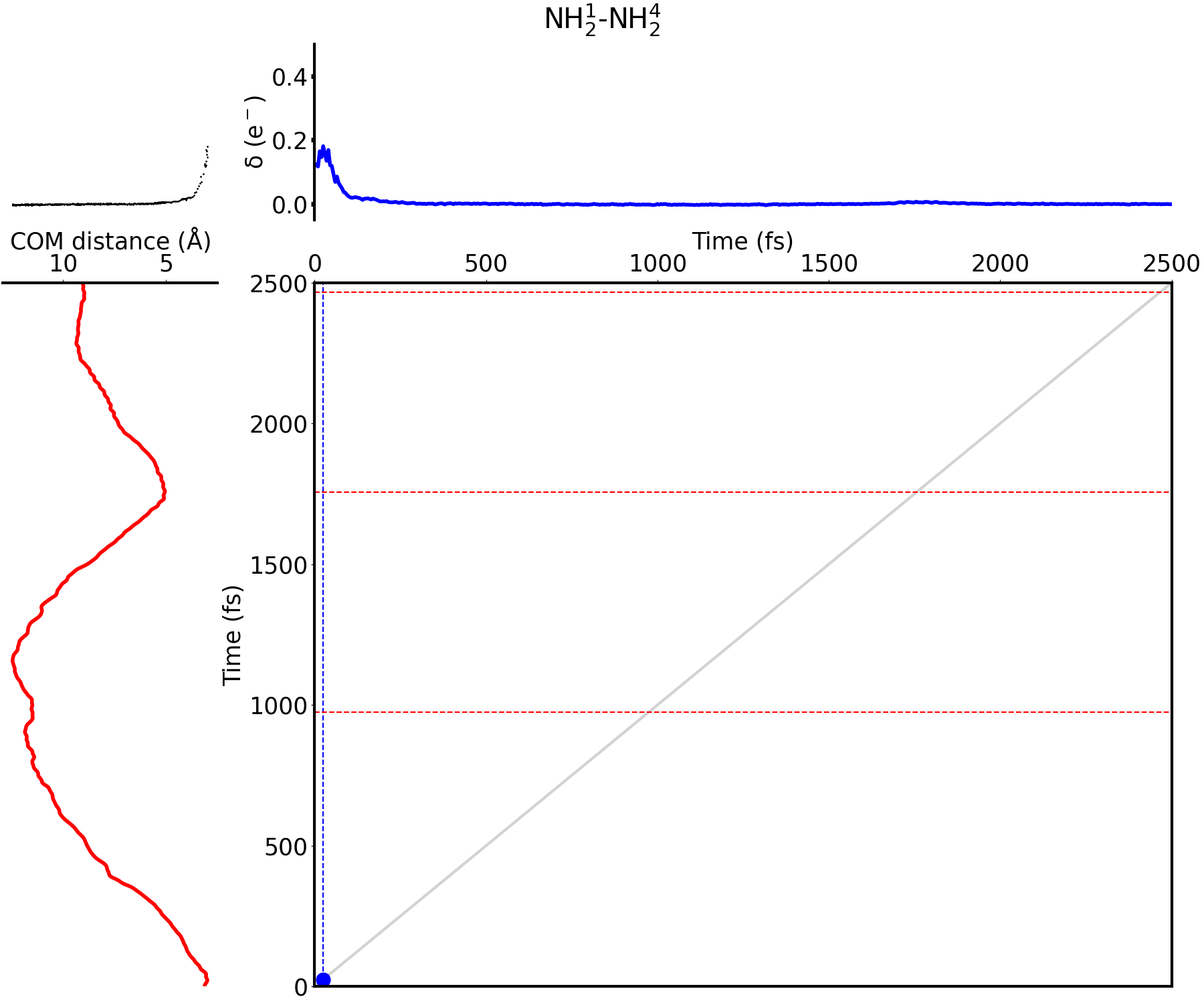

Supplement: Supplementary file 4 — Source Data [file 41467_2024_48567_MOESM4_ESM.zip › SI/Supplementary_Note_14/Supplementary_Figure_37/HT_NH21_NH24_corrmap.png]

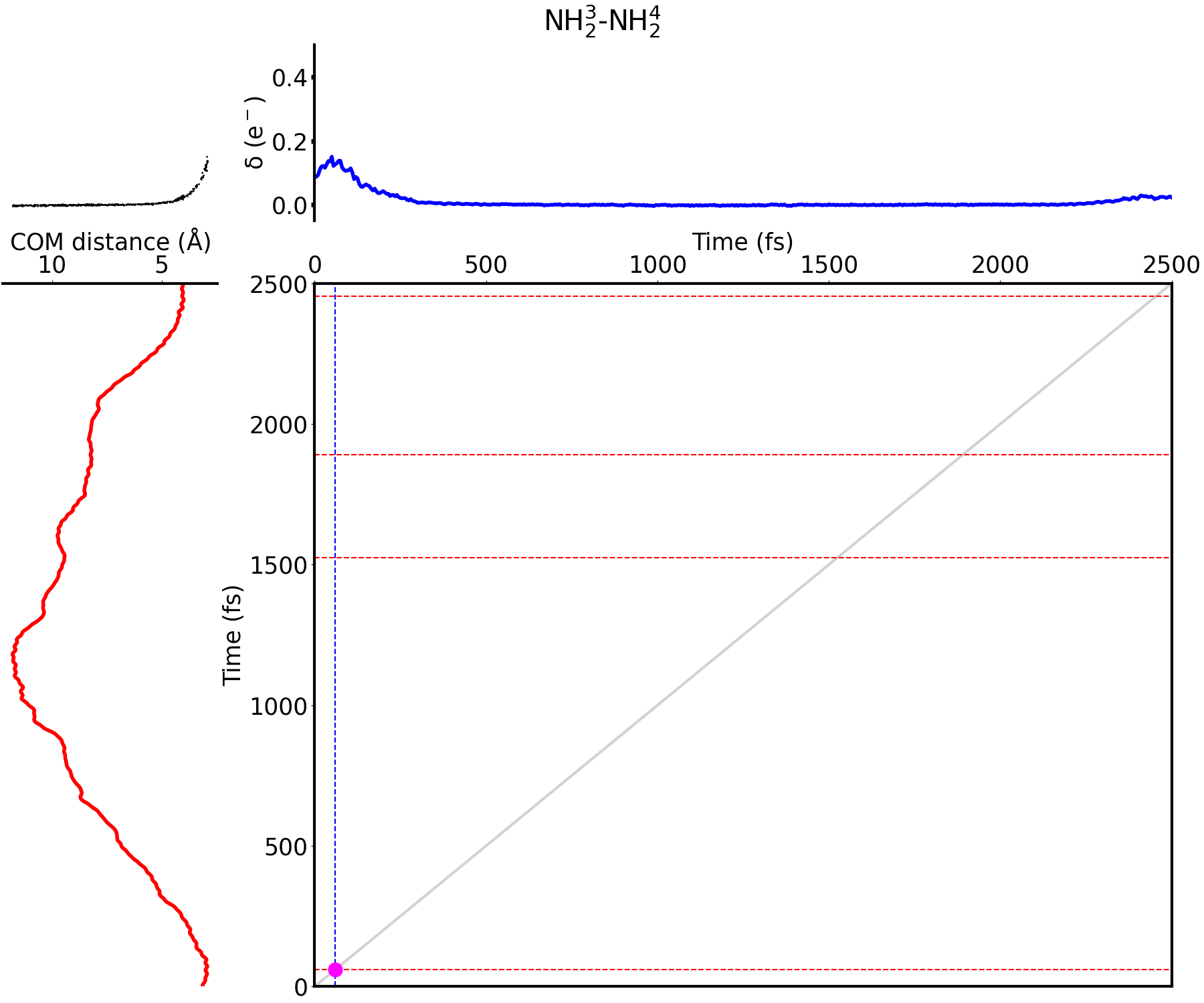

Supplement: Supplementary file 4 — Source Data [file 41467_2024_48567_MOESM4_ESM.zip › SI/Supplementary_Note_14/Supplementary_Figure_37/HT_NH23_NH24_corrmap.png]

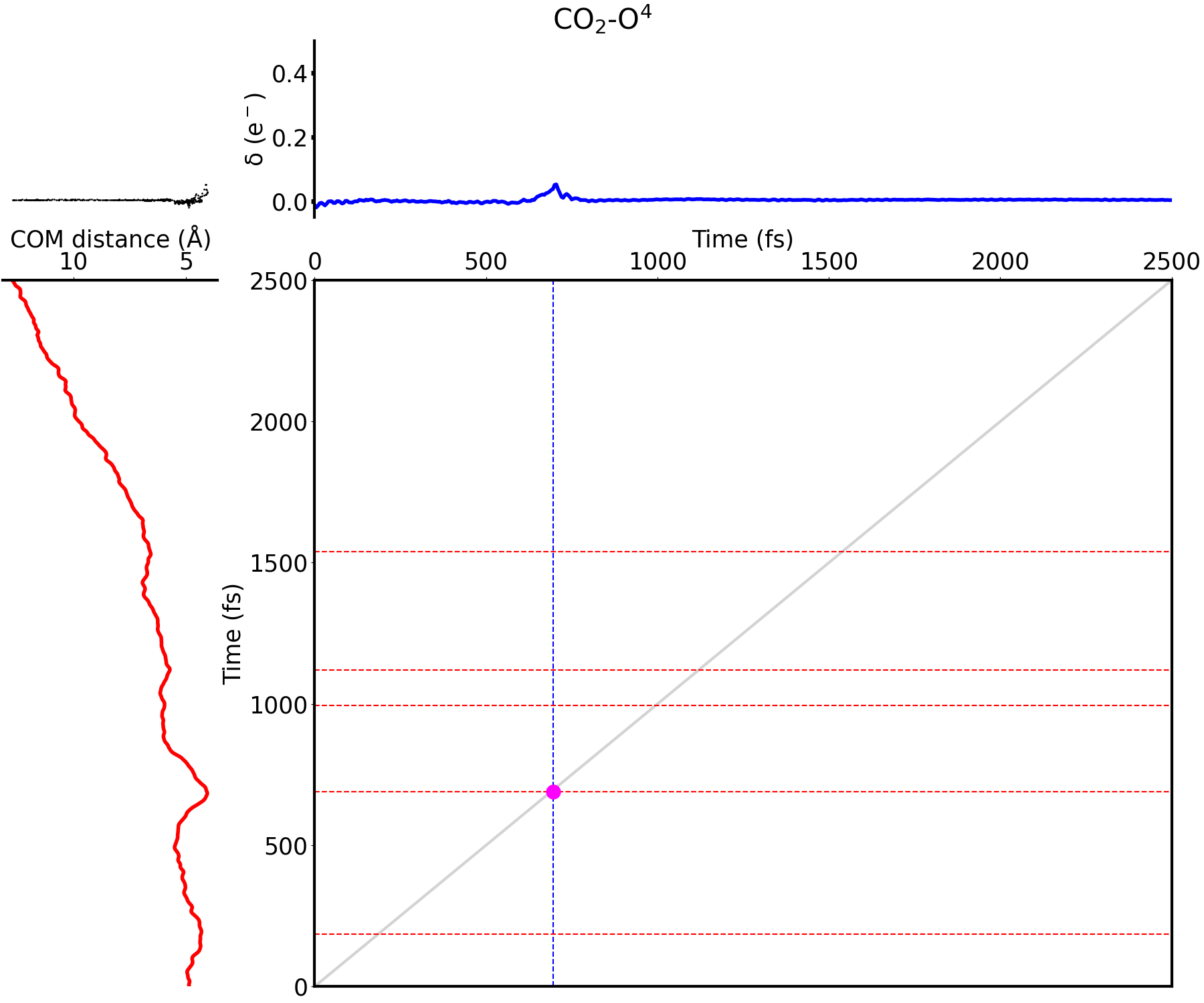

Supplement: Supplementary file 4 — Source Data [file 41467_2024_48567_MOESM4_ESM.zip › SI/Supplementary_Note_14/Supplementary_Figure_35/HT_CO2_O4_corrmap.png]

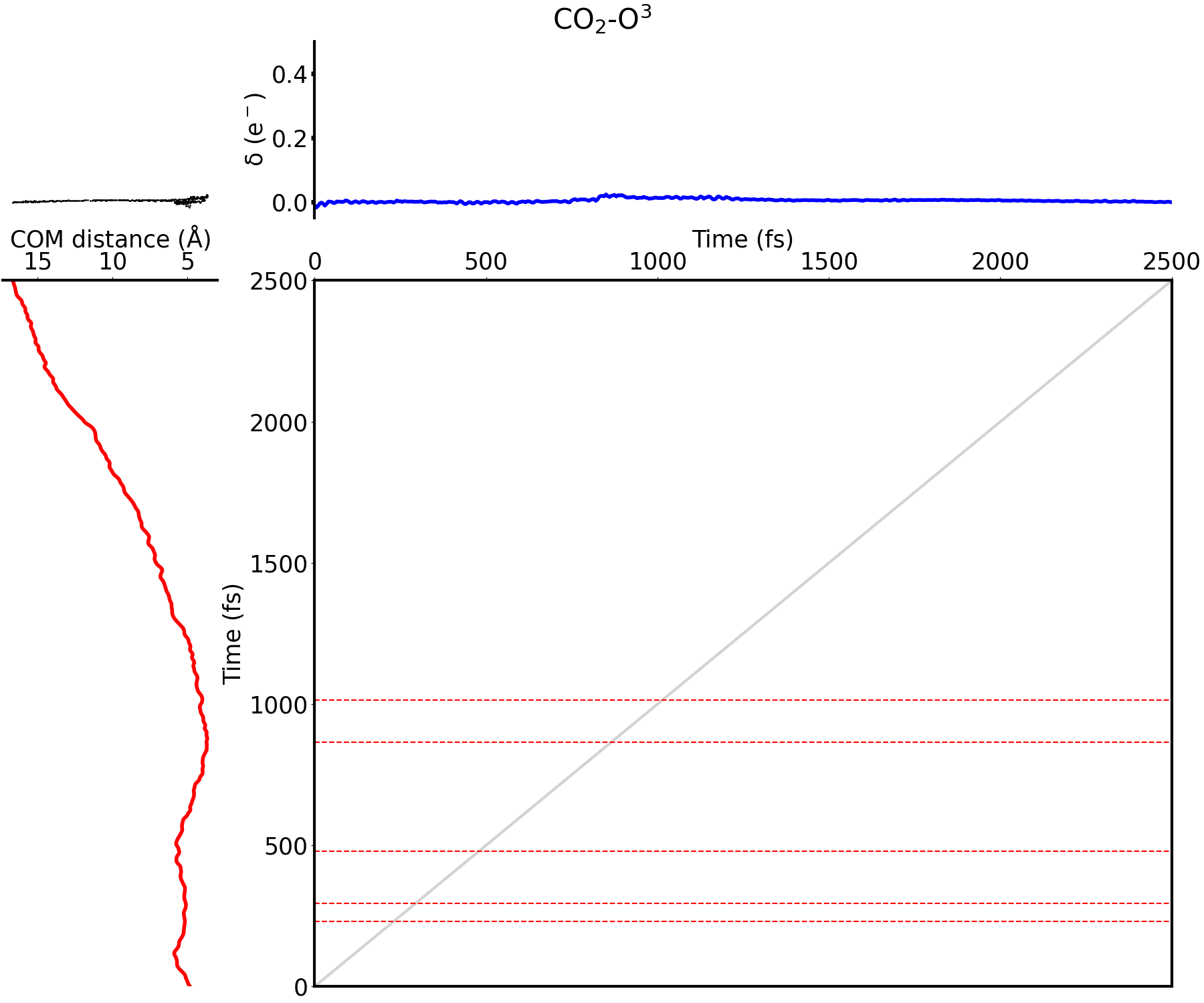

Supplement: Supplementary file 4 — Source Data [file 41467_2024_48567_MOESM4_ESM.zip › SI/Supplementary_Note_14/Supplementary_Figure_35/HT_CO2_O3_corrmap.png]

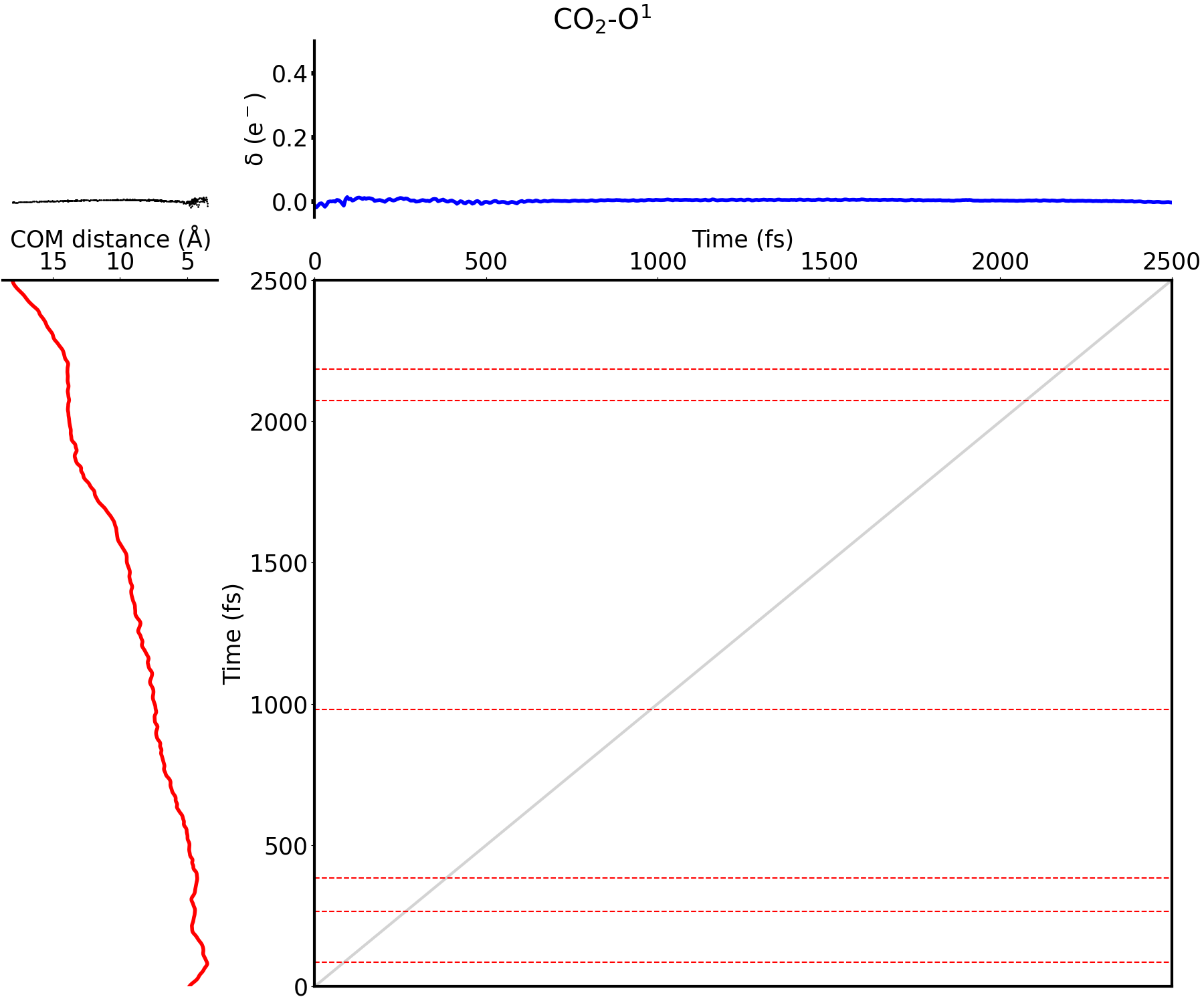

Supplement: Supplementary file 4 — Source Data [file 41467_2024_48567_MOESM4_ESM.zip › SI/Supplementary_Note_14/Supplementary_Figure_35/HT_CO2_O1_corrmap.png]

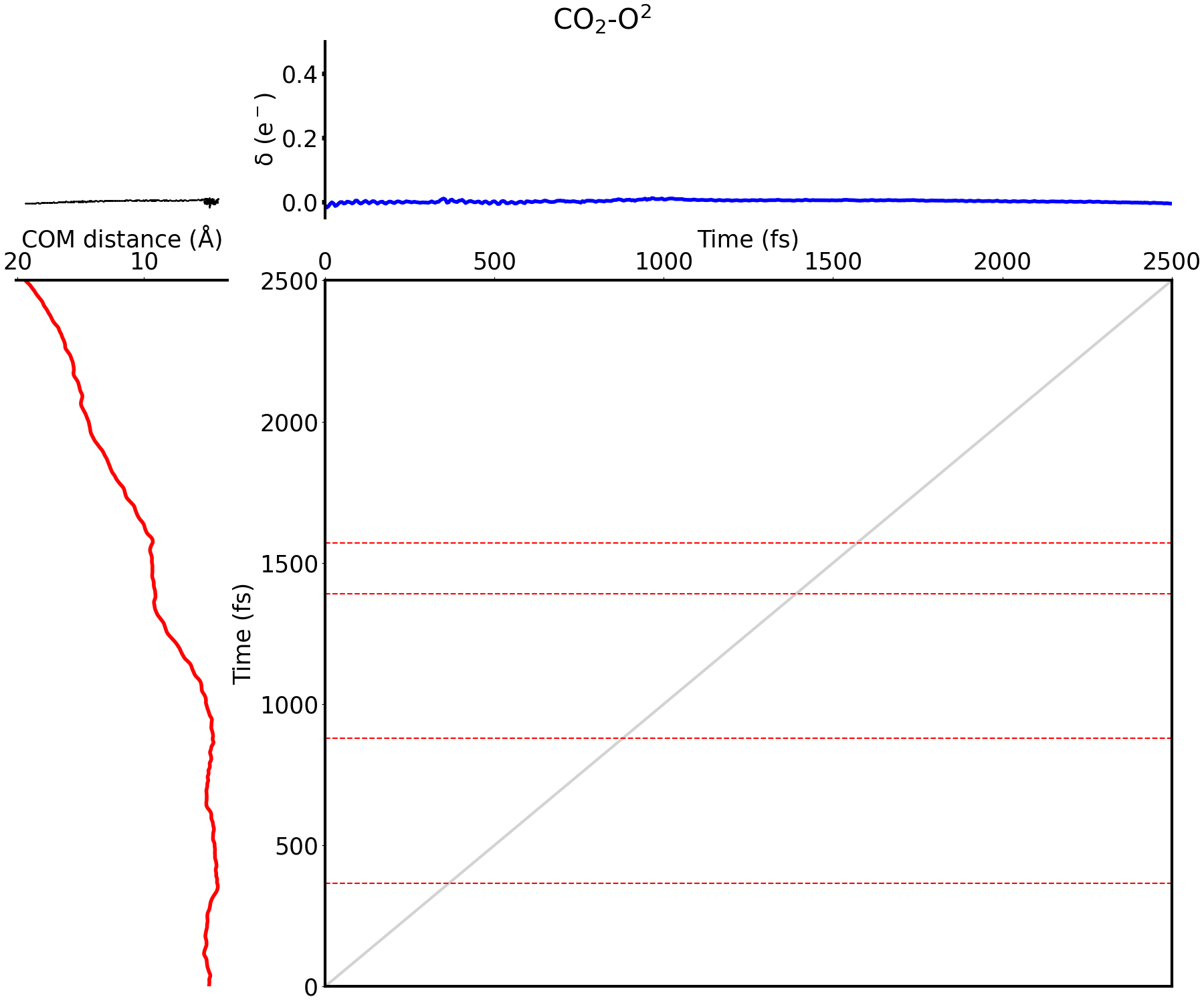

Supplement: Supplementary file 4 — Source Data [file 41467_2024_48567_MOESM4_ESM.zip › SI/Supplementary_Note_14/Supplementary_Figure_35/HT_CO2_O2_corrmap.png]

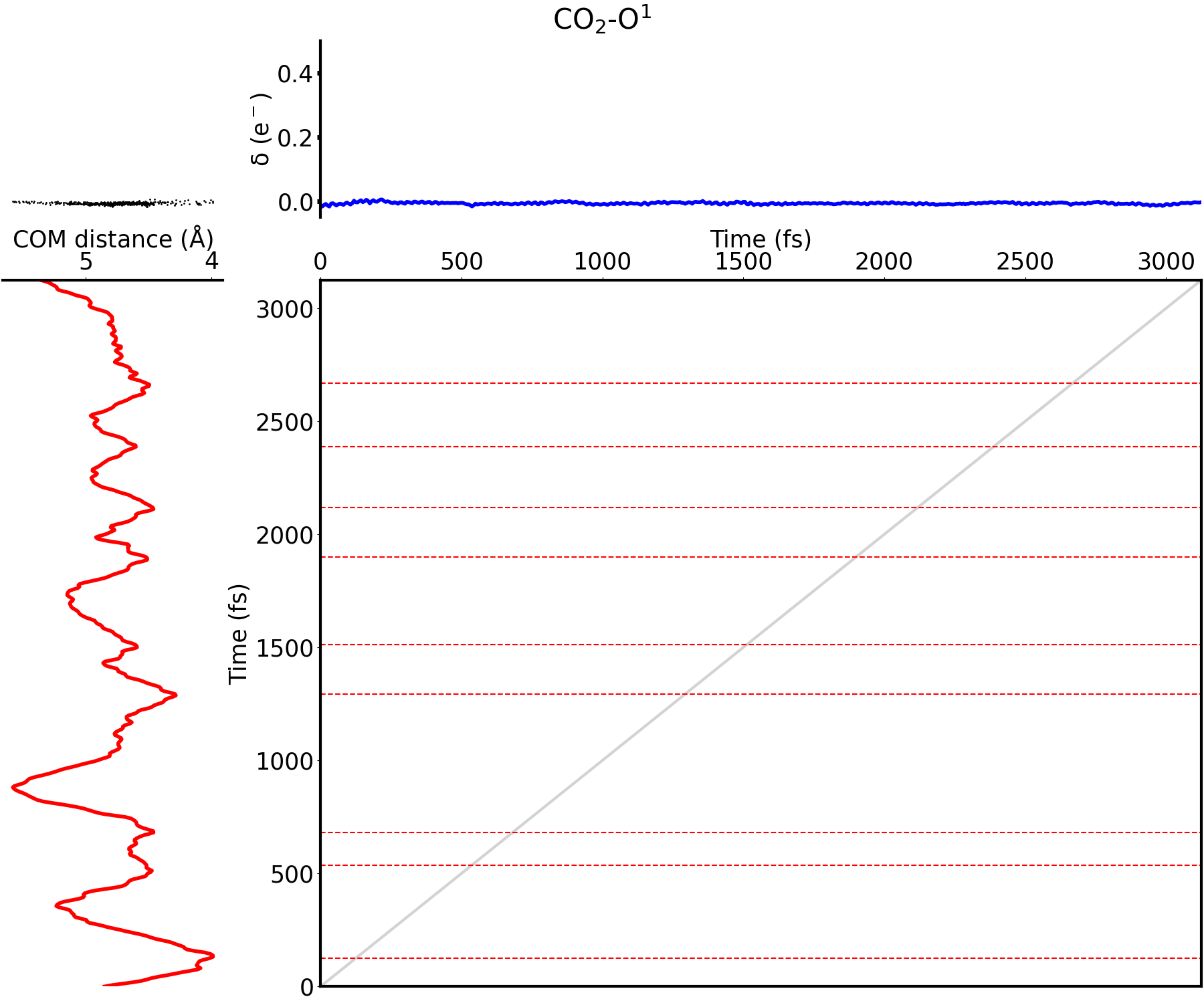

Supplement: Supplementary file 4 — Source Data [file 41467_2024_48567_MOESM4_ESM.zip › SI/Supplementary_Note_14/Supplementary_Figure_27/CO2_O1_corrmap.png]

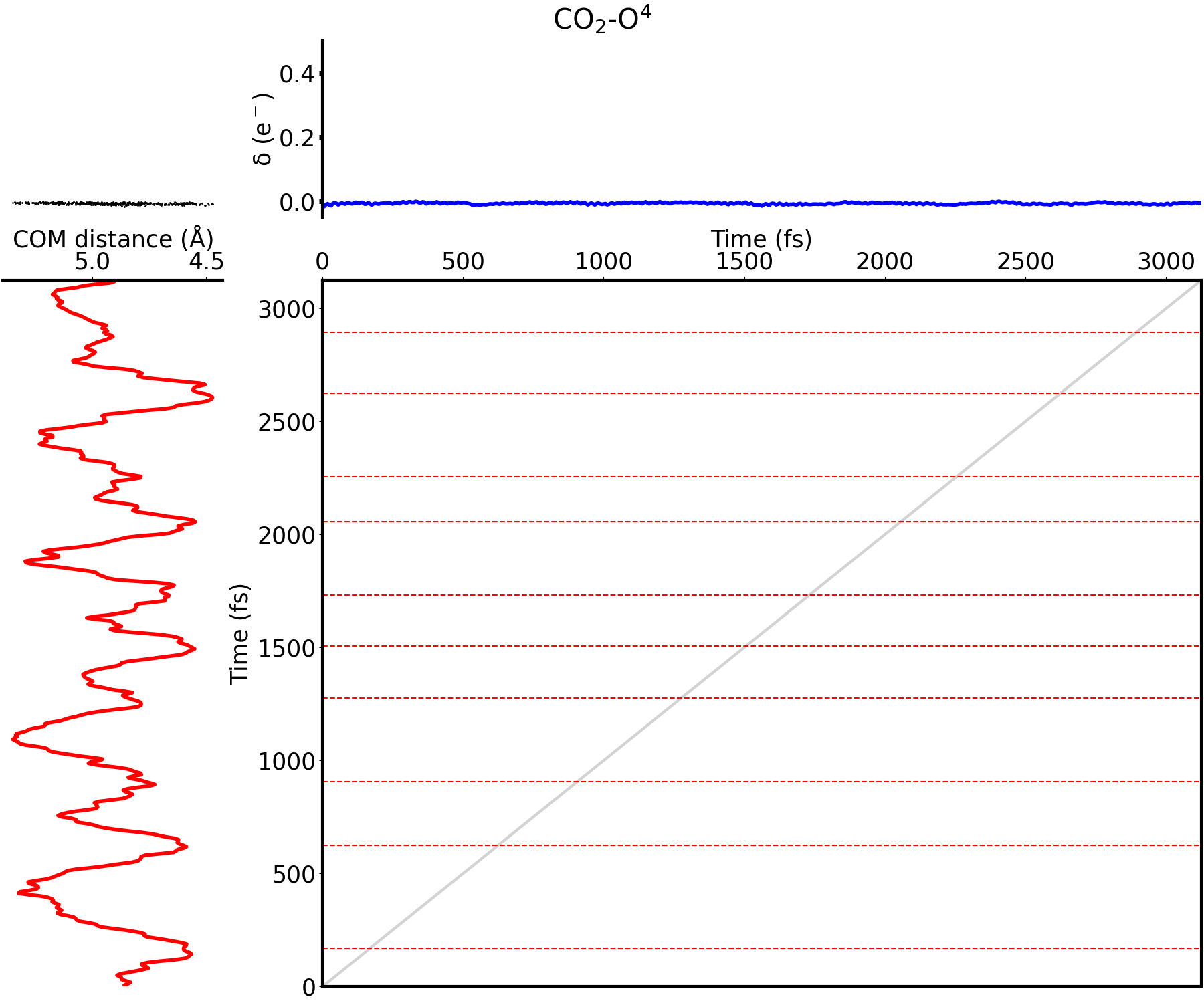

Supplement: Supplementary file 4 — Source Data [file 41467_2024_48567_MOESM4_ESM.zip › SI/Supplementary_Note_14/Supplementary_Figure_27/CO2_O4_corrmap.png]

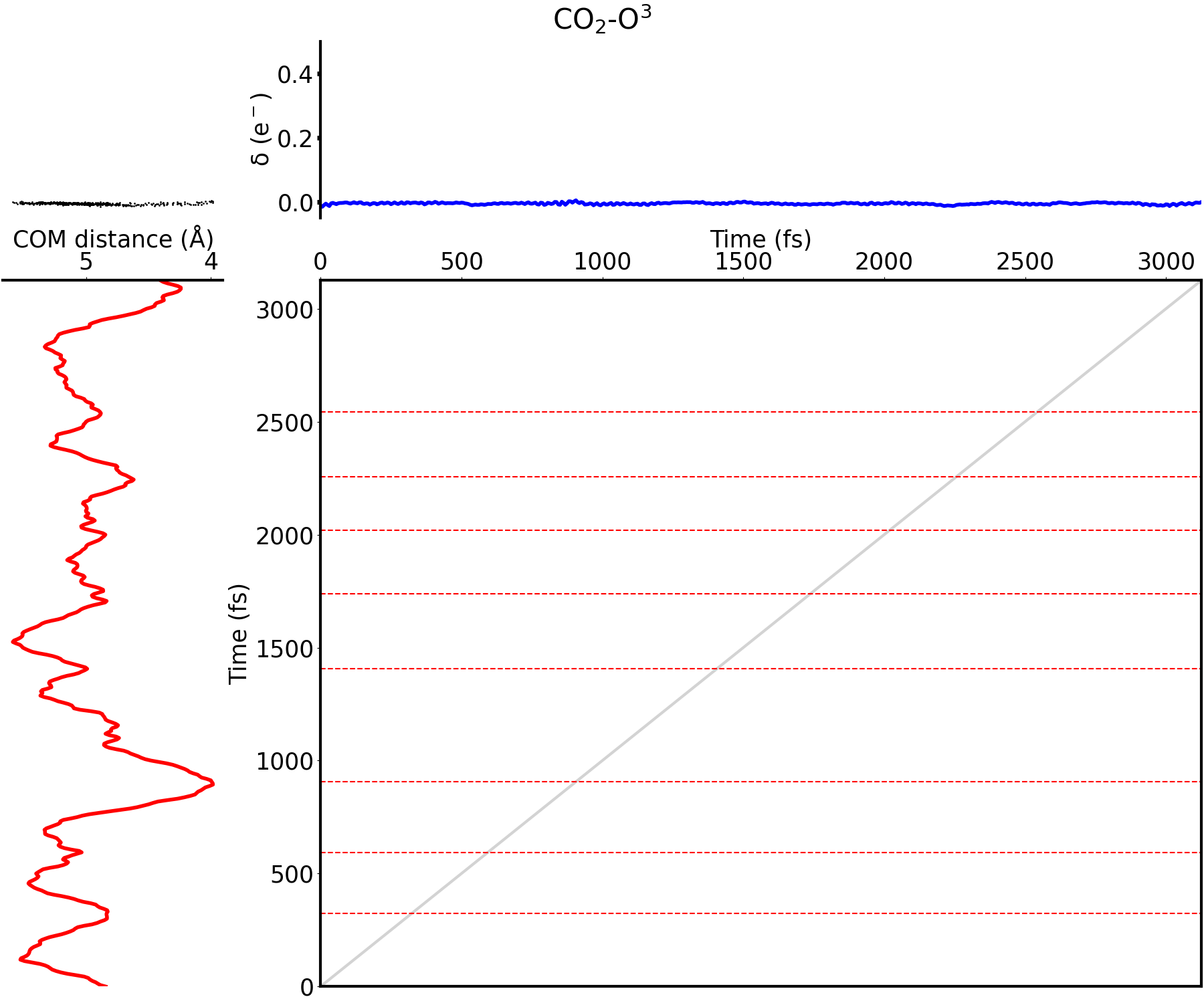

Supplement: Supplementary file 4 — Source Data [file 41467_2024_48567_MOESM4_ESM.zip › SI/Supplementary_Note_14/Supplementary_Figure_27/CO2_O3_corrmap.png]

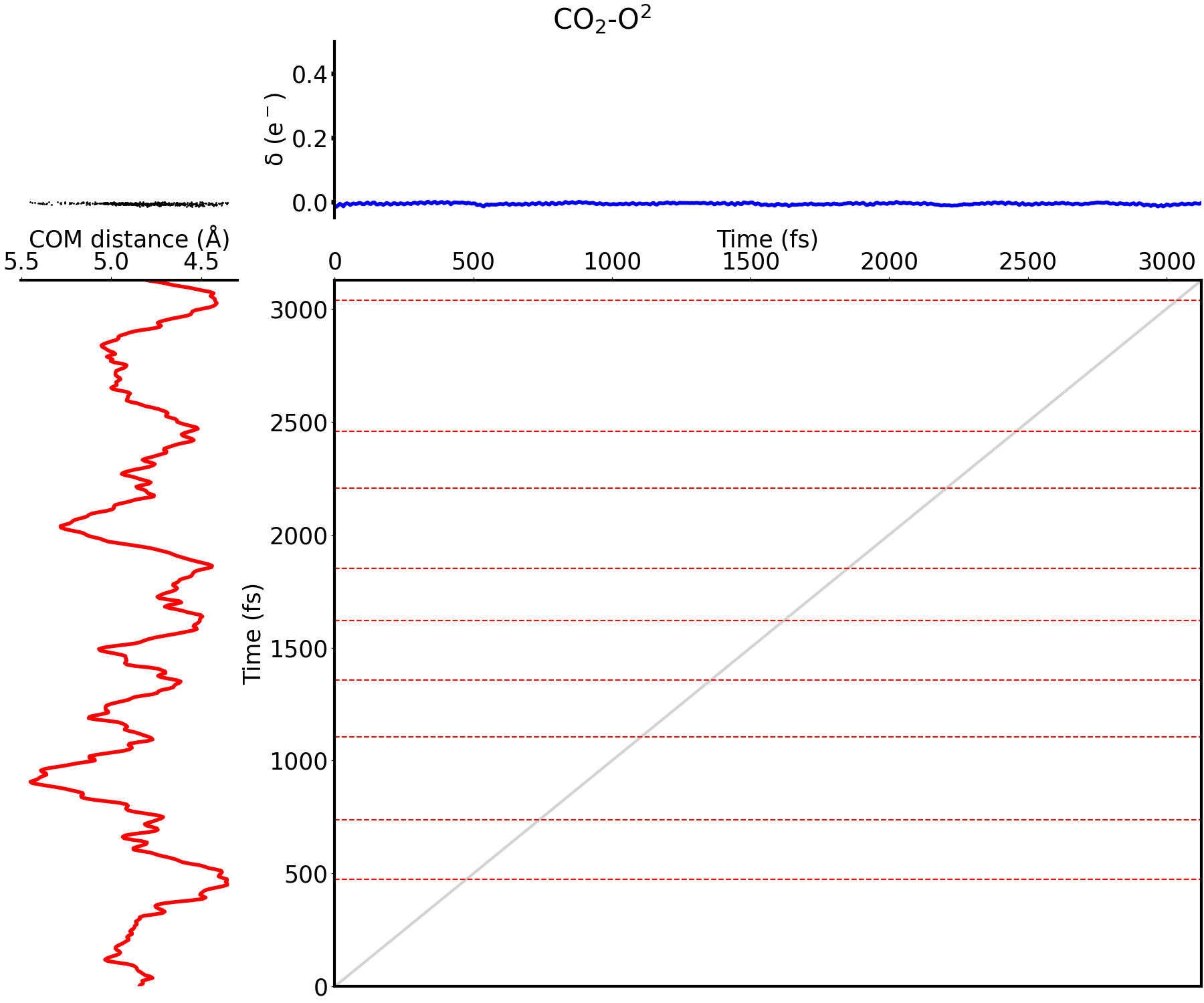

Supplement: Supplementary file 4 — Source Data [file 41467_2024_48567_MOESM4_ESM.zip › SI/Supplementary_Note_14/Supplementary_Figure_27/CO2_O2_corrmap.png]

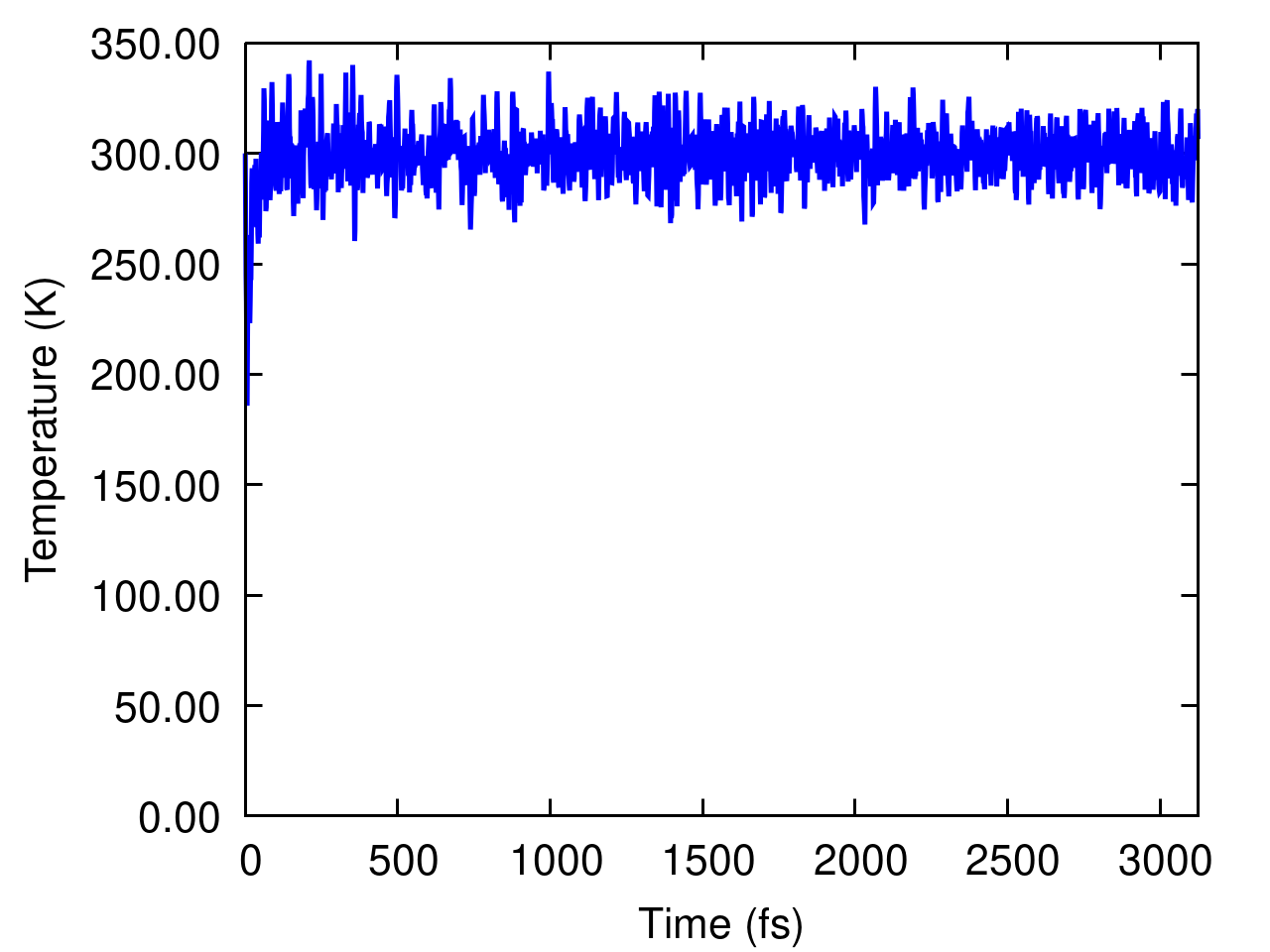

Supplement: Supplementary file 4 — Source Data [file 41467_2024_48567_MOESM4_ESM.zip › SI/Supplementary_Note_14/Supplementary_Figure_22/13Ptemp_HF3C.png]

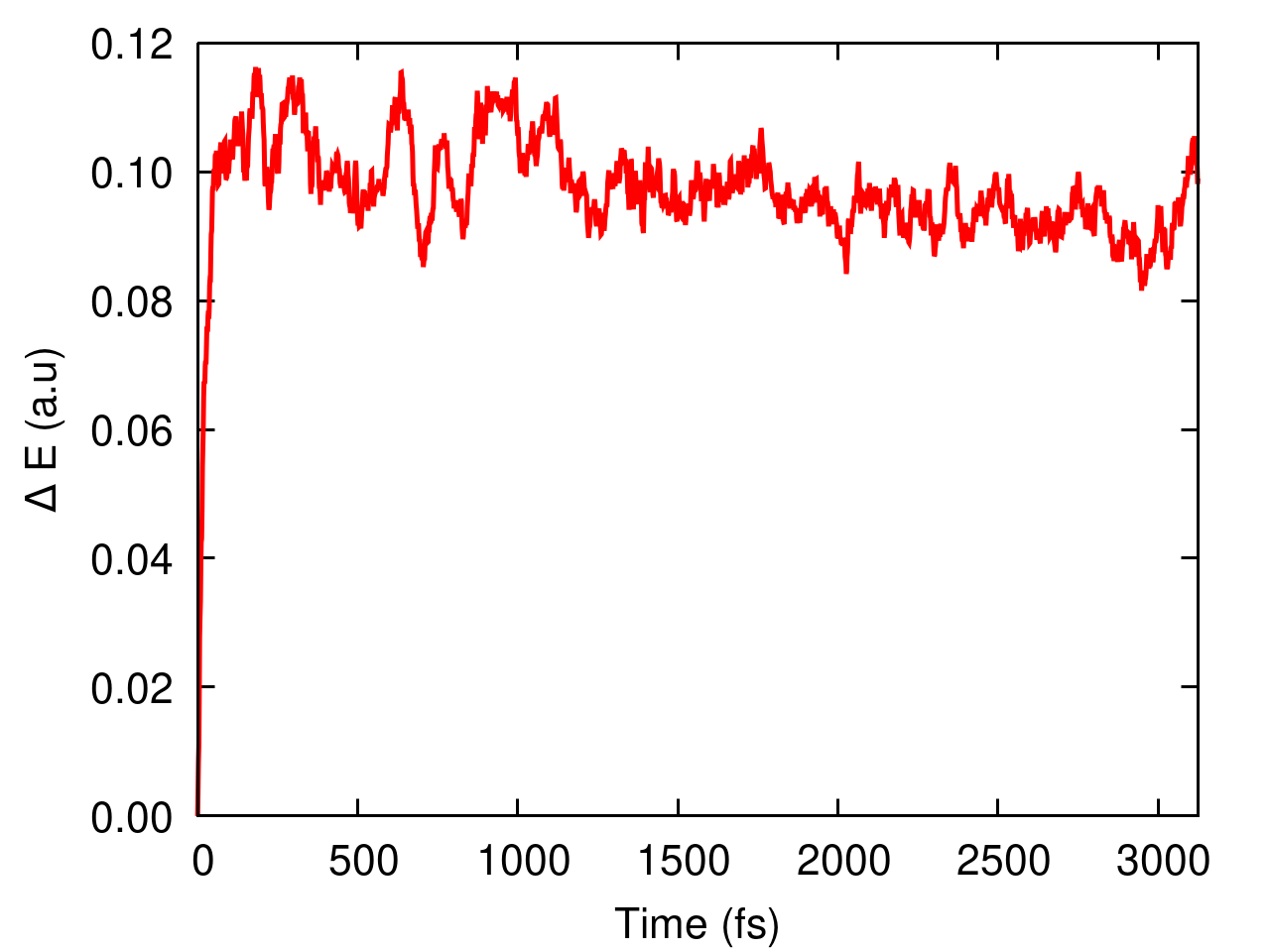

Supplement: Supplementary file 4 — Source Data [file 41467_2024_48567_MOESM4_ESM.zip › SI/Supplementary_Note_14/Supplementary_Figure_22/13Penergy_HF3C.png]

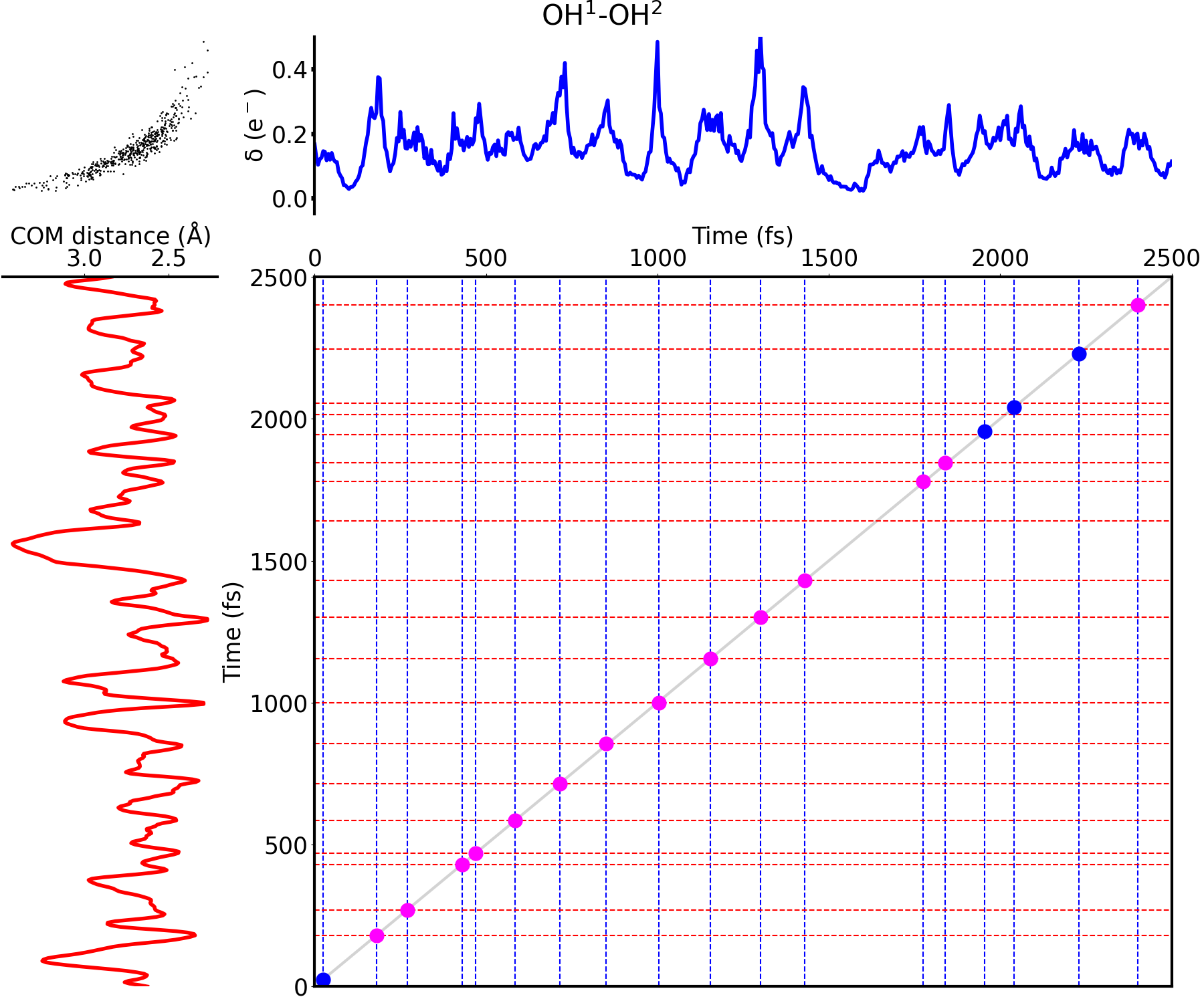

Supplement: Supplementary file 4 — Source Data [file 41467_2024_48567_MOESM4_ESM.zip › SI/Supplementary_Note_14/Supplementary_Figure_36/HT_OH1_OH2_corrmap.png]

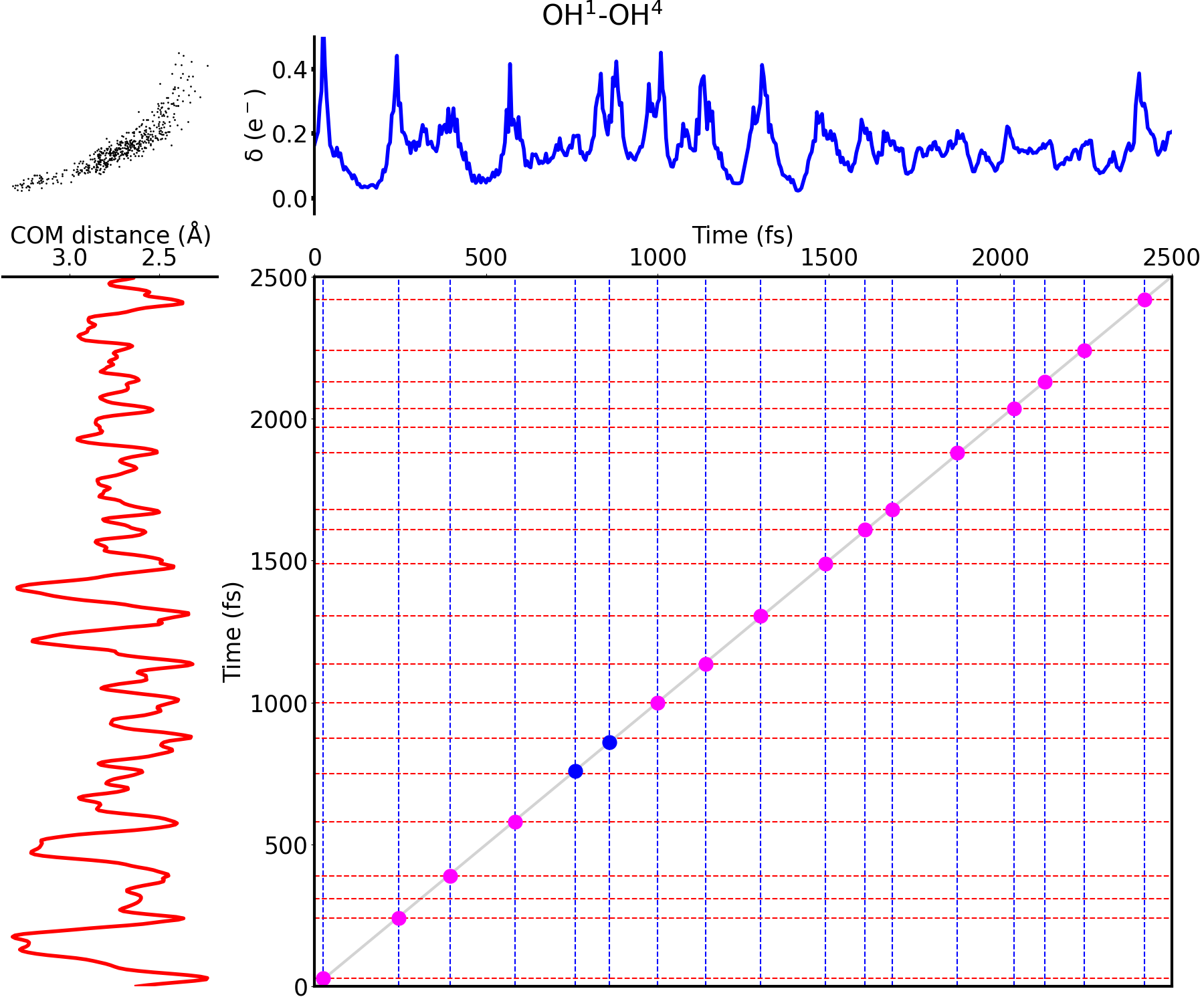

Supplement: Supplementary file 4 — Source Data [file 41467_2024_48567_MOESM4_ESM.zip › SI/Supplementary_Note_14/Supplementary_Figure_36/HT_OH1_OH4_corrmap.png]

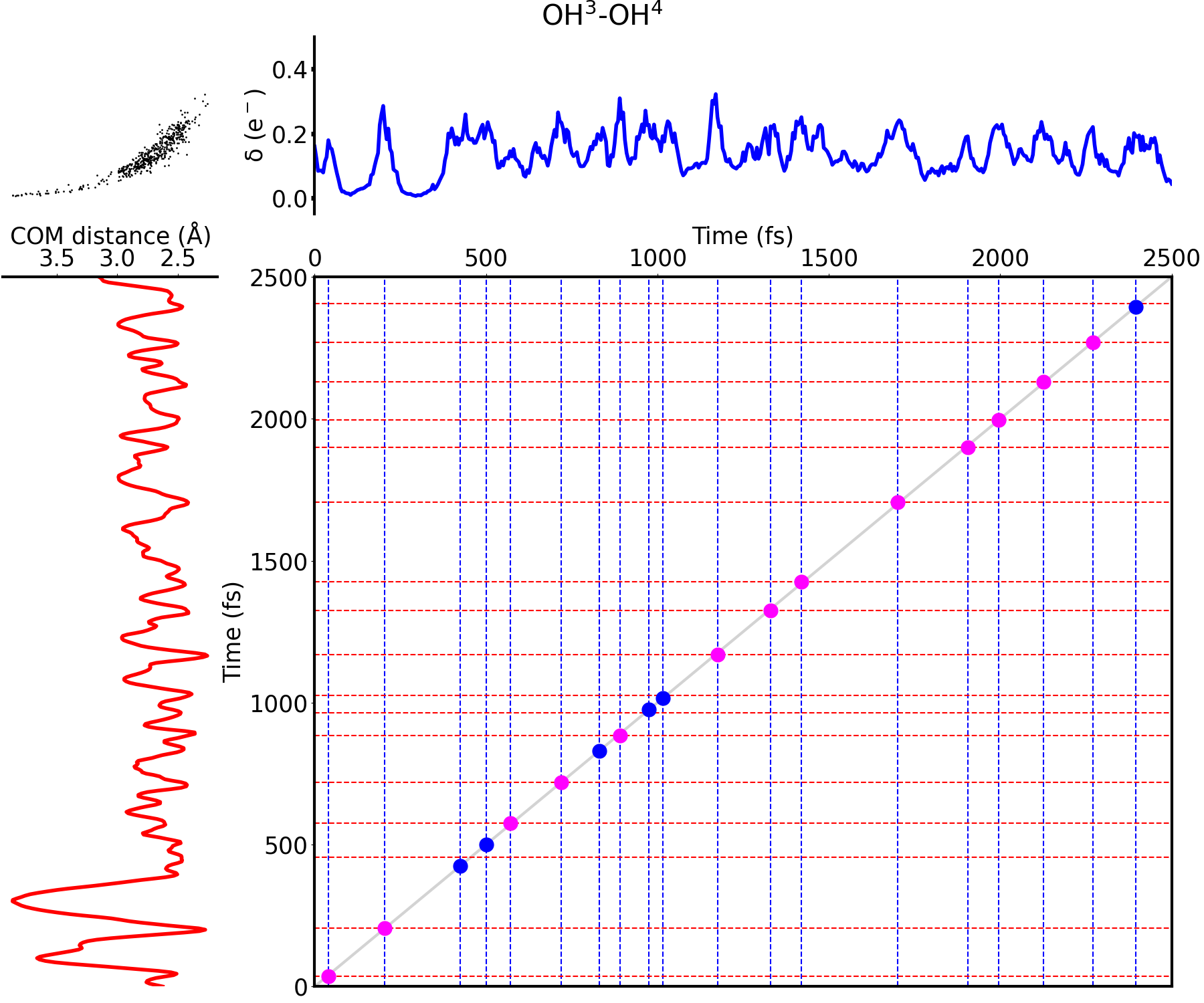

Supplement: Supplementary file 4 — Source Data [file 41467_2024_48567_MOESM4_ESM.zip › SI/Supplementary_Note_14/Supplementary_Figure_36/HT_OH3_OH4_corrmap.png]

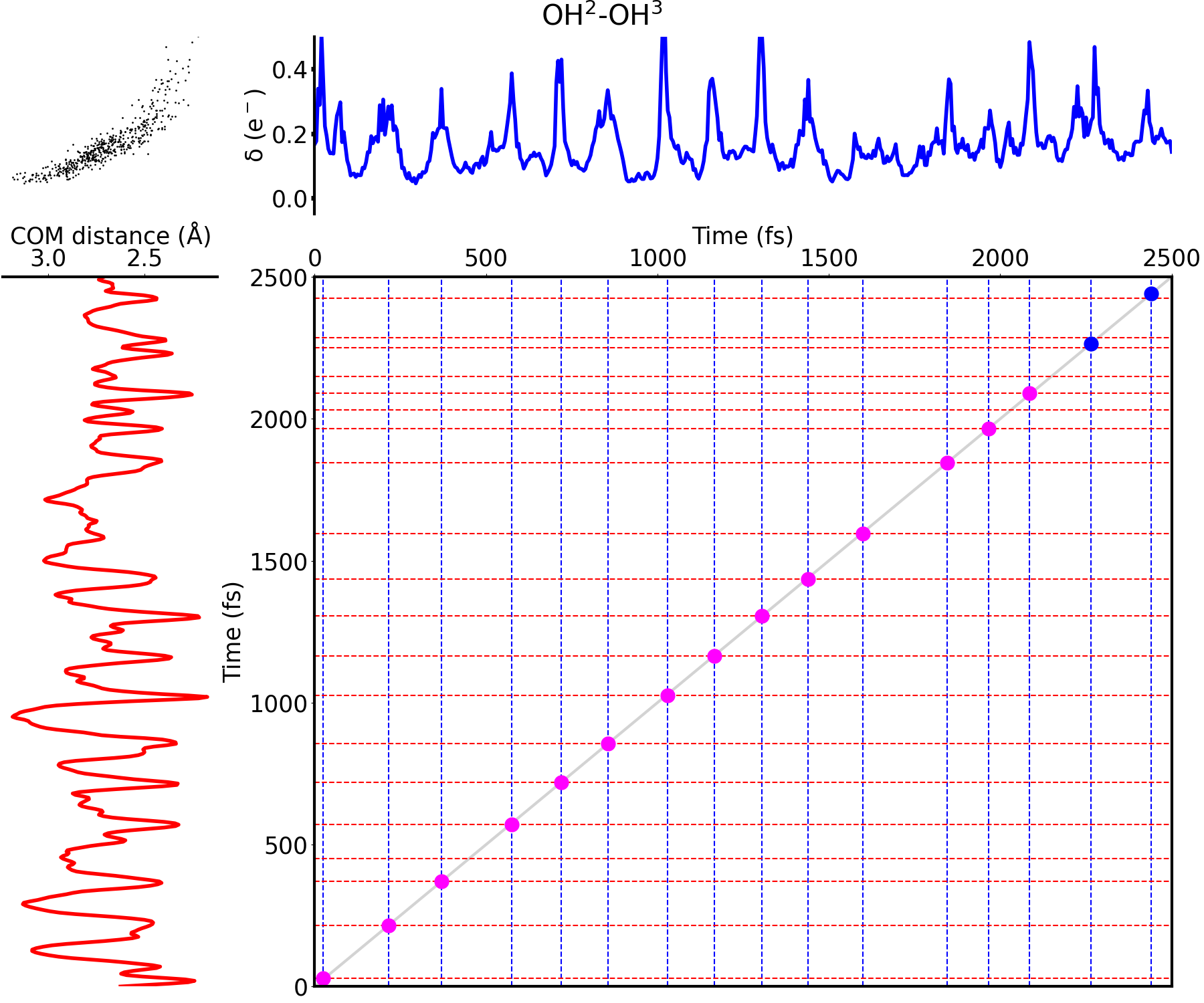

Supplement: Supplementary file 4 — Source Data [file 41467_2024_48567_MOESM4_ESM.zip › SI/Supplementary_Note_14/Supplementary_Figure_36/HT_OH2_OH3_corrmap.png]

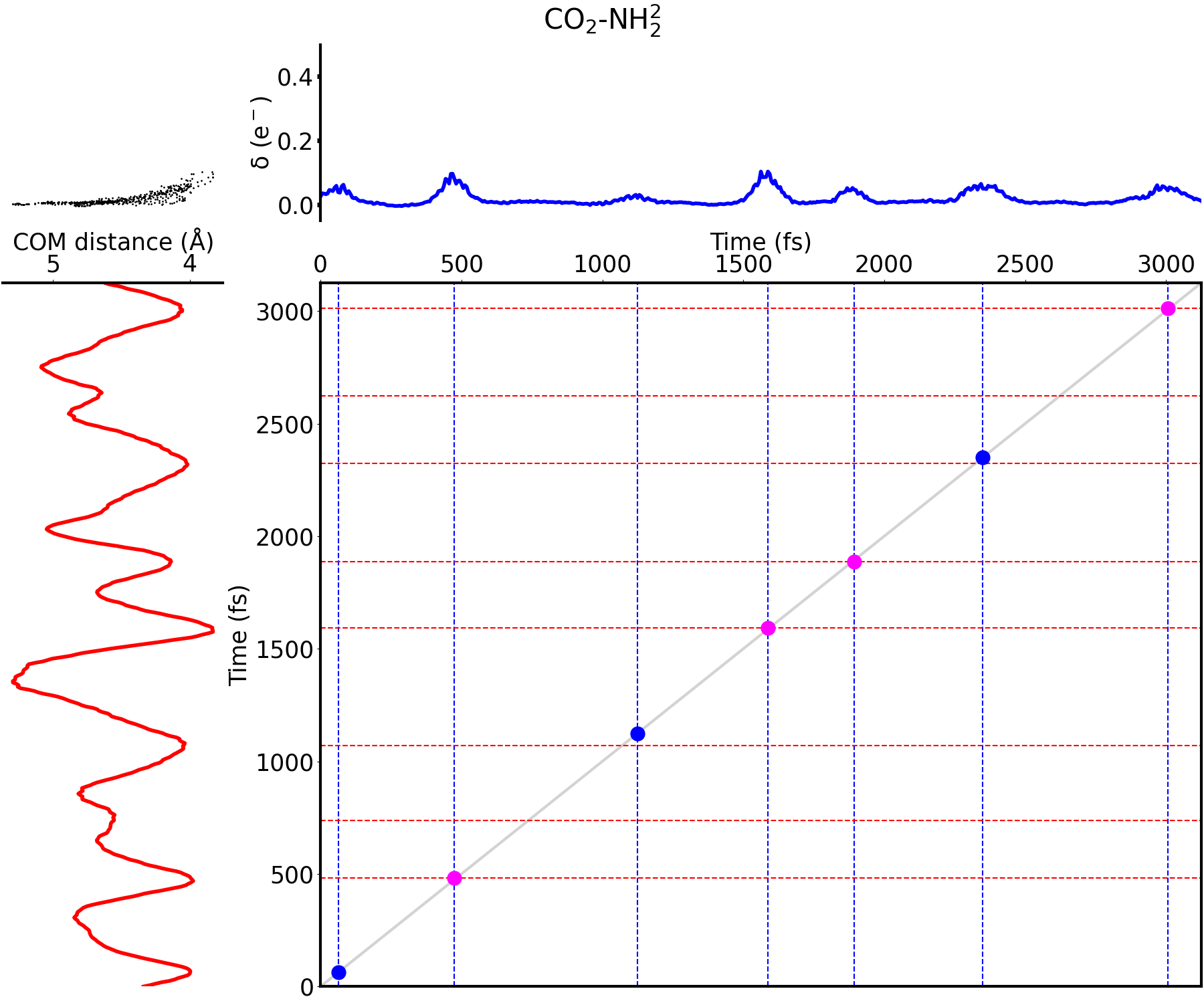

Supplement: Supplementary file 4 — Source Data [file 41467_2024_48567_MOESM4_ESM.zip › SI/Supplementary_Note_14/Supplementary_Figure_26/CO2_NH22_corrmap.png]

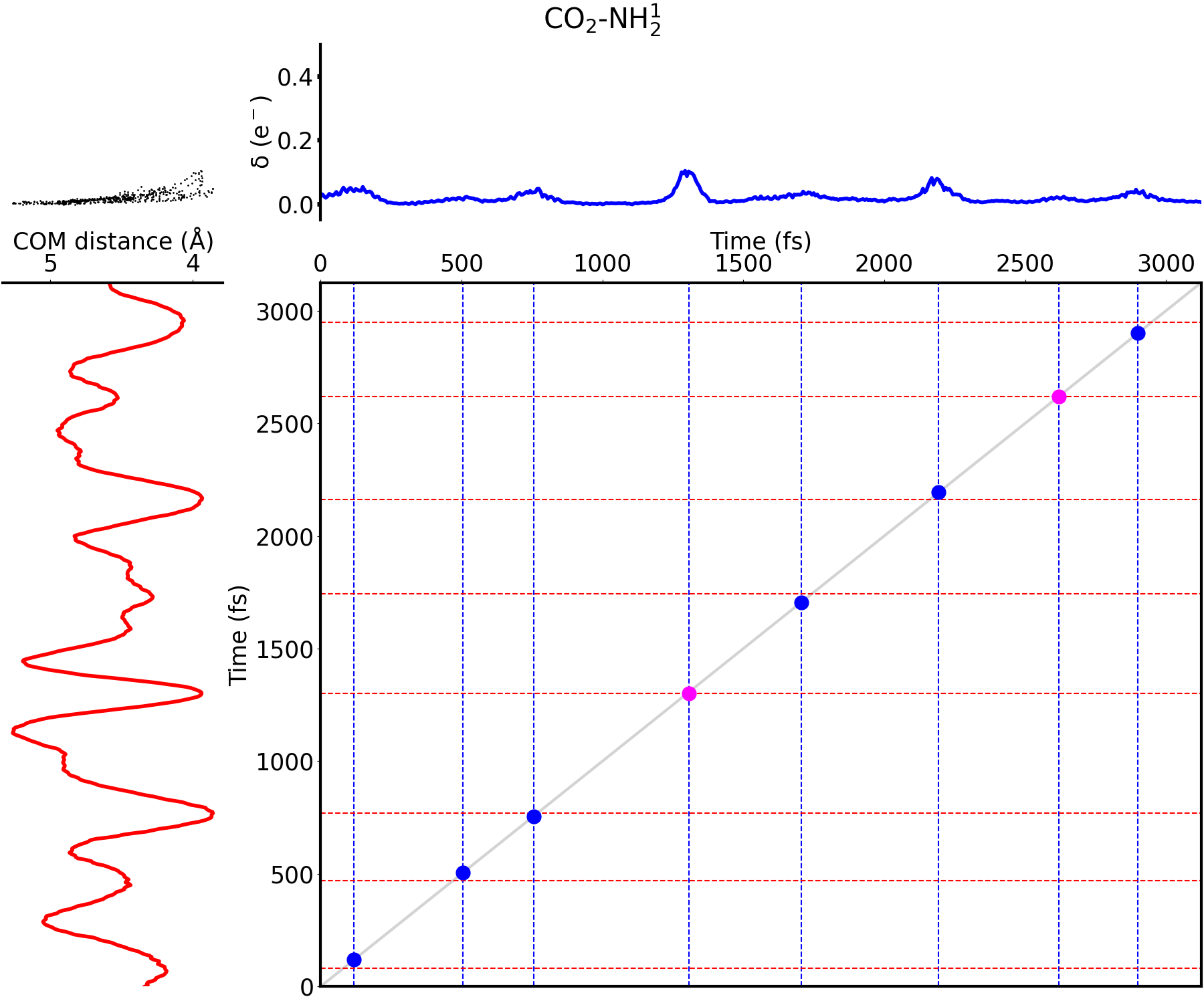

Supplement: Supplementary file 4 — Source Data [file 41467_2024_48567_MOESM4_ESM.zip › SI/Supplementary_Note_14/Supplementary_Figure_26/CO2_NH21_corrmap.png]
